# A System Pharmacology Model for Decoding the Synergistic Mechanisms of Compound Kushen Injection in Treating Breast Cancer

**DOI:** 10.3389/fphar.2021.723147

**Published:** 2021-11-16

**Authors:** Yi Li, Kexin Wang, Yupeng Chen, Jieqi Cai, Xuemei Qin, Aiping Lu, Daogang Guan, Genggeng Qin, Weiguo Chen

**Affiliations:** ^1^ Department of Radiology, Nanfang Hospital, Southern Medical University, Guangzhou, China; ^2^ Institute of Integrated Bioinformedicine and Translational Science, Hong Kong Baptist University, Hong Kong SAR, China; ^3^ Neurosurgery Center, Guangdong Provincial Key Laboratory on Brain Function Repair and Regeneration, Department of Cerebrovascular Surgery, Engineering Technology Research Center of Education Ministry of China on Diagnosis and Treatment of Cerebrovascular Disease, Zhujiang Hospital, Southern Medical University, Guangzhou, China; ^4^ Department of Biochemistry and Molecular Biology, School of Basic Medical Sciences, Southern Medical University, Guangzhou, China; ^5^ Guangdong Key Laboratory of Biochip Technology, Southern Medical University, Guangzhou, China; ^6^ Modern Research Center for Traditional Chinese Medicine, Shanxi University, Taiyuan, China

**Keywords:** breast cancer, compound kushen injection, traditional Chinese medicine, molecular docking, synergistic mechanism, system pharmacology

## Abstract

Breast cancer (BC) is one of the most common malignant tumors among women worldwide and can be treated using various methods; however, side effects of these treatments cannot be ignored. Increasing evidence indicates that compound kushen injection (CKI) can be used to treat BC. However, traditional Chinese medicine (TCM) is characterized by “multi-components” and “multi-targets”, which make it challenging to clarify the potential therapeutic mechanisms of CKI on BC. Herein, we designed a novel system pharmacology strategy using differentially expressed gene analysis, pharmacokinetics synthesis screening, target identification, network analysis, and docking validation to construct the synergy contribution degree (SCD) and therapeutic response index (TRI) model to capture the critical components responding to synergistic mechanisms of CKI in BC. Through our designed mathematical models, we defined 24 components as a high contribution group of synergistic components (HCGSC) from 113 potentially active components of CKI based on ADME parameters. Pathway enrichment analysis of HCGSC targets indicated that *Rhizoma Heterosmilacis* and *Radix Sophorae Flavescentis* could synergistically target the PI3K-Akt signaling pathway and the cAMP signaling pathway to treat BC. Additionally, TRI analysis showed that the average affinity of HCGSC and targets involved in the key pathways reached -6.47 kcal/mmol, while *in vitro* experiments proved that two of the three high TRI-scored components in the HCGSC showed significant inhibitory effects on breast cancer cell proliferation and migration. These results demonstrate the accuracy and reliability of the proposed strategy.

## Introduction

Breast cancer (BC) is the most common cancer in women worldwide and is responsible for the second highest death rate among female patients with cancer (Siegel et al., 2020). Many options are available to treat breast cancer, such as surgical treatment (Zheng et al., 2020), radiation therapy (Balaji et al., 2016), neoadjuvant endocrine therapy (Yao et al., 2019), neoadjuvant chemotherapy (Vaidya et al., 2018), and anti-HER2 therapy (Yao and Fu, 2018). However, side effects of these treatments are often observed. For example, radiation therapy can cause radiation-induced fibrosis (Straub et al., 2015) and radiodermatitis (Singh et al., 2016). Fatigue, pain, and systemic side effects can occur as a result of surgical treatment (Enien et al., 2018). Adjuvant endocrine therapy can cause vasomotor symptoms and musculoskeletal and vulvovaginal symptoms (Condorelli and Vaz-Luis, 2018). These side effects mainly influence the organs and blood system, which aggravates the psychological burden on the patients. Side effect symptoms include loss of appetite, vomiting, diarrhea, nausea, and ulcers (Meng et al., 2017; Recht, 2017; Cochran et al., 2019). In recent years, traditional Chinese medicine (TCM) has become increasingly popular in the treatment of BC. *Ganoderma lucidum* from *Ganoderma* suppresses the proliferation and migration of breast cancer by inhibiting Wnt/β-catenin signaling (Zhang, 2017), while puerarin from *Radix puerariae* inhibits cell migration, invasion, and adhesion of LPS-induced BC by blocking NF-κB and Erk pathways (Liu et al., 2017). In addition to these herbal components, many formulas and proprietary Chinese medicines are widely used in the treatment of BC, such as compound kushen injection (CKI) (Liu et al., 2020), Shugan Jianpi decoction (Jingyuan et al., 2021), and Fangjihuangqi decoction (Guo et al., 2020). Based on these formulas and proprietary Chinese medicines, CKI is a commonly used antitumor treatment in clinical practice.

CKI is a TCM formulae extract from kushen (*Radix Sophorae flavescentis*) and baituling (*Rhizoma Heterosmilacis*) at a ratio of 7:3, in which exist hundreds of components including alkaloids and flavonoids (Cui et al., 2020). As approved by the China Food and Drug Administration, CKI can be employed for cancer treatment (Guo et al., 2015) and has several pharmacological functions, including anticancer properties, hemostasis, and immunity enhancement (Wang et al., 2015). CKI is widely being used in the treatment of cancers, such as breast cancer (Xu et al., 2011a; Qu et al., 2016; Cui et al., 2019; Nourmohammadi et al., 2019; Cui et al., 2020), acute myeloid leukemia (Jin et al., 2018), and hepatocellular carcinoma (Gao et al., 2018; Yang et al., 2021). Additionally, the function of CKI in treating breast cancer was also proved in *in vitro* and *in vivo* experiments. CKI could reduce the tumor formation rates and tumor volume by the downregulated Wnt/b-catenin pathway in the MCF-7 SP xenograft model (Xu et al., 2011a). Besides, CKI could impair the migration and invasiveness of MDA-MB-231 cell lines (Nourmohammadi et al., 2019) and influence the cell cycle of MDA-MB-231 cell lines in promoting cell death by increasing the proportion of cells in the G1 phase and decreasing the cells in the S and G2/M phase (Cui et al., 2019; Cui et al., 2020), while it could inhibit proliferation and induce cell apoptosis of MCF-7 cell lines (Qu et al., 2016). These *in vitro* and *in vivo* experiments had proved that CKI has anticancer pharmacological function, especially in breast cancer. In CKI, matrine, oxymatrine, and sophocarpine are the main components of *Radix Sophorae flavescentis*, which display antitumor, anti-inflammatory, and antiviral properties, as well as cardiovascular protective abilities (Wang et al., 2015). *Rhizoma Heterosmilacis* may play a role in promoting oxidative stress–induced apoptosis (Liu et al., 2017), reducing oxidative stress (Hong et al., 2014), deoxidation, and dampness relief (Liang et al., 2019). Increasing evidence has shown that CKI can be used to halt cancer migration (Nourmohammadi et al., 2019), reduce anticancer drug resistance (Hy et al., 2014), increase cancer cell apoptosis (Qu et al., 2016), suppress the cancer cell cycle (Cui et al., 2019), and inhibit cancer progression (Wang et al., 2019). Matrine upregulates Bax and downregulates Bcl-2 to inhibit proliferation and increase apoptosis of breast cancer cells (Li et al., 2015) and further downregulates the canonical pathway to suppress human breast cancer stem-like cells (Xu et al., 2011b). Oxymatrine exerts its effects by blocking the cell cycle, initiating apoptosis (Binggang et al., 2002) and suppressing the epithelial–mesenchymal transition (Jiaqin et al., 2018). These sporadic reports suggest that different components of CKI play a role in the treatment of BC, but there is still a lack of systematic and overall research on the synergistic mechanism of different components of *Rhizoma Heterosmilacis* and *Radix Sophorae Flavescentis* in CKI.

Since TCM is characterized by “multi-components” and “multi-targets”, it is difficult to reveal the associated mechanisms between herbs, components, genes, and disease through traditional experimental methods. As an effective tool to elucidate the synergistic and potential mechanisms of the networks between component–target and target–disease, systemic pharmacology provides a new perspective on the therapeutic mechanisms of TCM in the treatment of BC at the systematic level.

**FIGURE 1:**
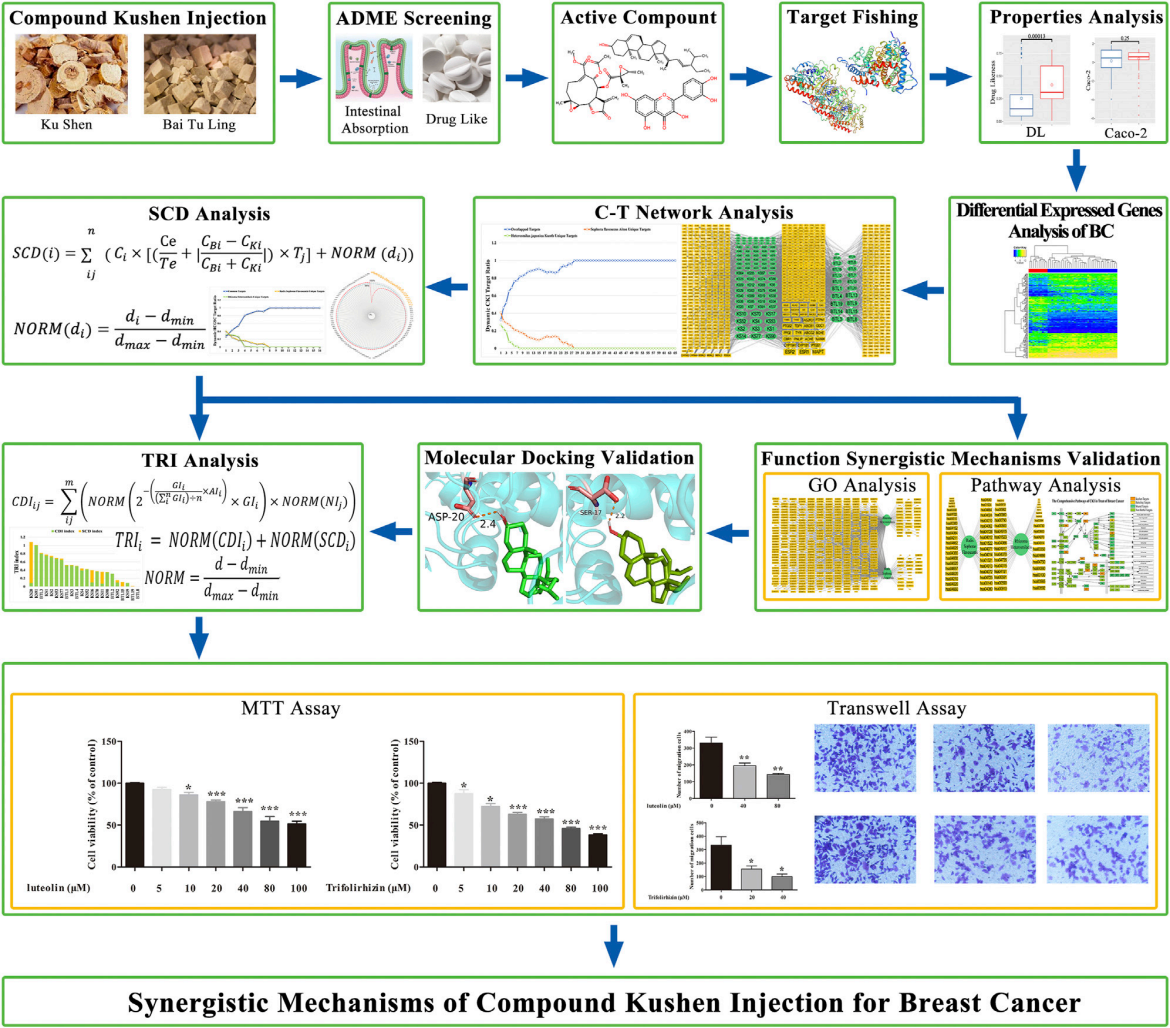
Workflow of the system pharmacology approach.

To explore the therapeutic mechanism of CKI in treating BC, a novel system pharmacology strategy (Figure 1), which integrates differentially expressed gene analysis (DEGs), pharmacokinetics synthesis screening, target identification, synergy contribution degree (SCD), network analysis, and therapeutic response index (TRI) calculation, was developed. The Cancer Genome Atlas (TCGA) database was used to explore the DEGs in BC. Subsequently, previously proposed absorption, distribution, metabolism, and excretion (ADME) screening models were employed to select potential active components; online tools were used to predict the targets of these potential active components, and a component–target (C-T) network was used for further network analysis. Network analysis of potential components combined with SCD was used to simulate the treatment effects of each component of CKI in BC and to explore a high contribution group of synergistic components (HCGSC). The virtual docking-aided TRI model was designed to determine whether highly reliable components may have a potential synergistic mechanism. Thereafter, GO enrichment and KEGG pathway enrichment analysis of BC, CKI, and highly reliable components in HCGSC were discussed to decode the potential synergistic mechanism analysis of CKI in the treatment of BC based on HCGSC. Ultimately, experimental validation was used to confirm the effect of high TRI-scored components in the HCGSC and evaluate the reliability of our model. We hope that these results will provide a strategy to reveal the therapeutic mechanism of TCM at the molecular level.

## Materials and Methods

### Chemical Components Collected From Database

All CKI components were collected from the Traditional Chinese Medicine Systems Pharmacology (TCMSP) database (http://www.tcmspw.com/tcmsp.php). TCMSP includes information from DrugBank, HIT, TTD, and PharmGKB. The pharmacokinetic properties of TCMSP include molecular weight (MW), AlogP, number of acceptor atoms for H-bonds (nHacc), number of donor atoms for H-bonds (nHdon), Caco-2 permeability (Caco-2), oral bioavailability (OB), drug-likeness (DL), blood–brain barrier (BBB), FASA-, and half-life (HL).

### ADME Screening

In the process of modern drug development, many drug candidates fail during development because of inadequate ADME properties (Rojas-Aguirre and Medina-Franco, 2014). Therefore, evaluating the ADME of drug components in the early stages has become an essential process. Components with better pharmacokinetic properties can be obtained by ADME screening, and the potential drug–drug interactions can be minimized (Wang et al., 2018). Two ADME-related models were employed in the present study, namely Caco-2 permeability and drug-likeness, to screen the potential active components of CKI (Supplementary Figure S1).

As a model system for intestinal epithelial permeability, the human colon carcinoma cell line (Caco-2) (Ij et al., 1989) is currently considered the gold standard (Jacobsen et al., 2020). The transport rates of components (nm/s) in Caco-2 monolayers represent intestinal epithelial permeability in TCMSP (Ru et al., 2014). Components with Caco-2 < −0.4 are not permeable; thus, Caco-2 > −0.4 were selected as candidate components.

Drug-likeness (DL) may be defined as a complex balance of various molecular properties and structural features that determine whether a particular molecule is similar to known drugs. The drug similarity index of the new compound was calculated using the Tanimoto coefficient, which is defined as

$$f\left(A,B\right)=\frac{A\times B}{{\left|A\right|}^{2}+{\left|B\right|}^{2}-A\times B}$$

In this equation, A represents the molecular descriptor of herbal components, and B is the average molecular property of all components in DrugBank (Tao et al., 2013). Based on the data from TCMSP, the average value of *Radix Sophorae Flavescentis* was 0.40, while that of *Rhizoma Heterosmilacis* was 0.25. Combined with other literature search criteria, we defined DL ≥ 0.18, as the screening criterion of DL.

### Target Identification

Three commonly used online tools were employed to identify the targets of active components in CKI, namely, similarity ensemble approach (SEA) (Keiser et al., 2007), HitPick (Liu et al., 2013), and SwissTargetPrediction (Gfeller et al., 2014). Open Babel 3.0.0 (O Boyle et al., 2011) was used to convert the SDF format of all CKI potential active components into the SMILES format. Potential active components in the SMILES format were imported to the SEA, HitPick, and SwissTargetPrediction to predict targets of potential active components. The homologous genes of other species provided by online tools were included in the discussion.

### Network Construction and Analysis

The C-T network was used as a frame to uncover the relationship between active components and targets. Cytoscape 3.8.0 (Shannon et al., 2003), an open-source software platform, was employed to visualize the networks.

### GO Enrichment and KEGG Pathway Enrichment Analysis

To analyze the main function of targets, clusterProfiler (Yu et al., 2012) in the Bioconductor package (https://bioconductor.org/) based on the R language was used for GO-BP enrichment analysis and KEGG pathway analysis. FDR-adjusted *p* values were set at 0.05, as the cut-off criterion.

### Synergy Contribution Degree Calculation

The SCD represents the contribution of a potential active component in the C-T network and its effectiveness in the treatment of BC. To evaluate the effect of CKI on the treatment of BC, we built a mathematical model to calculate the SCD of each active component in CKI:

$$\mathrm{SCD}\left(i\right)=\overset{n}{\underset{\mathrm{ij}}{\sum }}\left(C_{i}\times \left[\left(\frac{\mathrm{Ce}}{\mathrm{Te}}+\left|\frac{C_{\mathrm{Bi}}-C_{\mathrm{Ki}}}{C_{\mathrm{Bi}}+C_{\mathrm{Ki}}}\right|\right)\times T_{j}\right]+\mathrm{NORM}\left(d_{i}\right)\right),$$

$$\mathrm{NORM}\left(d_{i}\right)=\frac{d_{i}-d_{\min}}{d_{\max}-d_{\min}}.$$

In this equation, *i* is the number of components, and *j* is the number of targets. *C* represents the betweenness centrality of each component. *T* represents the betweenness centrality count of all targets of each component. *Ce* represents the edge count of each component. *Te* represents the edge count of all targets of each component. *C*_*B*_ represents the betweenness centrality of each component only in *Rhizoma Heterosmilacis*, and *C*_*K*_ represents the betweenness centrality of each component only in *Radix Sophorae Flavescentis*. If a component consists only of *Radix Sophorae Flavescentis* or *Rhizoma Heterosmilacis*, $\left|\frac{C_{Bi}-C_{Ki}}{C_{Bi}+C_{Ki}}\right|$ should be 1. If $\left|\frac{C_{Bi}-C_{Ki}}{C_{Bi}+C_{Ki}}\right|$ is zero, we assign 1e-6 to it. *d* represents the dose of each component in CKI, which was extracted using HPLC (Supplementary Table S1). *NORM (di)* is the index of min-max normalization to the dose of each component. The betweenness centrality of each component and target was calculated using Cytoscape version 3.8.0. based on the C-T network of the potential active components.

### Breast Cancer Differentially Expressed Genes Analysis

The TCGA database (https://portal.gdc.cancer.gov/) was used to explore the DEGs in BC. We used TCGAbiolinks (Colaprico et al., 2016) in the Bioconductor packages to access the HTSeq-Counts number of normal and BC samples from the TCGA-BRCA project of the TCGA program. DESeq (Anders and Huber, 2010) and limma (Ritchie et al., 2015) in the Bioconductor package were used to analyze the DEGs in BC. We set |log2FoldChange|≥2 and FDR adjusted the *p* value < 0.05, as the cut-off criterion. The results are presented and plotted using the gplots (Warnes et al., 2020) package.

### Molecular Docking Analysis of HCGSC in the Treatment of BC

The components of HCGSC were collected from the ZINC (Sterling and Irwin, 2015) and PubChem (https://pubchem.ncbi.nlm.nih.gov) databases in the MOL2 format. Human proteins were collected from the Protein Data Bank (PDB) (http://www.rcsb.org). AutoDock Tools (ADT) (Sanner, 1999) was used to pretreat the components and proteins. The pocket of the protein was automatically extracted from the space of the protein. AutoDock Vina (Trott and Olson, 2009) was used for docking. The seed of docking was 10,000, the energy range was 4, and the exhaustiveness was 4. The affinity (kcal/mol) index of each component–protein pair was used to estimate the docking results. The results were obtained using PyMOL (Schr Odinger, 2015).

### Therapeutic Response Index Model Calculation

We developed a mathematical model to calculate the therapeutic response index (TRI) of each component in a highly reliable docking relationship (HRDR) between HCGSC and its targets.

$$\mathrm{CD}I_{\mathrm{ij}}=\;\overset{m}{\underset{\mathrm{ij}}{\sum }}\left(\mathrm{NORM}\left(2^{-\left(\frac{GI_{i}}{\left(\sum_{i}^{n}GI_{i}\right)÷n}\times AI_{i}\right)}\times GI_{i}\right)\times \mathrm{NORM}\left(NI_{j}\right)\right),$$

$$\mathrm{TR}I_{i}\;=\mathrm{NORM}\left(\mathrm{CD}I_{i}\right)+\mathrm{NORM}\left(\mathrm{SC}D_{i}\right),$$

$$\mathrm{NORM}=\frac{d-d_{\min}}{d_{\max}-d_{\min}}.$$

In the above model, *i* is the number of components, *j* is the number of topology parameters, *n* is the collection of components, and *m* is the collection of components and topology parameters. CDI represents the docking index of HRDR. GI represents the number of genes targeted by each component. AI represents the average affinity of all component–protein binding relationships of each component. NI represents the C-T network topology parameters of HCGSC and their targets, including degree, betweenness centrality, closeness centrality, average shortest path length, eccentricity, radiality, neighborhood connectivity, and topological coefficient. SCD is the calculation result for each component from method 2.6. TRI represents the mechanism of CKI in the treatment of BC, which is the sum of normalized CDI and normalized SCD. NORM is a normalized equation.

## Materials

High-glucose Dulbecco’s modified Eagle medium (DMEM; 4.5 g/L), fetal bovine serum (FBS), 0.25% trypsin, and 3-(4,5-dimethylthiazol-2-yl)-2,5-diphenyltetrazolium bromide (MTT) were purchased from Shanghai Sangon Biotechnology Co., Ltd. (Shanghai, China). The transwell invasion chamber was purchased from Corning costar company (Andover, MA, United States). Matrigel was purchased from BD (Biosciences, Bedford, MA, United States). Luteolin and trifolirhizin (≥98% purity by HPLC) were obtained from Nanjing Jingzu Biotech Co., Ltd. (Nanjing, China).

### Cell Culture

The human breast cancer cell line MCF-7 was purchased from Procell Life Science & Technology Co., Ltd. Cells were cultured in an incubator at 37°C in DMEM containing 10% FBS. When the cells reached 80% confluency, they were exposed to different concentrations of luteolin and trifolirhizin (1, 2, and 4 mg/ml) for 24 h.

### Cell Viability Assay

MCF-7 cells (1 × 10^4^ cells/well) were seeded in 96-well plates and treated with 0, 5, 10, 20, 40, 80, and 100 μM luteolin and trifolirhizin for 24 h. MTT was added to a 96-well plate for 4 h, and the culture supernatant was removed. Finally, DMSO was used to dissolve the purple crystals. Absorbance at 570 nm was measured using a plate reader.

### Transwell Assay

Referring to our previous method (Gao et al., 2018), luteolin (40 and 80 μM) and trifolirhizin (20 and 40 μM) were added to the lower compartments. The migrating cells were observed under a microscope.

### Statistical Analysis

The R package ggpubr (Kassambara, 2020) was used to compare the molecular properties of all components in *Rhizoma Heterosmilacis* and *Radix Sophorae Flavescentis*. Data were analyzed using the Student’s *t*-test. Differences were considered statistically significant at *p* < 0.05.

## Results

Based on the systematic pharmacological model, the mechanism of CKI in BC treatment was clarified and validated. First, DEGs that could be used as potential pathogenic genes were determined based on the data from the TCGA-BRCA project. Second, the CKI components were collected from the TCMSP database, and the ADME method was used to screen active components in CKI. Third, the active components and their targets, which were predicted using three online tools, were used to construct the C-T network. Fourth, a network analysis of potential components combined with SCD was used to explore HCGSC. Thereafter, GO enrichment and KEGG pathway enrichment analysis of BC, CKI, and highly reliable components in HCGSC were discussed to decode the potential synergistic mechanism of CKI in the treatment of BC. Subsequently, the virtual docking-aided TRI model was designed to determine whether highly reliable components may have a synergistic mechanism. Ultimately, experimental validation was used to confirm the effect of high TRI-scored components in HCGSC and evaluate the reliability of our model.

### Differentially Expressed Genes Analysis of BC

To further analyze the mechanism of CKI in the treatment of BC, 113 normal breast samples and 1,102 breast cancer samples were extracted from the TCGA-BRCA project. A total of 979 DEGs were upregulated, and 990 were downregulated in cancer (Supplementary Table S2). The DEGs were used to determine the gene expression patterns of normal and tumor patients (Supplementary Figure S2). The results showed that the DEG pattern distinguished between diseased and normal states. From the expression pattern, we identified that the top 10 upregulated genes were *FTHL17, CSAG1, MUC2, COX7B2, CGA, CSAG4, CST4, MAGEA12, MAGEA1*, and *ACTL8*. Among these genes, *MUC2* influences proliferation, apoptosis, and metastasis of breast cancer cells (Astashchanka et al., 2019), while the upregulation of *MAGEA* in patients revealed a higher risk of recurrence (Otte et al., 2001). The top 10 downregulated genes were *LEP, GLYAT, AC087482.1, APOB, TRHDE-AS1, AQP7P2, FP325317.1, PLIN1, CA4*, and *AL845331.2*. *LEP* which inhibit apoptosis of breast cancer cells by coding leptin, while *PLIN1* inhibits invasion, migration, and proliferation of cells (Zhou et al., 2016; Crean-Tate and Reizes, 2018). The literature reports proved that these genes are related to the development of BC, which indicates that the DEGs were more likely to be pathogenic genes.

### Component Comparisons in *Rhizoma Heterosmilacis* and *Radix Sophorae Flavescentis*

**FIGURE 2:**
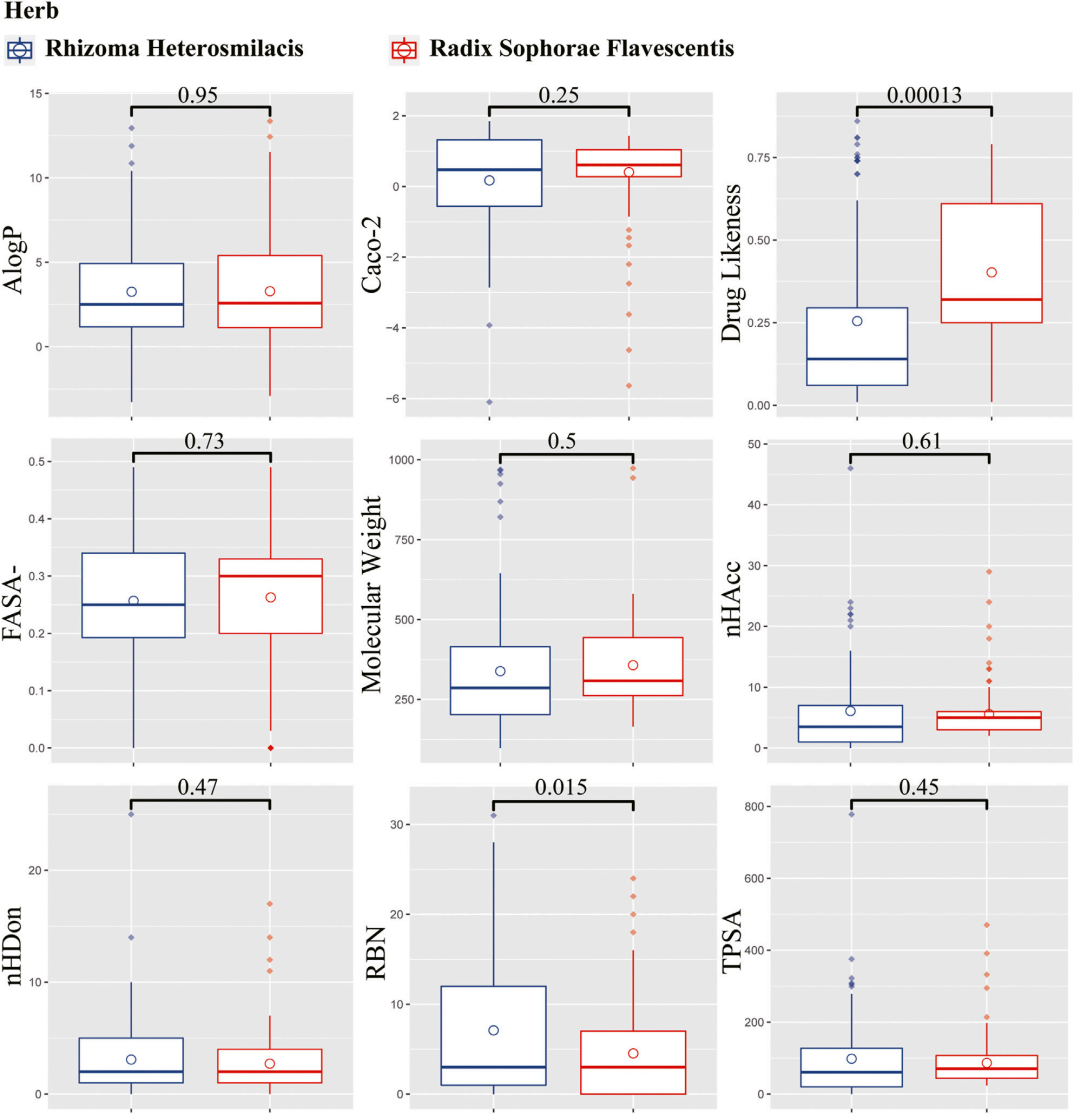
Boxplot of nine properties of the two components. The box in blue represents the value distribution of *Radix Sophorae Flavescentis*, and the box in red represents the value distribution of *Rhizoma Heterosmilacis*.

From the TCMSP database, 187 compounds were retrieved from *Rhizoma Heterosmilacis* (74) and *Radix Sophorae Flavescentis* (113). Detailed information on the components of *Rhizoma Heterosmilacis* and *Radix Sophorae Flavescentis* is provided in Supplementary Table S3. To further describe the differences between *Rhizoma Heterosmilacis* and *Radix Sophorae Flavescentis*, we compared nine properties of the two components, including drug likeness (DL), Caco-2, molecular weight (MW), AlogP, TPSA, FASA-, RBN, nHDon, and nHAcc, and drew boxplots using R packages ggplot2 (Wickham, 2016), ggpubr (Kassambara, 2020), and ggsci (Xiao, 2018). The results (Figure 2) showed most of chemical composition and properties between *Rhizoma Heterosmilacis* and *Radix Sophorae Flavescentis* had no statistical difference except for DL (*p* = 0.00013) and RBN (*p* = 0.015). These results indicate that *Rhizoma Heterosmilacis* and *Radix Sophorae Flavescentis* have similar chemical properties.

### Potential Active Components in *Rhizoma Heterosmilacis* and *Radix Sophorae Flavescentis*

Traditional Chinese medicine uses a combination of herbs, each of which usually contains hundreds of components, and only a few of these components possess satisfactory pharmacodynamic and pharmacokinetic properties. In this study, two ADME-related properties, DL and Caco-2, were used to screen for active components. Through ADME screening, 111 active components (*Radix Sophorae Flavescentis* 90 and *Rhizoma Heterosmilacis* 21) were selected from the 187 components of CKI. Additionally, some components, such as N-methylcytisine and trifolirhizin, did not pass the ADME screening, but were frequently reported in previous studies, so we added them to the list of active components (Xiumei and Cen, 2004; Juan et al., 2007; Yue, 2012; Liang et al., 2013). Therefore, 113 active components (*Radix Sophorae Flavescentis* 92 and *Rhizoma Heterosmilacis* 21) were included for further analysis. Detailed information is provided in Supplementary Table S4.

Through ADME screening and literature review, 92 potential components containing ideal pharmacokinetic profiles were selected from *Radix Sophorae Flavescentis*. For example, as one of the major components of CKI, sophoridine (KS18, DL = 0.25, Caco-2 = 1.13) displays antitumor, anti-allergic, anti-inflammatory, anti-arrhythmia, and antiviral properties, and affects the central nervous system (Han et al., 2009; Cuiping, 2010; Huang et al., 2014; Hu et al., 2016). Anagyrine (KS34, DL = 0.24, Caco-2 = 1.16) and (+)-lupanine (KS83, DL = 0.24, Caco-2 = 1.16) inhibit the proliferation and induction of apoptosis in human cervical cancer cells (Merghoub et al., 2011). Oxysophocarpine (KS89, DL = 0.29, Caco-2 = 1.042) suppressed cell proliferation, migration, invasion, and angiogenesis, induced cell cycle arrest, and enhanced apoptosis in oral squamous cell carcinoma (Liu et al., 2018). IDO1 expression can be downregulated by kushenol E (KS76, DL = 0.59, Caco-2 = 0.58) and kushenol F (KS74, DL = 0.61, Caco-2 = 0.45) to inhibit tumor proliferation (Kwon et al., 2019) and induce apoptosis (Kwon et al., 2020).

Among the 74 components in *Rhizoma Heterosmilacis*, 21 components met the screening criteria. For instance, diosgenin (BTL5, DL = 0.81, Caco-2 = 0.82), a steroid compound, has been shown to promote apoptosis and anticancer effects (Romero-Hernández et al., 2015; Jiang et al., 2016). Sitogluside (BTL1, DL = 0.62, Caco-2 = -0.14), beta-sitosterol (BTL2, DL = 0.75, Caco-2 = 1.32), sitosterol (BTL3, DL = 0.75, Caco-2 = 1.32), and stigmasterol (BTL4, DL = 0.75, Caco-2 = 1.45) can inhibit proliferation (Awad et al., 2000; Awad et al., 2003) and promote apoptosis (Awad et al., 2003), cell cycle arrest, and sphingomyelin cycle activation (Jia et al., 2018). Additionally, quercetin (BTL1, DL = 0.28, Caco-2 = 0.05) could be used to inhibit the proliferation of tumor cell lines (Ren et al., 2015) and induce apoptosis of tumor cells (Jia et al., 2018). Taxifolin (BTL13, DL = 0.27, Caco-2 = -0.23) inhibited proliferation, migration, and invasion of breast cancer cells by promoting EMT through β-catenin signal transduction (Li et al., 2019).

### Target Prediction and Analysis of *Rhizoma Heterosmilacis* and *Radix Sophorae Flavescentis*

To evaluate the effect of the active components of CKI, we used SEA, HitPick, and SwissTargetPrediction tools to predict the targets of 113 active components of CKI. Finally, 780 targets were obtained from these tools (Supplementary Table S5). To explore the mechanism of CKI in the treatment of BC, 113 active components and 780 targets were used to construct the C-T network (Supplementary Figure S3). The C-T network results showed that 252 of all 780 targets were overlapped by the target list of *Rhizoma Heterosmilacis* and *Radix Sophorae Flavescentis*, and 320 targets were unique to *Radix Sophorae Flavescentis*, while 208 targets were unique to *Rhizoma Heterosmilacis*. A total of 4,592 component–target associations between 113 active components and 780 targets were contained in the C-T network. The average number of targets per component is 40.64, and the mean number of components per target is 5.89, which shows that CKI has multi-component and multi-target characteristics in treating BC.

Among all components, six components displayed a number of degrees above 120, namely palmitone (BTL15, degree = 149), quercetin (BTL1, degree = 144), quercetin (KS3, degree = 144), norartocarpetin (KS77, degree = 130), apigenin (KS2, degree = 129), and luteolin (KS1, degree = 125), all of which exhibit anticancer functions (Imran et al., 2019), and suppress migration and EMT (Cao et al., 2020). These six high-degree components only accounted for 5.3% of all components but covered 58.3% of all common targets. They all targeted common targets with high degrees, including ESR1 (degree = 65), MAPT (degree = 65), and ESR2 (degree = 63). It is worth noting that the top 25 targets with the highest degree were all dropped in the common targets list between *Rhizoma Heterosmilacis* and *Radix Sophorae Flavescentis*, most of which were related to the pathogenesis or treatment of BC. As the coding genes of the estrogen receptor, methylation of the ESR1 (degree = 65) promoter may be associated with shorter survival time and increased risk of drug resistance to anti-endocrine therapy (Kirn et al., 2018) and is commonly targeted by oxysophocarpine (KS89), kushenol E (KS76), and kushenol F (KS74) of *Radix Sophorae Flavescentis* and Stigmasterol (BTL5), quercetin (BTL1), and taxifolin (BTL13) of *Rhizoma Heterosmilacis*. Additionally, MAPT (degree = 65) is correlated with microtubule assembly and stabilization (Weingarten et al., 1975), which can promote bicalutamide resistance and is associated with survival in prostate cancer (Sekino et al., 2020). Our data analysis showed that (+)-Lupanine (KS76) and leachianone,g (KS74) from *Radix Sophorae Flavescentis* and quercetin (BTL1) from *Rhizoma Heterosmilacis* could target MAPT. We noted that several components of *Rhizoma Heterosmilacis* and *Radix Sophorae Flavescentis* can both activate common targets to play their role in the treatment of BC. Thus, these results suggest that *Rhizoma Heterosmilacis* and *Radix Sophorae Flavescentis* may act synergistically to treat BC via the common targets based on the “multi-component” and “multi-target” features and provide therapeutic targets for the cooperative treatment of BC.

To further decode the synergistic mechanism between *Radix Sophorae Flavescentis* and *Rhizoma Heterosmilacis*, we investigated the change in common target proportion under the condition of different degrees as thresholds. The results showed that following the increase in degree, the proportion of common target retention maintained a high growth trend. The proportion of common targets increased from 32.31 to 100% when the degree threshold increased from 1 to 28. Additionally, when the degree threshold increased from 1 to 28, the proportion of unique targets of *Rhizoma Heterosmilacis* decreased from 26.76 to 0%; however, when the degree threshold increased from 1 to 8, the proportion of specific targets of *Radix Sophorae Flavescentis* decreased from 41.02 to 0%. The results indicated that the common targets of *Rhizoma Heterosmilacis* and *Radix Sophorae Flavescentis* have relatively high degrees. A higher degree indicates that these targets have a higher influence among all targets, and it also suggests that *Rhizoma Heterosmilacis* and *Radix Sophorae Flavescentis* exert major synergistic effects by targeting common targets, which have a greater influence on the intervention response network.

**FIGURE 3:**
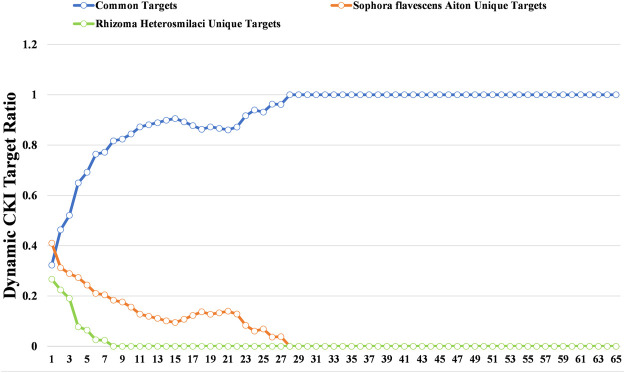
Dynamic ratio changes between common targets and unique targets of *Rhizoma Heterosmilacis* and *Radix Sophorae Flavescentis*.

To further decode the synergistic mechanism between them, we calculated the dynamic ratio change between common targets and unique targets of *Radix Sophorae Flavescentis* and *Rhizoma Heterosmilacis* using degree as the threshold. As shown in Figure 3, the proportion of common targets showed an overall upward trend from 32.31% (degree = 1) to 100% (degree = 28). However, the proportion of unique targets of *Rhizoma Heterosmilacis* and *Radix Sophorae Flavescentis* revealed a gradual downward trend. The ratio of unique targets of *Radix Sophorae Flavescentis* changed from 41.02 to 0% with a degree ranging from 1 to 28, while the ratio of unique targets of *Rhizoma Heterosmilacis* changed from 26.67 to 0% with a degree range from 1 to 7.

In the C-T network, nodes with a higher degree usually represent the importance of all nodes. The results indicated that too much background noise may obscure the synergistic mechanism. Therefore, a model should be used to extract the core components.

### Synergy Contribution Degree Calculation and Effect Verification

It is worth noting that *Rhizoma Heterosmilacis* and *Radix Sophorae Flavescentis* may play their roles through common targets based on the C-T network. However, the core component groups and mechanisms of synergy require further elucidation. To solve these problems, an SCD calculation method was designed that considers the synergy of both the topology of the components in the C-T network and the dose of the components. The SCD values of the active components of CKI are shown in Supplementary Table S6.

**FIGURE 4:**
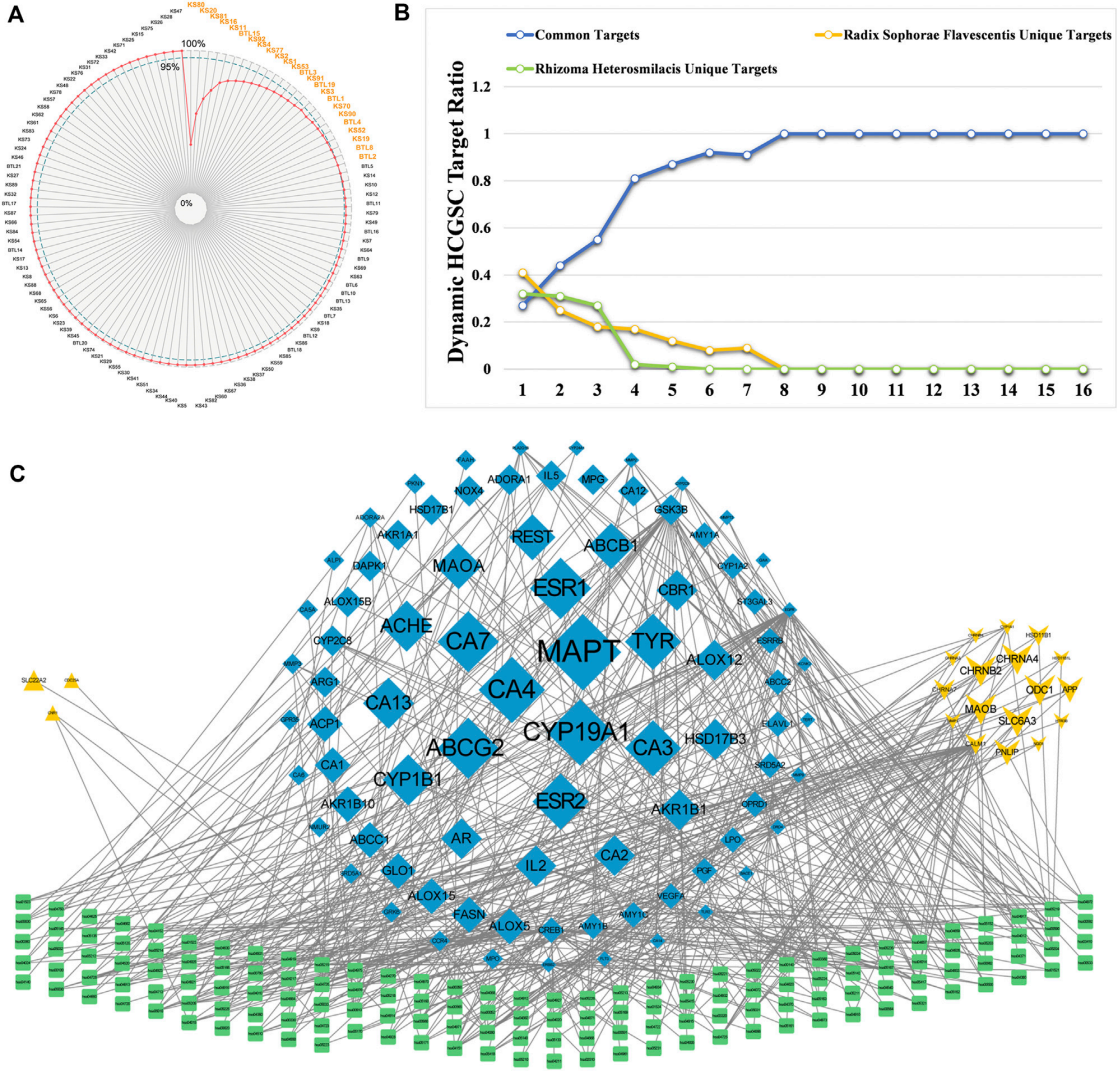
**(A)** Cumulative radar chart of the ratio between component accumulation targets and the targets of CKI. The label within the chart represented the ratio (%) between component accumulation targets and the targets of CKI. **(B)** The dynamic ratio changes between common targets of HCGSC and unique targets of *Radix Sophorae Flavescentis,* and *Rhizoma Heterosmilacis* of HCGSC. **(C)** The network with targets and their enriched pathways (the degrees of the targets were higher than the third quartile of the targets in the C-T network of HCGSC) and their enriched pathways.

According to the calculation results, the top two components with an SCD sum of 49.05% were oxymatrine (KS80) and matrine (KS20). Twenty-four components contribute to the effects of CKI on BC, with a total of 95.03%. Surprisingly, 24 components accounted for only 21.23% of all active components in CKI and could cover 631 targets (80.89% of all CKI targets) (Figure 4A). Thus, we define these 24 components as the high contribution group of synergistic components (HCGSC). These results show that HCGSC plays key roles in all active components of CKI and may clarify why the herbs in CKI generate synergistic and combination effects on BC.

To evaluate the reliability of HCGSC, we defined two references for further comparison: the first reference was 70 genes, which overlapped with DEGs and CKI targets, and the second reference was the 17 pathways, which are overlapped by enriched DEGs pathways and CKI targets. The targets of HCGSC contained 62 genes, which could cover 88.57% (Supplementary Figure S4A) of the first reference, while the HCGSC targets enriched 158 pathways, which could cover 94.11% (Supplementary Figure S4B) of the second reference. Of these 62 overlapped genes, 39 were targeted by *Rhizoma Heterosmilacis*, while 43 were targeted by *Radix Sophorae Flavescentis*. These results confirm the reliability and accuracy of the HCGSC selection model. Two networks were constructed to analyze HCGSC. The first is a C-T network based on 24 components and 631 targets. The second is the target–pathway network based on 532 targets and 158 enriched pathways.

In the first network, the degree of targets ranged from 1 to 16, with the third quartile equal to 3. The change in the proportion of common targets under different conditions showed that the common targets had a higher degree (Figure 4B). The proportion of common targets increased from 27.26% to 100%. Additionally, with the increase in the threshold of the degree, the proportion of unique targets of *Rhizoma Heterosmilacis* decreased from 32.17 to 0%, while the proportion of specific targets of *Radix Sophorae Flavescentis* decreased from 40.57 to 0%. In the second network, we assigned the degree from the first network to the targets; targets with a degree higher than 3 (the third quartile of the targets in the C-T network) and their enriched pathways were selected to construct the network (Figure 4C). Visualization showed that the size of the target was positively related to the degree. We noted that the common targets (in blue diamond) displayed more connections with pathways and larger sizes than the others. However, the unique targets of *Rhizoma Heterosmilacis* (yellow triangle) and *Radix Sophorae Flavescentis* (yellow arrow) not only showed fewer connections with pathways, but were also smaller than the common targets. These two networks indicated that the common targets of *Rhizoma Heterosmilacis* and *Radix Sophorae Flavescentis* in HCGSC have relatively high degrees, which shows that *Rhizoma Heterosmilacis* and *Radix Sophorae Flavescentis* have a synergistic effect on the nodes with higher influence.

### Potential Synergistic Mechanism Analysis of CKI in BC Treatment Based on HCGSC

#### GO Enrichment Analysis for CKI Based on HCGSC

To further interpret the potential synergistic mechanisms of *Rhizoma Heterosmilacis* and *Radix Sophorae Flavescentis*, we performed GO-BP enrichment analysis for the targets of *Radix Sophorae Flavescentis* and *Rhizoma Heterosmilacis* in HCGSC. *Radix Sophorae Flavescentis* targets in HCGSC were enriched in 1517 GO-BP terms, while *Rhizoma Heterosmilacis* targets in HCGSC were enriched in 1394 GO-BP terms. A total of 927 joint GO-BP terms were found between them (Supplementary Figure S4C).

**FIGURE 5:**
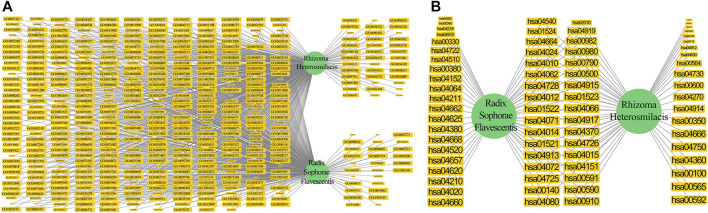
Components were represented in green. The GO-BP terms and pathways were represented in yellow. The size of the pathways was related to the FDR adjust *p* value. The lower FDR adjust *p* value represented the larger size. **(A)** The network of selected GO-BP terms based on the first quartile value of all GO-BP terms FDR adjust *p* value. The left of the network was the commonly enriched GO-BP terms. The upper right of the network was the unique GO-BP terms of *Rhizoma Heterosmilacis*. The lower right of the network was the unique GO-BP terms of *Radix Sophorae Flavescentis*. **(B)** The network of pathways between *Rhizoma Heterosmilacis* and *Radix Sophorae Flavescentis*. The center of the network was the commonly enriched pathways. The right of the network was the unique pathways of *Rhizoma Heterosmilacis*. The left of the network was the unique pathways of *Radix Sophorae Flavescentis*.

We selected the GO-BP terms with an FDR-adjusted *p* value lower than that of the first quartile of all GO-BP terms to build a network (Figure 5A). We found that 86.49% of the selected GO-BP terms belong to the commonly enriched GO-BP terms between the targets of *Radix Sophorae Flavescentis* and *Rhizoma Heterosmilacis* in HCGSC. This suggests that *Rhizoma Heterosmilacis* and *Radix Sophorae Flavescentis* have potential synergistic mechanisms in the treatment of BC through these highly reliable, commonly enriched GO-BP terms based on HCGSC.

It is worth noting that most of the joint GO-BP terms were closely related to BC, such as the steroid metabolic process (GO:0008202, FDR adjusted *p* value = 2.24E-27) and steroid biosynthetic process (GO:0006694, FDR adjusted *p* value = 1.23E-18). Regarding the genes of these GO terms, estrone (*E1*) can protect women from breast cancer, while estradiol (*E2*) and estriol (*E3*) may enhance the risk (Lemon et al., 1966; Cohn et al., 2017). The expression of *ATGL* is correlated with tumor aggressiveness *in vivo* (Wang et al., 2017), which is related to the lipid catabolic process (GO:0016042, FDR adjust *p* value = 1.46E-22), lipid transport (GO:0006869, FDR adjusted *p* value = 4.04E-17), and lipid localization (GO:0010876, FDR adjusted *p* value = 1.16E-16). Including TRPCs, TPRVs, TRPMs, TRPA1, TRPPs, and TRPMLs (Berridge et al., 2003; Rohacs, 2005; Monteith et al., 2007; Venkatachalam and Montell, 2007; Doherty et al., 2015; Vangeel and Voets, 2019), calcium homeostasis plays an important role in the occurrence, development, and metastasis of breast cancer, which is related to the response to metal ions (GO:0010038, FDR adjust *p* value = 9.17E-13).

Additionally, the other GO-BP terms of targets for *Radix Sophorae Flavescentis* and *Rhizoma Heterosmilacis* in HCGSC, respectively, are also related to BC treatment. The ERK/MAPK and JAK2/PI3K signaling cascades in breast cancer cells can be activated by α7-nAChR activation (Chen et al., 2006; Nishioka et al., 2011; Kalantari-Dehaghi et al., 2015), while α9-nAChR overexpression is observed in tumor tissues compared with adjacent normal tissues (Lee et al., 2010). These genes are involved in synaptic transmission, cholinergic (GO:0007271, FDR adjust *p* value = 3.54E-19), and acetylcholine receptor signaling pathways (GO:0095500, FDR adjust *p* value = 1.91E-10) of *Radix Sophorae Flavescentis* in HCGSC. For the remaining GO-BP terms of *Rhizoma Heterosmilacis* in HCGSC, some steroid hormones, such as vitamin D, may have anticancer properties, while others may favor cancer progression, including estrogens and androgens (Restrepo-Angulo et al., 2020), which are related to regulation of steroid metabolic processes (GO:0019218, FDR adjusted *p* value = 2.49E-13).

#### Pathway Analyses Exploring Therapeutic Mechanisms of CKI Based on HCGSC

To further dissect the potential synergistic mechanisms of *Rhizoma Heterosmilacis* and *Radix Sophorae Flavescentis*, we performed the KEGG pathway enrichment analysis for the targets of *Radix Sophorae Flavescentis* and *Rhizoma Heterosmilacis* in HCGSC, respectively. Targets of *Radix Sophorae Flavescentis* in HCGSC were enriched in 55 pathways, while those of *Rhizoma Heterosmilacis* were enriched in 54 pathways. Thirty-four commonly enriched pathways were found between them (Supplementary Figure S4D).

We used pathways to build a network (Figure 5B). Surprisingly, 45.33% of pathways belonged to the commonly enriched pathways between targets of *Radix Sophorae Flavescentis* and *Rhizoma Heterosmilacis* in HCGSC. This suggests that *Rhizoma Heterosmilacis* and *Radix Sophorae Flavescentis* have potential synergistic mechanisms in the treatment of BC through these commonly enriched pathways based on HCGSC.

As shown in Figure 5B, most of the commonly enriched pathways were closely related to BC. Arachidonic acid metabolism (hsa00590, FDR adjusted *p* value = 4.47E-10), as a metabolic process, and the arachidonic acid (AA) pathway play key roles in carcinogenesis (Yarla et al., 2016). Additionally, PLA2s, COXs, LOXs, CYP-dependent monooxygenases, and their metabolites are known to play key roles in carcinogenesis (Tong et al., 2002; Go et al., 2015; Wu et al., 2015; Yarla et al., 2016). The migration and invasion of MDA-MB-231 breast cancer cells may be induced by linoleic acid (Serna-Marquez et al., 2017), which is related to linoleic acid metabolism (hsa00591, FDR adjusted *p* value = 4.47E-10). The PI3K-Akt signaling pathway (hsa04151, FDR adjusted *p* value = 2.78E-07) plays an important role in the tumorigenesis of breast cancer (Yuan and Cantley, 2008), and its activation may promote tumor progression in mice (Mei et al., 2018).

Additionally, other pathways of targets for *Radix Sophorae Flavescentis* and *Rhizoma Heterosmilacis* in HCGSC, respectively, are also related to BC treatment. For other pathways of *Radix Sophorae Flavescentis* in HCGSC, cellular Ca^2+^ signals have been implicated in the induction of apoptosis and regulation of apoptotic pathways (Berridge et al., 1998; Sergeev, 2005), which is related to the calcium signaling pathway (hsa04020, FDR adjusted *p* value = 1.43E-04). Regarding other pathways of *Rhizoma Heterosmilacis* in HCGSC, the higher alpha-linolenic acid content may reduce the risk of breast cancer, which is related to alpha-linolenic acid metabolism (hsa00592, FDR adjusted *p* value = 3.13E-06).

**FIGURE 6:**
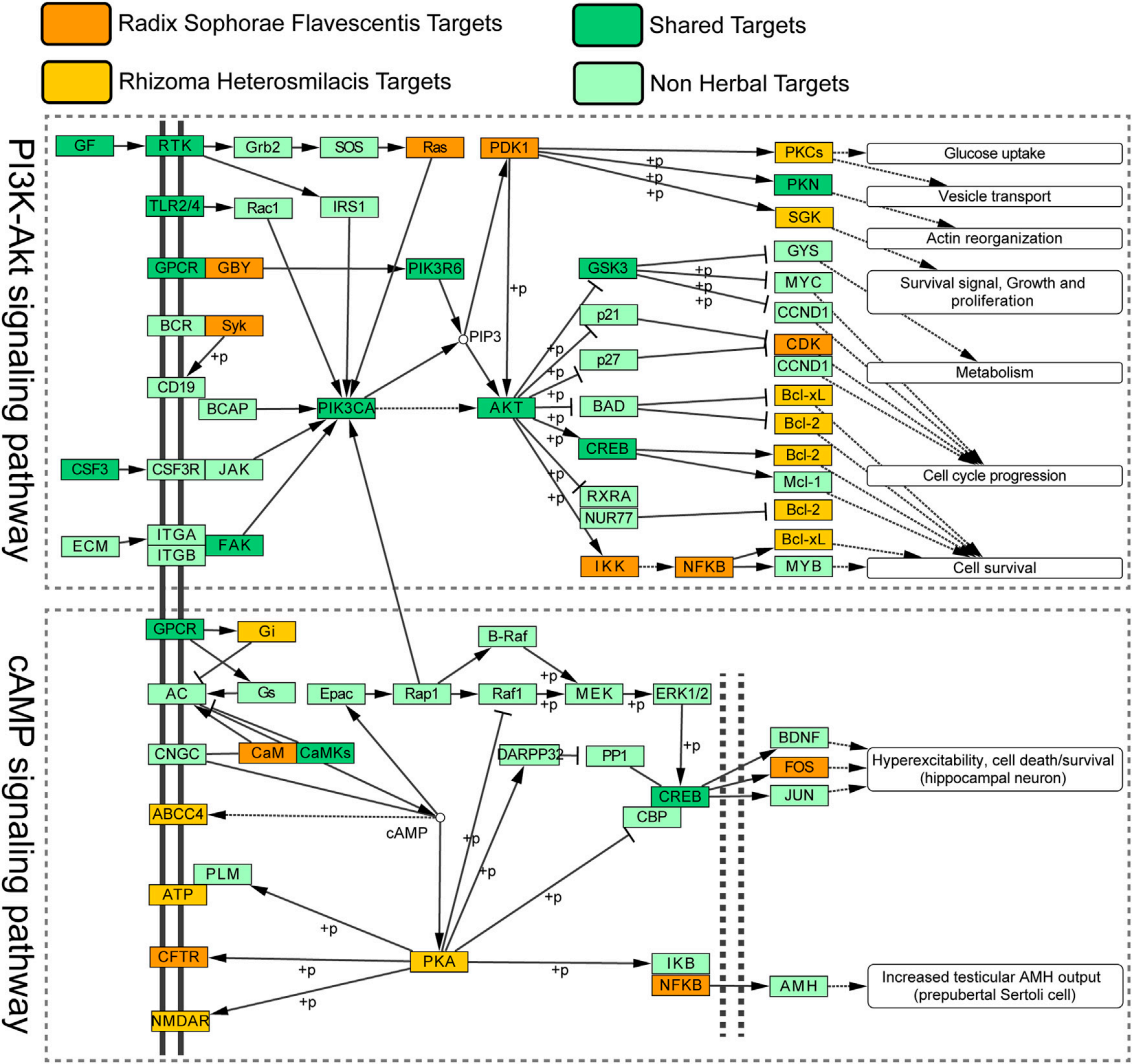
Distribution of target proteins of CKI on the compressed BC pathway. The colors of the blanks represent different types of targets.

To further explain the synergistic mechanisms, we obtained 34 commonly enriched pathways between targets for *Radix Sophorae Flavescentis* SC and *Rhizoma Heterosmilacis* in HCGSC and 25 DEG-enriched pathways. By analyzing these pathways, we found that five pathways overlapped between 34 and 25 pathways, which we considered highly reliable pathways. These include the PI3K-Akt signaling pathway (hsa04151), cAMP signaling pathway (hsa04024), neuroactive ligand–receptor interaction (hsa04080), dopaminergic synapse (hsa04728), and ABC transporters (hsa02010). The PI3K-Akt and cAMP signaling pathways are reportedly related to BC, with a greater number of published studies (Mei et al., 2018; Dong et al., 2015). Next, we constructed a comprehensive pathway (Figure 6), including the PI3K-Akt and cAMP signaling pathways, using CyKEGGParser to explore the synergetic mechanisms of *Rhizoma Heterosmilacis* and *Radix Sophorae Flavescentis* in the treatment of BC using CKI (Nersisyan et al., 2014).

In the PI3K-Akt signaling pathway, *Radix Sophorae Flavescentis* acts on targets of the upstream pathway, such as Ras, GBY, and Syk, while *Rhizoma Heterosmilacis* acts on downstream targets, such as PKCs, SGK, Bcl-2, and Bcl-xL. These results indicate that *Rhizoma Heterosmilacis* and *Radix Sophorae Flavescentis* have synergistic and complementary effects on cell survival, cell cycle progression, metabolism, survival signal, growth and proliferation, actin reorganization, vesicle transport, and glucose uptake. Additionally, in the cAMP signaling pathway, *Rhizoma Heterosmilacis* acts as a target of the upstream pathway, including Gi and PKA, while *Radix Sophorae Flavescentis* acts as a target of the downstream pathway, including FOS, NFKB, and CFTR, which are associated with pathways of hyperexcitability, cell death or survival (hippocampal neuron), and increased testicular AMH output (prepubertal Sertoli cells). As the pathogenic factors of BC are related to metabolism, cell survival, proliferation, cell death, and cell cycle (Jia et al., 2018), the above results suggest that *Rhizoma Heterosmilacis* and *Radix Sophorae Flavescentis* can exert a synergistic effect on BC at multiple pathways.

### Molecular Docking Validation of HCGSC in the Treatment of BC

**FIGURE 7:**
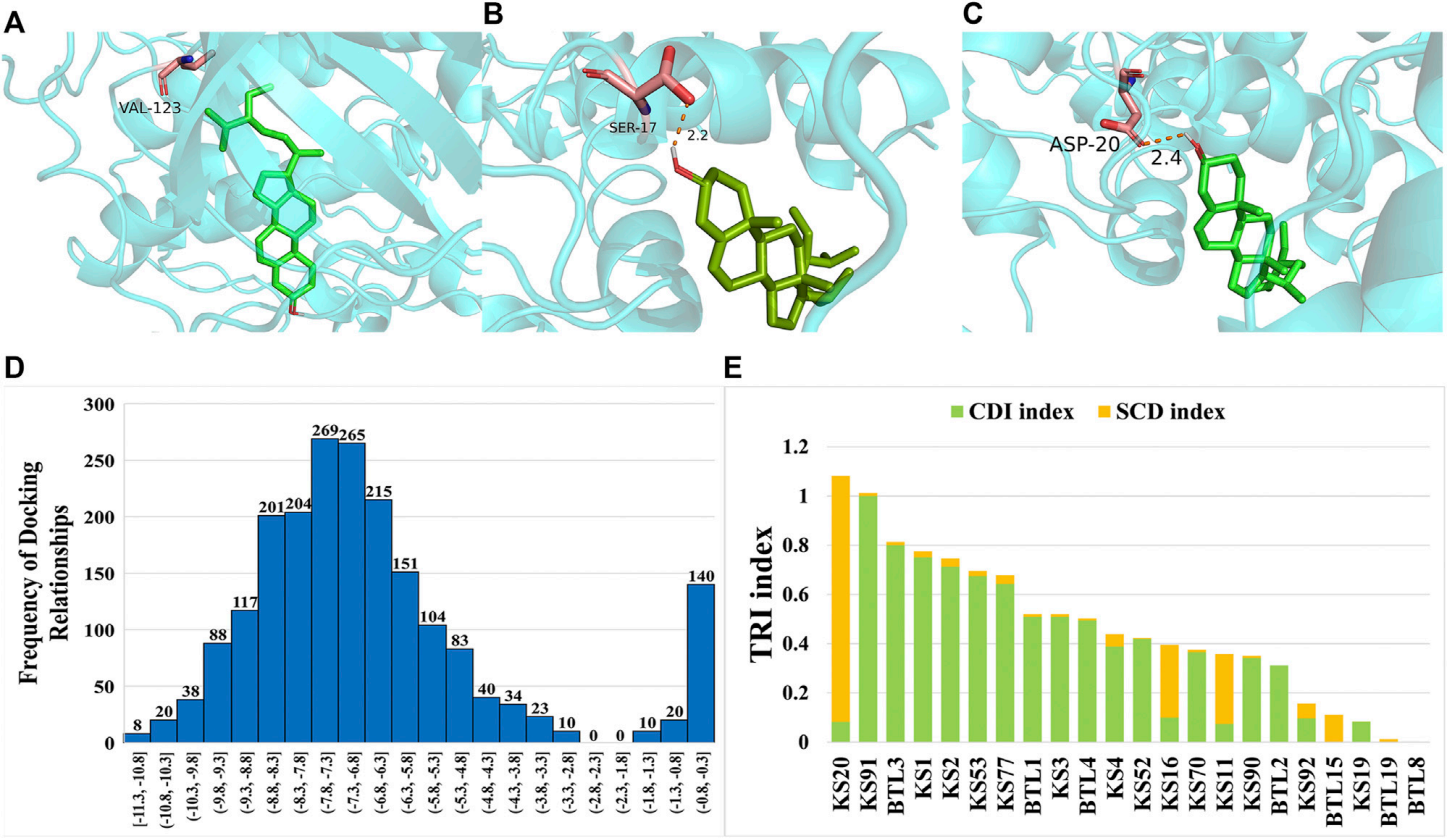
**(A)** Docking result visualization of BTL3-3ovv (coded by PKA). **(B)** Docking result visualization of BTL4-2w73 (coded by CaM). **(C)** Docking result visualization of BTL3-2w73 (coded by Cam). **(D)** The frequency histogram of the affinity value. **(E)** The bar plot to display the composition of CDI and SCD in each TRI.

To evaluate the function of HCGSC in BC treatment, molecular docking was employed to simulate the interaction between the ligands and the protein. Twenty-four components from the HCGSC of CKI and 85 proteins coded by 30 genes were used for molecular docking, and 18,254 docking relationships were obtained from the docking results (Supplementary Table S7). The result with the lowest affinity value in each component-protein was selected as the best docking relationship (2040 docking relationships), of which the affinity value ranged from −11.3 kcal/mol to −0.5 kcal/mol. Among the best docking relationships, three exhibited affinity values lower than −11.0 kcal/mol, BTL3 and BTL4 had the lowest affinity values of −11.3 kcal/mol, binding with protein 3ovv coded by PKA and protein 2w73 coded by CaM, respectively. BTL3 could bind 2w73 coded by CaM with the affinity values of −11.2 kcal/mol thereafter (Figures 7A–C). Based on the literature reports, improved binding between component–protein interactions should have a lower value of affinity (Elhenawy et al., 2019), and the histogram revealed that most of the results were concentrated in the lower affinity value position (Figure 7D).

To reduce the influence of background noise of docking relationships with high affinity values, we selected the docking relationships that were lower than the average affinity value (−6.743) from all the best docking relationships as a highly reliable docking relationship (HRDR). Twenty-two components and 82 proteins coded by 29 genes were identified in HRDR with 1,255 docking relationships. Among the 22 components, 15 components were from *Radix Sophorae Flavescentis*, including KS1, KS2, KS3, KS4, KS11, KS16, KS19, KS20, KS52, KS53, KS70, KS77, KS90, KS91, and KS92, and seven components were from *Rhizoma Heterosmilacis*, including BTL1, BTL2, BTL3, BTL4, BTL8, BTL15, and BTL19. The 15 components of *Radix Sophorae Flavescentis* could bind 82 proteins coded by 29 genes, and the seven components of *Rhizoma Heterosmilacis* could bind 81 proteins coded by 29 genes. These results suggest that *Rhizoma Heterosmilacis* and *Radix Sophorae Flavescentis* exert a synergistic effect on BC at multiple pathways.

To evaluate the therapeutic potential of components in HRDR, we designed a mathematical model to calculate the TRI of each component in the HRDR from the HCGSC. To further decode the mechanism of CKI in treating BC comprehensively, we integrated the CDI and SCD to calculate the TRI, which not only considered synergistic effects, but also considered the docking relationship. Detailed information on the TRI is listed in Supplementary Table S8. As shown in Figure 7E, KS20 (TRI = 1.08), KS91 (TRI = 1.01), BTL3 (TRI = 0.81), and KS1 (TRI = 0.78) were the top four components with TRI scores higher than 0.75. Among these four components, matrine (KS20), with the highest TRI, was confirmed by a number of published studies related to BC therapy (Li et al., 2015). The following three components with higher TRIs were selected for *in vitro* experiments to validate the reliability of our proposed strategy.

### Luteolin and Trifolirhizin Inhibited Proliferation and Metastasis of MCF-7 Cells *In Vitro*

**FIGURE 8:**
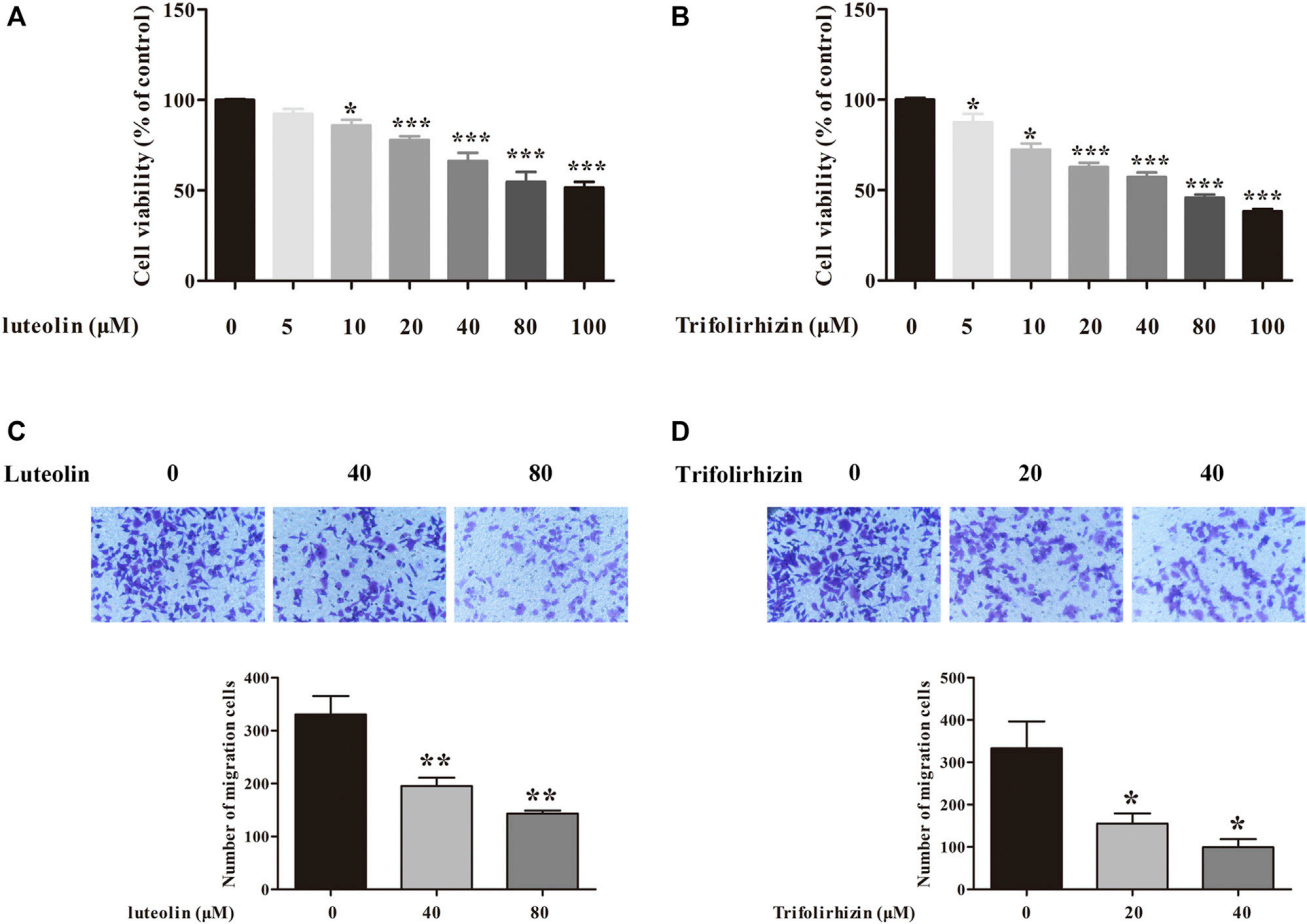
Inhibitory effects of luteolin **(A)** and trifolirhizin **(B)** on the proliferation of MCF-7 cells. Inhibitory effects of luteolin **(C)** and trifolirhizin **(D)** on migration in MCF-7 cells. Data are represented as mean ± SEM (n = 3). ^*^ *p* < 0.05, ^**^ *p* < 0.01, ^***^ *p* < 0.001 as compared with that of the control group.

Based on the TRI results, trifolirhizin (KS91), beta-sitosterol (BTL3), and luteolin (KS1) were selected for *in vitro* experiments. The *in vitro* experiments proved that two of the three core components of HCGSC showed significant inhibitory effects on breast cancer cell proliferation and migration. The effects of luteolin and trifolirhizin on the viability of MCF-7 cells were determined using the MTT assay. As shown in Figures 8A,B, the proliferation of MCF-7 cells was suppressed by luteolin and trifolirhizin in a dose-dependent manner. Subsequently, the effects of luteolin and trifolirhizin on the migration of MCF-7 cells were investigated. As presented in Figures 7C,D, luteolin (40 and 80 Μm) and trifolirhizin (20 and 40 Μm) dramatically inhibited the migration of MCF-7 cells. These results indicated that luteolin and trifolirhizin markedly inhibited the proliferation and migration of MCF-7 cells.

## Discussion

Breast cancer is one of the most common malignant tumors among women worldwide, with the second highest death rate in female cancer patients (Siegel et al., 2020). At present, research regarding cancer treatment using TCM is still in the exploratory stage, and CKI has a wide range of applications in the treatment of tumors. However, there are few studies on CKI in the treatment of BC through systematic pharmacology. Therefore, we designed a systematic pharmacology strategy combining differentially expressed gene analysis, pharmacokinetics synthesis screening, target identification, synergy contribution degree, network analysis, therapeutic response index calculation, and experimental validation and provided a reference for this new method.

In this study, we examined the synergistic effect of CKI on BC at three levels. At the first level, the target coincidence of *Rhizoma Heterosmilacis* and *Radix Sophorae Flavescentis* in CKI indicates that *Rhizoma Heterosmilacis* and *Radix Sophorae Flavescentis* may act synergistically to exert a therapeutic effect in treating BC. First, we constructed a C-T network and found that most targets with a higher degree were mapped on the joint targets between *Rhizoma Heterosmilacis* and *Radix Sophorae Flavescentis*. These results suggest that *Rhizoma Heterosmilacis* and *Radix Sophorae Flavescentis* may act synergistically to treat BC *via* common targets and provide therapeutic targets for the cooperative treatment of BC. However, in the complex C-T network, we know neither the key synergistic components in treating BC nor their synergistic mechanisms.

To solve this issue, at the second level, we designed an SCD model with the advantages of reflecting the topology character of the C-T network and the component dose, which implemented a semi-quantitative system pharmacology model. Through the analysis of the SCD model, 24 components compose HCGSC, which can contribute to the effects of CKI on BC with a sum of 95.03% and map 80.89% (631/780) of all targets of CKI. Two references were employed to confirm the reliability and accuracy of the HCGSC selection model, including genes and pathways. The targets of HCGSC contained 62 genes, which could cover 88.57% of the first reference, while the HCGSC targets enriched 81 pathways, which could cover 70.59% of the second reference. Third, we performed GO-BP and KEGG enrichment analysis for *Radix Sophorae Flavescentis* and *Rhizoma Heterosmilacis* targets of HCGSC. We found that most of the overlapped GO-BP terms and pathways between them affect BC treatment, and most of them have a lower FDR adjusted *p* value.

At the third level, to further understand the synergistic mechanisms of CKI and infer the function of synergistic components and verify their effectiveness, three aspects were employed. First, a comprehensive pathway was constructed to explore the synergetic mechanism of *Rhizoma Heterosmilacis* and *Radix Sophorae Flavescentis* in the treatment of BC using CKI. In the PI3K-Akt and cAMP signaling pathways, *Rhizoma Heterosmilacis* and *Radix Sophorae Flavescentis* can activate upstream and downstream targets to play their role in the treatment of BC. These results suggest that CKI can produce a combined effect on BC. Second, we designed a TRI model, which provides the results of SCD and docking, to comprehensively evaluate the synergy contribution rate of the components in HCGSC and the binding ability of the components and proteins. TRI calculation results showed that four components displayed a TRI score higher than 0.75, including matrine (KS20, TRI = 1.08), trifolirhizin (KS91, TRI = 1.01), beta-sitosterol (BTL3, TRI = 0.81), and luteolin (KS1, TRI = 0.78), which may be the core components in the treatment of BC with synergistic mechanisms. Third, MTT and Transwell assays were employed to determine the function of luteolin and trifolirhizin. The results showed that they markedly inhibited the proliferation and migration of MCF-7 cells.

In this study, we proposed two novel mathematical models for the speculation of synergistic components and mechanisms. The method provided a methodological reference for decoding the synergistic mechanism of TCM in treating complex diseases. However, several limitations must be mentioned. 1) The precise synergistic mechanisms based on the calculation predictions warrant further validation. 2) Because the interaction between herbs is extremely complex, it may be incomplete to explore the synergistic mechanisms based on our model. Multiple synergistic effects combining with other techniques should be further considered. 3) The TRI model is proposed based on network topology methodology, which is more suitable for the case that herbs have a complex network. This algorithm used undirected network, which ignores the activation or inhibition effects of the disease targets.

## Data Availability

Publicly available datasets were analyzed in this study. This data can be found here: https://portal.gdc.cancer.gov/ https://tcmsp-e.com/.

## Supplementary Material

Supplementary Figure S1 The process of ADME screening.

Supplementary Figure S2 The heatmap of DEGs of breast cancer. The red color represents the high expression of the gene and the green color represents the low expression of the gene.

Supplementary Figure S3 The C-T network of *Rhizoma Heterosmilacis* and *Radix Sophorae Flavescentis*. The targets were in yellow and the components were in the green. The common targets were placed at the center of the network. The targets at the left were the unique targets of *Radix Sophorae Flavescentis*, while those at the right were the unique targets of *Rhizoma Heterosmilacis*. The size of the targets and components were related to the degree. The higher degree represented the larger size.

Supplementary Figure S4
**(A)** The Venn diagram of the joint targets between BC and CKI and the targets of HCGSC. **(B)** The Venn diagram of the joint KEGG pathways between BC and CKI and the KEGG pathways of HCGSC. **(C)** The Venn diagram of joint GO-BP terms between *Radix Sophorae Flavescentis* in HCGSC and *Rhizoma Heterosmilacis* in HCGSC. **(D)** The Venn diagram of the joint pathway between *Radix Sophorae Flavescentis* in HCGSC and *Rhizoma Heterosmilacis* in HCGSC.

## Abbreviations

ADME, absorption, distribution, metabolism, and excretion; BC, breast cancer; CKI, compound kushen injection; C-T network, component–target network; DEGs, differentially expressed gene analysis; HCGSC, high contribution group of synergistic components; HPLC, high performance liquid chromatography; HRDR, highly reliable docking relationship; SCD, synergy contribution degree; TCGA, The Cancer Genome Atlas; TCM, traditional Chinese medicine; TRI, therapeutic response index.

## References

[B1] Anders S. Huber W. (2010). Differential expression analysis for sequence count data. Genome Biol. 11 (10), R106. 10.1186/gb-2010-11-10-r106 20979621 PMC3218662

[B2] Astashchanka A. Shroka T. M. Jacobsen B. M. (2019). Mucin 2 (MUC2) modulates the aggressiveness of breast cancer. Breast Cancer Res. Treat. 173 (2), 289–299. 10.1007/s10549-018-4989-2 30317423 PMC6813790

[B3] Awad A. B. Downie A. C. Fink C. S. (2000). Inhibition of growth and stimulation of apoptosis by beta-sitosterol treatment of MDA-MB-231 human breast cancer cells in culture. Int. J. Mol. Med. 5 (5), 541–545. 10.3892/ijmm.5.5.541 10762659

[B4] Awad A. B. Roy R. Fink C. S. (2003). Beta-sitosterol, a plant sterol, induces apoptosis and activates key caspases in MDA-MB-231 human breast cancer cells. Oncol. Rep. 10 (2), 497–500. 10.3892/or.10.2.497 12579296

[B5] Balaji K. Subramanian B. Yadav P. Anu Radha C. Ramasubramanian V. (2016). Radiation therapy for breast cancer: Literature review. Med. Dosim. 41 (3), 253–257. 10.1016/j.meddos.2016.06.005 27545009

[B6] Berridge M. J. Bootman M. D. Lipp P. (1998). Calcium--a life and death signal. Nature 395 (6703), 645–648. 10.1038/27094 9790183

[B7] Berridge M. J. Bootman M. D. Roderick H. L. (2003). Calcium signalling: dynamics, homeostasis and remodelling. Nat. Rev. Mol. Cel Biol 4 (7), 517–529. 10.1038/nrm1155 12838335

[B8] Binggang Z. Jing L. Yuzhuo F. Gang S. (2002). Experimental study on apoptosis of human breast cancer cell line MCF-7 induced by oxymatrine. Chin. Pharmacol. Bull. (06), 689–691.

[B9] Cao D. Zhu G. Y. Lu Y. Yang A. Chen D. Huang H. J. (2020). Luteolin suppresses epithelial-mesenchymal transition and migration of triple-negative breast cancer cells by inhibiting YAP/TAZ activity. Biomed. Pharmacother. 129, 110462. 10.1016/j.biopha.2020.110462 32768952

[B10] Chen Z. B. Liu C. Chen F. Q. Li S. Y. Liang Q. Liu L. Y. (2006). Effects of tobacco-specific carcinogen 4-(methylnitrosamino)-1-(3-pyridyl)-1-butanone (NNK) on the activation of ERK1/2 MAP kinases and the proliferation of human mammary epithelial cells. Environ. Toxicol. Pharmacol. 22 (3), 283–291. 10.1016/j.etap.2006.04.001 21783722

[B11] Cochran J. M. Busch D. R. Leproux A. Zhang Z. O'Sullivan T. D. Cerussi A. E. (2019). Tissue oxygen saturation predicts response to breast cancer neoadjuvant chemotherapy within 10 days of treatment. J. Biomed. Opt. 24 (02), 1–11. 10.1117/1.JBO.24.2.021202 PMC6194199 30338678

[B12] Cohn B. A. Cirillo P. M. Hopper B. R. Siiteri P. K. (2017). Third Trimester Estrogens and Maternal Breast Cancer: Prospective Evidence. J. Clin. Endocrinol. Metab. 102 (10), 3739–3748. 10.1210/jc.2016-3476 28973345 PMC5630249

[B13] Colaprico A. Silva T. C. Olsen C. Garofano L. Cava C. Garolini D. (2016). TCGAbiolinks: an R/Bioconductor package for integrative analysis of TCGA data. Nucleic Acids Res. 44 (8), e71. 10.1093/nar/gkv1507 26704973 PMC4856967

[B14] Condorelli R. Vaz-Luis I. (2018). Managing side effects in adjuvant endocrine therapy for breast cancer. Expert Rev. Anticancer Ther. 18 (11), 1101–1112. 10.1080/14737140.2018.1520096 30188738

[B15] Crean-Tate K. K. Reizes O. (2018). Leptin Regulation of Cancer Stem Cells in Breast and Gynecologic Cancer. Endocrinology 159 (8), 3069–3080. 10.1210/en.2018-00379 29955847 PMC6669812

[B16] Cui J. Qu Z. Harata-Lee Y. Nwe Aung T. Shen H. Wang W. (2019). Cell cycle, energy metabolism and DNA repair pathways in cancer cells are suppressed by Compound Kushen Injection. BMC Cancer 19 (1), 103. 10.1186/s12885-018-5230-8 30678652 PMC6345000

[B17] Cui J. Qu Z. Harata-Lee Y. Shen H. Aung T. N. Wang W. (2020). The effect of compound kushen injection on cancer cells: Integrated identification of candidate molecular mechanisms. PLoS One 15 (7), e0236395. 10.1371/journal.pone.0236395 32730293 PMC7392229

[B18] Cuiping Y. (2010). Review on Pharmacological Researches of Sophoridine. Chin. J. Exp. Traditional Med. Formulae.

[B19] Doherty A. H. Ghalambor C. K. Donahue S. W. (2015). Evolutionary Physiology of Bone: Bone Metabolism in Changing Environments. Physiology (Bethesda) 30 (1), 17–29. 10.1152/physiol.00022.2014 25559152

[B20] Dong H. Claffey K. P. Brocke S. Epstein P. M. (2015). Inhibition of breast cancer cell migration by activation of cAMP signaling. Breast Cancer Res. Treat. 152 (1), 17–28. 10.1007/s10549-015-3445-9 26022351

[B21] Elhenawy A. A. Al-Harbi L. M. El-Gazzar M. A. Khowdiary M. M. Moustfa A. (2019). Synthesis, molecular properties and comparative docking and QSAR of new 2-(7-hydroxy-2-oxo-2H-chromen-4-yl)acetic acid derivatives as possible anticancer agents. Spectrochim Acta A. Mol. Biomol. Spectrosc. 218, 248–262. 10.1016/j.saa.2019.02.074 31003050

[B22] Enien M. A. Ibrahim N. Makar W. Darwish D. Gaber M. (2018). Health-related quality of life: Impact of surgery and treatment modality in breast cancer. J. Cancer Res. Ther. 14 (5), 957–963. 10.4103/0973-1482.183214 30197331

[B23] Gao L. Wang K. X. Zhou Y. Z. Fang J. S. Qin X. M. Du G. H. (2018). Uncovering the anticancer mechanism of Compound Kushen Injection against HCC by integrating quantitative analysis, network analysis and experimental validation. Sci. Rep. 8 (1), 624. 10.1038/s41598-017-18325-7 29330507 PMC5766629

[B24] Gfeller D. Grosdidier A. Wirth M. Daina A. Michielin O. Zoete V. (2014). SwissTargetPrediction: a web server for target prediction of bioactive small molecules. Nucleic Acids Res. 42, W32–W38. (Web Server issue). 10.1093/nar/gku293 24792161 PMC4086140

[B25] Go R. E. Hwang K. A. Choi K. C. (2015). Cytochrome P450 1 family and cancers. J. Steroid Biochem. Mol. Biol. 147, 24–30. 10.1016/j.jsbmb.2014.11.003 25448748

[B26] Guo Y. Fan Y. Pei X. (2020). Fangjihuangqi Decoction inhibits MDA-MB-231 cell invasion *in vitro* and decreases tumor growth and metastasis in triple-negative breast cancer xenografts tumor zebrafish model. Cancer Med. 9 (7), 2564–2578. 10.1002/cam4.2894 32037729 PMC7131862

[B27] Guo Y. Huang Y. Shen H. Sang X. Ma X. Zhao Y. et alXiao X. (2015). Efficacy of Compound Kushen Injection in Relieving Cancer-Related Pain: A Systematic Review and Meta-Analysis. United States: Hindawi Publishing Corporation, 840742–840748. 10.1155/2015/840742 PMC4609400 26504481

[B28] Han F. M. Zhu M. M. Chen H. X. Chen Y. (2009). Identification of Sophoridine and its Metabolites in Rat Urine by Liquid Chromatography-Tandem Mass Spectrometry. Anal. Lett. 43 (1), 45–54. 10.1080/00032710903276471

[B29] Hong Q. Yu S. Mei Y. Lv Y. Chen D. Wang Y. (2014). Smilacis Glabrae Rhizoma Reduces Oxidative Stress Caused by Hyperuricemia via Upregulation of Catalase. Cell. Physiol. Biochem. 34 (5), 1675–1685. 10.1159/000366369 25401709

[B30] Hu S. T. Shen Y. F. Gong J. M. Yang Y. J. (2016). Effect of sophoridine on Ca²⁺ induced Ca²⁺ release during heart failure. Physiol. Res. 65, 43–52. 10.33549/physiolres.933052 26596316

[B31] Huang X. Li B. Shen L. (20142014). Studies on the Anti-inflammatory Effect and its Mechanisms of Sophoridine. J. Anal. Methods Chem. 2014, 502626–6. 10.1155/2014/502626 PMC4000653 24812589

[B32] Hy L. Qy H. (2014). Mechanisms of yanshu injection for overcoming multidrug resistance in breast carcinoma MCF-7 cells: an experimental research. Chin. J. integrated traditional West. Med. 34 (3), 324–328. 24758085

[B33] Ij H. Tj R. Rt B. (1989). Characterization of the human colon carcinoma cell line (Caco-2) as a model system for intestinal epithelial permeability. Gastroenterology 96 (3), 736–749. 2914637

[B34] Imran M. Rauf A. Abu-Izneid T. Nadeem M. Shariati M. A. Khan I. A. (2019). Luteolin, a flavonoid, as an anticancer agent: A review. Biomed. Pharmacother. 112, 108612. 10.1016/j.biopha.2019.108612 30798142

[B35] Jacobsen A. C. Nielsen S. Brandl M. Bauer-Brandl A. (2020). Drug Permeability Profiling Using the Novel Permeapad® 96-Well Plate. Pharm. Res. 37 (6), 93. 10.1007/s11095-020-02807-x 32394114

[B36] Jia L. Huang S. Yin X. Zan Y. Guo Y. Han L. (2018). Quercetin suppresses the mobility of breast cancer by suppressing glycolysis through Akt-mTOR pathway mediated autophagy induction. Life Sci. 208, 123–130. 10.1016/j.lfs.2018.07.027 30025823

[B37] Jiang S. Fan J. Wang Q. Ju D. Feng M. Li J. (2016). Diosgenin induces ROS-dependent autophagy and cytotoxicity via mTOR signaling pathway in chronic myeloid leukemia cells. Phytomedicine 23 (3), 243–252. 10.1016/j.phymed.2016.01.010 26969378

[B38] Jiaqin C. Xiaoxia W. Xuhui H. Jie Z. Hong S. (2018). Oxymatrine inhibits invasion and metastasis of triple negative breast cancer cells by regulating epithelial-mesenchymal transformation. Chin. J. Clin. Pharmacol. Ther. 23 (01), 13–17.

[B39] Jin Y. Yang Q. Liang L. Ding L. Liang Y. Zhang D. (2018). Compound kushen injection suppresses human acute myeloid leukaemia by regulating the Prdxs/ROS/Trx1 signalling pathway. J. Exp. Clin. Cancer Res. 37 (1), 277. 10.1186/s13046-018-0948-3 30454068 PMC6245615

[B40] Jingyuan W. Bowen X. Jie L. Weilu C. Taicheng L. Luchang C. (2021). [Exploring the Molecular Biological Mechanism of Shugan Jianpi Decoction in the Treatment of Depression-related Breast Cancer Based Treatment of Depression-related Breast Cancer Based]. Hainan: Journal of Hainan Medical University, 1–16. 10.13210/j.cnki.jhmu.20210315.001

[B41] Juan T. Weihao W. Huimin G. Zhimin W. (2007). Determination of matrine, sophoridine and oxymatrine in compound Sophora flavescens injection by HPLC. China J. Chin. Materia Med. (03), 222–224. 17432143

[B42] Kalantari-Dehaghi M. Parnell E. A. Armand T. Bernard H. U. Grando S. A. (2015). The nicotinic acetylcholine receptor-mediated reciprocal effects of the tobacco nitrosamine NNK and SLURP-1 on human mammary epithelial cells. Int. Immunopharmacol. 29 (1), 99–104. 10.1016/j.intimp.2015.04.041 25986726

[B43] Kassambara A. (2020). ggpubr: 'ggplot2' Based Publication Ready Plots. R package version.

[B44] Keiser M. J. Roth B. L. Armbruster B. N. Ernsberger P. Irwin J. J. Shoichet B. K. (2007). Relating protein pharmacology by ligand chemistry. Nat. Biotechnol. 25 (2), 197–206. 10.1038/nbt1284 17287757

[B45] Kirn V. Strake L. Thangarajah F. Richters L. Eischeid H. Koitzsch U. (2018). ESR1-promoter-methylation status in primary breast cancer and its corresponding metastases. Clin. Exp. Metastasis 35 (7), 707–712. 10.1007/s10585-018-9935-5 30173322

[B46] Kwon M. Jang M. Kim G. H. Oh T. Ryoo I. J. Ryu H. W. (2020). Kushenol E inhibits autophagy and impairs lysosomal positioning via VCP/p97 inhibition. Biochem. Pharmacol. 175, 113861. 10.1016/j.bcp.2020.113861 32081789

[B47] Kwon M. Ko S. K. Jang M. Kim G. H. Ryoo I. J. Son S. (2019). Inhibitory effects of flavonoids isolated from Sophora flavescens on indoleamine 2,3-dioxygenase 1 activity. J. Enzyme Inhib. Med. Chem. 34 (1), 1481–1488. 10.1080/14756366.2019.1640218 31423846 PMC6713164

[B48] Lee C. H. Huang C. S. Chen C. S. Tu S. H. Wang Y. J. Chang Y. J. (2010). Overexpression and activation of the alpha9-nicotinic receptor during tumorigenesis in human breast epithelial cells. J. Natl. Cancer Inst. 102 (17), 1322–1335. 10.1093/jnci/djq300 20733118

[B49] Lemon H. M. Wotiz H. H. Parsons L. Mozden P. J. (1966). Reduced estriol excretion in patients with breast cancer prior to endocrine therapy. JAMA 196 (13), 1128–1136. 10.1001/jama.1966.03100260066020 5952514

[B50] Li H. Li X. Bai M. Suo Y. Zhang G. Cao X. (2015). Matrine inhibited proliferation and increased apoptosis in human breast cancer MCF-7 cells via upregulation of Bax and downregulation of Bcl-2. Int. J. Clin. Exp. Pathol. 8 (11), 14793–14799. 26823806 PMC4713592

[B51] Li J. Hu L. Zhou T. Gong X. Jiang R. Li H. (2019). Taxifolin inhibits breast cancer cells proliferation, migration and invasion by promoting mesenchymal to epithelial transition via β-catenin signaling. Life Sci. 232, 116617. 10.1016/j.lfs.2019.116617 31260685

[B52] Liang G. Nie Y. Chang Y. Zeng S. Liang C. Zheng X. (2019). Protective effects of Rhizoma smilacis glabrae extracts on potassium oxonate- and monosodium urate-induced hyperuricemia and gout in mice. Phytomedicine 59, 152772. 10.1016/j.phymed.2018.11.032 31005813

[B53] Liu C. Kang Y. Zhou X. Yang Z. Gu J. Han C. (2017). Rhizoma smilacis glabrae protects rats with gentamicin-induced kidney injury from oxidative stress-induced apoptosis by inhibiting caspase-3 activation. J. Ethnopharmacol. 198, 122–130. 10.1016/j.jep.2016.12.034 28034658

[B54] Liu R. Peng J. Wang H. Li L. Wen X. Tan Y. (2018). Oxysophocarpine Retards the Growth and Metastasis of Oral Squamous Cell Carcinoma by Targeting the Nrf2/HO-1 Axis. Cel. Physiol. Biochem. 49 (5), 1717–1733. 10.1159/000493615 30231242

[B55] Liu S. Hu X. Fan X. Jin R. Yang W. Geng Y. (2020). A Bioinformatics Research on Novel Mechanism of Compound Kushen Injection for Treating Breast Cancer by Network Pharmacology and Molecular Docking Verification. Evid.-based Compl. Alt. 2020, 1–14. 10.1155/2020/2758640 PMC7439201 32849897

[B56] Liu X. Vogt I. Haque T. Campillos M. (2013). HitPick: a web server for hit identification and target prediction of chemical screenings. Bioinformatics 29 (15), 1910–1912. 10.1093/bioinformatics/btt303 23716196

[B57] Liu X. Zhao W. Wang W. Lin S. Yang L. (2017). Puerarin suppresses LPS-induced breast cancer cell migration, invasion and adhesion by blockage NF-Κb and Erk pathway. Biomed. Pharmacother. 92, 429–436. 10.1016/j.biopha.2017.05.102 28558356

[B58] Mei Y. Yang J. P. Lang Y. H. Peng L. X. Yang M. M. Liu Q. (2018). Global expression profiling and pathway analysis of mouse mammary tumor reveals strain and stage specific dysregulated pathways in breast cancer progression. Cell Cycle 17 (8), 963–973. 10.1080/15384101.2018.1442629 29712537 PMC6103659

[B59] Meng G. Tang X. Yang Z. Benesch M. G. K. Marshall A. Murray D. (2017). Implications for breast cancer treatment from increased autotaxin production in adipose tissue after radiotherapy. FASEB J. 31 (9), 4064–4077. 10.1096/fj.201700159R 28539367

[B60] Merghoub N. Benbacer L. El Btaouri H. Ait Benhassou H. Terryn C. Attaleb M. (2011). *In vitro* antiproliferative effect and induction of apoptosis by Retama monosperma L. extract in human cervical cancer cells. Cel Mol Biol (Noisy-le-grand) 57 Suppl (Suppl. l), OL1581–91. 10.5772/30025 22000488

[B61] Miller K. D. Fidler-Benaoudia M. Keegan T. H. Hipp H. S. Jemal A. Siegel R. L. (2020). Cancer statistics for adolescents and young adults, 2020. CA Cancer J. Clin. 70 (1), 443–459. 10.3322/caac.2159010.3322/caac.21637 32940362

[B62] Monteith G. R. Mcandrew D. Faddy H. M. Roberts-Thomson S. J. (2007). Calcium and cancer: targeting Ca2+ transport. Nat. Rev. Cancer 7 (7), 519–530. 10.1038/nrc2171 17585332

[B63] Nersisyan L. Samsonyan R. Arakelyan A. (2014). CyKEGGParser: tailoring KEGG pathways to fit into systems biology analysis workflows. F1000Res 3, 145. 10.12688/f1000research.4410.210.12688/f1000research.4410.1 25383185 PMC4215754

[B64] Nishioka T. Kim H. S. Luo L. Y. Huang Y. Guo J. Chen C. Y. (2011). Sensitization of epithelial growth factor receptors by nicotine exposure to promote breast cancer cell growth. Breast Cancer Res. 13 (6), R113. 10.1186/bcr3055 22085699 PMC3326555

[B65] Nourmohammadi S. Aung T. N. Cui J. Pei J. V. De Ieso M. L. Harata-Lee Y. (2019). Effect of Compound Kushen Injection, a Natural Compound Mixture, and its Identified Chemical Components on Migration and Invasion of Colon, Brain, and Breast Cancer Cell Lines. Front. Oncol. 9, 314. 10.3389/fonc.2019.00314 31106149 PMC6498862

[B66] O'Boyle N. M. Banck M. James C. A. Morley C. Vandermeersch T. Hutchison G. R. (2011). Open Babel: An open chemical toolbox. J. Cheminform 3 (1), 33–14. 10.1186/1758-2946-3-33 21982300 PMC3198950

[B67] Otte M. Zafrakas M. Riethdorf L. Pichlmeier U. Löning T. Jänicke F. (2001). MAGE-A gene expression pattern in primary breast cancer. Cancer Res. 61 (18), 6682–6687. 11559535

[B68] Qi L. Zhang J. Zhang Z. (2013). Determination of four alkaloids in Compound Kushen Injection by high performance liquid chromatography with ionic liquid as mobile phase additive. Se Pu 31 (03), 249–253. 10.3724/sp.j.1123.2012.10039 23785997

[B69] Qu Z. Cui J. Harata-Lee Y. Aung T. N. Feng Q. Raison J. M. (2016). Identification of candidate anti-cancer molecular mechanisms of Compound Kushen Injection using functional genomics. Oncotarget 7 (40), 66003–66019. 10.18632/oncotarget.11788 27602759 PMC5323210

[B70] Recht A. (2017). Radiation-Induced Heart Disease after Breast Cancer Treatment: How Big a Problem, and How Much Can-and Should-We Try to Reduce it. J. Clin. Oncol. 35 (11), 1146–1148. 10.1200/JCO.2016.71.4113 28095154

[B71] Ren M. X. Deng X. H. Ai F. Yuan G. Y. Song H. Y. (2015). Effect of quercetin on the proliferation of the human ovarian cancer cell line SKOV-3 *in vitro* . Exp. Ther. Med. 10 (2), 579–583. 10.3892/etm.2015.2536 26622357 PMC4508991

[B72] Restrepo-Angulo I. Bañuelos C. Camacho J. (2020). Ion Channel Regulation by Sex Steroid Hormones and Vitamin D in Cancer: A Potential Opportunity for Cancer Diagnosis and Therapy. Front. Pharmacol. 11, 152. 10.3389/fphar.2020.00152 32210800 PMC7076584

[B73] Ritchie M. E. Phipson B. Wu D. Hu Y. Law C. W. Shi W. (2015). limma powers differential expression analyses for RNA-sequencing and microarray studies. Nucleic Acids Res. 43 (7), e47. 10.1093/nar/gkv007 25605792 PMC4402510

[B74] Rohacs T. (2005). Teaching resources. TRP channels. Sci. STKE 2005 (282), tr14. 10.1126/stke.2822005tr14 15870425

[B75] Rojas-Aguirre Y. Medina-Franco J. L. (2014). Analysis of structure-Caco-2 permeability relationships using a property landscape approach. Mol. Divers. 18 (3), 599–610. 10.1007/s11030-014-9514-x 24710715

[B76] Romero-Hernández L. L. Merino-Montiel P. Montiel-Smith S. Meza-Reyes S. Vega-Báez J. L. Abasolo I. (2015). Diosgenin-based thio(seleno)ureas and triazolyl glycoconjugates as hybrid drugs. Antioxidant and antiproliferative profile. Eur. J. Med. Chem. 99, 67–81. 10.1016/j.ejmech.2015.05.018 26046314

[B77] Ru J. Li P. Wang J. Zhou W. Li B. Huang C. (2014). TCMSP: a database of systems pharmacology for drug discovery from herbal medicines. J. Cheminform 6 (1), 13–16. 10.1186/1758-2946-6-13 24735618 PMC4001360

[B78] Sanner M. F. (1999). Python: a programming language for software integration and development. J. Mol. Graph. Model. 17 (1), 57–61. 10660911

[B79] Schr Odinger L. D. D. (2015). The {PyMOL} Molecular Graphics System. Version∼1. 8.

[B80] Sekino Y. Han X. Babasaki T. Goto K. Inoue S. Hayashi T. (2020). Microtubule-associated protein tau (MAPT) promotes bicalutamide resistance and is associated with survival in prostate cancer. Urol. Oncol. Semin. Original Invest. 38, e1–795. 10.1016/j.urolonc.2020.04.032 32430253

[B81] Sergeev I. N. (2005). Calcium signaling in cancer and vitamin D. J. Steroid Biochem. Mol. Biol. 97 (1-2), 145–151. 10.1016/j.jsbmb.2005.06.007 16081284

[B82] Serna-Marquez N. Diaz-Aragon R. Reyes-Uribe E. Cortes-Reynosa P. Salazar E. P. (2017). Linoleic acid induces migration and invasion through FFAR4- and PI3K-/Akt-dependent pathway in MDA-MB-231 breast cancer cells. Med. Oncol. 34 (6), 111. 10.1007/s12032-017-0969-3 28456993

[B83] Shannon P. Markiel A. Ozier O. Baliga N. S. Wang J. T. Ramage D. (2003). Cytoscape: a software environment for integrated models of biomolecular interaction networks. Genome Res. 13 (11), 2498–2504. 10.1101/gr.1239303 14597658 PMC403769

[B84] Singh M. Alavi A. Wong R. Akita S. (2016). Radiodermatitis: A Review of Our Current Understanding. Am. J. Clin. Dermatol. 17 (3), 277–292. 10.1007/s40257-016-0186-4 27021652

[B85] Sterling T. Irwin J. J. (2015). ZINC 15--Ligand Discovery for Everyone. J. Chem. Inf. Model. 55 (11), 2324–2337. 10.1021/acs.jcim.5b00559 26479676 PMC4658288

[B86] Straub J. M. New J. Hamilton C. D. Lominska C. Shnayder Y. Thomas S. M. (2015). Radiation-induced fibrosis: mechanisms and implications for therapy. J. Cancer Res. Clin. Oncol. 141 (11), 1985–1994. 10.1007/s00432-015-1974-6 25910988 PMC4573901

[B87] Tao W. Xu X. Wang X. Li B. Wang Y. Li Y. (2013). Network pharmacology-based prediction of the active ingredients and potential targets of Chinese herbal Radix Curcumae formula for application to cardiovascular disease. J. Ethnopharmacol. 145 (1), 1–10. 10.1016/j.jep.2012.09.051 23142198

[B88] Tong W. G. Ding X. Z. Adrian T. E. (2002). The mechanisms of lipoxygenase inhibitor-induced apoptosis in human breast cancer cells. Biochem. Biophys. Res. Commun. 296 (4), 942–948. 10.1016/S0006-291X(02)02014-4 12200139

[B89] Trott O. Olson A. J. (2009). AutoDock Vina: Improving the speed and accuracy of docking with a new scoring function, efficient optimization, and multithreading. J. Comput. Chem., NA. 10.1002/jcc.21334 PMC3041641 19499576

[B90] Vaidya J. S. Massarut S. Vaidya H. J. Alexander E. C. Richards T. Caris J. A. (2018). Rethinking neoadjuvant chemotherapy for breast cancer. BMJ 360, j5913. 10.1136/bmj.j5913 29326104

[B91] Vangeel L. Voets T. (2019). Transient Receptor Potential Channels and Calcium Signaling. Cold Spring Harb Perspect. Biol. 11 (6). 10.1101/cshperspect.a035048 PMC6546042 30910771

[B92] Venkatachalam K. Montell C. (2007). TRP channels. Annu. Rev. Biochem. 76, 387–417. 10.1146/annurev.biochem.75.103004.142819 17579562 PMC4196875

[B93] Wang H. Hu H. Rong H. Zhao X. (2019). Effects of compound Kushen injection on pathology and angiogenesis of tumor tissues. Oncol. Lett. 17 (2), 2278–2282. 10.3892/ol.2018.9861 30719109 PMC6351733

[B94] Wang L. H. Zeng X. A. Wang M. S. Brennan C. S. Gong D. (2018). Modification of membrane properties and fatty acids biosynthesis-related genes in Escherichia coli and Staphylococcus aureus: Implications for the antibacterial mechanism of naringenin. Biochim. Biophys. Acta Biomembr 1860 (2), 481–490. 10.1016/j.bbamem.2017.11.007 29138066

[B95] Wang W. You R. L. Qin W. J. Hai L. N. Fang M. J. Huang G. H. (2015). Anti-tumor activities of active ingredients in Compound Kushen Injection. Acta Pharmacol. Sin. 36 (6), 676–679. 10.1038/aps.2015.24 25982630 PMC4594177

[B96] Wang Y. Y. Attané C. Milhas D. Dirat B. Dauvillier S. Guerard A. (2017). Mammary adipocytes stimulate breast cancer invasion through metabolic remodeling of tumor cells. JCI Insight 2 (4), e87489. 10.1172/jci.insight.87489 28239646 PMC5313068

[B97] Warnes G. R. Bolker B. Bonebakker L. Gentleman R. Huber W. Liaw A. (2020). gplots: Various R Programming Tools for Plotting Data. R package version 3.

[B98] Weingarten M. D. Lockwood A. H. Hwo S. Y. Kirschner M. W. (1975). A protein factor essential for microtubule assembly. Proc. Natl. Acad. Sci. U S A. 72 (5), 1858–1862. 10.1073/pnas.72.5.1858 1057175 PMC432646

[B99] Wickham H. (2016). ggplot2: Elegant Graphics for Data Analysis. New York: Springer-Verlag.

[B100] Wu K. Fukuda K. Xing F. Zhang Y. Sharma S. Liu Y. (2015). Roles of the Cyclooxygenase 2 Matrix Metalloproteinase 1 Pathway in Brain Metastasis of Breast Cancer. J. Biol. Chem. 290 (15), 9842–9854. 10.1074/jbc.M114.602185 25691572 PMC4392281

[B101] Xiao N. (2018). ggsci: Scientific Journal and Sci-Fi Themed Color Palettes for 'ggplot2'. R package version 2.9.

[B102] Xiumei Z. Cen S. (2004). Simultaneous determination of three Alkaloids in Compound Kushen Injection by HPLC. China J. Chin. Materia Med. (07), 99–100.

[B103] Xu W. Lin H. Zhang Y. Chen X. Hua B. Hou W. (2011a). Compound Kushen Injection suppresses human breast cancer stem-like cells by down-regulating the canonical Wnt/β-catenin pathway. J. Exp. Clin. Cancer Res. 30, 103. 10.1186/1756-9966-30-103 22032476 PMC3219673

[B104] Xu W. Lin H. Zhang Y. Chen X. Hua B. Hou W. (2011b). Compound Kushen Injection suppresses human breast cancer stem-like cells by down-regulating the canonical Wnt/β-catenin pathway. J. Exp. Clin. Cancer Res. 30, 103. 10.1186/1756-9966-30-103 22032476 PMC3219673

[B105] Yang Y. Sun M. Li W. Liu C. Jiang Z. Gu P. (2021). Rebalancing TGF-β/Smad7 signaling via Compound kushen injection in hepatic stellate cells protects against liver fibrosis and hepatocarcinogenesis. Clin. Transl Med. 11 (7), e410. 10.1002/ctm2.410 34323416 PMC8255064

[B106] Yao L. T. Wang M. Z. Wang M. S. Yu X. T. Guo J. Y. Sun T. (2019). Neoadjuvant endocrine therapy: A potential strategy for ER-positive breast cancer. World J. Clin. Cases 7 (15), 1937–1953. 10.12998/wjcc.v7.i15.1937 31423426 PMC6695538

[B107] Yao M. Fu P. (2018). Advances in anti-HER2 therapy in metastatic breast cancer. Chin. Clin. Oncol. 7 (3), 27. 10.21037/cco.2018.05.04 30056729

[B108] Yarla N. S. Bishayee A. Sethi G. Reddanna P. Kalle A. M. Dhananjaya B. L. (2016). Targeting arachidonic acid pathway by natural products for cancer prevention and therapy. Semin. Cancer Biol. 40-41, 48–81. 10.1016/j.semcancer.2016.02.001 26853158

[B109] Yu G. Wang L. G. Han Y. He Q. Y. (2012). clusterProfiler: an R Package for Comparing Biological Themes Among Gene Clusters. OMICS 16 (5), 284–287. 10.1089/omi.2011.0118 22455463 PMC3339379

[B110] Yuan T. L. Cantley L. C. (2008). PI3K pathway alterations in cancer: variations on a theme. Oncogene 27 (41), 5497–5510. 10.1038/onc.2008.245 18794884 PMC3398461

[B111] Yue M. (2012). [Study on Chemical Constituents and Quality Control of Compound Kushen Injection.

[B112] Zhang Y. (2017). Ganoderma lucidum (Reishi) suppresses proliferation and migration of breast cancer cells via inhibiting Wnt/β-catenin signaling. Biochem. Biophys. Res. Commun. 488 (4), 679–684. 10.1016/j.bbrc.2017.04.086 28427938

[B113] Zheng Y. Zhong G. Yu K. Lei K. Yang Q. (2020). Individualized Prediction of Survival Benefit from Locoregional Surgical Treatment for Patients with Metastatic Breast Cancer. Front. Oncol. 10, 148. 10.3389/fonc.2020.00148 32133290 PMC7040087

[B114] Zhou C. Wang M. Zhou L. Zhang Y. Liu W. Qin W. (2016). Prognostic significance of PLIN1 expression in human breast cancer. Oncotarget 7 (34), 54488–54502. 10.18632/oncotarget.10239 27359054 PMC5342357

