# Supplementary material for: A System Pharmacology Model for Decoding the Synergistic Mechanisms of Compound Kushen Injection in Treating Breast Cancer
Source: Front Pharmacol. 2021 Nov 16;12:723147. doi: 10.3389/fphar.2021.723147 (PMC8660088; doi:10.3389/fphar.2021.723147)
Supplement: Supplementary file 1 [file Table1.DOCX]

**Table S1 |** The information on chemical analysis of the components from the literature in CKI

| **Formula** | **Method** | **Component** | **Concentration** | **References** |
| --- | --- | --- | --- | --- |
| Compound Kushen Injection (CKI) | HPLC | Matrine | 4.275±1.965 mg/ml | Yue et al. [34] |
|  |  | Oxymatrine | 6.59±2.79 mg/g |  |
|  |  | Sophocarpine | 0.956±0.474 mg/g |  |
|  |  | Oxysophocarpine | 1.805±0.695 mg/g |  |
|  |  | N-Methylcytisine | 0.3025±0.1675 mg/g |  |
|  |  | Trifolirhizin | 0.11585±0.08815 mg/g |  |
| Compound Kushen Injection (CKI) | HPLC | Matrine | 2.012±0.269 mg/g | Liang et al. [35] |
|  |  | Oxymatrine | 2.411±0.165 mg/g |  |
|  |  | Sophoridine | 1.7475±0.4215 mg/g |  |
| Compound Kushen Injection (CKI) | HPLC | Matrine | 1.7165±0.6305 mg/g | Juan et al. [36] |
|  |  | Sophoridine | 0.6205±0.3445 mg/g |  |
