# Supplementary material for: A System Pharmacology Model for Decoding the Synergistic Mechanisms of Compound Kushen Injection in Treating Breast Cancer
Source: Front Pharmacol. 2021 Nov 16;12:723147. doi: 10.3389/fphar.2021.723147 (PMC8660088; doi:10.3389/fphar.2021.723147)
Supplement: Supplementary file 2 [file Table8.DOCX]

**Table S8 |** The result of TRI values of each components in HRDR

| Component | GI | AI | NI | | | | | | | | SCD | TRI |
| --- | --- | --- | --- | --- | --- | --- | --- | --- | --- | --- | --- | --- |
|  |  |  | Average Shortest  Path Length | Betweenness  Centrality | Closeness  Centrality | Degree | Eccentricity | Neighborhood  Connectivity | Radiality | Topological  Coefficient |  |  |
| KS20 | 23 | -7.7222 | 3.0077 | 0.0182 | 0.3325 | 38 | 5 | 4.9500 | 0.9865 | 0.1881 | 0.6409 | 1.0818 |
| KS91 | 29 | -8.7388 | 2.9924 | 0.0541 | 0.3342 | 41 | 5 | 4.4634 | 0.9866 | 0.2037 | 0.0173 | 1.0124 |
| BTL3 | 29 | -8.4446 | 2.7661 | 0.0833 | 0.3615 | 78 | 5 | 4.1026 | 0.9882 | 0.1410 | 0.0177 | 0.8135 |
| KS1 | 28 | -8.3104 | 2.6223 | 0.0704 | 0.3813 | 125 | 5 | 6.5200 | 0.9891 | 0.2509 | 0.0246 | 0.7755 |
| KS2 | 28 | -8.2324 | 2.6101 | 0.0764 | 0.3831 | 129 | 5 | 6.5194 | 0.9892 | 0.2509 | 0.0306 | 0.7464 |
| KS53 | 29 | -8.1606 | 2.7936 | 0.1036 | 0.3580 | 83 | 5 | 4.3253 | 0.9880 | 0.1663 | 0.0231 | 0.6957 |
| KS77 | 28 | -8.1167 | 2.6651 | 0.0992 | 0.3752 | 130 | 5 | 5.8846 | 0.9888 | 0.2571 | 0.0325 | 0.6788 |
| BTL1 | 28 | -8.0493 | 2.5245 | 0.0986 | 0.3961 | 144 | 3 | 6.0347 | 0.9898 | 0.2189 | 0.0167 | 0.5203 |
| KS3 | 28 | -8.0493 | 2.5245 | 0.0986 | 0.3961 | 144 | 3 | 6.0347 | 0.9898 | 0.2189 | 0.0167 | 0.5203 |
| BTL4 | 28 | -8.2222 | 2.7783 | 0.0755 | 0.3599 | 76 | 5 | 4.0132 | 0.9881 | 0.1435 | 0.0148 | 0.5031 |
| KS4 | 27 | -7.9397 | 2.6835 | 0.1096 | 0.3727 | 105 | 5 | 5.6571 | 0.9887 | 0.2117 | 0.0413 | 0.4390 |
| KS52 | 27 | -8.0127 | 2.7385 | 0.0614 | 0.3652 | 87 | 5 | 6.6207 | 0.9883 | 0.2555 | 0.0123 | 0.4236 |
| KS16 | 24 | -7.7905 | 3.0719 | 0.0172 | 0.3255 | 20 | 5 | 4.2500 | 0.9861 | 0.1711 | 0.1965 | 0.3958 |
| KS70 | 27 | -7.9986 | 2.7936 | 0.0774 | 0.3580 | 69 | 5 | 5.5507 | 0.9880 | 0.2069 | 0.0158 | 0.3749 |
| KS11 | 23 | -7.7857 | 3.1422 | 0.0192 | 0.3183 | 17 | 5 | 4.1765 | 0.9856 | 0.1672 | 0.1890 | 0.3577 |
| KS90 | 27 | -7.9870 | 2.8395 | 0.0734 | 0.3522 | 68 | 5 | 4.9265 | 0.9877 | 0.1963 | 0.0151 | 0.3505 |
| BTL2 | 27 | -8.1507 | 2.7966 | 0.0732 | 0.3576 | 57 | 5 | 3.4386 | 0.9879 | 0.1109 | 0.0095 | 0.3113 |
| KS92 | 25 | -7.8408 | 4.1025 | 0.0378 | 0.2438 | 21 | 5 | 2.2381 | 0.9792 | 0.1548 | 0.0480 | 0.1576 |
| BTL15 | 5 | -6.9429 | 2.5489 | 0.2507 | 0.3923 | 149 | 5 | 3.0738 | 0.9896 | 0.0943 | 0.0792 | 0.1107 |
| KS19 | 23 | -7.3460 | 2.8242 | 0.0461 | 0.3541 | 59 | 5 | 6.5932 | 0.9878 | 0.2542 | 0.0099 | 0.0834 |
| BTL19 | 3 | -7.1000 | 2.7630 | 0.0699 | 0.3619 | 79 | 5 | 4.2025 | 0.9882 | 0.1456 | 0.0171 | 0.0121 |
| BTL8 | 3 | -6.8333 | 2.8272 | 0.0652 | 0.3537 | 80 | 5 | 3.8125 | 0.9877 | 0.1480 | 0.0098 | 0.0005 |
