# Supplementary figures and images for: A System Pharmacology Model for Decoding the Synergistic Mechanisms of Compound Kushen Injection in Treating Breast Cancer
Source: Front Pharmacol. 2021 Nov 16;12:723147. doi: 10.3389/fphar.2021.723147 (PMC8660088; doi:10.3389/fphar.2021.723147)

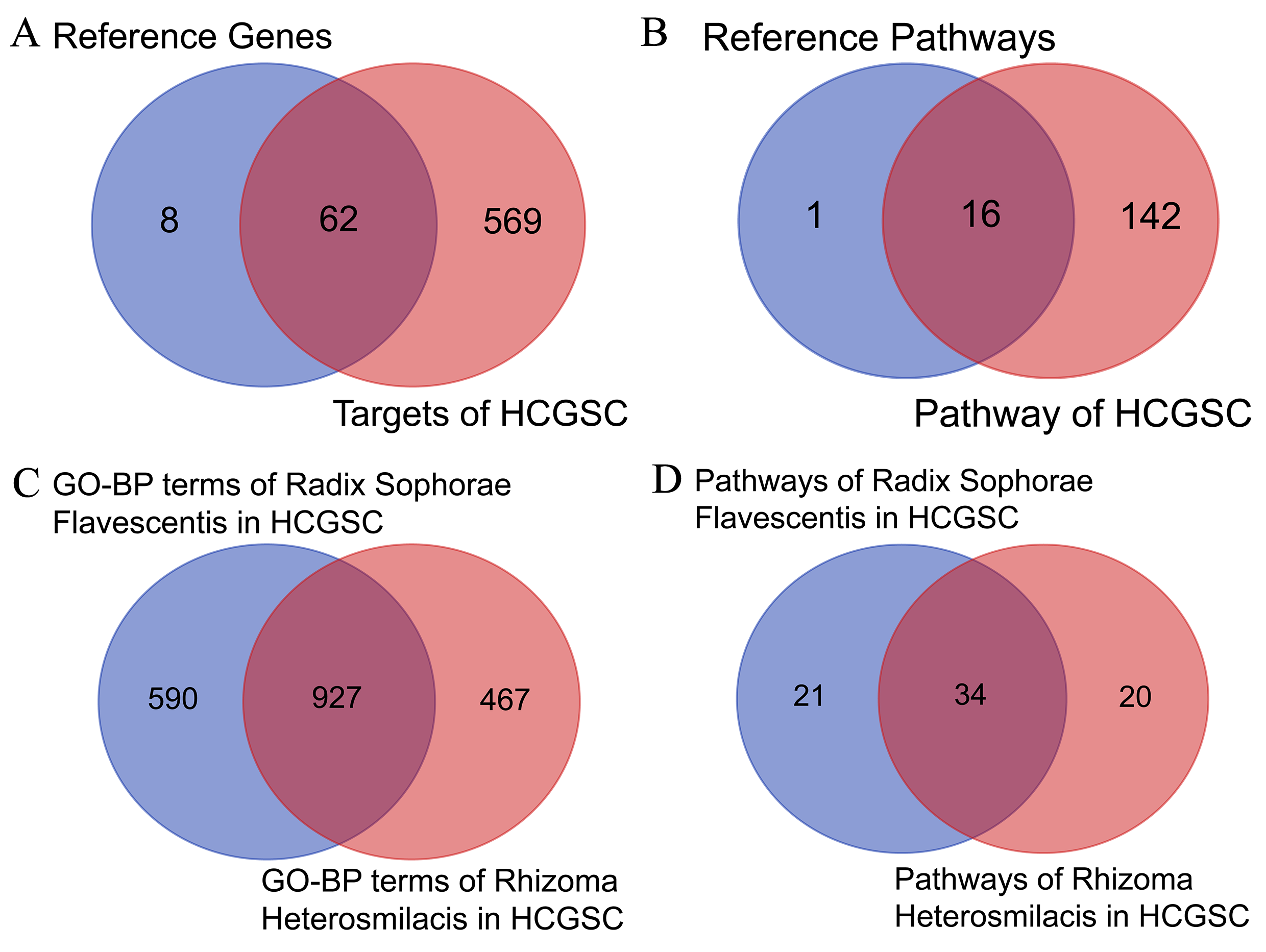

Supplement: Supplementary file 4 [file Image4.TIF]

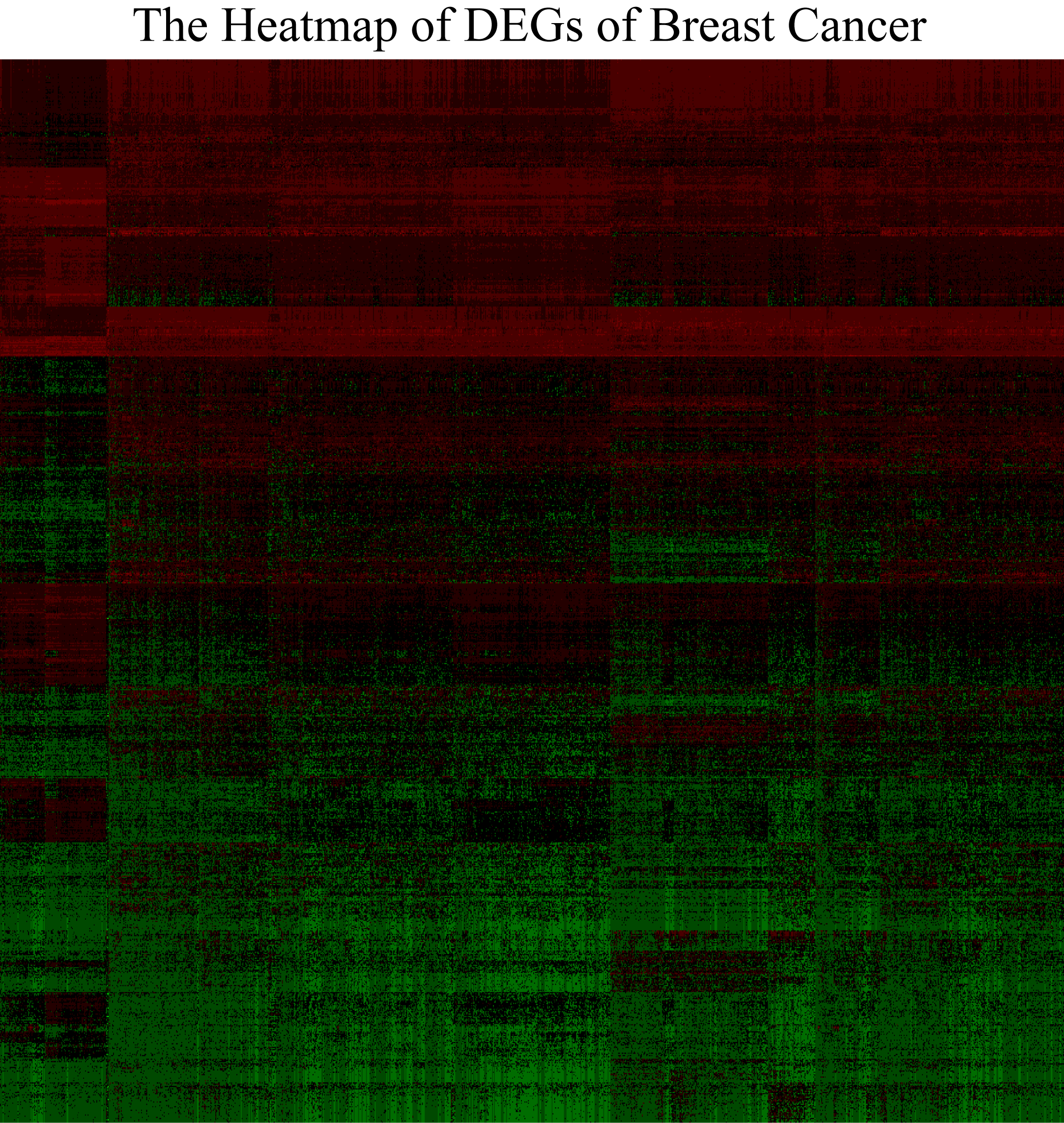

Supplement: Supplementary file 5 [file Image2.TIF]

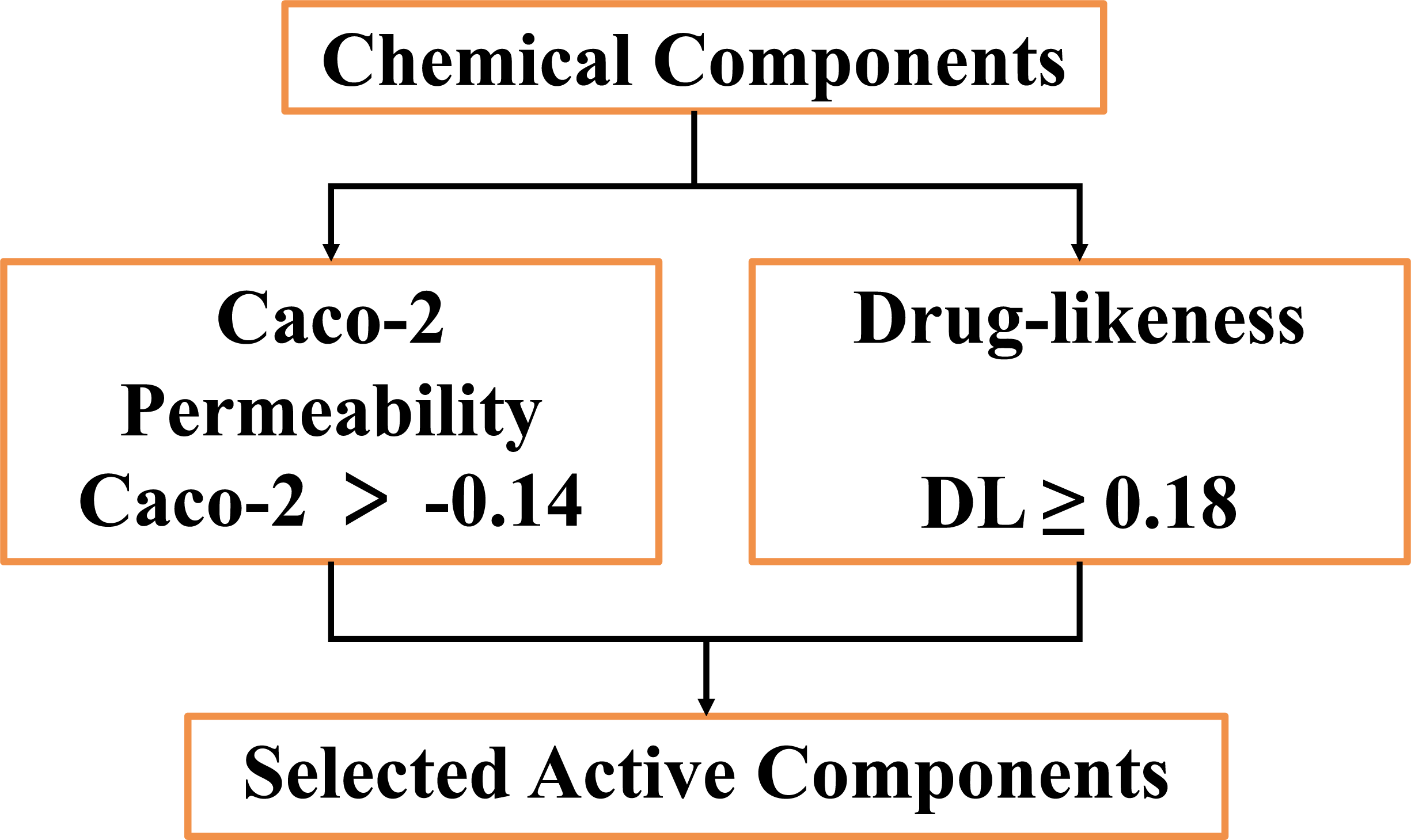

Supplement: Supplementary file 6 [file Image1.TIF]
