# Supplementary material for: A System Pharmacology Model for Decoding the Synergistic Mechanisms of Compound Kushen Injection in Treating Breast Cancer
Source: Front Pharmacol. 2021 Nov 16;12:723147. doi: 10.3389/fphar.2021.723147 (PMC8660088; doi:10.3389/fphar.2021.723147)
Supplement: Supplementary file 7 [file Table2.DOCX]

**Table S2 |** The detail information of DEGs of BC.

| **Gene Symbol** | **FoldChange** | **Log2**  **FoldChange** | **P value** | **P.adjust value** |
| --- | --- | --- | --- | --- |
| MMP11 | 76.44654535 | 6.256379402 | 1.38E-114 | 4.72E-110 |
| COL10A1 | 139.7980444 | 7.127200369 | 7.01E-97 | 1.20E-92 |
| CAV1 | 0.095210835 | -3.392730433 | 2.18E-89 | 2.48E-85 |
| GSN | 0.172622593 | -2.534306796 | 2.15E-88 | 1.83E-84 |
| OXTR | 0.059072815 | -4.081361824 | 2.07E-79 | 1.41E-75 |
| CHRDL1 | 0.045674927 | -4.452453761 | 1.23E-72 | 7.00E-69 |
| COL11A1 | 73.04300206 | 6.190674156 | 3.33E-71 | 1.62E-67 |
| SYNM | 0.117983589 | -3.083341893 | 3.21E-68 | 1.24E-64 |
| DST | 0.192660373 | -2.375868233 | 3.27E-68 | 1.24E-64 |
| SCARA5 | 0.029484827 | -5.083883483 | 3.96E-65 | 1.35E-61 |
| LYVE1 | 0.031537827 | -4.986772912 | 4.50E-65 | 1.40E-61 |
| ITIH5 | 0.061957498 | -4.012577303 | 2.58E-64 | 7.33E-61 |
| EGR1 | 0.155571502 | -2.684350288 | 3.00E-63 | 7.27E-60 |
| TNS1 | 0.115798687 | -3.110309198 | 3.19E-63 | 7.27E-60 |
| CD300LG | 0.030879638 | -5.017200341 | 1.51E-62 | 3.22E-59 |
| MME | 0.103033274 | -3.278817769 | 1.43E-61 | 2.87E-58 |
| TXNIP | 0.241407286 | -2.050458876 | 2.77E-60 | 5.25E-57 |
| TNXB | 0.057467785 | -4.121102749 | 3.48E-59 | 6.25E-56 |
| CD36 | 0.055113078 | -4.181461481 | 5.36E-56 | 9.15E-53 |
| CAVIN2 | 0.063830065 | -3.969620065 | 3.74E-55 | 6.08E-52 |
| TRARG1 | 0.029804984 | -5.068302586 | 2.01E-54 | 3.12E-51 |
| PLIN1 | 0.025295797 | -5.304958507 | 2.25E-54 | 3.24E-51 |
| FN1 | 7.257358607 | 2.859444559 | 2.28E-54 | 3.24E-51 |
| ANXA1 | 0.197292731 | -2.341590288 | 1.15E-53 | 1.56E-50 |
| KIT | 0.129686155 | -2.946903628 | 2.76E-53 | 3.58E-50 |
| SORBS1 | 0.078305579 | -3.674741095 | 2.83E-53 | 3.58E-50 |
| AOC3 | 0.063157026 | -3.984912963 | 1.15E-52 | 1.41E-49 |
| SVEP1 | 0.102631508 | -3.284454391 | 2.83E-52 | 3.33E-49 |
| PCOLCE2 | 0.041197792 | -4.601289181 | 8.03E-52 | 9.13E-49 |
| ITGA7 | 0.078840628 | -3.664916923 | 2.00E-51 | 2.20E-48 |
| FABP4 | 0.033677655 | -4.892064522 | 2.63E-51 | 2.81E-48 |
| LEP | 0.014032434 | -6.155090936 | 9.93E-50 | 1.03E-46 |
| BTNL9 | 0.062189267 | -4.007190585 | 2.86E-49 | 2.87E-46 |
| GPD1 | 0.025573283 | -5.289218783 | 3.21E-49 | 3.13E-46 |
| CIDEC | 0.025587114 | -5.288438779 | 4.26E-49 | 4.03E-46 |
| COMP | 22.59208786 | 4.497745699 | 4.59E-49 | 4.24E-46 |
| PLIN4 | 0.027640553 | -5.177069699 | 4.04E-48 | 3.62E-45 |
| RBP4 | 0.032930479 | -4.924432706 | 4.78E-47 | 4.18E-44 |
| KCNIP2 | 0.028486749 | -5.133565227 | 8.58E-47 | 7.32E-44 |
| SPTBN1 | 0.241910682 | -2.047453619 | 1.10E-46 | 9.16E-44 |
| GPX3 | 0.076948093 | -3.69997062 | 3.42E-46 | 2.78E-43 |
| AQP7 | 0.028381368 | -5.138912041 | 4.54E-46 | 3.60E-43 |
| SAA1 | 0.087251246 | -3.518680452 | 6.51E-46 | 5.05E-43 |
| CNN1 | 0.112206392 | -3.155773225 | 8.81E-46 | 6.68E-43 |
| ABCA8 | 0.066749631 | -3.905096335 | 1.60E-45 | 1.19E-42 |
| GLYAT | 0.016514471 | -5.920125443 | 2.16E-45 | 1.57E-42 |
| LPL | 0.038049498 | -4.715978776 | 2.48E-45 | 1.76E-42 |
| FMO2 | 0.106751077 | -3.227677467 | 3.11E-45 | 2.16E-42 |
| LIPE | 0.042803516 | -4.546126883 | 5.59E-45 | 3.82E-42 |
| MYLK | 0.216206647 | -2.209517218 | 9.33E-45 | 6.25E-42 |
| COL17A1 | 0.142217693 | -2.813827135 | 3.94E-44 | 2.59E-41 |
| TMEM132C | 0.038021629 | -4.717035835 | 1.52E-43 | 9.82E-41 |
| CLEC3B | 0.066832773 | -3.903300456 | 2.68E-43 | 1.69E-40 |
| PI16 | 0.078020979 | -3.679994088 | 3.01E-43 | 1.86E-40 |
| FOSB | 0.092634257 | -3.432310379 | 3.91E-43 | 2.38E-40 |
| FOS | 0.168136698 | -2.572293451 | 1.94E-42 | 1.16E-39 |
| SLC19A3 | 0.030942823 | -5.014251357 | 2.73E-42 | 1.61E-39 |
| G0S2 | 0.059155153 | -4.079352337 | 3.19E-42 | 1.85E-39 |
| TRHDE-AS1 | 0.022335102 | -5.484543368 | 6.65E-42 | 3.79E-39 |
| GYG2 | 0.057303501 | -4.125232899 | 9.09E-42 | 5.09E-39 |
| TIMP4 | 0.043294205 | -4.529682252 | 1.01E-41 | 5.56E-39 |
| DDR2 | 0.156389086 | -2.676788257 | 1.79E-41 | 9.70E-39 |
| MYH11 | 0.106984637 | -3.22452445 | 2.53E-41 | 1.35E-38 |
| LRRC15 | 14.13746906 | 3.821451962 | 4.30E-41 | 2.26E-38 |
| CAV2 | 0.133398031 | -2.906190728 | 7.04E-41 | 3.64E-38 |
| ACACB | 0.078995271 | -3.662089898 | 8.61E-41 | 4.39E-38 |
| CX3CL1 | 0.19225798 | -2.378884617 | 1.06E-40 | 5.33E-38 |
| VWF | 0.234473302 | -2.092504432 | 1.11E-40 | 5.49E-38 |
| NPR1 | 0.081580778 | -3.615626918 | 1.87E-40 | 9.13E-38 |
| PPP1R1A | 0.067773128 | -3.883142829 | 2.31E-40 | 1.11E-37 |
| KLB | 0.038129361 | -4.712953852 | 2.66E-40 | 1.26E-37 |
| DPT | 0.086930248 | -3.523997932 | 3.12E-40 | 1.46E-37 |
| FZD4 | 0.155771549 | -2.682496336 | 1.68E-39 | 7.74E-37 |
| ADIPOQ | 0.027074497 | -5.206921635 | 1.94E-39 | 8.82E-37 |
| SFRP1 | 0.171733393 | -2.541757502 | 3.04E-39 | 1.36E-36 |
| ADH1B | 0.028849957 | -5.115287033 | 3.06E-39 | 1.36E-36 |
| MAOA | 0.099131462 | -3.334513178 | 3.73E-39 | 1.63E-36 |
| GPAM | 0.045937804 | -4.444174298 | 5.38E-39 | 2.33E-36 |
| TOP2A | 11.34657283 | 3.5041847 | 1.34E-38 | 5.66E-36 |
| ACVR1C | 0.026983936 | -5.211755404 | 3.40E-38 | 1.42E-35 |
| TGFBR2 | 0.21540466 | -2.214878636 | 5.44E-38 | 2.21E-35 |
| VEGFD | 0.033673009 | -4.892263538 | 7.56E-38 | 3.03E-35 |
| MFAP4 | 0.178495615 | -2.486039463 | 1.65E-37 | 6.56E-35 |
| ZFP36 | 0.215321374 | -2.215436557 | 2.00E-37 | 7.86E-35 |
| AQP1 | 0.232791151 | -2.102891879 | 2.77E-37 | 1.07E-34 |
| POSTN | 4.427617377 | 2.146530554 | 5.46E-37 | 2.09E-34 |
| GHR | 0.113409467 | -3.140387019 | 6.40E-37 | 2.43E-34 |
| PALMD | 0.134579147 | -2.893473211 | 7.56E-37 | 2.83E-34 |
| CA4 | 0.025383327 | -5.299975035 | 7.98E-37 | 2.96E-34 |
| AKR1C1 | 0.090234 | -3.470185056 | 1.17E-36 | 4.31E-34 |
| ENPP2 | 0.150460479 | -2.732543505 | 1.30E-36 | 4.73E-34 |
| BGN | 5.030509037 | 2.330704394 | 1.42E-36 | 5.10E-34 |
| NDRG2 | 0.217798087 | -2.198936814 | 1.48E-36 | 5.27E-34 |
| PDK4 | 0.082566798 | -3.598294436 | 2.75E-36 | 9.68E-34 |
| DUSP1 | 0.247932157 | -2.011982693 | 8.41E-36 | 2.93E-33 |
| ACSL1 | 0.144286481 | -2.792991961 | 9.36E-36 | 3.23E-33 |
| ALDH2 | 0.204091052 | -2.292715166 | 1.36E-35 | 4.63E-33 |
| TGFBR3 | 0.179716039 | -2.476208929 | 1.38E-35 | 4.66E-33 |
| MCAM | 0.219925923 | -2.184910429 | 1.43E-35 | 4.80E-33 |
| CCDC69 | 0.126188175 | -2.986351369 | 7.31E-35 | 2.42E-32 |
| AKAP12 | 0.152329029 | -2.714737195 | 8.45E-35 | 2.77E-32 |
| APOB | 0.022095687 | -5.500091415 | 1.30E-34 | 4.24E-32 |
| C14orf180 | 0.028552321 | -5.130248182 | 1.48E-34 | 4.76E-32 |
| MUC1 | 5.774288622 | 2.529643221 | 3.03E-34 | 9.65E-32 |
| COL1A1 | 4.773004974 | 2.25489784 | 6.95E-34 | 2.19E-31 |
| SEMA3G | 0.138231991 | -2.854836563 | 1.27E-33 | 3.93E-31 |
| LIFR | 0.170425479 | -2.552787057 | 1.80E-33 | 5.53E-31 |
| PRKAR2B | 0.178079631 | -2.489405586 | 2.33E-33 | 7.11E-31 |
| PDE3B | 0.072600635 | -3.783874022 | 2.54E-33 | 7.66E-31 |
| SLIT3 | 0.186815248 | -2.420315879 | 3.40E-33 | 1.02E-30 |
| MTURN | 0.170092845 | -2.555605642 | 6.31E-33 | 1.87E-30 |
| DMD | 0.124384006 | -3.007127112 | 9.11E-33 | 2.66E-30 |
| PPARG | 0.095502889 | -3.388311815 | 1.23E-32 | 3.54E-30 |
| ADAMTS5 | 0.120223124 | -3.056213677 | 1.36E-32 | 3.87E-30 |
| HSPB6 | 0.041261664 | -4.599054182 | 2.28E-32 | 6.39E-30 |
| MATN2 | 0.172110951 | -2.538589204 | 2.95E-32 | 8.19E-30 |
| IGFBP6 | 0.107592615 | -3.216349034 | 3.71E-32 | 1.01E-29 |
| DEPP1 | 0.212789585 | -2.232500554 | 5.17E-32 | 1.40E-29 |
| LGALS12 | 0.04239882 | -4.559832067 | 5.41E-32 | 1.45E-29 |
| PAMR1 | 0.086996003 | -3.522907076 | 5.56E-32 | 1.48E-29 |
| PLXNA4 | 0.098064044 | -3.350131938 | 7.22E-32 | 1.91E-29 |
| MGLL | 0.213113873 | -2.230303587 | 1.63E-31 | 4.26E-29 |
| LVRN | 0.029658157 | -5.075427223 | 2.16E-31 | 5.58E-29 |
| NTRK2 | 0.164849155 | -2.6007816 | 4.50E-31 | 1.15E-28 |
| GPRC5A | 5.150021064 | 2.364578333 | 1.01E-30 | 2.56E-28 |
| SIK2 | 0.182373275 | -2.455033759 | 1.79E-30 | 4.50E-28 |
| ABCA9 | 0.081586691 | -3.615522354 | 2.84E-30 | 7.03E-28 |
| GALNT15 | 0.108019372 | -3.210638031 | 3.26E-30 | 8.00E-28 |
| PCK1 | 0.026421777 | -5.242128673 | 3.84E-30 | 9.36E-28 |
| CES1 | 0.072655006 | -3.782793983 | 9.12E-30 | 2.19E-27 |
| CENPF | 9.627310432 | 3.267132811 | 1.54E-29 | 3.67E-27 |
| CCDC3 | 0.141648253 | -2.819615282 | 2.46E-29 | 5.84E-27 |
| LMOD1 | 0.158173551 | -2.660419713 | 6.49E-29 | 1.53E-26 |
| DGAT2 | 0.099500944 | -3.329145971 | 7.38E-29 | 1.73E-26 |
| EBF1 | 0.119437814 | -3.06566843 | 9.36E-29 | 2.17E-26 |
| SLC7A10 | 0.038064513 | -4.715409555 | 1.01E-28 | 2.32E-26 |
| ADAMTS1 | 0.210783426 | -2.246166661 | 1.02E-28 | 2.35E-26 |
| S100A14 | 6.35879804 | 2.668754089 | 1.29E-28 | 2.93E-26 |
| CFD | 0.118582842 | -3.076032813 | 1.41E-28 | 3.19E-26 |
| CIDEA | 0.032365046 | -4.949419618 | 1.57E-28 | 3.52E-26 |
| SYNE3 | 0.124477942 | -3.006037983 | 2.36E-28 | 5.26E-26 |
| KLF4 | 0.167697677 | -2.576065394 | 3.51E-28 | 7.78E-26 |
| TPX2 | 11.87752682 | 3.570162559 | 4.52E-28 | 9.96E-26 |
| MMD | 0.139741272 | -2.839169922 | 4.61E-28 | 1.01E-25 |
| MMRN1 | 0.096130176 | -3.378866817 | 8.51E-28 | 1.85E-25 |
| CD34 | 0.229147353 | -2.125652472 | 1.21E-27 | 2.61E-25 |
| SLC16A7 | 0.095856847 | -3.3829747 | 1.83E-27 | 3.93E-25 |
| CCL28 | 0.188210786 | -2.409578784 | 1.94E-27 | 4.11E-25 |
| NAT8L | 0.107668912 | -3.215326345 | 2.38E-27 | 5.02E-25 |
| CDO1 | 0.079938959 | -3.644957406 | 3.89E-27 | 8.15E-25 |
| LEPR | 0.127235209 | -2.974430146 | 9.22E-27 | 1.91E-24 |
| HEPACAM | 0.0262194 | -5.253221513 | 1.09E-26 | 2.24E-24 |
| PDZD2 | 0.1612119 | -2.632969855 | 1.67E-26 | 3.38E-24 |
| MKI67 | 8.504524511 | 3.088230577 | 1.84E-26 | 3.69E-24 |
| MYBL2 | 14.25638642 | 3.833536442 | 3.32E-26 | 6.62E-24 |
| HSD17B13 | 0.029644319 | -5.076100523 | 3.73E-26 | 7.41E-24 |
| INHBA | 10.63429711 | 3.410652774 | 4.33E-26 | 8.55E-24 |
| GJB2 | 19.66114464 | 4.29727541 | 4.55E-26 | 8.92E-24 |
| CRYAB | 0.141246925 | -2.82370863 | 1.18E-25 | 2.27E-23 |
| ECM2 | 0.16318807 | -2.615392503 | 1.32E-25 | 2.53E-23 |
| PDE2A | 0.113741375 | -3.136170941 | 2.32E-25 | 4.41E-23 |
| CAT | 0.244110415 | -2.034394245 | 2.94E-25 | 5.54E-23 |
| ALDH1A1 | 0.170146315 | -2.555152192 | 2.97E-25 | 5.56E-23 |
| FP325317.1 | 0.024977488 | -5.323227803 | 3.04E-25 | 5.67E-23 |
| CLMP | 0.186435972 | -2.42324785 | 1.03E-24 | 1.91E-22 |
| NKAIN1 | 15.79217704 | 3.981138164 | 2.78E-24 | 5.13E-22 |
| MTARC1 | 0.149977846 | -2.737178691 | 2.86E-24 | 5.24E-22 |
| CST1 | 122.9957728 | 6.942464922 | 2.90E-24 | 5.30E-22 |
| SPX | 0.027273121 | -5.196376369 | 3.83E-24 | 6.94E-22 |
| TMEM37 | 0.124433974 | -3.006547659 | 6.72E-24 | 1.20E-21 |
| AC087482.1 | 0.021650182 | -5.52947706 | 1.04E-23 | 1.84E-21 |
| UBE2C | 18.22953188 | 4.188205609 | 1.05E-23 | 1.84E-21 |
| ALDH1L1 | 0.037391029 | -4.741164001 | 1.23E-23 | 2.15E-21 |
| CHL1 | 0.126061155 | -2.987804311 | 1.78E-23 | 3.10E-21 |
| BMPR1B | 18.8141767 | 4.233748254 | 3.61E-23 | 6.26E-21 |
| CPED1 | 0.133250285 | -2.907789477 | 4.15E-23 | 7.15E-21 |
| PPP1R12B | 0.233548058 | -2.098208643 | 4.95E-23 | 8.49E-21 |
| TP63 | 0.170694586 | -2.550510791 | 7.62E-23 | 1.30E-20 |
| AL845331.2 | 0.025491205 | -5.293856641 | 8.33E-23 | 1.41E-20 |
| GPIHBP1 | 0.087050629 | -3.522001472 | 9.07E-23 | 1.53E-20 |
| ABCD2 | 0.05786846 | -4.111078935 | 1.48E-22 | 2.47E-20 |
| IL33 | 0.152547674 | -2.712667915 | 2.25E-22 | 3.75E-20 |
| ATF3 | 0.240318869 | -2.056978165 | 2.49E-22 | 4.12E-20 |
| AC002546.1 | 0.030888917 | -5.01676689 | 2.78E-22 | 4.56E-20 |
| SH3D19 | 0.246811386 | -2.018519146 | 6.95E-22 | 1.12E-19 |
| MRAP | 0.038581133 | -4.695960668 | 1.19E-21 | 1.89E-19 |
| RRM2 | 10.63564786 | 3.410836011 | 1.30E-21 | 2.06E-19 |
| AQP7P1 | 0.030594628 | -5.030577809 | 1.70E-21 | 2.69E-19 |
| COX6C | 6.841683799 | 2.774351429 | 1.74E-21 | 2.74E-19 |
| CRABP2 | 4.410459132 | 2.140928849 | 1.81E-21 | 2.84E-19 |
| RDH5 | 0.051469744 | -4.280131592 | 2.33E-21 | 3.64E-19 |
| ACKR1 | 0.198616601 | -2.33194188 | 2.59E-21 | 4.00E-19 |
| BIRC5 | 11.85327815 | 3.567214202 | 2.98E-21 | 4.58E-19 |
| MRAS | 0.214914248 | -2.218166961 | 5.85E-21 | 8.92E-19 |
| CARMN | 0.135824131 | -2.880188282 | 6.85E-21 | 1.03E-18 |
| PER1 | 0.238166465 | -2.069957805 | 7.12E-21 | 1.07E-18 |
| SLC44A4 | 5.871139664 | 2.553640576 | 7.59E-21 | 1.13E-18 |
| HAS3 | 0.134463467 | -2.894713847 | 8.64E-21 | 1.28E-18 |
| SRPX | 0.176142397 | -2.505185889 | 9.80E-21 | 1.44E-18 |
| AP001528.2 | 0.082488808 | -3.599657802 | 9.95E-21 | 1.45E-18 |
| HLF | 0.138630676 | -2.850681567 | 1.19E-20 | 1.72E-18 |
| SAA2-SAA4 | 0.075057474 | -3.735860461 | 1.28E-20 | 1.86E-18 |
| TK1 | 8.476737551 | 3.08350912 | 1.31E-20 | 1.88E-18 |
| DEFB132 | 0.0335579 | -4.89720375 | 1.64E-20 | 2.36E-18 |
| FAM13A | 0.182449023 | -2.454434673 | 1.78E-20 | 2.54E-18 |
| MAMDC2 | 0.119548907 | -3.064327156 | 1.86E-20 | 2.65E-18 |
| EGR3 | 0.230105429 | -2.11963307 | 2.07E-20 | 2.93E-18 |
| FOXM1 | 11.04887165 | 3.465827138 | 2.32E-20 | 3.27E-18 |
| MLXIPL | 0.086066079 | -3.538411444 | 2.81E-20 | 3.94E-18 |
| IQGAP3 | 12.98811208 | 3.699119834 | 3.04E-20 | 4.26E-18 |
| ALDH1L1-AS2 | 0.029891017 | -5.064144189 | 3.61E-20 | 5.03E-18 |
| THRSP | 0.143966156 | -2.7961984 | 4.30E-20 | 5.91E-18 |
| FGF2 | 0.151364504 | -2.723901172 | 4.85E-20 | 6.65E-18 |
| OGN | 0.190908535 | -2.38904649 | 6.00E-20 | 8.16E-18 |
| TSHZ2 | 0.192424551 | -2.377635212 | 6.84E-20 | 9.27E-18 |
| S100P | 27.05175024 | 4.757650034 | 9.88E-20 | 1.33E-17 |
| PITX1 | 13.99559632 | 3.806901053 | 1.11E-19 | 1.48E-17 |
| CXCL2 | 0.092838273 | -3.429136508 | 1.32E-19 | 1.75E-17 |
| ANLN | 10.37655762 | 3.37525601 | 1.55E-19 | 2.06E-17 |
| AC044784.1 | 104.70456 | 6.710180464 | 1.70E-19 | 2.24E-17 |
| SAMD5 | 0.127965797 | -2.966169839 | 1.75E-19 | 2.30E-17 |
| PLAC9 | 0.117376775 | -3.090781117 | 1.76E-19 | 2.30E-17 |
| CNTNAP2 | 15.55783835 | 3.959569717 | 2.78E-19 | 3.58E-17 |
| SLC2A4 | 0.039626709 | -4.657383036 | 3.21E-19 | 4.12E-17 |
| ACO1 | 0.242629548 | -2.04317284 | 3.92E-19 | 5.00E-17 |
| ADRA1A | 0.042357539 | -4.561237431 | 4.10E-19 | 5.20E-17 |
| NAT1 | 9.448809623 | 3.240132588 | 4.40E-19 | 5.56E-17 |
| KPNA2 | 4.471479779 | 2.160752352 | 4.74E-19 | 5.97E-17 |
| GPC3 | 0.173044237 | -2.530787198 | 4.91E-19 | 6.16E-17 |
| CDC20 | 11.65934215 | 3.543414485 | 5.02E-19 | 6.27E-17 |
| NEK2 | 19.01317459 | 4.248927531 | 5.13E-19 | 6.38E-17 |
| RNF150 | 0.161368133 | -2.631572396 | 5.62E-19 | 6.97E-17 |
| LINC02237 | 0.027534128 | -5.182635274 | 6.00E-19 | 7.42E-17 |
| AQP7P2 | 0.022786467 | -5.455678915 | 6.85E-19 | 8.38E-17 |
| ADH1A | 0.036454866 | -4.777744798 | 6.98E-19 | 8.51E-17 |
| TMTC1 | 0.213769375 | -2.225872914 | 7.09E-19 | 8.58E-17 |
| ITM2A | 0.211391159 | -2.242013053 | 7.09E-19 | 8.58E-17 |
| LHFPL6 | 0.24226733 | -2.045328227 | 8.20E-19 | 9.89E-17 |
| ADIPOQ-AS1 | 0.027163568 | -5.202183187 | 9.39E-19 | 1.12E-16 |
| PRELP | 0.238647635 | -2.067046055 | 9.75E-19 | 1.16E-16 |
| SYT13 | 31.87030995 | 4.994141145 | 1.24E-18 | 1.47E-16 |
| AKR1C3 | 0.193588209 | -2.368937014 | 1.54E-18 | 1.81E-16 |
| CXCL9 | 8.964370265 | 3.164202239 | 1.60E-18 | 1.87E-16 |
| ACSM5 | 0.073871768 | -3.758833087 | 1.62E-18 | 1.88E-16 |
| SOD3 | 0.201495863 | -2.31117788 | 2.24E-18 | 2.59E-16 |
| ANGPTL7 | 0.058349748 | -4.099129763 | 2.86E-18 | 3.29E-16 |
| EGR2 | 0.218960871 | -2.191255017 | 4.25E-18 | 4.81E-16 |
| STARD10 | 4.40414072 | 2.138860566 | 4.42E-18 | 5.00E-16 |
| HBA2 | 0.045503166 | -4.457889254 | 6.24E-18 | 7.01E-16 |
| HSD11B1-AS1 | 0.048121427 | -4.377176757 | 8.43E-18 | 9.34E-16 |
| LINC01697 | 0.053720995 | -4.218370164 | 9.52E-18 | 1.05E-15 |
| AGR2 | 5.336160079 | 2.415801946 | 1.05E-17 | 1.15E-15 |
| FAM107A | 0.159460766 | -2.648726589 | 1.09E-17 | 1.20E-15 |
| AK5 | 0.193930158 | -2.366390919 | 1.15E-17 | 1.26E-15 |
| GNG11 | 0.221742075 | -2.17304555 | 1.21E-17 | 1.32E-15 |
| KCNJ3 | 36.21098536 | 5.17835553 | 1.70E-17 | 1.85E-15 |
| KIF4A | 14.31183168 | 3.83913642 | 1.76E-17 | 1.91E-15 |
| KLHL13 | 0.164246521 | -2.606065286 | 1.94E-17 | 2.10E-15 |
| CDK1 | 8.904057054 | 3.154462837 | 2.25E-17 | 2.41E-15 |
| CTHRC1 | 5.369436855 | 2.424770787 | 2.61E-17 | 2.80E-15 |
| LINC01230 | 0.03788377 | -4.722276283 | 2.86E-17 | 3.05E-15 |
| HBB | 0.032431951 | -4.946440371 | 2.90E-17 | 3.08E-15 |
| GNAI1 | 0.178321329 | -2.48744882 | 3.51E-17 | 3.72E-15 |
| CACNG4 | 5.296786168 | 2.405117268 | 3.80E-17 | 4.01E-15 |
| SPP1 | 4.92897291 | 2.301287052 | 3.86E-17 | 4.07E-15 |
| AGR3 | 5.093306281 | 2.348602475 | 3.90E-17 | 4.09E-15 |
| FRMD1 | 0.048405631 | -4.368681309 | 4.09E-17 | 4.28E-15 |
| DOCK11 | 0.197584408 | -2.339458992 | 4.14E-17 | 4.32E-15 |
| CLDN5 | 0.168142187 | -2.572246352 | 4.23E-17 | 4.40E-15 |
| ABI3BP | 0.225054885 | -2.151651217 | 4.66E-17 | 4.83E-15 |
| S100B | 0.145037533 | -2.785501804 | 4.73E-17 | 4.89E-15 |
| ABCA10 | 0.098165936 | -3.348633707 | 5.13E-17 | 5.29E-15 |
| PYCR1 | 5.141113343 | 2.362080819 | 5.28E-17 | 5.43E-15 |
| ASPM | 11.57679799 | 3.53316437 | 5.59E-17 | 5.72E-15 |
| MISP | 9.46413935 | 3.242471316 | 6.17E-17 | 6.28E-15 |
| SPRY2 | 0.184228599 | -2.440431061 | 6.58E-17 | 6.69E-15 |
| HSPB7 | 0.028849513 | -5.115309199 | 6.86E-17 | 6.94E-15 |
| CCL21 | 0.189049978 | -2.40316041 | 6.91E-17 | 6.98E-15 |
| SQLE | 4.980799647 | 2.31637738 | 8.00E-17 | 8.05E-15 |
| LHCGR | 0.04575887 | -4.449804763 | 8.10E-17 | 8.13E-15 |
| BHMT2 | 0.111551963 | -3.164212196 | 9.46E-17 | 9.40E-15 |
| NUSAP1 | 8.267296019 | 3.047415545 | 1.08E-16 | 1.06E-14 |
| NNAT | 0.085855658 | -3.541942975 | 1.09E-16 | 1.07E-14 |
| COL6A6 | 0.108505015 | -3.204166369 | 1.24E-16 | 1.22E-14 |
| HIF3A | 0.074509326 | -3.746435186 | 1.26E-16 | 1.23E-14 |
| ATOH8 | 0.102135506 | -3.291443611 | 1.39E-16 | 1.35E-14 |
| JPT1 | 4.635434038 | 2.212704432 | 1.57E-16 | 1.52E-14 |
| SLC22A12 | 0.033057799 | -4.918865505 | 1.62E-16 | 1.56E-14 |
| ANGPTL1 | 0.118493658 | -3.077118254 | 1.73E-16 | 1.67E-14 |
| APOLD1 | 0.237442838 | -2.074347857 | 1.80E-16 | 1.73E-14 |
| AADAC | 0.06221917 | -4.006497042 | 2.15E-16 | 2.05E-14 |
| SAA2 | 0.110698396 | -3.175293774 | 3.75E-16 | 3.56E-14 |
| PRAME | 30.53142587 | 4.932223062 | 3.79E-16 | 3.59E-14 |
| INSYN2B | 0.072263213 | -3.790594784 | 3.91E-16 | 3.70E-14 |
| APCDD1 | 0.224753168 | -2.153586642 | 4.07E-16 | 3.83E-14 |
| AL591686.1 | 0.041259295 | -4.599137012 | 4.23E-16 | 3.98E-14 |
| ZBTB16 | 0.122300473 | -3.031498108 | 4.30E-16 | 4.04E-14 |
| PLK1 | 10.71507879 | 3.421570554 | 4.87E-16 | 4.56E-14 |
| DEGS2 | 5.741054437 | 2.521315735 | 6.55E-16 | 6.04E-14 |
| ADH1C | 0.091732461 | -3.446423848 | 7.20E-16 | 6.63E-14 |
| MMP1 | 91.1044855 | 6.509450181 | 7.41E-16 | 6.80E-14 |
| LRRN4CL | 0.098533255 | -3.34324548 | 7.81E-16 | 7.14E-14 |
| ECRG4 | 0.131995323 | -2.921441289 | 8.60E-16 | 7.84E-14 |
| H1-2 | 4.833619491 | 2.273103907 | 8.83E-16 | 8.02E-14 |
| IFI6 | 7.410411405 | 2.889553639 | 9.23E-16 | 8.36E-14 |
| TSPAN7 | 0.177753181 | -2.492052719 | 1.15E-15 | 1.04E-13 |
| HMGB3 | 5.386939741 | 2.429465927 | 1.17E-15 | 1.05E-13 |
| AKR1C2 | 0.121194255 | -3.044606786 | 1.30E-15 | 1.16E-13 |
| AC007423.1 | 0.037013596 | -4.755800869 | 1.31E-15 | 1.16E-13 |
| KIF20A | 10.39598883 | 3.377955084 | 1.47E-15 | 1.30E-13 |
| MYZAP | 0.106275924 | -3.234113296 | 1.80E-15 | 1.59E-13 |
| CPM | 0.239137234 | -2.064089317 | 1.95E-15 | 1.72E-13 |
| RET | 7.452579017 | 2.897739767 | 2.09E-15 | 1.84E-13 |
| SCN4B | 0.169090002 | -2.564136738 | 2.16E-15 | 1.89E-13 |
| PRC1 | 6.270014277 | 2.648468728 | 2.20E-15 | 1.93E-13 |
| PKMYT1 | 15.92508301 | 3.993228988 | 2.28E-15 | 1.99E-13 |
| KIFC1 | 8.919863645 | 3.157021656 | 2.44E-15 | 2.13E-13 |
| TMEM132A | 4.55526851 | 2.187536096 | 2.48E-15 | 2.15E-13 |
| CYP2B7P | 14.98801717 | 3.905737631 | 2.61E-15 | 2.26E-13 |
| PFKFB1 | 0.08345222 | -3.582905758 | 2.89E-15 | 2.50E-13 |
| TNMD | 0.066841537 | -3.903111291 | 3.74E-15 | 3.22E-13 |
| KLF15 | 0.09541005 | -3.38971495 | 3.92E-15 | 3.36E-13 |
| ANK2 | 0.195593363 | -2.354070681 | 4.02E-15 | 3.43E-13 |
| TRHDE | 0.061437154 | -4.024744799 | 4.21E-15 | 3.59E-13 |
| ASPA | 0.08270835 | -3.59582321 | 4.45E-15 | 3.78E-13 |
| ABCA6 | 0.156948242 | -2.67163923 | 4.73E-15 | 3.99E-13 |
| LINC01070 | 0.033058516 | -4.918834239 | 5.10E-15 | 4.30E-13 |
| ANGPTL8 | 0.056748505 | -4.139273815 | 6.33E-15 | 5.27E-13 |
| GRM4 | 41.49281876 | 5.374789763 | 7.09E-15 | 5.89E-13 |
| HCAR2 | 0.092679651 | -3.431603575 | 8.15E-15 | 6.75E-13 |
| DSCAM-AS1 | 56.56233579 | 5.821769794 | 9.41E-15 | 7.76E-13 |
| TMEM100 | 0.105131029 | -3.249739558 | 9.85E-15 | 8.10E-13 |
| PLSCR4 | 0.230673064 | -2.116078549 | 1.16E-14 | 9.45E-13 |
| GRIA4 | 0.095458712 | -3.388979315 | 1.18E-14 | 9.59E-13 |
| FREM1 | 0.130455741 | -2.938367658 | 1.20E-14 | 9.75E-13 |
| SLC7A5 | 6.075425884 | 2.602985544 | 1.26E-14 | 1.02E-12 |
| CCL14 | 0.100080515 | -3.32076698 | 1.27E-14 | 1.02E-12 |
| CFB | 5.824247309 | 2.542071616 | 1.40E-14 | 1.13E-12 |
| KIF2C | 10.03182553 | 3.326512257 | 1.42E-14 | 1.14E-12 |
| FAT2 | 0.221944862 | -2.171726787 | 1.46E-14 | 1.16E-12 |
| CREB3L1 | 4.905939762 | 2.29452952 | 2.07E-14 | 1.64E-12 |
| PTGIS | 0.245162951 | -2.028187118 | 2.20E-14 | 1.74E-12 |
| PKHD1L1 | 0.078107015 | -3.678404069 | 2.32E-14 | 1.83E-12 |
| KIAA0408 | 0.044671425 | -4.484503912 | 2.57E-14 | 2.02E-12 |
| LMNB1 | 5.471013647 | 2.451808154 | 2.64E-14 | 2.07E-12 |
| PDE1C | 0.147342565 | -2.762753833 | 2.81E-14 | 2.19E-12 |
| CPA1 | 0.055577382 | -4.16935831 | 3.03E-14 | 2.35E-12 |
| S1PR1 | 0.243928976 | -2.035466949 | 3.15E-14 | 2.44E-12 |
| CLDN11 | 0.184217581 | -2.440517339 | 3.52E-14 | 2.71E-12 |
| ADAM33 | 0.153411983 | -2.704516923 | 3.82E-14 | 2.93E-12 |
| MELK | 12.44122191 | 3.637056282 | 3.87E-14 | 2.97E-12 |
| TSPAN1 | 5.452875437 | 2.447017199 | 4.35E-14 | 3.33E-12 |
| BUB1 | 9.827704232 | 3.29685444 | 4.47E-14 | 3.40E-12 |
| GABRE | 0.198382084 | -2.333646351 | 4.60E-14 | 3.49E-12 |
| PAFAH1B3 | 5.595272046 | 2.484208278 | 4.72E-14 | 3.57E-12 |
| SYNPO2 | 0.168956999 | -2.565271978 | 5.31E-14 | 3.99E-12 |
| PPP2R2C | 11.30672265 | 3.499108907 | 7.28E-14 | 5.43E-12 |
| NRN1 | 0.212211424 | -2.236425772 | 7.29E-14 | 5.43E-12 |
| MASP1 | 0.074572723 | -3.745208174 | 7.47E-14 | 5.54E-12 |
| CCNB1 | 5.94149674 | 2.57082641 | 7.53E-14 | 5.57E-12 |
| DIPK2B | 0.241329151 | -2.050925903 | 7.76E-14 | 5.73E-12 |
| CEP55 | 10.78747089 | 3.431284761 | 7.79E-14 | 5.74E-12 |
| RBMS3 | 0.22246272 | -2.168364504 | 7.97E-14 | 5.85E-12 |
| MMP13 | 78.49037591 | 6.294443864 | 8.75E-14 | 6.41E-12 |
| MEOX1 | 0.155883882 | -2.68145633 | 8.94E-14 | 6.54E-12 |
| PGM5 | 0.171159506 | -2.546586673 | 9.48E-14 | 6.90E-12 |
| ARHGAP20 | 0.116714777 | -3.098940863 | 9.52E-14 | 6.90E-12 |
| MEOX2 | 0.150388101 | -2.733237669 | 1.11E-13 | 8.05E-12 |
| CRHBP | 0.067323676 | -3.892742242 | 1.15E-13 | 8.30E-12 |
| FAM180B | 0.061469413 | -4.023987484 | 1.29E-13 | 9.29E-12 |
| CEBPA | 0.21502771 | -2.217405506 | 1.44E-13 | 1.04E-11 |
| ADGRD2 | 0.082880841 | -3.592817553 | 1.51E-13 | 1.08E-11 |
| LINC02587 | 0.071782822 | -3.800217548 | 1.74E-13 | 1.24E-11 |
| GYS2 | 0.033418304 | -4.903217653 | 1.82E-13 | 1.29E-11 |
| KCNB1 | 0.137659314 | -2.860825864 | 1.83E-13 | 1.29E-11 |
| CDCA5 | 8.544343182 | 3.094969594 | 1.87E-13 | 1.32E-11 |
| PIK3C2G | 0.161253758 | -2.632595312 | 2.08E-13 | 1.46E-11 |
| LINC00968 | 0.071919048 | -3.797482256 | 2.24E-13 | 1.56E-11 |
| LINC02511 | 0.058319661 | -4.099873848 | 2.25E-13 | 1.56E-11 |
| SLC29A4 | 0.174642109 | -2.51752664 | 2.34E-13 | 1.62E-11 |
| LINC01485 | 0.065121379 | -3.940724936 | 2.43E-13 | 1.68E-11 |
| NUF2 | 13.7003377 | 3.776139549 | 2.45E-13 | 1.69E-11 |
| IGSF10 | 0.137922038 | -2.858075097 | 2.52E-13 | 1.74E-11 |
| ABCC9 | 0.196070898 | -2.350552674 | 2.86E-13 | 1.95E-11 |
| DTL | 8.242328736 | 3.043052005 | 2.96E-13 | 2.02E-11 |
| LDB2 | 0.22537274 | -2.149615071 | 3.01E-13 | 2.05E-11 |
| CCN4 | 11.59517377 | 3.535452536 | 3.11E-13 | 2.11E-11 |
| KCNMB1 | 0.188115647 | -2.410308243 | 3.12E-13 | 2.11E-11 |
| CXCL10 | 12.17878559 | 3.606298377 | 3.47E-13 | 2.34E-11 |
| RELN | 0.151312498 | -2.724396944 | 3.59E-13 | 2.42E-11 |
| H2BC5 | 6.410335131 | 2.680399783 | 3.63E-13 | 2.45E-11 |
| ANGPT1 | 0.145796151 | -2.777975459 | 4.48E-13 | 2.99E-11 |
| VIT | 0.142187038 | -2.814138139 | 4.49E-13 | 2.99E-11 |
| JAM2 | 0.238868265 | -2.065712898 | 4.95E-13 | 3.28E-11 |
| PRRT4 | 0.08077864 | -3.629882336 | 4.98E-13 | 3.30E-11 |
| SPAG5 | 6.997396024 | 2.806818145 | 5.01E-13 | 3.31E-11 |
| CLDN19 | 0.089879638 | -3.475861873 | 5.02E-13 | 3.31E-11 |
| UHRF1 | 10.15456863 | 3.344057049 | 5.33E-13 | 3.50E-11 |
| KIF26B | 7.077078302 | 2.823153882 | 6.07E-13 | 3.99E-11 |
| FAM189A2 | 0.226992136 | -2.139285777 | 6.72E-13 | 4.40E-11 |
| AURKA | 8.667026483 | 3.115537113 | 6.91E-13 | 4.52E-11 |
| RIMS4 | 10.03831154 | 3.327444721 | 7.06E-13 | 4.61E-11 |
| PLPP4 | 32.22562848 | 5.01013659 | 7.10E-13 | 4.62E-11 |
| CACHD1 | 0.231630292 | -2.110104159 | 7.17E-13 | 4.66E-11 |
| CDC6 | 8.037733647 | 3.00678877 | 7.30E-13 | 4.74E-11 |
| FAM83D | 11.3501848 | 3.504643882 | 7.49E-13 | 4.85E-11 |
| ABCB5 | 0.084935052 | -3.557496128 | 7.60E-13 | 4.90E-11 |
| MAP1LC3C | 0.074191734 | -3.752597737 | 8.08E-13 | 5.20E-11 |
| MEDAG | 0.191195149 | -2.386882178 | 8.49E-13 | 5.46E-11 |
| ANGPTL5 | 0.057719599 | -4.114794924 | 8.61E-13 | 5.52E-11 |
| SMIM22 | 5.744020158 | 2.522060812 | 8.98E-13 | 5.75E-11 |
| CNTFR | 0.159047666 | -2.652468893 | 9.04E-13 | 5.78E-11 |
| AC134043.3 | 0.089051832 | -3.489210906 | 1.03E-12 | 6.55E-11 |
| MRPS30-DT | 8.16393267 | 3.029264284 | 1.04E-12 | 6.63E-11 |
| ASF1B | 8.333143965 | 3.058860905 | 1.09E-12 | 6.93E-11 |
| KLHL31 | 0.068638004 | -3.864848593 | 1.13E-12 | 7.18E-11 |
| EBF3 | 0.148352587 | -2.752898008 | 1.16E-12 | 7.36E-11 |
| CLDN8 | 0.236498554 | -2.080096735 | 1.19E-12 | 7.52E-11 |
| H3C10 | 19.62315847 | 4.294485366 | 1.30E-12 | 8.16E-11 |
| ISG15 | 11.55177082 | 3.53004212 | 1.30E-12 | 8.19E-11 |
| UBE2T | 9.301227392 | 3.217421107 | 1.34E-12 | 8.39E-11 |
| ZWINT | 5.982079534 | 2.580647092 | 1.39E-12 | 8.68E-11 |
| METRN | 5.209499783 | 2.381144851 | 1.40E-12 | 8.74E-11 |
| PTTG1 | 9.30846291 | 3.218542958 | 1.42E-12 | 8.83E-11 |
| GNG2 | 0.238118403 | -2.070248973 | 1.60E-12 | 9.94E-11 |
| FGF1 | 0.23782553 | -2.072024502 | 1.81E-12 | 1.11E-10 |
| ACADL | 0.099108659 | -3.334845085 | 2.16E-12 | 1.32E-10 |
| HOXB13 | 51.57372411 | 5.68856432 | 2.17E-12 | 1.32E-10 |
| ADH4 | 0.076765851 | -3.703391519 | 2.24E-12 | 1.36E-10 |
| H2BC21 | 4.025162055 | 2.009046868 | 2.31E-12 | 1.41E-10 |
| PREX2 | 0.201178853 | -2.313449432 | 2.57E-12 | 1.56E-10 |
| ISM1 | 0.219902538 | -2.18506384 | 2.63E-12 | 1.59E-10 |
| HPSE2 | 0.109434663 | -3.191858313 | 2.92E-12 | 1.76E-10 |
| CSF3 | 0.077135223 | -3.696466382 | 3.12E-12 | 1.86E-10 |
| FXYD1 | 0.081981853 | -3.608551588 | 3.35E-12 | 2.00E-10 |
| MOCS1 | 0.222675155 | -2.166987495 | 3.58E-12 | 2.12E-10 |
| LINC01614 | 47.06009428 | 5.556432306 | 3.64E-12 | 2.15E-10 |
| AC093425.1 | 0.046652127 | -4.421913319 | 3.73E-12 | 2.20E-10 |
| RBP7 | 0.215880461 | -2.211695421 | 4.17E-12 | 2.45E-10 |
| KCNE4 | 6.015233959 | 2.588620851 | 4.18E-12 | 2.46E-10 |
| FHL1 | 0.030555058 | -5.03244495 | 4.50E-12 | 2.64E-10 |
| MAPK8IP2 | 6.624571332 | 2.727827102 | 4.73E-12 | 2.76E-10 |
| CCNB2 | 8.252019946 | 3.044747308 | 5.00E-12 | 2.91E-10 |
| CEMIP | 9.110928986 | 3.187598164 | 5.46E-12 | 3.17E-10 |
| LINC01537 | 0.08244935 | -3.600348074 | 6.20E-12 | 3.57E-10 |
| LINC01028 | 0.05811193 | -4.10502183 | 6.49E-12 | 3.73E-10 |
| CXCL11 | 13.45336336 | 3.749894989 | 6.56E-12 | 3.76E-10 |
| MAGI2-AS3 | 0.216702837 | -2.206210057 | 6.96E-12 | 3.98E-10 |
| TACC3 | 5.059016648 | 2.338856987 | 7.35E-12 | 4.19E-10 |
| EDNRB | 0.219353409 | -2.188670965 | 7.87E-12 | 4.48E-10 |
| CDKN1C | 0.233640141 | -2.097639937 | 9.11E-12 | 5.11E-10 |
| C6 | 0.108262893 | -3.207389245 | 9.26E-12 | 5.19E-10 |
| KIF11 | 5.711494896 | 2.513868398 | 9.55E-12 | 5.34E-10 |
| BCAS1 | 5.431163743 | 2.44126136 | 9.84E-12 | 5.48E-10 |
| EPYC | 67.23769168 | 6.071198291 | 9.98E-12 | 5.54E-10 |
| PCLAF | 9.927713878 | 3.311461537 | 1.20E-11 | 6.58E-10 |
| ALDH1A2 | 0.141069139 | -2.825525681 | 1.23E-11 | 6.74E-10 |
| CDCA8 | 8.162027658 | 3.028927599 | 1.25E-11 | 6.84E-10 |
| SGK2 | 0.112061948 | -3.157631618 | 1.35E-11 | 7.34E-10 |
| PAQR4 | 5.96198114 | 2.575791812 | 1.38E-11 | 7.53E-10 |
| HJURP | 11.52332393 | 3.52648502 | 1.41E-11 | 7.63E-10 |
| HBA1 | 0.057813067 | -4.112460588 | 1.46E-11 | 7.87E-10 |
| PID1 | 0.179077274 | -2.481345834 | 1.48E-11 | 7.96E-10 |
| IQANK1 | 4.674748194 | 2.224888656 | 1.49E-11 | 8.02E-10 |
| RECQL4 | 6.955329855 | 2.798118936 | 1.50E-11 | 8.06E-10 |
| CILP2 | 11.02889182 | 3.463215931 | 1.56E-11 | 8.34E-10 |
| ARNT2 | 4.108201081 | 2.038506798 | 1.58E-11 | 8.44E-10 |
| MAGEA3 | 210.669449 | 7.718837302 | 1.67E-11 | 8.92E-10 |
| PLAAT5 | 0.220671122 | -2.180030253 | 1.68E-11 | 8.97E-10 |
| MATN3 | 7.193239665 | 2.846641673 | 1.69E-11 | 9.00E-10 |
| FAM89A | 0.176937231 | -2.498690445 | 1.74E-11 | 9.25E-10 |
| DLGAP5 | 9.880571872 | 3.304594545 | 1.75E-11 | 9.29E-10 |
| LILRB5 | 0.143753204 | -2.798333985 | 1.76E-11 | 9.31E-10 |
| NPR3 | 0.246854152 | -2.018269187 | 1.82E-11 | 9.60E-10 |
| KLHL29 | 0.1785539 | -2.485568453 | 1.88E-11 | 9.89E-10 |
| TROAP | 11.88017109 | 3.570483708 | 2.20E-11 | 1.15E-09 |
| SLC16A3 | 4.735772921 | 2.243599906 | 2.25E-11 | 1.18E-09 |
| AL391883.1 | 0.048453136 | -4.367266148 | 2.39E-11 | 1.25E-09 |
| CKS2 | 6.30375161 | 2.656210689 | 2.45E-11 | 1.28E-09 |
| NMUR1 | 0.090243429 | -3.470034309 | 3.52E-11 | 1.82E-09 |
| ANGPT4 | 0.088328694 | -3.500974006 | 3.60E-11 | 1.85E-09 |
| HOXC10 | 5.570082786 | 2.47769877 | 3.67E-11 | 1.89E-09 |
| EPB42 | 0.084540058 | -3.564221086 | 3.87E-11 | 1.98E-09 |
| IL6 | 0.143861021 | -2.797252347 | 4.00E-11 | 2.04E-09 |
| C1QTNF9 | 0.078699667 | -3.667498651 | 4.03E-11 | 2.05E-09 |
| CCNA2 | 7.295967557 | 2.867099314 | 4.12E-11 | 2.09E-09 |
| AC097713.1 | 0.071448378 | -3.806954933 | 4.24E-11 | 2.15E-09 |
| CD209 | 0.161373777 | -2.631521933 | 4.47E-11 | 2.26E-09 |
| CTXN1 | 7.32860143 | 2.873537905 | 4.70E-11 | 2.38E-09 |
| WASF3 | 0.221688787 | -2.173392295 | 4.78E-11 | 2.41E-09 |
| MFAP2 | 5.099607098 | 2.350386098 | 4.79E-11 | 2.42E-09 |
| STARD9 | 0.225502123 | -2.148787078 | 5.08E-11 | 2.55E-09 |
| SLC24A2 | 31.10186966 | 4.958929404 | 5.55E-11 | 2.78E-09 |
| AL049749.1 | 0.039892055 | -4.64775474 | 6.37E-11 | 3.18E-09 |
| GXYLT1P6 | 0.029111057 | -5.102288977 | 6.38E-11 | 3.19E-09 |
| AOX1 | 0.20405652 | -2.292959287 | 6.47E-11 | 3.22E-09 |
| KIF1A | 19.57851762 | 4.291199631 | 6.54E-11 | 3.25E-09 |
| FGD3 | 4.121985619 | 2.043339472 | 6.79E-11 | 3.37E-09 |
| ADRB2 | 0.141527719 | -2.820843457 | 6.97E-11 | 3.45E-09 |
| CSAG1 | 512.2907545 | 9.000819045 | 7.05E-11 | 3.48E-09 |
| PBK | 10.98944856 | 3.45804709 | 7.30E-11 | 3.61E-09 |
| SYN2 | 0.147322318 | -2.762952095 | 7.43E-11 | 3.66E-09 |
| AP001528.1 | 0.109551622 | -3.190317244 | 7.59E-11 | 3.73E-09 |
| SUSD3 | 4.286701254 | 2.099867877 | 7.91E-11 | 3.88E-09 |
| RHPN1 | 4.908107433 | 2.295166828 | 7.98E-11 | 3.91E-09 |
| MAGEA1 | 259.3475705 | 8.018743044 | 8.22E-11 | 4.02E-09 |
| TYRO3 | 0.2216452 | -2.173675977 | 8.25E-11 | 4.02E-09 |
| EXO1 | 10.87005772 | 3.442287696 | 8.59E-11 | 4.17E-09 |
| GLP2R | 0.093954019 | -3.411901308 | 8.72E-11 | 4.23E-09 |
| NCAPG | 8.367295148 | 3.064761326 | 8.93E-11 | 4.32E-09 |
| RHOXF1-AS1 | 0.111511389 | -3.164737033 | 9.30E-11 | 4.49E-09 |
| AC073850.1 | 0.071191083 | -3.812159639 | 9.33E-11 | 4.50E-09 |
| FAM111B | 7.015487542 | 2.810543368 | 9.64E-11 | 4.63E-09 |
| SBK1 | 4.842540173 | 2.275764017 | 1.07E-10 | 5.12E-09 |
| CACNA1H | 5.011777893 | 2.32532248 | 1.11E-10 | 5.28E-09 |
| TACR1 | 0.165528252 | -2.594850619 | 1.12E-10 | 5.32E-09 |
| KIF23 | 6.206361447 | 2.633747719 | 1.54E-10 | 7.28E-09 |
| KIF18B | 11.826165 | 3.563910406 | 1.55E-10 | 7.34E-09 |
| ADAMTS9-AS2 | 0.117213039 | -3.092795031 | 1.63E-10 | 7.68E-09 |
| EPN3 | 4.578943196 | 2.195014668 | 1.71E-10 | 8.05E-09 |
| AC007906.2 | 0.146468745 | -2.771335253 | 1.76E-10 | 8.26E-09 |
| MYMX | 0.0901851 | -3.470967091 | 1.81E-10 | 8.49E-09 |
| ACTA2-AS1 | 0.172113391 | -2.538568745 | 1.89E-10 | 8.83E-09 |
| CLCA4 | 0.103031123 | -3.278847895 | 1.91E-10 | 8.90E-09 |
| NDC80 | 9.221084036 | 3.204936365 | 1.91E-10 | 8.92E-09 |
| C1QTNF6 | 4.651755246 | 2.217775191 | 1.92E-10 | 8.94E-09 |
| BUB1B | 7.761038613 | 2.956249732 | 2.00E-10 | 9.28E-09 |
| AC091939.1 | 0.056374272 | -4.148819293 | 2.08E-10 | 9.65E-09 |
| DTX1 | 0.181841308 | -2.459248126 | 2.13E-10 | 9.86E-09 |
| CBX2 | 6.47182062 | 2.694171621 | 2.17E-10 | 1.00E-08 |
| LRRC3B | 0.084918207 | -3.557782281 | 2.29E-10 | 1.06E-08 |
| ABCC13 | 45.89061328 | 5.520127182 | 2.31E-10 | 1.07E-08 |
| KY | 0.100816327 | -3.310198799 | 2.44E-10 | 1.12E-08 |
| AL445426.1 | 0.071961546 | -3.796630004 | 2.46E-10 | 1.13E-08 |
| INMT | 0.171890118 | -2.540441492 | 2.58E-10 | 1.18E-08 |
| KIF14 | 11.42770793 | 3.514464164 | 2.68E-10 | 1.22E-08 |
| AURKB | 10.50975882 | 3.393657657 | 2.75E-10 | 1.25E-08 |
| PRR15 | 5.198448883 | 2.378081215 | 2.91E-10 | 1.32E-08 |
| BICDL1 | 6.62084382 | 2.727015099 | 3.26E-10 | 1.47E-08 |
| SLC16A6 | 5.514779018 | 2.463303077 | 3.37E-10 | 1.52E-08 |
| CAPS | 4.515001608 | 2.174726501 | 3.46E-10 | 1.56E-08 |
| SERTM1 | 0.069001885 | -3.857220422 | 3.85E-10 | 1.73E-08 |
| LINC02660 | 0.068668596 | -3.864205723 | 4.41E-10 | 1.96E-08 |
| HPN | 4.644068891 | 2.215389373 | 4.68E-10 | 2.08E-08 |
| PAK3 | 0.170867973 | -2.549046091 | 4.99E-10 | 2.20E-08 |
| DIO1 | 11.41479907 | 3.51283356 | 5.08E-10 | 2.24E-08 |
| CA9 | 27.93682739 | 4.804096287 | 5.19E-10 | 2.28E-08 |
| PAK5 | 0.111487076 | -3.165051614 | 5.24E-10 | 2.30E-08 |
| NR3C2 | 0.224763326 | -2.153521441 | 5.24E-10 | 2.30E-08 |
| AC016924.1 | 0.068228644 | -3.873478648 | 5.68E-10 | 2.48E-08 |
| URAD | 0.05574581 | -4.164992813 | 5.71E-10 | 2.49E-08 |
| TSLP | 0.105719806 | -3.241682416 | 5.99E-10 | 2.60E-08 |
| NGF-AS1 | 0.060664809 | -4.042996324 | 6.13E-10 | 2.66E-08 |
| JPH2 | 0.189325122 | -2.401062238 | 6.26E-10 | 2.71E-08 |
| HSD11B1 | 0.147891069 | -2.75739316 | 6.30E-10 | 2.73E-08 |
| DEPDC1 | 9.571926803 | 3.258809365 | 6.39E-10 | 2.76E-08 |
| CBLN2 | 66.58702629 | 6.057169207 | 6.51E-10 | 2.80E-08 |
| PYDC1 | 21.79091681 | 4.445654991 | 6.94E-10 | 2.98E-08 |
| OVCH2 | 0.108835484 | -3.199779093 | 7.25E-10 | 3.11E-08 |
| ADCYAP1R1 | 0.159838855 | -2.645309944 | 7.46E-10 | 3.18E-08 |
| HAGHL | 11.06323619 | 3.467701556 | 7.95E-10 | 3.38E-08 |
| E2F1 | 6.257478739 | 2.645581484 | 8.04E-10 | 3.42E-08 |
| PARAL1 | 0.049647073 | -4.332147526 | 9.67E-10 | 4.08E-08 |
| SCN4A | 0.082368736 | -3.601759338 | 9.87E-10 | 4.15E-08 |
| SHE | 0.227481959 | -2.136175959 | 1.06E-09 | 4.46E-08 |
| FGFR3 | 5.251517647 | 2.39273441 | 1.09E-09 | 4.55E-08 |
| CKAP2L | 10.09633412 | 3.335759654 | 1.09E-09 | 4.57E-08 |
| GBP5 | 5.80810618 | 2.538067828 | 1.15E-09 | 4.81E-08 |
| AP001360.1 | 0.082915033 | -3.592222499 | 1.16E-09 | 4.84E-08 |
| CDKN2A | 6.809672637 | 2.767585445 | 1.25E-09 | 5.21E-08 |
| MCM10 | 8.821953591 | 3.141098171 | 1.29E-09 | 5.34E-08 |
| AC025470.2 | 0.084499982 | -3.564905155 | 1.29E-09 | 5.36E-08 |
| MAGEA6 | 130.878347 | 7.032082622 | 1.32E-09 | 5.46E-08 |
| NIPSNAP3B | 0.134885108 | -2.890197017 | 1.35E-09 | 5.60E-08 |
| CLEC3A | 51.64186431 | 5.690469179 | 1.36E-09 | 5.62E-08 |
| B3GALT1 | 0.11375778 | -3.135962878 | 1.45E-09 | 5.98E-08 |
| HNRNPA1P57 | 57.39291559 | 5.842800761 | 1.47E-09 | 6.05E-08 |
| STAB2 | 0.12623276 | -2.985841727 | 1.48E-09 | 6.07E-08 |
| CDCA3 | 8.339896858 | 3.060029542 | 1.55E-09 | 6.33E-08 |
| RAMP1 | 6.097806418 | 2.608290351 | 1.63E-09 | 6.66E-08 |
| RSPO3 | 0.184342945 | -2.439535893 | 1.63E-09 | 6.66E-08 |
| SLC22A3 | 0.137725414 | -2.860133294 | 1.68E-09 | 6.83E-08 |
| HMMR | 8.230951 | 3.041059129 | 1.69E-09 | 6.84E-08 |
| NRG1 | 0.229419535 | -2.123939854 | 1.74E-09 | 7.06E-08 |
| AC036108.2 | 0.10029966 | -3.317611376 | 1.76E-09 | 7.11E-08 |
| LRG1 | 4.237315625 | 2.083150595 | 1.80E-09 | 7.24E-08 |
| LINC02202 | 0.109435204 | -3.191851185 | 1.82E-09 | 7.32E-08 |
| AKR7A3 | 5.867701738 | 2.552795539 | 1.83E-09 | 7.34E-08 |
| RERGL | 0.139710296 | -2.839489754 | 1.86E-09 | 7.43E-08 |
| AC104407.1 | 0.093761602 | -3.414858975 | 1.87E-09 | 7.48E-08 |
| PRRG3 | 0.129018431 | -2.954350919 | 1.87E-09 | 7.48E-08 |
| COX7A1 | 0.174570821 | -2.518115658 | 1.90E-09 | 7.59E-08 |
| ADGRD1 | 0.169719353 | -2.55877701 | 1.92E-09 | 7.63E-08 |
| CFL2 | 0.208495551 | -2.261911495 | 2.00E-09 | 7.93E-08 |
| MEX3A | 4.180719168 | 2.063751136 | 2.01E-09 | 7.97E-08 |
| MAGEA12 | 347.541035 | 8.44103952 | 2.04E-09 | 8.06E-08 |
| TBX15 | 0.205359998 | -2.283772911 | 2.24E-09 | 8.85E-08 |
| DNAJC12 | 4.185001764 | 2.065228231 | 2.42E-09 | 9.54E-08 |
| BBOX1 | 0.246614601 | -2.019669879 | 2.56E-09 | 1.01E-07 |
| GGTA1P | 0.239055984 | -2.064579578 | 2.79E-09 | 1.10E-07 |
| CST4 | 361.2699956 | 8.49693363 | 2.92E-09 | 1.14E-07 |
| WT1 | 23.52319779 | 4.556012291 | 3.44E-09 | 1.33E-07 |
| FOXJ1 | 22.63475462 | 4.500467761 | 3.49E-09 | 1.35E-07 |
| PRR36 | 4.622130857 | 2.208558105 | 3.59E-09 | 1.39E-07 |
| ESPL1 | 6.659415866 | 2.735395636 | 3.67E-09 | 1.42E-07 |
| AC084064.1 | 0.067684667 | -3.885027148 | 3.86E-09 | 1.48E-07 |
| SLC7A3 | 0.122107658 | -3.033774408 | 3.90E-09 | 1.50E-07 |
| NWD2 | 0.080443147 | -3.63588666 | 4.28E-09 | 1.63E-07 |
| NPY2R | 0.087056247 | -3.521908362 | 4.50E-09 | 1.72E-07 |
| AL845321.1 | 0.089257832 | -3.485877424 | 4.64E-09 | 1.77E-07 |
| CDH20 | 0.102569177 | -3.285330845 | 5.20E-09 | 1.97E-07 |
| SEL1L2 | 0.078277155 | -3.675264862 | 5.31E-09 | 2.01E-07 |
| PGM5-AS1 | 0.086233456 | -3.535608483 | 5.38E-09 | 2.03E-07 |
| TTK | 7.335811533 | 2.874956575 | 5.38E-09 | 2.03E-07 |
| ANKRD29 | 0.216035474 | -2.210659864 | 5.51E-09 | 2.07E-07 |
| CENPE | 6.11814614 | 2.613094568 | 5.67E-09 | 2.13E-07 |
| DACT2 | 0.125899424 | -2.989656415 | 5.76E-09 | 2.16E-07 |
| LAMP5 | 7.784157303 | 2.960540864 | 6.17E-09 | 2.30E-07 |
| AC084759.3 | 0.098133022 | -3.349117504 | 6.33E-09 | 2.36E-07 |
| AL356489.2 | 0.105414457 | -3.245855358 | 6.37E-09 | 2.37E-07 |
| NR4A3 | 0.233668811 | -2.09746291 | 6.38E-09 | 2.37E-07 |
| CSRNP3 | 0.196007897 | -2.351016316 | 6.39E-09 | 2.37E-07 |
| EPHB1 | 0.215560617 | -2.213834472 | 6.55E-09 | 2.43E-07 |
| AC003986.2 | 0.102251764 | -3.289802366 | 6.64E-09 | 2.46E-07 |
| TMOD1 | 0.142781573 | -2.808118296 | 6.68E-09 | 2.47E-07 |
| TLX1 | 59.57988736 | 5.896753491 | 6.82E-09 | 2.51E-07 |
| RNF186 | 0.121168662 | -3.044911477 | 6.87E-09 | 2.53E-07 |
| CNN2P3 | 0.029031452 | -5.106239465 | 6.97E-09 | 2.56E-07 |
| GPRASP1 | 0.241306193 | -2.051063155 | 7.11E-09 | 2.61E-07 |
| AP005264.7 | 0.078856866 | -3.664619823 | 7.21E-09 | 2.65E-07 |
| UBE2S | 5.764153737 | 2.527108814 | 7.22E-09 | 2.65E-07 |
| CREB5 | 0.241090641 | -2.052352447 | 7.24E-09 | 2.65E-07 |
| SOX11 | 8.294095044 | 3.052084579 | 7.40E-09 | 2.70E-07 |
| LINGO1 | 5.656994119 | 2.500035671 | 7.80E-09 | 2.84E-07 |
| HSH2D | 6.334763375 | 2.663290731 | 7.92E-09 | 2.88E-07 |
| PPP1R14A | 0.199421797 | -2.326104987 | 8.01E-09 | 2.90E-07 |
| PKDCC | 0.242549516 | -2.043648793 | 8.08E-09 | 2.92E-07 |
| LINC00377 | 0.09533698 | -3.390820264 | 8.08E-09 | 2.92E-07 |
| CALB2 | 0.23732731 | -2.075049967 | 8.46E-09 | 3.05E-07 |
| PGM5P3-AS1 | 0.074639605 | -3.743914837 | 8.83E-09 | 3.17E-07 |
| GTSE1 | 8.223969361 | 3.03983489 | 8.86E-09 | 3.18E-07 |
| HOXA5 | 0.194500089 | -2.362157277 | 9.64E-09 | 3.44E-07 |
| SLC17A7 | 0.125689956 | -2.992058724 | 1.04E-08 | 3.69E-07 |
| SCN3A | 0.142153446 | -2.814479019 | 1.04E-08 | 3.69E-07 |
| HOXC11 | 11.88507157 | 3.571078686 | 1.05E-08 | 3.70E-07 |
| NCAPH | 6.894625743 | 2.785472241 | 1.06E-08 | 3.74E-07 |
| AC002398.2 | 0.072352553 | -3.788812274 | 1.09E-08 | 3.83E-07 |
| AC104212.2 | 0.073328452 | -3.769483116 | 1.09E-08 | 3.84E-07 |
| CENPU | 5.178525581 | 2.372541395 | 1.18E-08 | 4.13E-07 |
| NEURL1 | 5.638292839 | 2.49525841 | 1.18E-08 | 4.13E-07 |
| SPC24 | 8.876911429 | 3.150057803 | 1.23E-08 | 4.31E-07 |
| CAVIN2-AS1 | 0.112982296 | -3.14583137 | 1.25E-08 | 4.35E-07 |
| C19orf33 | 6.911574173 | 2.789014335 | 1.25E-08 | 4.37E-07 |
| TRIP13 | 5.947023426 | 2.572167759 | 1.27E-08 | 4.43E-07 |
| CHST9 | 0.197144716 | -2.342673052 | 1.29E-08 | 4.49E-07 |
| CEACAM6 | 19.43735997 | 4.280760377 | 1.39E-08 | 4.82E-07 |
| SCN2B | 0.139346924 | -2.843246936 | 1.40E-08 | 4.85E-07 |
| NAALAD2 | 0.133635324 | -2.90362669 | 1.46E-08 | 5.04E-07 |
| LINC02082 | 0.069802343 | -3.840580727 | 1.47E-08 | 5.06E-07 |
| CCDC178 | 0.124472213 | -3.006104384 | 1.48E-08 | 5.09E-07 |
| GIPC2 | 0.159105788 | -2.651941773 | 1.49E-08 | 5.12E-07 |
| CLGN | 6.223169756 | 2.637649601 | 1.50E-08 | 5.17E-07 |
| TPRN | 4.028553389 | 2.010261875 | 1.54E-08 | 5.29E-07 |
| AL160412.1 | 0.079191197 | -3.658516126 | 1.59E-08 | 5.44E-07 |
| SPHKAP | 0.13879301 | -2.848993182 | 1.59E-08 | 5.45E-07 |
| PEAR1 | 0.243029183 | -2.040798534 | 1.67E-08 | 5.71E-07 |
| KNL1 | 7.086298494 | 2.825032237 | 1.78E-08 | 6.05E-07 |
| FSIP1 | 6.486836542 | 2.697515086 | 1.93E-08 | 6.54E-07 |
| HOXC12 | 17.16445114 | 4.10135182 | 1.97E-08 | 6.68E-07 |
| CYP2T1P | 6.92132702 | 2.791048671 | 1.98E-08 | 6.68E-07 |
| PIGR | 0.205390464 | -2.283558894 | 2.07E-08 | 6.99E-07 |
| CMA1 | 0.131864196 | -2.922875205 | 2.08E-08 | 7.00E-07 |
| ALX4 | 0.199232954 | -2.3274718 | 2.12E-08 | 7.12E-07 |
| LUARIS | 0.111292902 | -3.167566516 | 2.24E-08 | 7.53E-07 |
| SLC66A1L | 0.13509453 | -2.887958838 | 2.27E-08 | 7.62E-07 |
| CLEC4G | 0.113618273 | -3.137733216 | 2.29E-08 | 7.65E-07 |
| AC092155.1 | 0.07387146 | -3.758839096 | 2.32E-08 | 7.75E-07 |
| H2BC12 | 4.27918496 | 2.097336038 | 2.35E-08 | 7.82E-07 |
| EZH2 | 4.973304747 | 2.314204837 | 2.39E-08 | 7.96E-07 |
| CORO2B | 0.194577561 | -2.361582751 | 2.76E-08 | 9.09E-07 |
| DENND2A | 0.219310205 | -2.188955147 | 2.91E-08 | 9.54E-07 |
| AP000892.4 | 0.221776347 | -2.172822587 | 3.08E-08 | 1.01E-06 |
| TINCR | 6.017029262 | 2.589051373 | 3.10E-08 | 1.01E-06 |
| LRTM2 | 47.94348991 | 5.583263025 | 3.15E-08 | 1.02E-06 |
| RMI2 | 6.328397505 | 2.661840223 | 3.20E-08 | 1.04E-06 |
| MYT1 | 22.47339648 | 4.490146275 | 3.27E-08 | 1.06E-06 |
| C1QTNF7 | 0.177423732 | -2.494729101 | 3.31E-08 | 1.07E-06 |
| IGF1 | 0.176040022 | -2.506024638 | 3.33E-08 | 1.08E-06 |
| GLRA4 | 0.103067087 | -3.278344395 | 3.37E-08 | 1.09E-06 |
| LINC00445 | 0.088390237 | -3.49996916 | 3.47E-08 | 1.12E-06 |
| SYT1 | 7.711764063 | 2.947060914 | 3.49E-08 | 1.13E-06 |
| MAPK10 | 0.244330768 | -2.033092547 | 3.53E-08 | 1.14E-06 |
| LINC02607 | 0.154146969 | -2.697621574 | 3.54E-08 | 1.14E-06 |
| AF001548.3 | 0.091980541 | -3.442527505 | 3.54E-08 | 1.14E-06 |
| DNASE1L3 | 0.145459759 | -2.781308005 | 3.61E-08 | 1.16E-06 |
| SEMA3D | 0.236301705 | -2.081298057 | 3.61E-08 | 1.16E-06 |
| C2orf88 | 0.215614995 | -2.213470579 | 3.90E-08 | 1.24E-06 |
| HS6ST3 | 7.202917919 | 2.848581464 | 4.18E-08 | 1.32E-06 |
| XPNPEP2 | 0.141866075 | -2.817398465 | 4.25E-08 | 1.34E-06 |
| F10 | 0.1635289 | -2.612382474 | 4.30E-08 | 1.36E-06 |
| DMGDH | 0.144966853 | -2.786205033 | 4.35E-08 | 1.37E-06 |
| GINS1 | 5.642096575 | 2.496231361 | 4.42E-08 | 1.39E-06 |
| AL353693.1 | 0.103198451 | -3.27650678 | 4.44E-08 | 1.40E-06 |
| SKA1 | 10.39418717 | 3.377705038 | 4.47E-08 | 1.40E-06 |
| AP000445.1 | 0.069262437 | -3.851783039 | 4.57E-08 | 1.43E-06 |
| GPBAR1 | 0.127344007 | -2.973197031 | 4.61E-08 | 1.45E-06 |
| GNAL | 0.23928967 | -2.063169977 | 4.81E-08 | 1.50E-06 |
| HS3ST4 | 0.15062818 | -2.730936397 | 4.89E-08 | 1.53E-06 |
| AOC4P | 0.103838823 | -3.267582159 | 4.93E-08 | 1.54E-06 |
| AC002066.1 | 0.098151076 | -3.34885211 | 4.95E-08 | 1.54E-06 |
| TWIST2 | 0.20501918 | -2.286169211 | 5.13E-08 | 1.59E-06 |
| FBN2 | 8.397921934 | 3.070032377 | 5.13E-08 | 1.59E-06 |
| MAB21L1 | 0.153570013 | -2.70303156 | 5.18E-08 | 1.61E-06 |
| CYP26B1 | 0.229290216 | -2.124753299 | 5.28E-08 | 1.64E-06 |
| LINC00645 | 0.095890125 | -3.382473945 | 5.83E-08 | 1.79E-06 |
| AC010969.1 | 0.105913928 | -3.239035775 | 6.14E-08 | 1.88E-06 |
| SKA3 | 8.4843102 | 3.084797369 | 6.30E-08 | 1.92E-06 |
| MAPK15 | 6.461199442 | 2.691802008 | 6.83E-08 | 2.08E-06 |
| GDF15 | 5.189484236 | 2.375591161 | 7.01E-08 | 2.13E-06 |
| FBXL16 | 4.061123052 | 2.021878742 | 7.10E-08 | 2.16E-06 |
| CEACAM5 | 20.13226207 | 4.331437378 | 7.22E-08 | 2.19E-06 |
| PTN | 0.195704783 | -2.353249079 | 7.39E-08 | 2.24E-06 |
| BMX | 0.153083745 | -2.707606998 | 7.54E-08 | 2.28E-06 |
| SLC1A1 | 4.825991826 | 2.270825472 | 7.77E-08 | 2.34E-06 |
| CCDC78 | 11.59822085 | 3.535831609 | 7.88E-08 | 2.37E-06 |
| RAB26 | 8.691126195 | 3.119543133 | 7.94E-08 | 2.39E-06 |
| KCNK15 | 5.645212746 | 2.497027952 | 7.98E-08 | 2.40E-06 |
| ADAM8 | 4.857642925 | 2.280256444 | 8.34E-08 | 2.50E-06 |
| SLC25A24P1 | 19.71931145 | 4.301537272 | 8.60E-08 | 2.58E-06 |
| PGM5P4-AS1 | 0.081872026 | -3.610485586 | 8.65E-08 | 2.59E-06 |
| EBF2 | 0.206854588 | -2.273311139 | 9.12E-08 | 2.72E-06 |
| OASL | 5.152458512 | 2.365260983 | 9.37E-08 | 2.79E-06 |
| HOXC13 | 7.838069362 | 2.97049834 | 9.64E-08 | 2.86E-06 |
| INSYN2A | 12.92976797 | 3.69262448 | 9.66E-08 | 2.86E-06 |
| EGFEM1P | 0.105794665 | -3.240661218 | 1.01E-07 | 2.98E-06 |
| ACTL8 | 259.1231851 | 8.017494296 | 1.06E-07 | 3.12E-06 |
| PKD1L2 | 0.156923239 | -2.671869077 | 1.07E-07 | 3.15E-06 |
| CDT1 | 5.586384222 | 2.481914803 | 1.07E-07 | 3.15E-06 |
| GPR146 | 0.14598778 | -2.776080487 | 1.10E-07 | 3.22E-06 |
| SCUBE3 | 7.394093799 | 2.886373345 | 1.10E-07 | 3.22E-06 |
| ALAS2 | 0.104055206 | -3.264578948 | 1.12E-07 | 3.28E-06 |
| AVPR2 | 0.147764409 | -2.758629273 | 1.13E-07 | 3.32E-06 |
| LGI4 | 0.190942783 | -2.388787704 | 1.22E-07 | 3.56E-06 |
| GRIN2B | 0.142958342 | -2.806333288 | 1.23E-07 | 3.59E-06 |
| ADAMTS9-AS1 | 0.162354719 | -2.622778774 | 1.25E-07 | 3.65E-06 |
| CADM3 | 0.207379764 | -2.269652968 | 1.26E-07 | 3.67E-06 |
| MAD2L1 | 4.524553463 | 2.177775417 | 1.27E-07 | 3.68E-06 |
| PROX1 | 0.214339575 | -2.222029845 | 1.27E-07 | 3.70E-06 |
| CENPA | 9.678021382 | 3.274712127 | 1.38E-07 | 3.97E-06 |
| ASCL1 | 35.42772697 | 5.146807 | 1.45E-07 | 4.18E-06 |
| CDC45 | 7.481907572 | 2.903406144 | 1.48E-07 | 4.25E-06 |
| ARHGAP11A | 4.670493237 | 2.223574917 | 1.52E-07 | 4.35E-06 |
| AC004490.1 | 0.10895135 | -3.198244023 | 1.52E-07 | 4.36E-06 |
| RNU5B-3P | 0.077882764 | -3.682552103 | 1.54E-07 | 4.42E-06 |
| BCAS4 | 4.065537716 | 2.023446179 | 1.69E-07 | 4.82E-06 |
| CNTNAP3B | 0.150066678 | -2.736324426 | 1.71E-07 | 4.86E-06 |
| FHL5 | 0.161196077 | -2.633111459 | 1.75E-07 | 4.96E-06 |
| SMIM10L2A | 0.224119948 | -2.157657031 | 1.75E-07 | 4.96E-06 |
| CELSR3 | 5.621702164 | 2.491007022 | 1.82E-07 | 5.15E-06 |
| PRG4 | 0.086117205 | -3.537554699 | 1.86E-07 | 5.26E-06 |
| MRGPRX2 | 0.089315161 | -3.484951092 | 1.88E-07 | 5.30E-06 |
| TCEAL5 | 0.14603776 | -2.775586652 | 1.89E-07 | 5.32E-06 |
| TMEM220 | 0.211894536 | -2.238581706 | 2.00E-07 | 5.61E-06 |
| TMEM252 | 0.107353915 | -3.219553285 | 2.07E-07 | 5.81E-06 |
| IGHG3 | 5.89558955 | 2.559636088 | 2.11E-07 | 5.92E-06 |
| NSG1 | 0.213693455 | -2.226385371 | 2.15E-07 | 6.02E-06 |
| SAPCD2 | 4.790343329 | 2.260129059 | 2.18E-07 | 6.10E-06 |
| ATP1A2 | 0.043471795 | -4.523776508 | 2.19E-07 | 6.11E-06 |
| ESM1 | 7.779226332 | 2.959626682 | 2.23E-07 | 6.21E-06 |
| MAB21L4 | 11.42253342 | 3.513810758 | 2.27E-07 | 6.30E-06 |
| ABCB1 | 0.24497235 | -2.029309175 | 2.28E-07 | 6.34E-06 |
| AP005264.1 | 0.10360128 | -3.270886272 | 2.28E-07 | 6.34E-06 |
| KL | 0.178850242 | -2.483176023 | 2.29E-07 | 6.35E-06 |
| ITPRIPL1 | 0.195035433 | -2.358191843 | 2.30E-07 | 6.37E-06 |
| CDKN3 | 7.517735532 | 2.910298163 | 2.39E-07 | 6.60E-06 |
| MRGPRF | 0.243733158 | -2.036625564 | 2.45E-07 | 6.77E-06 |
| PARD6B | 4.547814915 | 2.185173541 | 2.49E-07 | 6.86E-06 |
| TYMS | 4.056859866 | 2.020363469 | 2.50E-07 | 6.88E-06 |
| SPTSSB | 4.287208389 | 2.100038544 | 2.58E-07 | 7.08E-06 |
| PLCZ1 | 0.125376975 | -2.995655664 | 2.66E-07 | 7.28E-06 |
| GPLD1 | 0.155111851 | -2.688619182 | 2.68E-07 | 7.34E-06 |
| DPP6 | 0.157528623 | -2.6663141 | 2.73E-07 | 7.44E-06 |
| COL22A1 | 8.568870314 | 3.099105018 | 2.74E-07 | 7.45E-06 |
| GPRIN1 | 7.39157302 | 2.885881421 | 2.77E-07 | 7.51E-06 |
| MS4A15 | 18.25507661 | 4.190225818 | 2.83E-07 | 7.68E-06 |
| NELL2 | 9.596130963 | 3.262452847 | 2.85E-07 | 7.72E-06 |
| AC063919.1 | 0.071569454 | -3.804512219 | 2.86E-07 | 7.74E-06 |
| TFF3 | 6.005104304 | 2.586189305 | 2.88E-07 | 7.79E-06 |
| AC044810.2 | 0.130209621 | -2.941092049 | 2.91E-07 | 7.86E-06 |
| CDCA2 | 7.66871782 | 2.938985385 | 3.03E-07 | 8.18E-06 |
| PGM5P4 | 0.100739489 | -3.311298771 | 3.07E-07 | 8.28E-06 |
| HORMAD1 | 17.61501165 | 4.138733524 | 3.09E-07 | 8.31E-06 |
| TFF1 | 7.347830303 | 2.877318308 | 3.13E-07 | 8.41E-06 |
| SCN2A | 0.202570713 | -2.303502485 | 3.16E-07 | 8.48E-06 |
| NKX2-2 | 51.50314244 | 5.686588555 | 3.19E-07 | 8.55E-06 |
| TONSL | 4.323559891 | 2.112219674 | 3.24E-07 | 8.67E-06 |
| TDO2 | 13.54964877 | 3.76018355 | 3.39E-07 | 9.04E-06 |
| PDE11A | 0.179411256 | -2.47865769 | 3.41E-07 | 9.10E-06 |
| PPIAP39 | 0.14920271 | -2.744654358 | 3.55E-07 | 9.46E-06 |
| AC110619.1 | 8.788980142 | 3.135695767 | 3.59E-07 | 9.53E-06 |
| RXRG | 0.121674191 | -3.038904913 | 3.75E-07 | 9.93E-06 |
| C5orf66-AS1 | 66.95511084 | 6.065122279 | 3.82E-07 | 1.01E-05 |
| GNG13 | 48.82102739 | 5.609430751 | 3.87E-07 | 1.02E-05 |
| DMRT2 | 0.142513546 | -2.810829043 | 3.90E-07 | 1.03E-05 |
| OLR1 | 4.694422929 | 2.230947823 | 4.07E-07 | 1.07E-05 |
| CADM3-AS1 | 0.157008819 | -2.671082504 | 4.07E-07 | 1.07E-05 |
| EDN3 | 0.191506197 | -2.384537018 | 4.12E-07 | 1.08E-05 |
| LINC02613 | 0.212681133 | -2.23323604 | 4.36E-07 | 1.14E-05 |
| LGR4-AS1 | 0.11989763 | -3.06012495 | 4.43E-07 | 1.16E-05 |
| RYR3 | 0.205795794 | -2.280714597 | 4.44E-07 | 1.16E-05 |
| COL25A1 | 0.178691587 | -2.484456386 | 4.48E-07 | 1.17E-05 |
| PRAC2 | 88.11877344 | 6.461377509 | 4.66E-07 | 1.21E-05 |
| AC024909.1 | 0.135488524 | -2.883757436 | 4.69E-07 | 1.22E-05 |
| AC012349.1 | 0.096690222 | -3.370486188 | 4.70E-07 | 1.22E-05 |
| TMEM255A | 0.210434119 | -2.248559458 | 4.74E-07 | 1.23E-05 |
| AC104211.2 | 0.095689476 | -3.385495918 | 4.75E-07 | 1.23E-05 |
| AC084866.1 | 0.065363193 | -3.935377722 | 4.81E-07 | 1.24E-05 |
| HES6 | 7.350440499 | 2.877830711 | 5.19E-07 | 1.33E-05 |
| LINC02224 | 24.07727068 | 4.589599957 | 5.24E-07 | 1.34E-05 |
| CLEC5A | 9.641579001 | 3.269269436 | 5.29E-07 | 1.35E-05 |
| CNTD2 | 8.127713248 | 3.022849504 | 5.32E-07 | 1.36E-05 |
| SCTR | 0.119132677 | -3.069358911 | 5.34E-07 | 1.36E-05 |
| CDC25C | 10.47268316 | 3.388559211 | 5.51E-07 | 1.41E-05 |
| ZMYND10 | 5.753061026 | 2.524329774 | 5.56E-07 | 1.42E-05 |
| SACS-AS1 | 0.122098423 | -3.033883527 | 5.81E-07 | 1.47E-05 |
| PALM2AKAP2 | 0.142413277 | -2.811844437 | 6.14E-07 | 1.55E-05 |
| PVALB | 31.20769033 | 4.963829683 | 6.17E-07 | 1.56E-05 |
| MME-AS1 | 0.112984141 | -3.145807813 | 6.24E-07 | 1.57E-05 |
| IL4I1 | 6.453038296 | 2.689978587 | 6.57E-07 | 1.65E-05 |
| AC113368.1 | 0.096223614 | -3.377465205 | 6.65E-07 | 1.67E-05 |
| CYP1A1 | 0.080975078 | -3.626378234 | 6.81E-07 | 1.71E-05 |
| LINC01705 | 53.65665033 | 5.745685088 | 6.93E-07 | 1.73E-05 |
| SLC6A4 | 13.89662321 | 3.796662455 | 7.07E-07 | 1.76E-05 |
| AC022081.1 | 0.082075073 | -3.606912062 | 7.20E-07 | 1.79E-05 |
| KIF15 | 5.825662718 | 2.542422178 | 7.36E-07 | 1.83E-05 |
| KIFC2 | 4.269550546 | 2.094084206 | 7.52E-07 | 1.86E-05 |
| P2RY12 | 0.172012409 | -2.539415449 | 7.59E-07 | 1.88E-05 |
| SHCBP1 | 6.090074542 | 2.606459887 | 7.62E-07 | 1.88E-05 |
| AC073359.1 | 0.103934937 | -3.266247411 | 7.71E-07 | 1.91E-05 |
| DSC1 | 0.164460687 | -2.604185339 | 7.72E-07 | 1.91E-05 |
| PDE9A-AS1 | 0.128694468 | -2.957978056 | 7.73E-07 | 1.91E-05 |
| PIMREG | 8.292861694 | 3.051870032 | 7.74E-07 | 1.91E-05 |
| TMEM145 | 13.79702127 | 3.786284923 | 7.79E-07 | 1.92E-05 |
| TRPM3 | 0.154363484 | -2.695596586 | 7.91E-07 | 1.94E-05 |
| LINC01942 | 0.080408739 | -3.636503885 | 8.34E-07 | 2.04E-05 |
| AC147651.2 | 0.091827502 | -3.444929892 | 8.45E-07 | 2.06E-05 |
| MUC5B | 7.322231889 | 2.872283463 | 8.50E-07 | 2.07E-05 |
| LEF1 | 4.057316588 | 2.020525879 | 8.50E-07 | 2.07E-05 |
| CNTNAP3P2 | 0.140533392 | -2.831015122 | 8.61E-07 | 2.10E-05 |
| IGHGP | 5.416472261 | 2.437353533 | 8.77E-07 | 2.13E-05 |
| GINS2 | 4.434318271 | 2.148712323 | 8.96E-07 | 2.17E-05 |
| ANKRD53 | 0.154489622 | -2.694418169 | 9.00E-07 | 2.18E-05 |
| FGFBP2 | 0.151781271 | -2.719934312 | 9.04E-07 | 2.19E-05 |
| LINC01985 | 0.107122706 | -3.222663779 | 9.06E-07 | 2.19E-05 |
| VSTM2A | 15.80264225 | 3.982093897 | 9.16E-07 | 2.21E-05 |
| FOXD3-AS1 | 21.33603383 | 4.415220112 | 9.21E-07 | 2.22E-05 |
| SOX17 | 0.219042839 | -2.190715046 | 9.27E-07 | 2.24E-05 |
| TNFRSF18 | 7.554336102 | 2.917304972 | 9.44E-07 | 2.27E-05 |
| ADAMDEC1 | 6.866308249 | 2.779534626 | 9.46E-07 | 2.27E-05 |
| ADAMTS14 | 8.583113204 | 3.101501026 | 9.79E-07 | 2.35E-05 |
| H2AC13 | 27.13664105 | 4.762170251 | 9.95E-07 | 2.38E-05 |
| EMX2OS | 0.194116867 | -2.365002613 | 9.97E-07 | 2.38E-05 |
| AC018816.1 | 9.45477215 | 3.241042691 | 1.00E-06 | 2.39E-05 |
| IL20 | 10.8715641 | 3.442487612 | 1.06E-06 | 2.52E-05 |
| IGHG1 | 9.523305773 | 3.251462455 | 1.06E-06 | 2.52E-05 |
| KCNE1B | 0.123460827 | -3.017874737 | 1.07E-06 | 2.54E-05 |
| SGO1 | 9.667893533 | 3.273201586 | 1.08E-06 | 2.55E-05 |
| SEC24AP1 | 0.169250202 | -2.562770543 | 1.11E-06 | 2.63E-05 |
| AC105021.1 | 0.091706207 | -3.446836809 | 1.12E-06 | 2.65E-05 |
| PTGFR | 0.223271021 | -2.163132084 | 1.15E-06 | 2.71E-05 |
| CLSPN | 7.327177971 | 2.873257658 | 1.15E-06 | 2.71E-05 |
| LRRN3 | 0.22376376 | -2.159951694 | 1.17E-06 | 2.75E-05 |
| GDF10 | 0.118493124 | -3.077124747 | 1.17E-06 | 2.75E-05 |
| AL109809.1 | 0.142092138 | -2.815101359 | 1.19E-06 | 2.80E-05 |
| MMP3 | 4.946134934 | 2.306301598 | 1.21E-06 | 2.83E-05 |
| CCNE1 | 7.007777817 | 2.808957035 | 1.23E-06 | 2.88E-05 |
| GFAP | 0.186299446 | -2.424304708 | 1.28E-06 | 2.98E-05 |
| IRX6 | 0.184019846 | -2.442066727 | 1.28E-06 | 2.99E-05 |
| WDR86 | 0.242377693 | -2.04467117 | 1.31E-06 | 3.06E-05 |
| AARD | 4.530892511 | 2.179795265 | 1.35E-06 | 3.13E-05 |
| KREMEN2 | 15.69537017 | 3.97226715 | 1.35E-06 | 3.14E-05 |
| APOBEC3B | 6.017135953 | 2.589076954 | 1.41E-06 | 3.26E-05 |
| NRG2 | 0.195998753 | -2.351083622 | 1.42E-06 | 3.28E-05 |
| SLC27A6 | 0.243377755 | -2.038730785 | 1.43E-06 | 3.31E-05 |
| F12 | 7.219659056 | 2.851930708 | 1.44E-06 | 3.32E-05 |
| MYBL1 | 4.410228204 | 2.140853309 | 1.44E-06 | 3.33E-05 |
| RNASE7 | 0.170921972 | -2.548590231 | 1.45E-06 | 3.35E-05 |
| FGF13-AS1 | 0.13155394 | -2.926273638 | 1.47E-06 | 3.39E-05 |
| AC055854.1 | 9.696684439 | 3.277491535 | 1.47E-06 | 3.39E-05 |
| CENPM | 7.035488708 | 2.814650641 | 1.48E-06 | 3.41E-05 |
| CASQ2 | 0.092846344 | -3.429011088 | 1.51E-06 | 3.48E-05 |
| RTBDN | 81.06889826 | 6.341076632 | 1.52E-06 | 3.50E-05 |
| PAPPA2 | 0.170514677 | -2.552032172 | 1.53E-06 | 3.50E-05 |
| TRBV26OR9-2 | 0.089144286 | -3.487713867 | 1.55E-06 | 3.55E-05 |
| CALML5 | 6.404862094 | 2.679167508 | 1.55E-06 | 3.56E-05 |
| HNRNPA1P66 | 0.100020046 | -3.321638919 | 1.58E-06 | 3.61E-05 |
| CSAG3 | 70.12155642 | 6.131786113 | 1.63E-06 | 3.71E-05 |
| ALPK3 | 0.169565918 | -2.560081874 | 1.64E-06 | 3.74E-05 |
| HTR2A | 0.170069864 | -2.555800574 | 1.67E-06 | 3.80E-05 |
| SYNDIG1 | 7.118157716 | 2.831503899 | 1.68E-06 | 3.83E-05 |
| FAM13C | 0.209995002 | -2.251573106 | 1.72E-06 | 3.91E-05 |
| GATA4 | 32.36618522 | 5.01641543 | 1.79E-06 | 4.05E-05 |
| KCNA2 | 0.13624184 | -2.875758273 | 1.79E-06 | 4.05E-05 |
| CHL1-AS2 | 0.122199925 | -3.032684699 | 1.81E-06 | 4.09E-05 |
| GLDN | 0.227134764 | -2.138379559 | 1.82E-06 | 4.10E-05 |
| AFAP1-AS1 | 13.05883175 | 3.706953933 | 1.82E-06 | 4.10E-05 |
| AC104237.3 | 0.119423454 | -3.065841892 | 1.84E-06 | 4.15E-05 |
| C10orf90 | 0.187439112 | -2.415506071 | 1.95E-06 | 4.37E-05 |
| E2F2 | 6.01836638 | 2.589371936 | 1.95E-06 | 4.37E-05 |
| AC036108.3 | 0.184144295 | -2.441091397 | 1.96E-06 | 4.39E-05 |
| AC015656.1 | 0.125245833 | -2.997165492 | 2.03E-06 | 4.53E-05 |
| AC010319.5 | 0.104277758 | -3.261496627 | 2.04E-06 | 4.56E-05 |
| ADGRG4 | 0.116484361 | -3.101791823 | 2.05E-06 | 4.58E-05 |
| TLCD1 | 5.44683621 | 2.445418485 | 2.08E-06 | 4.63E-05 |
| PGAP4 | 0.210891799 | -2.245425098 | 2.19E-06 | 4.88E-05 |
| ITIH2 | 0.201685725 | -2.309819122 | 2.19E-06 | 4.88E-05 |
| CCNO | 5.518068666 | 2.46416341 | 2.36E-06 | 5.22E-05 |
| BMP2 | 0.222763032 | -2.166418261 | 2.38E-06 | 5.25E-05 |
| LMO7DN | 0.119554255 | -3.064262618 | 2.38E-06 | 5.25E-05 |
| CNIH2 | 10.11761256 | 3.338796995 | 2.51E-06 | 5.52E-05 |
| SSTR2 | 4.19018738 | 2.067014761 | 2.52E-06 | 5.54E-05 |
| STX11 | 0.223538279 | -2.161406191 | 2.64E-06 | 5.77E-05 |
| RASSF9 | 0.24390939 | -2.035582798 | 2.67E-06 | 5.84E-05 |
| APCDD1L-DT | 0.222657557 | -2.16710152 | 2.69E-06 | 5.88E-05 |
| TLX3 | 76.60889602 | 6.259440026 | 2.71E-06 | 5.91E-05 |
| TUBB3 | 12.87635932 | 3.686652837 | 2.75E-06 | 5.98E-05 |
| CNTNAP3 | 0.203978898 | -2.293508184 | 2.78E-06 | 6.06E-05 |
| ADGRL3 | 0.245244485 | -2.027707402 | 2.84E-06 | 6.17E-05 |
| CR381670.1 | 0.137352153 | -2.864048568 | 2.92E-06 | 6.34E-05 |
| LINC02185 | 0.133652427 | -2.903442061 | 3.01E-06 | 6.52E-05 |
| GMNC | 0.114291844 | -3.129205646 | 3.08E-06 | 6.67E-05 |
| STIL | 4.29840027 | 2.103799834 | 3.11E-06 | 6.72E-05 |
| NKAPL | 0.152105525 | -2.716855542 | 3.12E-06 | 6.73E-05 |
| FAM149A | 0.228063031 | -2.132495489 | 3.14E-06 | 6.78E-05 |
| NTF4 | 0.184656363 | -2.437085115 | 3.15E-06 | 6.78E-05 |
| RAD51 | 5.982734592 | 2.580805063 | 3.20E-06 | 6.86E-05 |
| AC087242.1 | 0.118863152 | -3.072626545 | 3.21E-06 | 6.88E-05 |
| HSPB2 | 0.173421146 | -2.527648272 | 3.21E-06 | 6.88E-05 |
| LINC01586 | 0.1022082 | -3.290417147 | 3.22E-06 | 6.89E-05 |
| CAPN11 | 0.1966324 | -2.346427035 | 3.37E-06 | 7.20E-05 |
| PLAC1 | 27.27619997 | 4.769570762 | 3.46E-06 | 7.37E-05 |
| CAPN9 | 6.522852733 | 2.705503057 | 3.49E-06 | 7.44E-05 |
| LINC01186 | 0.123069865 | -3.022450546 | 3.50E-06 | 7.45E-05 |
| SLCO1A2 | 0.18737696 | -2.415984528 | 3.50E-06 | 7.45E-05 |
| PTK6 | 4.06279457 | 2.022472418 | 3.51E-06 | 7.47E-05 |
| PTH1R | 0.230316481 | -2.118310446 | 3.58E-06 | 7.59E-05 |
| TEDC2 | 7.165798253 | 2.841127426 | 3.65E-06 | 7.73E-05 |
| MRGPRF-AS1 | 0.120981907 | -3.047136784 | 3.86E-06 | 8.15E-05 |
| AC093110.1 | 0.178162668 | -2.488733031 | 3.87E-06 | 8.15E-05 |
| KLHL30 | 0.145562773 | -2.780286656 | 3.87E-06 | 8.15E-05 |
| MICU3 | 0.213542826 | -2.227402663 | 3.90E-06 | 8.21E-05 |
| AC006963.2 | 0.129611007 | -2.947739855 | 3.99E-06 | 8.36E-05 |
| MESTIT1 | 0.165582019 | -2.594382079 | 4.08E-06 | 8.53E-05 |
| CT83 | 27.22089585 | 4.766642642 | 4.15E-06 | 8.65E-05 |
| DCT | 0.174576436 | -2.518069256 | 4.15E-06 | 8.66E-05 |
| MIEN1 | 4.133080652 | 2.047217516 | 4.16E-06 | 8.68E-05 |
| HMGB3P10 | 0.127279687 | -2.973925904 | 4.20E-06 | 8.75E-05 |
| TMEM178A | 0.230454614 | -2.117445442 | 4.30E-06 | 8.93E-05 |
| SPC25 | 7.039475886 | 2.815468019 | 4.31E-06 | 8.94E-05 |
| E2F8 | 6.05836632 | 2.598928814 | 4.43E-06 | 9.18E-05 |
| LINC01625 | 0.126543802 | -2.982291246 | 4.65E-06 | 9.59E-05 |
| RAD54L | 6.916942204 | 2.790134402 | 4.67E-06 | 9.63E-05 |
| ST8SIA6-AS1 | 8.694624712 | 3.120123757 | 4.69E-06 | 9.67E-05 |
| GRIA2 | 9.850388825 | 3.300180673 | 4.75E-06 | 9.78E-05 |
| EFCAB1 | 0.197455127 | -2.340403268 | 4.77E-06 | 9.81E-05 |
| TLX1NB | 93.87695314 | 6.552699114 | 4.77E-06 | 9.81E-05 |
| AGAP11 | 0.146960164 | -2.766502956 | 4.86E-06 | 9.98E-05 |
| AC113133.1 | 0.091688806 | -3.447110577 | 4.87E-06 | 9.99E-05 |
| ATP1A3 | 9.285689536 | 3.215009046 | 4.97E-06 | 0.000101762 |
| CLEC4GP1 | 0.132312986 | -2.917973428 | 4.99E-06 | 0.000102085 |
| CXADRP3 | 32.62197882 | 5.027772392 | 5.17E-06 | 0.000105537 |
| DEPDC1B | 7.703938306 | 2.94559615 | 5.24E-06 | 0.000106816 |
| LINC01140 | 0.196036586 | -2.350805169 | 5.30E-06 | 0.000108029 |
| HMCN2 | 0.225483355 | -2.148907159 | 5.31E-06 | 0.000108162 |
| OTC | 0.099172284 | -3.333919208 | 5.40E-06 | 0.000109872 |
| H2BC4 | 6.466752666 | 2.693041433 | 5.92E-06 | 0.000119707 |
| ERCC6L | 7.242210437 | 2.856430098 | 6.05E-06 | 0.000121681 |
| SAMD11 | 6.444200264 | 2.688001329 | 6.10E-06 | 0.000122549 |
| POLQ | 6.146803602 | 2.61983639 | 6.10E-06 | 0.000122549 |
| GATA3-AS1 | 5.925638771 | 2.566970682 | 6.11E-06 | 0.000122734 |
| ESCO2 | 6.543978849 | 2.710168085 | 6.18E-06 | 0.000123988 |
| CPNE7 | 6.071061913 | 2.601948886 | 6.19E-06 | 0.000124289 |
| RGS6 | 0.199358593 | -2.326562304 | 6.31E-06 | 0.000126331 |
| F7 | 6.968106951 | 2.800766767 | 6.37E-06 | 0.000127448 |
| AC006159.1 | 0.166482638 | -2.586556367 | 6.78E-06 | 0.000135184 |
| CCDC141 | 0.165787679 | -2.592591305 | 7.20E-06 | 0.000142503 |
| ADM2 | 4.55351507 | 2.186980659 | 7.23E-06 | 0.000142957 |
| CAPZA3 | 0.1450415 | -2.785462344 | 7.41E-06 | 0.000146303 |
| RAD51AP1 | 4.873448537 | 2.28494301 | 7.45E-06 | 0.000146989 |
| MMP27 | 0.174215269 | -2.521057022 | 7.72E-06 | 0.000151751 |
| SGCG | 0.154438274 | -2.694897756 | 7.82E-06 | 0.000153306 |
| AC093895.2 | 34.98338979 | 5.128598184 | 7.83E-06 | 0.00015339 |
| LINC00511 | 7.61881256 | 2.929566162 | 7.93E-06 | 0.000155279 |
| ERVV-2 | 45.97853305 | 5.522888532 | 8.02E-06 | 0.000156993 |
| PNMA8C | 0.144627411 | -2.789587085 | 8.03E-06 | 0.000157126 |
| CLIC5 | 0.177445952 | -2.49454843 | 8.14E-06 | 0.0001591 |
| AC084030.1 | 0.096895318 | -3.367429236 | 8.26E-06 | 0.000161096 |
| H4C8 | 6.209773098 | 2.634540554 | 8.28E-06 | 0.000161411 |
| MMP10 | 8.624131378 | 3.108379156 | 8.40E-06 | 0.000163415 |
| WT1-AS | 27.91254093 | 4.802841557 | 8.55E-06 | 0.000166185 |
| AADACL3 | 0.113945881 | -3.13357932 | 8.60E-06 | 0.000167112 |
| TCEAL7 | 0.224695234 | -2.153958572 | 8.76E-06 | 0.000169884 |
| H2AW | 4.133811565 | 2.047472627 | 8.88E-06 | 0.000172078 |
| HSD17B6 | 7.987746417 | 2.997788533 | 9.05E-06 | 0.000175121 |
| DIAPH3 | 4.873527733 | 2.284966454 | 9.07E-06 | 0.00017527 |
| COX7B2 | 427.7892032 | 8.740756261 | 9.25E-06 | 0.00017822 |
| VGF | 13.4518646 | 3.749734257 | 9.28E-06 | 0.000178565 |
| LINC00922 | 24.06198626 | 4.588683834 | 9.29E-06 | 0.000178628 |
| ONECUT2 | 10.22784844 | 3.354430783 | 9.48E-06 | 0.000182226 |
| AL158071.1 | 0.168399326 | -2.570041733 | 9.50E-06 | 0.00018244 |
| MKNK2P1 | 50.60628024 | 5.66124453 | 9.52E-06 | 0.000182778 |
| AP001922.3 | 0.151184099 | -2.72562168 | 9.57E-06 | 0.000183482 |
| P4HA3 | 5.490238258 | 2.456868759 | 9.63E-06 | 0.000184357 |
| APLP1 | 6.10641482 | 2.610325598 | 9.99E-06 | 0.000190392 |
| GOLGA8M | 0.135833951 | -2.880083978 | 1.00E-05 | 0.000191193 |
| SLITRK6 | 5.817738224 | 2.540458382 | 1.02E-05 | 0.000193184 |
| SLC30A8 | 57.04718031 | 5.834083675 | 1.04E-05 | 0.00019663 |
| KCNE1 | 0.18121393 | -2.464234231 | 1.04E-05 | 0.000197332 |
| KCNF1 | 5.740520272 | 2.521181497 | 1.05E-05 | 0.0001998 |
| TP53AIP1 | 0.20896588 | -2.258660699 | 1.07E-05 | 0.000202843 |
| SLC35G2 | 0.246553748 | -2.020025912 | 1.10E-05 | 0.000207336 |
| AC013275.1 | 0.128156126 | -2.964025653 | 1.10E-05 | 0.000207696 |
| PLCXD3 | 0.204949658 | -2.286658514 | 1.13E-05 | 0.000213417 |
| CDC20B | 37.33911519 | 5.222615836 | 1.13E-05 | 0.000213541 |
| ARTN | 9.29477451 | 3.216419866 | 1.14E-05 | 0.000214263 |
| MT1M | 0.223878977 | -2.159209033 | 1.17E-05 | 0.000218924 |
| HAPLN1 | 40.03899418 | 5.323333828 | 1.17E-05 | 0.000219261 |
| SIX2 | 4.95517685 | 2.308936548 | 1.18E-05 | 0.000221165 |
| SDS | 4.744615525 | 2.246291185 | 1.21E-05 | 0.000224925 |
| HOTAIR | 6.703624513 | 2.744941342 | 1.22E-05 | 0.000227182 |
| AKAP6 | 0.216567777 | -2.207109493 | 1.25E-05 | 0.000232567 |
| NEIL3 | 10.58225937 | 3.403575778 | 1.25E-05 | 0.000233252 |
| METTL11B | 50.59511937 | 5.660926318 | 1.26E-05 | 0.000233494 |
| GABRA3 | 23.29956765 | 4.542231279 | 1.32E-05 | 0.000244267 |
| AC068057.2 | 0.121026478 | -3.046605388 | 1.32E-05 | 0.000244947 |
| EN1 | 4.732200414 | 2.242511175 | 1.33E-05 | 0.000247225 |
| AC092979.1 | 24.9598906 | 4.641539706 | 1.37E-05 | 0.000253769 |
| AC104237.2 | 0.133130528 | -2.909086666 | 1.38E-05 | 0.000255165 |
| STMND1 | 5.161978559 | 2.367924149 | 1.39E-05 | 0.000256404 |
| PDLIM3 | 0.180846868 | -2.467159479 | 1.40E-05 | 0.000258483 |
| WDR86-AS1 | 0.197768058 | -2.338118665 | 1.43E-05 | 0.000263834 |
| TEPP | 0.175559206 | -2.509970447 | 1.48E-05 | 0.000270624 |
| MYOCD | 0.183367126 | -2.447193076 | 1.48E-05 | 0.000271707 |
| AC093297.1 | 27.52215456 | 4.78252151 | 1.49E-05 | 0.000271965 |
| GABRD | 9.316166449 | 3.219736416 | 1.51E-05 | 0.000275565 |
| E2F7 | 7.417580803 | 2.890948738 | 1.52E-05 | 0.000276954 |
| H4C9 | 4.08294151 | 2.029608901 | 1.52E-05 | 0.000277523 |
| BRIP1 | 4.607821543 | 2.204084844 | 1.52E-05 | 0.000277777 |
| ADRB3 | 0.132856176 | -2.912062799 | 1.54E-05 | 0.000280267 |
| HAS1 | 0.204103129 | -2.292629796 | 1.54E-05 | 0.000280578 |
| NUAK2 | 4.741205568 | 2.245253946 | 1.55E-05 | 0.00028166 |
| IGFL3 | 33.92771924 | 5.084392544 | 1.57E-05 | 0.000284705 |
| GLDC | 5.512702067 | 2.462759633 | 1.58E-05 | 0.00028539 |
| CDH12 | 0.202146965 | -2.306523552 | 1.59E-05 | 0.000287502 |
| DAAM2-AS1 | 0.207150974 | -2.271245492 | 1.59E-05 | 0.000287549 |
| LINC00924 | 0.194097784 | -2.365144451 | 1.64E-05 | 0.000295602 |
| AOC2 | 0.198977631 | -2.329321839 | 1.64E-05 | 0.000295815 |
| AL358332.1 | 0.147089071 | -2.765238035 | 1.68E-05 | 0.000302683 |
| LINC01474 | 0.128113372 | -2.964507033 | 1.69E-05 | 0.000304043 |
| AC093496.1 | 0.154013847 | -2.698868025 | 1.70E-05 | 0.00030538 |
| CCNE2 | 5.380117754 | 2.427637749 | 1.72E-05 | 0.000309357 |
| SYT5 | 18.74292425 | 4.228274153 | 1.73E-05 | 0.000310939 |
| TMPRSS4 | 7.386953534 | 2.884979503 | 1.79E-05 | 0.000319777 |
| TMPRSS6 | 5.706150046 | 2.512517684 | 1.80E-05 | 0.000321618 |
| FAM228A | 0.155283779 | -2.687020962 | 1.82E-05 | 0.000325074 |
| POTEC | 65.56149845 | 6.034776923 | 1.83E-05 | 0.000326033 |
| AL603840.1 | 0.168193494 | -2.571806192 | 1.87E-05 | 0.00033238 |
| KIF18A | 5.395223262 | 2.431682662 | 1.87E-05 | 0.000332513 |
| GALR1 | 0.145640623 | -2.77951528 | 1.88E-05 | 0.000333269 |
| AC008667.2 | 0.116876945 | -3.096937725 | 1.94E-05 | 0.000343053 |
| GPR17 | 0.199450943 | -2.325894148 | 1.94E-05 | 0.000343529 |
| TPSP2 | 13.43812322 | 3.748259759 | 1.98E-05 | 0.000349664 |
| HOXA4 | 0.204148674 | -2.292307896 | 2.05E-05 | 0.00036109 |
| RPE65 | 0.160683225 | -2.637708769 | 2.08E-05 | 0.00036469 |
| PHYHIP | 0.23331657 | -2.099639325 | 2.10E-05 | 0.00036727 |
| ROBO2 | 6.421195172 | 2.68284185 | 2.10E-05 | 0.000368066 |
| LINC02544 | 12.70345523 | 3.667149046 | 2.14E-05 | 0.000373582 |
| UNC13A | 15.33036966 | 3.93832058 | 2.15E-05 | 0.000376257 |
| DOK7 | 4.881804817 | 2.287414615 | 2.17E-05 | 0.000378908 |
| ORC6 | 5.866714446 | 2.552552773 | 2.19E-05 | 0.000382772 |
| RPL6P4 | 19.93269762 | 4.317065067 | 2.21E-05 | 0.000385521 |
| AC008738.4 | 0.118813707 | -3.073226807 | 2.22E-05 | 0.00038595 |
| POC1A | 4.321422088 | 2.111506151 | 2.23E-05 | 0.000387404 |
| SCN9A | 0.198896438 | -2.329910658 | 2.25E-05 | 0.000390402 |
| MTHFD2P1 | 0.134880642 | -2.890244786 | 2.26E-05 | 0.000392784 |
| RBMS3-AS3 | 0.156564549 | -2.675170516 | 2.27E-05 | 0.000395011 |
| CNTFR-AS1 | 0.13284649 | -2.912167988 | 2.29E-05 | 0.000396859 |
| SCN3B | 0.219363728 | -2.188603099 | 2.32E-05 | 0.000402578 |
| AC040173.2 | 0.145028837 | -2.785588304 | 2.33E-05 | 0.000403407 |
| SLC7A14 | 0.170233303 | -2.554414791 | 2.34E-05 | 0.000405831 |
| AC135178.2 | 0.134151296 | -2.898067099 | 2.34E-05 | 0.000405831 |
| GAS1RR | 0.217551057 | -2.20057407 | 2.36E-05 | 0.000408624 |
| CRACD | 6.357370415 | 2.66843015 | 2.36E-05 | 0.000408624 |
| ARHGEF7-AS2 | 0.127367478 | -2.972931149 | 2.37E-05 | 0.000408964 |
| ZNF300P1 | 0.222907736 | -2.16548141 | 2.40E-05 | 0.000413952 |
| SGCZ | 0.162208381 | -2.624079731 | 2.43E-05 | 0.000418246 |
| CCL11 | 18.45607577 | 4.206023927 | 2.48E-05 | 0.000425743 |
| LINC01953 | Inf | Inf | 2.48E-05 | 0.000425867 |
| LINC01561 | 30.2117473 | 4.91703772 | 2.50E-05 | 0.000429023 |
| SLC47A1P1 | 0.140200941 | -2.834432059 | 2.52E-05 | 0.000432115 |
| FAM110D | 0.238979196 | -2.065043061 | 2.58E-05 | 0.000440586 |
| DQX1 | 15.05063487 | 3.911752439 | 2.59E-05 | 0.00044234 |
| TFR2 | 9.299027024 | 3.217079772 | 2.63E-05 | 0.000448349 |
| MYLKP1 | 0.153952081 | -2.699446728 | 2.68E-05 | 0.000456349 |
| CPB1 | 27.02215393 | 4.756070771 | 2.75E-05 | 0.000466807 |
| FABP6 | 20.13551924 | 4.331670771 | 2.83E-05 | 0.000479213 |
| PLPPR1 | 0.207917092 | -2.265919732 | 2.83E-05 | 0.000479213 |
| CSAG4 | 400.5076585 | 8.64568602 | 2.87E-05 | 0.000485875 |
| AL391421.1 | 8.017837149 | 3.003213115 | 2.90E-05 | 0.000489951 |
| OR2B6 | 23.83999605 | 4.575312092 | 2.93E-05 | 0.000494035 |
| BMPER | 0.243255162 | -2.039457676 | 2.99E-05 | 0.000502755 |
| CCDC187 | 28.49701219 | 4.832738761 | 3.03E-05 | 0.00050858 |
| KISS1R | 18.44232151 | 4.204948368 | 3.06E-05 | 0.000514509 |
| KLHL1 | 224.9715636 | 7.813598846 | 3.11E-05 | 0.000522046 |
| H2BC17 | 25.64464993 | 4.680585972 | 3.17E-05 | 0.000531021 |
| AC009779.4 | 0.198710591 | -2.331259323 | 3.19E-05 | 0.000533226 |
| HOXA6 | 0.197409847 | -2.340734143 | 3.20E-05 | 0.000534963 |
| LHX2 | 21.42728498 | 4.421377154 | 3.23E-05 | 0.000539308 |
| VSTM2A-OT1 | 19.01415217 | 4.249001706 | 3.24E-05 | 0.000540297 |
| AC110772.2 | 0.149227247 | -2.744417119 | 3.25E-05 | 0.000541978 |
| BMPR1B-DT | 20.82358705 | 4.380146702 | 3.28E-05 | 0.000545932 |
| LINC01238 | 8.785419427 | 3.135111164 | 3.28E-05 | 0.000545932 |
| CYP4F24P | 0.1771081 | -2.497297896 | 3.40E-05 | 0.000563904 |
| LMO7DN-IT1 | 0.134843537 | -2.890641723 | 3.45E-05 | 0.000571194 |
| SIM2 | 4.71551801 | 2.237416263 | 3.47E-05 | 0.000574027 |
| HSPB2-C11orf52 | 0.108231852 | -3.207802959 | 3.48E-05 | 0.000575102 |
| IGFBP1 | 0.148094842 | -2.7554067 | 3.59E-05 | 0.000591707 |
| EMX1 | 11.98655143 | 3.583344746 | 3.72E-05 | 0.000611533 |
| GPR182 | 0.202250365 | -2.305785786 | 3.79E-05 | 0.000622153 |
| GNGT1 | 28.05680949 | 4.810279055 | 3.81E-05 | 0.000624595 |
| CFAP45 | 6.456372046 | 2.690723716 | 3.84E-05 | 0.000628667 |
| CETP | 0.210622791 | -2.247266536 | 3.92E-05 | 0.0006414 |
| EDN2 | 5.759515157 | 2.525947369 | 4.00E-05 | 0.000653694 |
| AC096921.2 | 0.20670059 | -2.274385585 | 4.02E-05 | 0.000655295 |
| MROH6 | 4.038077626 | 2.013668645 | 4.02E-05 | 0.000655479 |
| AL161457.1 | 0.166542871 | -2.586034497 | 4.14E-05 | 0.000674555 |
| XKR7 | 30.14447495 | 4.913821696 | 4.19E-05 | 0.00068094 |
| LINC00906 | 0.142885494 | -2.807068637 | 4.31E-05 | 0.000696921 |
| SLC16A12 | 0.219622522 | -2.186902085 | 4.55E-05 | 0.000732245 |
| GTF2IP6 | 80.14380786 | 6.324519153 | 4.56E-05 | 0.000733543 |
| USP44 | 0.207358131 | -2.269803478 | 4.64E-05 | 0.000745865 |
| AP005271.1 | 0.123275093 | -3.020046756 | 4.67E-05 | 0.000749576 |
| P2RX6 | 0.185982073 | -2.426764531 | 4.67E-05 | 0.000749677 |
| LINC00844 | 0.178924105 | -2.482580332 | 4.89E-05 | 0.000781759 |
| AC009269.4 | 0.145923182 | -2.776719002 | 4.90E-05 | 0.000782702 |
| PCSK1N | 7.692946823 | 2.943536336 | 4.93E-05 | 0.000787021 |
| RIPPLY3 | 9.000770631 | 3.170048528 | 4.94E-05 | 0.000787675 |
| LINC00640 | 0.203145146 | -2.299417205 | 5.04E-05 | 0.000802041 |
| IQSEC3 | 0.230744526 | -2.11563167 | 5.04E-05 | 0.000802041 |
| FEZF2 | 0.163938646 | -2.608772103 | 5.04E-05 | 0.000802041 |
| MUC2 | 436.944552 | 8.771306404 | 5.06E-05 | 0.000804271 |
| GAL3ST2 | 16.86179806 | 4.075686482 | 5.08E-05 | 0.00080758 |
| LINC01497 | 0.106649609 | -3.229049421 | 5.13E-05 | 0.000814664 |
| TRIM59 | 4.001569159 | 2.000565844 | 5.29E-05 | 0.000836437 |
| HCN2 | 12.90377444 | 3.68972122 | 5.30E-05 | 0.000836742 |
| TEX26 | 0.137078669 | -2.866924001 | 5.34E-05 | 0.000841715 |
| MAGEA4-AS1 | 138.3698843 | 7.11238617 | 5.37E-05 | 0.000846276 |
| LINC01929 | 11.63411247 | 3.540289251 | 5.38E-05 | 0.000846276 |
| LINC01594 | 0.098494243 | -3.343816796 | 5.47E-05 | 0.000858707 |
| GNG4 | 7.070826082 | 2.821878774 | 5.48E-05 | 0.000859267 |
| LMX1A | 0.18633603 | -2.424021434 | 5.49E-05 | 0.000860863 |
| ERICH4 | 0.149928256 | -2.737655789 | 5.63E-05 | 0.000882107 |
| AL390061.1 | 51.1874589 | 5.677718483 | 5.64E-05 | 0.000882425 |
| CRYBG2 | 9.506517288 | 3.248916907 | 5.66E-05 | 0.000885106 |
| AC069061.2 | 109.7880414 | 6.778577108 | 5.67E-05 | 0.000886231 |
| AC002401.3 | 0.14231389 | -2.812851621 | 5.72E-05 | 0.000892211 |
| SPRR2F | 0.147914479 | -2.757164812 | 5.72E-05 | 0.000892586 |
| CNKSR2 | 0.2338542 | -2.096318752 | 5.96E-05 | 0.000926538 |
| LINC01088 | 0.233971311 | -2.095596451 | 5.97E-05 | 0.000927998 |
| CXCR2P1 | 9.336606072 | 3.222898214 | 6.04E-05 | 0.00093661 |
| NPW | 17.99857885 | 4.169811092 | 6.08E-05 | 0.000941287 |
| ORM1 | 40.93840449 | 5.355382972 | 6.08E-05 | 0.000941834 |
| ASTN1 | 0.248656548 | -2.007773674 | 6.11E-05 | 0.000944441 |
| IL6-AS1 | 0.180534254 | -2.469655496 | 6.12E-05 | 0.000944933 |
| SCN5A | 0.217354051 | -2.201881109 | 6.17E-05 | 0.000952793 |
| SYNGR3 | 9.033221164 | 3.175240531 | 6.22E-05 | 0.000958996 |
| IGFALS | 7.294450626 | 2.866799327 | 6.27E-05 | 0.000965354 |
| AC002401.2 | 0.132351213 | -2.917556679 | 6.35E-05 | 0.000976914 |
| UNC5A | 4.943256013 | 2.305461626 | 6.51E-05 | 0.001000705 |
| BOK-AS1 | 0.145996629 | -2.775993038 | 6.52E-05 | 0.00100107 |
| LINC02227 | 0.149224149 | -2.744447067 | 6.60E-05 | 0.001009547 |
| VSTM2L | 6.432734003 | 2.685432034 | 6.78E-05 | 0.00103428 |
| COL2A1 | 10.15677751 | 3.344370839 | 6.79E-05 | 0.001035993 |
| LINC02747 | 10.3703809 | 3.374396979 | 6.79E-05 | 0.001036078 |
| TNNI3 | 11.76550216 | 3.556490992 | 6.90E-05 | 0.001050661 |
| PAX7 | 7.954093056 | 2.99169744 | 6.96E-05 | 0.001058265 |
| NLGN1 | 0.236832397 | -2.07806165 | 7.01E-05 | 0.001065089 |
| AC092164.1 | 0.229801954 | -2.121537032 | 7.03E-05 | 0.001067497 |
| AC005515.1 | 12.90120023 | 3.689433384 | 7.16E-05 | 0.001083975 |
| TMC2 | 0.193773709 | -2.367555252 | 7.20E-05 | 0.001087713 |
| RHOXF1 | 0.1669904 | -2.582162929 | 7.32E-05 | 0.001105036 |
| HHIPL2 | 9.235548433 | 3.207197635 | 7.32E-05 | 0.001105208 |
| SIX3 | 8.236229287 | 3.041983993 | 7.42E-05 | 0.001118294 |
| NXPH4 | 5.479916513 | 2.454153914 | 7.42E-05 | 0.001118294 |
| EME1 | 6.116987504 | 2.612821329 | 7.44E-05 | 0.001119915 |
| AL133467.1 | 0.190287091 | -2.393750404 | 7.50E-05 | 0.0011278 |
| SNHG26 | 0.240441923 | -2.056239631 | 7.51E-05 | 0.001128045 |
| AC103702.2 | 20.68201023 | 4.370304513 | 7.61E-05 | 0.001140646 |
| EIF4E1B | 23.3777131 | 4.547061902 | 7.62E-05 | 0.001142215 |
| SLC25A24P2 | 18.51137038 | 4.210339796 | 7.81E-05 | 0.001168851 |
| AP004608.1 | 0.234275313 | -2.093723156 | 7.84E-05 | 0.001172812 |
| PHEX-AS1 | 0.179894695 | -2.474775451 | 7.87E-05 | 0.001175602 |
| GFRA2 | 0.228457773 | -2.130000568 | 7.87E-05 | 0.001175602 |
| NXNL1 | 0.136232534 | -2.875856819 | 7.92E-05 | 0.001182637 |
| C2CD4B | 0.236075754 | -2.082678218 | 7.94E-05 | 0.001184092 |
| MGARP | 0.201837882 | -2.308731121 | 8.01E-05 | 0.001194539 |
| CAPSL | 16.13371066 | 4.012006383 | 8.04E-05 | 0.001197096 |
| CPLX2 | 84.57558566 | 6.402169357 | 8.10E-05 | 0.001203414 |
| ODF3L1 | 0.205604019 | -2.282059632 | 8.12E-05 | 0.001206706 |
| SMYD1 | 0.032183543 | -4.957533014 | 8.15E-05 | 0.001209015 |
| AL022329.1 | 0.161235264 | -2.632760786 | 8.20E-05 | 0.001214431 |
| MMP12 | 5.690194588 | 2.508477989 | 8.29E-05 | 0.001225665 |
| LINC01224 | 14.07634037 | 3.8152004 | 8.42E-05 | 0.001242102 |
| CAMP | 20.45997989 | 4.354732822 | 8.45E-05 | 0.001245433 |
| TMEM270 | 20.56258973 | 4.361950069 | 8.74E-05 | 0.001282889 |
| LINC01589 | 0.184863944 | -2.435464226 | 8.82E-05 | 0.001291919 |
| AC254633.1 | 0.184770273 | -2.436195427 | 9.01E-05 | 0.001318079 |
| TF | 0.225284868 | -2.150177681 | 9.26E-05 | 0.001352294 |
| SIRLNT | 120.6813065 | 6.91505841 | 9.30E-05 | 0.001358385 |
| AC012213.4 | 26.23321804 | 4.713322889 | 9.32E-05 | 0.001359598 |
| PACSIN1 | 6.48926372 | 2.698054798 | 9.42E-05 | 0.001373225 |
| HOXA2 | 0.231012329 | -2.113958242 | 9.52E-05 | 0.001384996 |
| H3C4 | 11.0702052 | 3.468610059 | 9.62E-05 | 0.001396595 |
| MTFR2 | 7.1703724 | 2.842048049 | 9.75E-05 | 0.001414094 |
| LINC02377 | 61.90633264 | 5.952015091 | 9.89E-05 | 0.001431693 |
| COPDA1 | 17.22680862 | 4.106583552 | 0.000101328 | 0.001464194 |
| AC017037.4 | 35.84914208 | 5.163866689 | 0.000102028 | 0.00147306 |
| TMEFF2 | 0.241576538 | -2.04944775 | 0.00010287 | 0.001483331 |
| FABP9 | 0.124263962 | -3.008520133 | 0.000103942 | 0.001495892 |
| KLK4 | 8.594756851 | 3.103456826 | 0.000103977 | 0.001495892 |
| TICRR | 4.21578279 | 2.075800537 | 0.000104004 | 0.001495892 |
| AC079414.1 | 47.15492137 | 5.559336442 | 0.00010414 | 0.001496957 |
| CYP2A7P2 | 18.93824802 | 4.243230968 | 0.000104705 | 0.00150408 |
| AC010976.2 | 0.187969257 | -2.411431375 | 0.000104878 | 0.001504708 |
| CRB2 | 0.232506889 | -2.104654632 | 0.000105422 | 0.001511201 |
| IL21R | 6.211854079 | 2.63502394 | 0.000106069 | 0.001519191 |
| PTENP1-AS | 0.146138238 | -2.774594381 | 0.000106508 | 0.001524841 |
| CDC25A | 4.532792781 | 2.180400209 | 0.000108365 | 0.001547534 |
| SLC14A2 | 0.213862689 | -2.225243284 | 0.000108577 | 0.001549915 |
| CSAG2 | 35.24378641 | 5.139297023 | 0.000112119 | 0.001597139 |
| PDX1 | 42.84768787 | 5.421145451 | 0.000112904 | 0.001606307 |
| CABP1 | 0.212420597 | -2.235004436 | 0.000113287 | 0.001610407 |
| OR7E13P | 0.227269037 | -2.137526951 | 0.00011701 | 0.001657123 |
| KCNH1 | 8.056449027 | 3.010144094 | 0.000117278 | 0.001660226 |
| AP004782.1 | 0.146337987 | -2.772623773 | 0.000118416 | 0.001674954 |
| H2AC17 | 17.84747427 | 4.157648017 | 0.00011867 | 0.00167785 |
| CASP14 | 106.2534645 | 6.731366073 | 0.000119121 | 0.001681882 |
| H2BU1 | 8.926750545 | 3.158135111 | 0.000119221 | 0.001682151 |
| AGMO | 0.236536606 | -2.079864626 | 0.000121004 | 0.001704954 |
| LINC02104 | 0.177428537 | -2.494690026 | 0.000122057 | 0.001717902 |
| CERS3-AS1 | 0.169071916 | -2.564291053 | 0.00012338 | 0.001735101 |
| AL020994.3 | 0.132690601 | -2.91386191 | 0.000124033 | 0.001742127 |
| AL845331.3 | 0.15981957 | -2.645484021 | 0.000126696 | 0.001776606 |
| MRGPRX3 | 0.227983493 | -2.132998726 | 0.00012897 | 0.001804781 |
| TMEM215 | 16.05299931 | 4.004770967 | 0.000131842 | 0.001841111 |
| AC005225.4 | 0.213393363 | -2.228412788 | 0.000135882 | 0.001892975 |
| PRDM16-DT | 0.206722784 | -2.274230693 | 0.000138044 | 0.001919181 |
| A2ML1 | 11.38077695 | 3.508527147 | 0.000138152 | 0.001919892 |
| AC092376.2 | 0.236497981 | -2.080100226 | 0.000138479 | 0.001923656 |
| FAM162B | 0.226198673 | -2.144337627 | 0.000139872 | 0.001939846 |
| LINC00671 | 0.172150284 | -2.538259532 | 0.000143155 | 0.001980556 |
| AC079414.2 | 53.92481706 | 5.752877472 | 0.000143621 | 0.001986197 |
| GATA5 | 13.08754849 | 3.710122978 | 0.000143909 | 0.001989378 |
| OR2L13 | 0.191180526 | -2.386992522 | 0.000144506 | 0.00199601 |
| AC005906.2 | 0.166969045 | -2.582347432 | 0.000144648 | 0.001997163 |
| AP003071.4 | 0.205363963 | -2.283745052 | 0.000144984 | 0.002000988 |
| SPATA20P1 | 24.68167954 | 4.625368665 | 0.000145538 | 0.002007017 |
| KCNA1 | 0.237798405 | -2.072189056 | 0.000146394 | 0.002017183 |
| HORMAD2-AS1 | 0.156263204 | -2.677949997 | 0.000147737 | 0.002034049 |
| CDH7 | 10.9844937 | 3.457396469 | 0.000152692 | 0.00209382 |
| AC015712.4 | 0.230926261 | -2.114495846 | 0.000153263 | 0.00209828 |
| FAM230C | 91.24845113 | 6.511728165 | 0.000153852 | 0.002104658 |
| AC022784.1 | 16.48428133 | 4.043019086 | 0.000154087 | 0.002107027 |
| OPRD1 | 6.939425506 | 2.794816231 | 0.000156499 | 0.002136912 |
| STK32A-AS1 | 0.152517381 | -2.712954436 | 0.000156715 | 0.002137823 |
| RCOR2 | 5.060025926 | 2.339144777 | 0.000156872 | 0.002139116 |
| ORC1 | 4.881589361 | 2.287350941 | 0.000157095 | 0.002141303 |
| AL162413.1 | 24.40125887 | 4.608883674 | 0.000159148 | 0.002164488 |
| ZNF695 | 9.434298273 | 3.237915214 | 0.000159148 | 0.002164488 |
| PLEKHD1 | 5.500050512 | 2.459444868 | 0.000159958 | 0.002172511 |
| Z82185.1 | 22.22411166 | 4.474053848 | 0.000161374 | 0.002190117 |
| AP003555.2 | 0.199029126 | -2.328948526 | 0.000161511 | 0.002190117 |
| H2AC7 | 12.21656082 | 3.610766293 | 0.00016188 | 0.002194258 |
| MEF2C-AS1 | 0.216655448 | -2.206525579 | 0.000165184 | 0.002233719 |
| AC037198.2 | 5.171789248 | 2.370663486 | 0.0001688 | 0.002276302 |
| H2BC9 | 11.48380291 | 3.521528571 | 0.0001691 | 0.002279448 |
| AC010457.1 | 0.182223918 | -2.456215762 | 0.000169546 | 0.002283648 |
| H3C12 | 24.55628215 | 4.618020246 | 0.000170001 | 0.002288877 |
| LHFPL5 | 14.34069461 | 3.842042999 | 0.000170348 | 0.002292642 |
| C8orf88 | 0.235752534 | -2.084654814 | 0.000172655 | 0.002320039 |
| HTR1D | 12.49582707 | 3.643374488 | 0.00017493 | 0.002346914 |
| AC131097.2 | 11.60872173 | 3.537137217 | 0.000179327 | 0.002398465 |
| LINC01532 | 0.169910429 | -2.557153688 | 0.000181146 | 0.002419844 |
| OXCT1-AS1 | 0.203598057 | -2.296204301 | 0.000182988 | 0.002442531 |
| ADAMTS20 | 23.49257595 | 4.554133008 | 0.000183113 | 0.002443255 |
| H2BC7 | 15.21551428 | 3.927471192 | 0.000184239 | 0.002456359 |
| RTKN2 | 4.245797935 | 2.086035713 | 0.000185951 | 0.002476442 |
| AC007938.1 | 0.195678765 | -2.35344089 | 0.000186559 | 0.002483402 |
| AL031710.1 | 0.187085136 | -2.418233152 | 0.000186963 | 0.00248781 |
| SCT | 13.16592194 | 3.718736644 | 0.000190782 | 0.002532698 |
| MND1 | 6.440696386 | 2.687216685 | 0.000191433 | 0.002538378 |
| AP000696.1 | 17.81396282 | 4.154936583 | 0.00019153 | 0.002538688 |
| SHISA9 | 4.306460932 | 2.106502743 | 0.000193009 | 0.002556735 |
| LCN6 | 0.201176307 | -2.313467692 | 0.000193486 | 0.002560635 |
| AC024337.2 | 0.209625802 | -2.254111794 | 0.000193673 | 0.002561125 |
| AL589182.1 | 37.21463145 | 5.217798044 | 0.000193811 | 0.002561955 |
| GBX2 | 24.12323185 | 4.592351297 | 0.000194408 | 0.002567856 |
| FAM83A | 7.366203234 | 2.880921202 | 0.000194998 | 0.002574655 |
| MALRD1 | 8.044246405 | 3.007957274 | 0.000195312 | 0.002577804 |
| RPLP0P2 | 6.437826565 | 2.686573711 | 0.000198368 | 0.002614087 |
| KLHDC7B | 7.543357107 | 2.915206725 | 0.000199893 | 0.00263216 |
| LINC01833 | 7.962666551 | 2.993251644 | 0.000204371 | 0.002682837 |
| FGF14-AS2 | 0.240862776 | -2.053716644 | 0.000206826 | 0.002714022 |
| AC108515.1 | 53.3916329 | 5.738541767 | 0.00020824 | 0.002731524 |
| AC005682.1 | 0.244253018 | -2.033551709 | 0.00021118 | 0.002766905 |
| RHBDL1 | 6.088616729 | 2.6061145 | 0.000211891 | 0.002775159 |
| H2BC8 | 5.207100004 | 2.380480115 | 0.000213597 | 0.002794284 |
| MAGEA4 | 57.44567219 | 5.844126303 | 0.000214159 | 0.002799487 |
| KCNJ2-AS1 | 0.229290161 | -2.124753646 | 0.000214633 | 0.002804606 |
| AL136982.1 | 0.206707002 | -2.274340832 | 0.0002152 | 0.002810942 |
| LINC00636 | 0.176217079 | -2.504574339 | 0.000215456 | 0.002812704 |
| SOX2 | 10.03893676 | 3.327534574 | 0.000217447 | 0.002834866 |
| TMEM179 | 9.718724458 | 3.280766979 | 0.000225742 | 0.002932922 |
| IGHG4 | 4.599937312 | 2.2016142 | 0.000226564 | 0.002942486 |
| C9orf116 | 4.084249078 | 2.030070852 | 0.000230653 | 0.002989903 |
| AMZ1 | 6.356479693 | 2.668228002 | 0.000231729 | 0.003002111 |
| LINC01908 | 0.171064721 | -2.547385835 | 0.000231859 | 0.003002111 |
| ENTPD8 | 6.367848467 | 2.670806005 | 0.000232562 | 0.003006358 |
| AL032819.2 | 8.683507673 | 3.118277932 | 0.000235317 | 0.003031937 |
| WFDC21P | 8.176339718 | 3.03145514 | 0.000238282 | 0.003065516 |
| FOXG1 | 21.99005506 | 4.458779311 | 0.000240318 | 0.003088208 |
| AC093292.1 | 21.45278428 | 4.423092997 | 0.000242302 | 0.003110197 |
| CST6 | 6.272600509 | 2.649063683 | 0.000243562 | 0.003124025 |
| KLHL4 | 0.24210179 | -2.046314347 | 0.000243786 | 0.003125714 |
| LRRC70 | 0.242964575 | -2.041182116 | 0.000244478 | 0.003133419 |
| ELANE | 0.185940218 | -2.427089245 | 0.000244717 | 0.003135299 |
| OR10A3 | 0.161626108 | -2.629267837 | 0.000253579 | 0.003237895 |
| SPAG6 | 5.429729622 | 2.44088036 | 0.000254339 | 0.003246388 |
| LINC02242 | 0.178209529 | -2.488353617 | 0.000255145 | 0.003255461 |
| HCAR3 | 0.215237072 | -2.216001507 | 0.000255323 | 0.003256512 |
| LINC01644 | 13.43202309 | 3.74760471 | 0.000255435 | 0.003256725 |
| SYT9 | 4.07974657 | 2.028479536 | 0.000255632 | 0.003258016 |
| AC005089.1 | 0.187368676 | -2.41604831 | 0.000256665 | 0.003268743 |
| OSTN | 0.181316722 | -2.463416113 | 0.000260505 | 0.003310233 |
| PGLYRP2 | 4.611286708 | 2.205169369 | 0.000261489 | 0.003321509 |
| KCNH2 | 4.966655052 | 2.312274551 | 0.000261886 | 0.003324137 |
| CENPI | 4.636444404 | 2.213018856 | 0.000261891 | 0.003324137 |
| TMEM151A | 13.62105242 | 3.767766272 | 0.000267437 | 0.003381958 |
| SBK2 | 17.76368366 | 4.15085888 | 0.000267683 | 0.003383809 |
| LINC02580 | 0.216845246 | -2.205262282 | 0.000269904 | 0.003405585 |
| LINC02247 | 22.65642661 | 4.501848431 | 0.000271465 | 0.003421484 |
| AMER3 | 38.13477644 | 5.253035337 | 0.000272066 | 0.003427787 |
| FFAR4 | 0.243464637 | -2.038215857 | 0.000274882 | 0.003461988 |
| CPA2 | 0.192250883 | -2.378937867 | 0.000277664 | 0.003491877 |
| SRMS | 5.109368458 | 2.353144978 | 0.000282824 | 0.003550154 |
| GPR139 | 16.29509157 | 4.026365554 | 0.00028371 | 0.003557427 |
| PF4 | 0.168229452 | -2.57149779 | 0.000283955 | 0.003559189 |
| CCL7 | 13.13596533 | 3.715450319 | 0.000285273 | 0.003571771 |
| SCN11A | 0.231794265 | -2.109083225 | 0.000289049 | 0.003613753 |
| CCL16 | 0.196613016 | -2.346569259 | 0.000292282 | 0.003647483 |
| BEX1 | 6.884373047 | 2.783325276 | 0.000295163 | 0.003680753 |
| MSLNL | 17.99143212 | 4.169238125 | 0.000296867 | 0.003697948 |
| RNU6ATAC35P | 0.168870099 | -2.566014199 | 0.000298487 | 0.003708653 |
| AC009806.1 | 0.199540928 | -2.325243403 | 0.000306888 | 0.003799312 |
| LINC00466 | 19.77847497 | 4.305859286 | 0.000306897 | 0.003799312 |
| KIF18BP1 | 0.074650206 | -3.743709939 | 0.000307096 | 0.003800398 |
| AL161658.1 | 26.32408664 | 4.71831157 | 0.000311502 | 0.003846549 |
| IL17B | 0.240864223 | -2.053707979 | 0.000311768 | 0.00384845 |
| OTX1 | 5.792214274 | 2.534114974 | 0.000316114 | 0.003895354 |
| AC012213.1 | 16.53340761 | 4.047312196 | 0.000316801 | 0.003902105 |
| RNFT2 | 4.340106157 | 2.117730331 | 0.000317107 | 0.003904463 |
| AC092071.1 | 15.6485832 | 3.967960139 | 0.000319052 | 0.003926999 |
| CLPSL1 | 8.178088095 | 3.031763604 | 0.000319562 | 0.003931858 |
| EPHA8 | 13.04059702 | 3.704938015 | 0.000321291 | 0.003948858 |
| CHL1-AS1 | 0.195860434 | -2.352102108 | 0.000321797 | 0.003953655 |
| PGLYRP4 | 14.95202925 | 3.902269391 | 0.000323831 | 0.003977214 |
| BRSK2 | 7.186560512 | 2.845301462 | 0.000326007 | 0.004001057 |
| SLC7A11 | 4.662382312 | 2.221067309 | 0.00032822 | 0.004025327 |
| ITPKA | 10.17408628 | 3.34682733 | 0.000329581 | 0.004038573 |
| AC243562.2 | 0.244188821 | -2.033930938 | 0.000332671 | 0.004072597 |
| AC015849.5 | 12.41005039 | 3.633437068 | 0.000335479 | 0.004099617 |
| IFNL2 | 43.01166849 | 5.426656192 | 0.000338096 | 0.004125685 |
| AC092851.1 | 0.218536581 | -2.194053302 | 0.000338863 | 0.004133569 |
| ESR2 | 0.240371072 | -2.056664812 | 0.000341046 | 0.004158714 |
| AC095032.1 | 0.174569749 | -2.518124514 | 0.000345056 | 0.004203101 |
| TNFRSF9 | 6.486846341 | 2.697517265 | 0.000345726 | 0.004209767 |
| AC020637.1 | 0.17116123 | -2.546572142 | 0.000348602 | 0.004237228 |
| AC015908.3 | 0.210193989 | -2.250206686 | 0.000351452 | 0.004268825 |
| SLC5A8 | 15.44926002 | 3.949465833 | 0.00035205 | 0.004273055 |
| AL031777.1 | 10.46847353 | 3.387979185 | 0.000352186 | 0.004273186 |
| ANKRD30B | 4.68153041 | 2.226980229 | 0.000353525 | 0.004285774 |
| AL139241.1 | 0.214924304 | -2.218099457 | 0.000353575 | 0.004285774 |
| AL139023.1 | 35.94926147 | 5.167890228 | 0.000355216 | 0.004300776 |
| AC105053.1 | 0.16041026 | -2.640161678 | 0.000355887 | 0.004305844 |
| ZIC1 | 5.357434415 | 2.421542284 | 0.000356451 | 0.004311132 |
| LINC02335 | 89.87318499 | 6.489818825 | 0.000357041 | 0.004315213 |
| KCNH1-IT1 | 16.11601289 | 4.010422959 | 0.00035861 | 0.004328049 |
| AC108156.1 | 0.166136314 | -2.589560639 | 0.00035874 | 0.004328086 |
| SPINK4 | 13.32982071 | 3.736585471 | 0.000361256 | 0.004356896 |
| MIR23A | 0.191385777 | -2.385444477 | 0.000364416 | 0.004391908 |
| LINC01344 | 13.99248591 | 3.80658039 | 0.0003647 | 0.004392233 |
| AL445307.1 | 0.160832172 | -2.636372072 | 0.00036939 | 0.004439319 |
| SAA4 | 0.2359061 | -2.08371537 | 0.000369618 | 0.004440495 |
| AC019117.3 | 23.37645551 | 4.546984291 | 0.000373055 | 0.004477054 |
| MAGEC2 | 120.098965 | 6.908079908 | 0.000373676 | 0.004479906 |
| AC123595.1 | 0.197026346 | -2.343539538 | 0.000373686 | 0.004479906 |
| NBPF4 | 9.016424043 | 3.172555367 | 0.000376674 | 0.004514132 |
| AC073130.2 | 0.249242796 | -2.004376291 | 0.000382295 | 0.004576674 |
| HOXC-AS3 | 10.98605192 | 3.45760111 | 0.000385546 | 0.004612365 |
| MNX1 | 9.188015086 | 3.199753225 | 0.000387397 | 0.004632407 |
| CASP12 | 0.221521444 | -2.174481733 | 0.000387709 | 0.004633363 |
| ELAVL2 | 6.176543117 | 2.626799618 | 0.000388393 | 0.004639918 |
| TMEM220-AS1 | 0.244167521 | -2.03405679 | 0.00039438 | 0.004698278 |
| FAM25A | 21.14061472 | 4.401945422 | 0.00039478 | 0.004701404 |
| CLPSL2 | 10.35379882 | 3.372088286 | 0.000397519 | 0.004725779 |
| AC103591.2 | 0.177886685 | -2.490969567 | 0.000398021 | 0.004730099 |
| PSAT1P3 | 0.187354488 | -2.416157558 | 0.000402104 | 0.004770316 |
| RGS7BP | 0.224591143 | -2.154627061 | 0.000405109 | 0.004795969 |
| ANKRD22 | 4.35325937 | 2.122095979 | 0.000405363 | 0.00479731 |
| AC104260.2 | 0.216315267 | -2.208792603 | 0.000411308 | 0.004855884 |
| UTS2B | 0.214967306 | -2.217810833 | 0.000415697 | 0.004904305 |
| COX6CP1 | 6.256010747 | 2.645242991 | 0.000417483 | 0.004919741 |
| MIR27A | 0.182281254 | -2.455761898 | 0.000423604 | 0.004982093 |
| LRRC31 | 5.214819456 | 2.382617306 | 0.000424237 | 0.004987812 |
| USP24P1 | Inf | Inf | 0.000424975 | 0.004994082 |
| Z82186.1 | 17.18295464 | 4.102906227 | 0.000425792 | 0.005000929 |
| RBPMS-AS1 | 0.23253815 | -2.104460675 | 0.000427279 | 0.005016666 |
| AC105398.2 | 0.177611056 | -2.493206708 | 0.000427879 | 0.005020262 |
| AC093515.1 | 29.69809601 | 4.892298535 | 0.000430134 | 0.005041524 |
| AP001434.1 | 24.35792028 | 4.606319054 | 0.000434913 | 0.005090541 |
| HID1-AS1 | 0.214251161 | -2.222625073 | 0.000436075 | 0.005102396 |
| AC139099.2 | 0.15881124 | -2.654615073 | 0.00043658 | 0.005106555 |
| NFE4 | 20.11469543 | 4.330177988 | 0.000437017 | 0.005109905 |
| AC226118.1 | 0.235482205 | -2.086310053 | 0.000441103 | 0.005155915 |
| CCDC85A | 0.241198701 | -2.051705958 | 0.000441921 | 0.005163716 |
| LIN28A | 42.32023028 | 5.403275573 | 0.000443247 | 0.005177439 |
| AC090125.1 | 21.95451986 | 4.456446078 | 0.000449199 | 0.005234595 |
| RDH16 | 6.38925067 | 2.675646742 | 0.000450391 | 0.00524652 |
| AC017037.2 | 28.75490931 | 4.845736383 | 0.000453414 | 0.005276334 |
| DKK1 | 4.548155915 | 2.185281712 | 0.00045446 | 0.005282778 |
| LINC01385 | 28.73211228 | 4.844592153 | 0.00045663 | 0.005302905 |
| AL135960.1 | 0.221198528 | -2.176586308 | 0.000457428 | 0.005310362 |
| KCNIP1-OT1 | 0.198387518 | -2.333606835 | 0.000460001 | 0.005332976 |
| MIR548XHG | 25.06637717 | 4.647681594 | 0.000460836 | 0.005340849 |
| LINC02086 | 13.50830539 | 3.755774795 | 0.000461801 | 0.005350216 |
| H2BC11 | 6.022963922 | 2.590473617 | 0.000468043 | 0.005415181 |
| AC078882.1 | 107.9087566 | 6.753668131 | 0.000490769 | 0.005645585 |
| ORM2 | 19.40144633 | 4.278092301 | 0.00049251 | 0.005657983 |
| MED15P4 | 87.06595726 | 6.444036831 | 0.000495249 | 0.005683706 |
| TUBA3D | 7.372347047 | 2.882123986 | 0.000496847 | 0.005700125 |
| PRDM13 | 18.6860702 | 4.223891288 | 0.000503553 | 0.005771247 |
| LINC01354 | 0.245409195 | -2.026738791 | 0.000505107 | 0.005785284 |
| TIGIT | 4.179142868 | 2.063207079 | 0.000513684 | 0.005871588 |
| DLGAP2 | 0.238351947 | -2.068834685 | 0.000514412 | 0.005877937 |
| GP2 | 6.316364065 | 2.659094328 | 0.000519721 | 0.005928365 |
| AC134312.5 | 9.745926494 | 3.284799341 | 0.000519867 | 0.005928365 |
| LINC01606 | 13.48750173 | 3.753551239 | 0.000520125 | 0.005929325 |
| PRR11 | 6.812579291 | 2.768201116 | 0.00052106 | 0.00593307 |
| IGFL2 | 9.437262789 | 3.238368477 | 0.000521245 | 0.00593307 |
| ZIC2 | 7.654726441 | 2.936350821 | 0.000531323 | 0.006026575 |
| GAL | 6.015462787 | 2.588675732 | 0.000531481 | 0.006026575 |
| ADGRF4 | 10.51901377 | 3.394927543 | 0.000532566 | 0.006034868 |
| LINC01883 | 0.232880221 | -2.10233998 | 0.000535434 | 0.006065348 |
| AC098934.1 | 5.705607762 | 2.512380571 | 0.000537133 | 0.006082581 |
| LCT-AS1 | 16.90351873 | 4.079251692 | 0.000540433 | 0.006113861 |
| C10orf82 | 4.614777848 | 2.206261199 | 0.00054452 | 0.00615398 |
| NXPH1 | 16.81042636 | 4.071284411 | 0.000546853 | 0.006174213 |
| MARCHF4 | 28.42337258 | 4.829005843 | 0.000548929 | 0.006193547 |
| LIN28B | 23.21857258 | 4.537207377 | 0.00054922 | 0.006194784 |
| RNU6-242P | 0.180509927 | -2.469849913 | 0.000553974 | 0.006240157 |
| CST2 | 38.21722811 | 5.256151239 | 0.000554197 | 0.006240615 |
| KCNIP2-AS1 | 0.214747378 | -2.219287581 | 0.000557302 | 0.006271436 |
| RUNDC3A | 8.995199079 | 3.169155211 | 0.000570248 | 0.006402345 |
| SPOCD1 | 4.432928258 | 2.148260014 | 0.000576348 | 0.006456098 |
| FTHL17 | 2169.354046 | 11.08304981 | 0.000577839 | 0.006468422 |
| IL17D | 0.244213657 | -2.033784215 | 0.000585637 | 0.006544983 |
| H2BC18 | 6.59546757 | 2.72147494 | 0.000587782 | 0.00655783 |
| FOXP3 | 4.444550934 | 2.15203766 | 0.000588236 | 0.006558994 |
| AC010307.4 | 22.79917873 | 4.510909952 | 0.000591742 | 0.006589474 |
| AC073862.5 | 0.176158655 | -2.505052734 | 0.000592353 | 0.006594124 |
| AC061975.6 | 19.66415365 | 4.297496189 | 0.000595469 | 0.006626658 |
| LTB | 4.522262157 | 2.177044628 | 0.000607485 | 0.006745614 |
| AC016813.1 | 0.23922486 | -2.063560773 | 0.000613171 | 0.006805899 |
| AC020891.2 | 42.33748991 | 5.403863833 | 0.000617273 | 0.006849198 |
| AC116312.1 | 0.178386592 | -2.486920911 | 0.000618138 | 0.006856567 |
| U62317.1 | 8.324396386 | 3.057345664 | 0.000620964 | 0.006883443 |
| SLCO1B7 | 0.106256994 | -3.234370291 | 0.000627168 | 0.006940372 |
| MTDHP3 | 0.193561244 | -2.369137977 | 0.00064311 | 0.007089794 |
| GABRG1 | 0.184055845 | -2.441784529 | 0.000643434 | 0.007091066 |
| AC092916.1 | 17.69439689 | 4.145220684 | 0.000645291 | 0.007106947 |
| ULBP1 | 8.594130148 | 3.103351625 | 0.000647144 | 0.007125058 |
| RIIAD1 | 10.2900331 | 3.363175718 | 0.000648362 | 0.00713386 |
| H1-12P | 9.852123121 | 3.300434657 | 0.000656041 | 0.007202107 |
| LINC02593 | 4.718073843 | 2.238197999 | 0.000660236 | 0.007238845 |
| CST5 | 246.6761111 | 7.946474197 | 0.000669911 | 0.007328443 |
| LINC02289 | 0.228068175 | -2.132462949 | 0.000670173 | 0.007328958 |
| TLCD3B | 7.243396445 | 2.856666339 | 0.000686852 | 0.00748976 |
| AP003031.3 | 0.187992645 | -2.411251879 | 0.000690386 | 0.00752109 |
| RNU6-1189P | 0.193221355 | -2.371673546 | 0.000695528 | 0.00756021 |
| AC110772.1 | 0.176387346 | -2.503181029 | 0.000704782 | 0.007653493 |
| IFNL3 | 30.24030286 | 4.918400683 | 0.000706376 | 0.007665919 |
| TCAM1P | 8.431352917 | 3.075764148 | 0.000707503 | 0.007670838 |
| ENAM | 0.189831479 | -2.397208845 | 0.000708563 | 0.007678185 |
| LINC00052 | 52.45244447 | 5.712938104 | 0.000718209 | 0.00776963 |
| AC136621.1 | 0.223941136 | -2.158808533 | 0.000732819 | 0.007910137 |
| AC112777.1 | 9.030757965 | 3.17484708 | 0.000737978 | 0.007958271 |
| H2BC6 | 14.07254857 | 3.814811723 | 0.000749167 | 0.008058565 |
| LINC00989 | 0.228832161 | -2.127638269 | 0.000750367 | 0.008068929 |
| LINC02881 | 0.209205242 | -2.257009097 | 0.000755664 | 0.008123324 |
| GSTM5 | 0.124487723 | -3.005924619 | 0.000756779 | 0.008132757 |
| OR2W6P | 31.57859562 | 4.980875107 | 0.000763626 | 0.008196109 |
| POU4F1 | 9.64195908 | 3.269326307 | 0.000768607 | 0.008240939 |
| AC025423.3 | 9.413031602 | 3.234659438 | 0.000780488 | 0.008350729 |
| NBPF6 | 8.728520614 | 3.125737154 | 0.000783837 | 0.008381312 |
| LINC01340 | 0.202896455 | -2.301184437 | 0.000786918 | 0.008410063 |
| ETV7 | 4.010100174 | 2.003638276 | 0.000796866 | 0.008504639 |
| NKX6-1 | 6.830332409 | 2.771955791 | 0.00079881 | 0.008520053 |
| LINC00221 | 18.5500627 | 4.213352158 | 0.000813991 | 0.008657623 |
| TENM3-AS1 | 0.249966372 | -2.000194075 | 0.0008183 | 0.008689919 |
| RIMS2 | 4.815629517 | 2.267724405 | 0.000824661 | 0.008752023 |
| INMT-MINDY4 | 0.16470413 | -2.602051367 | 0.00082592 | 0.008762437 |
| LINC00461 | 10.89619352 | 3.445752327 | 0.000826854 | 0.008767113 |
| FOXD1 | 7.85785315 | 2.974135207 | 0.00084273 | 0.008910539 |
| DNAAF3 | 5.408011378 | 2.435098187 | 0.000843539 | 0.008916326 |
| LRRC46 | 4.288615409 | 2.100511945 | 0.000844075 | 0.008916476 |
| AC004160.2 | 0.199047434 | -2.328815822 | 0.000852226 | 0.008979388 |
| CCR8 | 9.36805555 | 3.227749631 | 0.000854176 | 0.008987019 |
| CST9 | 5.869646544 | 2.55327363 | 0.000857264 | 0.009016726 |
| INSM1 | 37.77392051 | 5.239318622 | 0.000859384 | 0.009031481 |
| AC024619.3 | 0.231391476 | -2.111592375 | 0.000860053 | 0.009032688 |
| HOXB-AS3 | 6.10986979 | 2.611141634 | 0.000860105 | 0.009032688 |
| LINC01956 | 7.509382509 | 2.908694281 | 0.000863404 | 0.009061759 |
| RNU6-813P | 10.93916258 | 3.451430396 | 0.000866248 | 0.009077655 |
| SALL4 | 7.058847357 | 2.819432624 | 0.000871642 | 0.009131374 |
| ARMC3 | 7.366097442 | 2.880900482 | 0.000872328 | 0.009135753 |
| AC011306.1 | 0.210529263 | -2.247907315 | 0.000877886 | 0.009179884 |
| AL358913.1 | 38.16473908 | 5.254168423 | 0.000892449 | 0.009312189 |
| MRGPRX7P | 0.181839388 | -2.459263361 | 0.000893074 | 0.009314335 |
| AC079160.1 | 15.02844535 | 3.909623869 | 0.000903827 | 0.009407904 |
| SLCO1C1 | 0.245057565 | -2.028807411 | 0.000904782 | 0.009414977 |
| AC015908.2 | 0.247849264 | -2.012465121 | 0.000909413 | 0.009454513 |
| CD1A | 7.794627647 | 2.962480106 | 0.000918482 | 0.009540086 |
| TPSG1 | 5.523585631 | 2.465605095 | 0.000919735 | 0.009550188 |
| MRPL15P1 | 0.202618129 | -2.303164831 | 0.000928145 | 0.009620533 |
| CAMK2N2 | 8.73086138 | 3.126123996 | 0.000941448 | 0.009738156 |
| AL138878.2 | 10.22059971 | 3.353407946 | 0.000946775 | 0.009789265 |
| OR7E101P | 0.22673816 | -2.140900881 | 0.000953888 | 0.009850882 |
| AC008687.3 | 0.216499589 | -2.207563808 | 0.00095789 | 0.009889219 |
| CCL24 | 0.231951889 | -2.108102499 | 0.000959275 | 0.009894534 |
| AC012462.1 | 28.08495431 | 4.811725551 | 0.000972909 | 0.010013976 |
| IL17REL | 8.830217823 | 3.142449027 | 0.000975091 | 0.010030384 |
| CYP4F23P | 4.144239669 | 2.051107439 | 0.000980012 | 0.01006887 |
| RPS4XP5 | 0.2043354 | -2.290988927 | 0.000986383 | 0.010125184 |
| AC009123.1 | 0.210928608 | -2.245173316 | 0.000987681 | 0.010135455 |
| MS4A8 | 9.647634487 | 3.27017525 | 0.00099477 | 0.010195942 |
| MAFA-AS1 | 15.51527486 | 3.95561735 | 0.001001268 | 0.010253308 |
| INSYN1-AS1 | 16.35250101 | 4.031439399 | 0.001002968 | 0.010267638 |
| AL161668.4 | 0.215627678 | -2.213385722 | 0.001006452 | 0.010294364 |
| LINC01894 | 0.209315947 | -2.256245863 | 0.001011108 | 0.010338566 |
| CELF3 | 23.0646041 | 4.527608624 | 0.001016135 | 0.010380636 |
| STON1-GTF2A1L | 0.205340781 | -2.28390792 | 0.001017052 | 0.010385709 |
| ELFN2 | 6.966548223 | 2.800444008 | 0.001019728 | 0.010404135 |
| DBX2 | 0.245096369 | -2.028578986 | 0.001021314 | 0.010414844 |
| OIP5 | 5.398008346 | 2.432427207 | 0.001028711 | 0.010478333 |
| HNRNPA1P21 | 7.850037559 | 2.972699557 | 0.001037918 | 0.010558916 |
| TPPP2 | 0.218902939 | -2.191636769 | 0.001044247 | 0.010610661 |
| AF015262.1 | 15.76785508 | 3.978914517 | 0.001045696 | 0.01061906 |
| AC013652.1 | 9.358267786 | 3.226241512 | 0.00105351 | 0.010679712 |
| MKRN3 | 7.31494235 | 2.870846494 | 0.0010606 | 0.010741645 |
| GRIN2D | 4.617648457 | 2.207158344 | 0.001063511 | 0.010764741 |
| RPL21P135 | 0.234438733 | -2.092717148 | 0.001071242 | 0.010830147 |
| H2AC11 | 4.751911184 | 2.248507872 | 0.00107212 | 0.010834392 |
| OR5E1P | 0.228683648 | -2.128574885 | 0.001072297 | 0.010834392 |
| AC073325.1 | 13.95086018 | 3.802282173 | 0.001078802 | 0.010890441 |
| LINC01612 | 0.135495873 | -2.883679185 | 0.001079713 | 0.010896421 |
| GABBR2 | 13.83480463 | 3.790230365 | 0.001085787 | 0.010951244 |
| H3C8 | 8.280791356 | 3.049768646 | 0.001087669 | 0.010966983 |
| AC105118.1 | 13.11324217 | 3.712952523 | 0.0010928 | 0.011012214 |
| AC099850.4 | 6.823624393 | 2.770538236 | 0.001093658 | 0.011017599 |
| HOXB9 | 6.225819709 | 2.638263799 | 0.001107547 | 0.011144363 |
| LINC01886 | 0.129509401 | -2.948871274 | 0.001131089 | 0.011357807 |
| LINC00518 | 16.67146437 | 4.059308926 | 0.001152938 | 0.011530345 |
| AUNIP | 4.667000625 | 2.222495661 | 0.001153001 | 0.011530345 |
| AC114803.1 | 44.61845793 | 5.479568748 | 0.001155147 | 0.011536096 |
| BMP3 | 0.088620417 | -3.496217073 | 0.001156428 | 0.011544328 |
| AC104984.5 | 12.9464561 | 3.694485331 | 0.001197512 | 0.01191278 |
| MAS1L | 0.214549086 | -2.220620343 | 0.001212231 | 0.012041522 |
| NKX3-2 | 7.68799485 | 2.94260737 | 0.001213602 | 0.01205163 |
| TCF21 | 0.205858531 | -2.28027486 | 0.00121658 | 0.012077686 |
| CLDN25 | 19.61230848 | 4.293687454 | 0.001225081 | 0.012155011 |
| AC024132.4 | 0.103832464 | -3.267670508 | 0.001229923 | 0.012195961 |
| AJM1 | 4.07540396 | 2.026943068 | 0.001242419 | 0.012301998 |
| GPR158 | 5.333027945 | 2.414954888 | 0.001249599 | 0.012365918 |
| AL133325.3 | 13.54199938 | 3.759368853 | 0.00125342 | 0.01239295 |
| MYO3B | 9.958858729 | 3.315980421 | 0.001263137 | 0.012478186 |
| ELK2AP | 30.54253505 | 4.932747907 | 0.001267779 | 0.012509557 |
| AC112721.2 | 12.10879181 | 3.597983018 | 0.001271023 | 0.012537942 |
| UMODL1 | 7.283649438 | 2.864661487 | 0.001277589 | 0.012588717 |
| OFCC1 | 12.32161171 | 3.623119073 | 0.001278739 | 0.012595859 |
| LINC02484 | 99.43485677 | 6.63567977 | 0.001297991 | 0.012742835 |
| RDM1 | 8.433984114 | 3.076214304 | 0.001298141 | 0.012742835 |
| AL117329.1 | 11.43131838 | 3.514919895 | 0.001300601 | 0.012759652 |
| LINC01665 | 24.18487336 | 4.596033078 | 0.001316415 | 0.012885158 |
| AC007368.1 | 0.204942655 | -2.28670781 | 0.001325902 | 0.012959442 |
| AC092325.1 | 0.092036328 | -3.441652772 | 0.00132701 | 0.012965409 |
| MT1JP | 0.194539176 | -2.361867382 | 0.001331221 | 0.012996539 |
| P2RX6P | 0.218988099 | -2.19107563 | 0.001335486 | 0.013027002 |
| MIR8071-1 | 11.20877507 | 3.486556719 | 0.001343512 | 0.013090328 |
| CCL23 | 0.248432934 | -2.009071653 | 0.001347829 | 0.01312489 |
| RN7SL826P | 70.64962162 | 6.142609929 | 0.001351669 | 0.013143535 |
| PWRN1 | 0.21123151 | -2.243103031 | 0.001357719 | 0.013194848 |
| DMBX1 | 9.004649178 | 3.17067007 | 0.001359225 | 0.013205729 |
| C3orf67 | 4.284766787 | 2.099216683 | 0.001362566 | 0.013230652 |
| KLF14 | 0.24520233 | -2.027955408 | 0.001364673 | 0.013247343 |
| DNM1P47 | 0.240156748 | -2.057951747 | 0.001366916 | 0.013261576 |
| CFAP47 | 8.562116401 | 3.097967449 | 0.001371934 | 0.013302692 |
| FOXD3 | 8.354301251 | 3.062519167 | 0.001375903 | 0.013337389 |
| AC138356.1 | 0.243005412 | -2.040939649 | 0.001378143 | 0.013351886 |
| JAKMIP1 | 6.833916008 | 2.772712516 | 0.001378181 | 0.013351886 |
| LINC02489 | 12.47609258 | 3.641094258 | 0.001400445 | 0.013544517 |
| KRT81 | 5.386307661 | 2.429296638 | 0.001420505 | 0.013715205 |
| PIF1 | 5.09336591 | 2.348619365 | 0.001432483 | 0.013815222 |
| SRGAP3-AS2 | 29.98569518 | 4.906202515 | 0.001446447 | 0.013934142 |
| PNCK | 5.97409582 | 2.578720378 | 0.001451296 | 0.013976914 |
| ARHGAP29-AS1 | 8.800733395 | 3.137623753 | 0.001463895 | 0.014071144 |
| KCNJ6 | 7.811732292 | 2.965642509 | 0.001467283 | 0.014099057 |
| AL928921.1 | 0.175688039 | -2.508912118 | 0.001472296 | 0.014139259 |
| LINC01484 | 0.234088553 | -2.094873704 | 0.001476322 | 0.014161982 |
| LAG3 | 4.01173759 | 2.004227242 | 0.001488197 | 0.014255869 |
| AC106799.1 | 0.246386606 | -2.021004263 | 0.001489993 | 0.014265061 |
| CASC16 | 15.77990278 | 3.980016412 | 0.001491929 | 0.014275593 |
| AC112721.1 | 13.94628724 | 3.801809196 | 0.001498528 | 0.014334714 |
| LINC01412 | 0.184410107 | -2.439010366 | 0.001504775 | 0.014382383 |
| AMH | 6.55278363 | 2.712107895 | 0.001509097 | 0.014415614 |
| LINC02620 | 9.101122467 | 3.186044488 | 0.001511774 | 0.014433103 |
| SHOX | 0.208963879 | -2.25867451 | 0.001517125 | 0.014476099 |
| LINC02197 | 11.21407402 | 3.487238592 | 0.001519471 | 0.01449038 |
| LINC01842 | 9.814882439 | 3.294970987 | 0.001521246 | 0.014503254 |
| DLX5 | 5.891651631 | 2.558672127 | 0.001530531 | 0.014583631 |
| AL356274.2 | 18.77367908 | 4.230639498 | 0.001546524 | 0.014707278 |
| AC133644.1 | 10.03499863 | 3.326968515 | 0.001562265 | 0.014843554 |
| FAM47E-STBD1 | 0.213259096 | -2.229320815 | 0.001583939 | 0.015012892 |
| PPM1E | 4.955755116 | 2.3091049 | 0.001586218 | 0.015030315 |
| VAX1 | 15.90107705 | 3.991052584 | 0.001587041 | 0.015033947 |
| AP001628.2 | 0.202909987 | -2.301088219 | 0.001588201 | 0.015036583 |
| CR381653.2 | 0.224551217 | -2.154883557 | 0.001594512 | 0.015086118 |
| VIP | 0.245435423 | -2.02658461 | 0.001596371 | 0.015097181 |
| AL096711.2 | 0.234218932 | -2.0940704 | 0.001600141 | 0.015128648 |
| KRT37 | 10.29680378 | 3.364124676 | 0.001602055 | 0.015142547 |
| NEU4 | 7.761207655 | 2.956281155 | 0.00160315 | 0.01514597 |
| SEZ6L | 11.12524085 | 3.475764664 | 0.001615044 | 0.015239995 |
| MOGAT1 | 0.209375541 | -2.255835177 | 0.00163661 | 0.015400924 |
| IGHE | 19.01233547 | 4.248863858 | 0.001652115 | 0.015533973 |
| LINC01429 | 16.77318261 | 4.068084553 | 0.001659805 | 0.015589104 |
| AC107057.1 | 37.55635747 | 5.23098524 | 0.001666273 | 0.015636941 |
| LINC02766 | 0.188356742 | -2.408460421 | 0.001684604 | 0.015774273 |
| AL391361.2 | 0.223593983 | -2.16104673 | 0.001703171 | 0.015917563 |
| PNLIPRP2 | 9.759776654 | 3.286848133 | 0.001705705 | 0.015936877 |
| PAX1 | 7.724746842 | 2.949487653 | 0.001717992 | 0.016025362 |
| AC090044.1 | 0.249530322 | -2.002712956 | 0.00172722 | 0.01609384 |
| Z82214.2 | 14.90423252 | 3.897650182 | 0.00173506 | 0.016140459 |
| MNX1-AS1 | 7.385658907 | 2.884726636 | 0.00173718 | 0.016151377 |
| CLCA4-AS1 | 0.210057548 | -2.251143466 | 0.001748464 | 0.016234172 |
| SMIM32 | 11.5561246 | 3.530585759 | 0.001756212 | 0.016288349 |
| ZFP42 | 31.61092329 | 4.982351269 | 0.001758336 | 0.016299228 |
| AC092801.1 | 21.51221418 | 4.427084119 | 0.001794712 | 0.016586875 |
| AC092198.1 | 9.452082138 | 3.240632166 | 0.001801288 | 0.016637535 |
| LINC00659 | 14.5166712 | 3.859638763 | 0.001820255 | 0.016782041 |
| ASIC5 | 23.07290663 | 4.528127855 | 0.001842188 | 0.01692938 |
| LINC01811 | 16.18434433 | 4.016527014 | 0.001847909 | 0.016968253 |
| AC120498.8 | 18.4613934 | 4.206439541 | 0.001864289 | 0.017091071 |
| TGFBR3L | 8.645838045 | 3.112005812 | 0.001876692 | 0.017190925 |
| C1DP5 | 0.196863398 | -2.344733194 | 0.001889694 | 0.017296095 |
| SULT1C3 | 0.110980614 | -3.171620406 | 0.001891951 | 0.017312116 |
| TUBA3E | 8.559061719 | 3.097452651 | 0.001903348 | 0.017397742 |
| SLC8A2 | 6.193488867 | 2.630752326 | 0.001907057 | 0.017426974 |
| APOBEC4 | 14.78202812 | 3.885772319 | 0.001911061 | 0.017454225 |
| MIR221 | 0.229098108 | -2.125962552 | 0.00191305 | 0.017467714 |
| CHST6 | 4.732898595 | 2.242724012 | 0.001926151 | 0.017577934 |
| SYNE1-AS1 | 0.190030516 | -2.395696983 | 0.001929669 | 0.017591223 |
| LRRC37A9P | 9.640700366 | 3.269137957 | 0.001935998 | 0.017639492 |
| CHST1 | 4.196172623 | 2.069074029 | 0.001938806 | 0.017655651 |
| AC002451.1 | 0.232934528 | -2.10200359 | 0.001940066 | 0.017662411 |
| CLDN9 | 6.824784138 | 2.770783415 | 0.001949036 | 0.017729889 |
| AC007128.2 | 25.87992169 | 4.693761347 | 0.001959396 | 0.017805146 |
| MCIDAS | 6.586704176 | 2.719556757 | 0.001971747 | 0.017912105 |
| FRG2DP | Inf | Inf | 0.001972216 | 0.017912105 |
| AC011487.1 | 0.222606547 | -2.167432069 | 0.001993221 | 0.01809325 |
| AL109741.1 | 0.246339488 | -2.021280188 | 0.002005023 | 0.018185875 |
| GDPD2 | 7.327275176 | 2.873276797 | 0.002029371 | 0.018367668 |
| MSLN | 14.73215987 | 3.880897053 | 0.002031748 | 0.018384302 |
| UPK1A | 6.419212408 | 2.6823963 | 0.002039928 | 0.018443647 |
| SPEF1 | 5.445038264 | 2.444942187 | 0.002053861 | 0.018545057 |
| ZIC5 | 8.464991319 | 3.081508589 | 0.002060393 | 0.018584377 |
| CSMD2 | 4.290656367 | 2.101198362 | 0.002095303 | 0.018869337 |
| AC105219.1 | 13.8037052 | 3.786983663 | 0.002100657 | 0.018892626 |
| AC055874.1 | 0.209634243 | -2.254053697 | 0.002105609 | 0.018932178 |
| BRINP3 | 8.480149112 | 3.084089633 | 0.002122604 | 0.019059874 |
| ASCL2 | 4.240640576 | 2.084282209 | 0.002144008 | 0.019231835 |
| EPO | 15.2652002 | 3.932174605 | 0.002147245 | 0.019255814 |
| RN7SL417P | 0.239302289 | -2.0630939 | 0.00214971 | 0.019267792 |
| SLC14A2-AS1 | 0.238244524 | -2.069485042 | 0.00217011 | 0.019420039 |
| NELL1 | 22.70176115 | 4.504732317 | 0.002199937 | 0.019671486 |
| CCDC196 | 7.912181548 | 2.98407553 | 0.002202921 | 0.019689139 |
| SLCO1B3-SLCO1B7 | 0.14950319 | -2.741751827 | 0.002203261 | 0.019689139 |
| HTR1E | 14.97765219 | 3.904739588 | 0.002206559 | 0.019710036 |
| AC093904.4 | 6.72355764 | 2.749224809 | 0.002214141 | 0.019772595 |
| AL023803.2 | 12.06223395 | 3.592425217 | 0.002218093 | 0.019797527 |
| LRRC2 | 0.064796902 | -3.94793135 | 0.002226212 | 0.019844039 |
| CYP21A1P | 4.65454189 | 2.218639182 | 0.002232646 | 0.019875437 |
| AC044893.1 | 0.233115524 | -2.100883014 | 0.00225108 | 0.020003021 |
| S100A9 | 7.203865196 | 2.848771185 | 0.002257834 | 0.020052594 |
| AC093821.1 | 0.201803503 | -2.308976879 | 0.002270391 | 0.020137922 |
| LINC02616 | 13.06202047 | 3.70730617 | 0.002287511 | 0.0202687 |
| AP005262.1 | 13.09204529 | 3.710618593 | 0.002308527 | 0.020439 |
| OR56A3 | 17.50484355 | 4.129682263 | 0.002311243 | 0.020452431 |
| PLPPR5 | 8.844425839 | 3.144768489 | 0.002320733 | 0.02049921 |
| CST9L | 9.041524861 | 3.176566105 | 0.002326409 | 0.020544024 |
| TCEAL6 | 0.224356788 | -2.156133259 | 0.002338846 | 0.020632499 |
| IGHEP1 | 16.45546609 | 4.040494985 | 0.002342261 | 0.020651954 |
| GRM8 | 7.806398867 | 2.964657179 | 0.002371922 | 0.020902674 |
| AP001542.1 | 10.27539469 | 3.361121905 | 0.002378296 | 0.020939533 |
| LINC02408 | 10.33000689 | 3.368769312 | 0.002406583 | 0.021148068 |
| FFAR3 | 0.213429798 | -2.228166484 | 0.002408973 | 0.021159079 |
| C6orf58 | 0.242174587 | -2.045880616 | 0.002412051 | 0.021168647 |
| AC010595.1 | 13.58885645 | 3.764352148 | 0.002416217 | 0.021194525 |
| H1-6 | 14.71202805 | 3.87892423 | 0.002428194 | 0.021261663 |
| FAM72D | 7.804291163 | 2.964267603 | 0.002479751 | 0.021646136 |
| AL449403.3 | 0.24402953 | -2.034872355 | 0.002480968 | 0.021651219 |
| CYP21A2 | 4.997073092 | 2.32108332 | 0.002488494 | 0.021705801 |
| AC009005.1 | 5.74541802 | 2.522411863 | 0.002490955 | 0.021721712 |
| SLC25A34 | 0.23636112 | -2.080935355 | 0.002503756 | 0.021827761 |
| CCDC60 | 11.84774019 | 3.566540004 | 0.002514217 | 0.021902178 |
| PRICKLE2-DT | 22.71370109 | 4.505490901 | 0.002518495 | 0.02193385 |
| RNA5SP515 | 0.212262238 | -2.236080359 | 0.002521048 | 0.021947755 |
| CIB3 | 8.500746815 | 3.087589592 | 0.002521378 | 0.021947755 |
| LINC01366 | 0.227290896 | -2.137388198 | 0.00253246 | 0.022010542 |
| NKX2-5 | 7.751227698 | 2.954424834 | 0.002572899 | 0.022276922 |
| KCNG1 | 5.220815552 | 2.38427519 | 0.002601087 | 0.022492449 |
| TRPM1 | 0.220349054 | -2.182137394 | 0.002605551 | 0.022525343 |
| ERMN | 5.940789043 | 2.570654559 | 0.002614884 | 0.022600304 |
| NCCRP1 | 4.97241675 | 2.313947217 | 0.002643067 | 0.02279061 |
| GPR26 | 23.87503547 | 4.577430972 | 0.002647255 | 0.022816527 |
| AF127577.3 | 15.4791571 | 3.952255009 | 0.0026529 | 0.022858017 |
| KCNK9 | 8.692804121 | 3.119821636 | 0.002664127 | 0.022915654 |
| AC026369.1 | 0.243579675 | -2.037534339 | 0.002685109 | 0.023078688 |
| POU6F2-AS2 | 19.35959638 | 4.27497697 | 0.002698103 | 0.023167036 |
| C11orf87 | 0.220018014 | -2.184306444 | 0.002730738 | 0.023376675 |
| PAX2 | 5.751872073 | 2.524031589 | 0.002735111 | 0.0234082 |
| CGA | 422.3472523 | 8.722285855 | 0.002739745 | 0.023436149 |
| FSD1 | 7.446229428 | 2.896510068 | 0.0027511 | 0.023521491 |
| AC106900.1 | 17.55070558 | 4.133457127 | 0.002755469 | 0.02354705 |
| KLHL33 | 0.05930317 | -4.075746955 | 0.002757551 | 0.023551641 |
| AC004990.1 | 10.49269242 | 3.391313016 | 0.002779618 | 0.023705931 |
| SFTPA1 | 0.238930721 | -2.065335731 | 0.002786983 | 0.023750941 |
| OTOR | 8.909542706 | 3.155351385 | 0.002800091 | 0.0238199 |
| IVL | 17.39515912 | 4.120613971 | 0.002800658 | 0.0238199 |
| AL136982.6 | 0.239231341 | -2.063521691 | 0.002806303 | 0.023861966 |
| MIR8071-2 | 8.655742703 | 3.113657615 | 0.002900058 | 0.024512601 |
| LINC01980 | 9.575964273 | 3.25941777 | 0.002909992 | 0.024572228 |
| UGT2B28 | 0.241512881 | -2.049827955 | 0.002928124 | 0.024694789 |
| OR1F1 | 19.06647628 | 4.252966335 | 0.002929688 | 0.024701872 |
| LINC01267 | 0.239706824 | -2.060657114 | 0.00296206 | 0.024931705 |
| DEFB126 | 21.79911155 | 4.446197432 | 0.002964708 | 0.024947842 |
| LINC01402 | 0.236827731 | -2.078090076 | 0.00303019 | 0.025417407 |
| AP005901.3 | 27.62252096 | 4.787773087 | 0.003038588 | 0.025475324 |
| MYOM1 | 0.036662345 | -4.769557126 | 0.003041379 | 0.025492459 |
| FCRLB | 8.999860216 | 3.169902594 | 0.003048524 | 0.025539805 |
| CLPS | 8.195205388 | 3.034780107 | 0.003052236 | 0.02556029 |
| KIAA0319 | 5.382466361 | 2.428267398 | 0.003052467 | 0.02556029 |
| AC093642.2 | 4.509142156 | 2.172852993 | 0.003091228 | 0.025859485 |
| GUCY1B2 | 7.07407421 | 2.822541354 | 0.003119637 | 0.026039698 |
| NKX2-3 | 9.311920022 | 3.219078667 | 0.003137595 | 0.026151221 |
| AC090796.1 | 44.57260492 | 5.478085373 | 0.003170904 | 0.026377315 |
| SYNGR4 | 9.166864052 | 3.196428278 | 0.003174074 | 0.026378586 |
| AC245123.1 | 0.209042395 | -2.258132539 | 0.003180895 | 0.02642179 |
| H4C14 | 7.429456723 | 2.893256718 | 0.003222938 | 0.0267074 |
| LINC02351 | 0.202831557 | -2.301645967 | 0.00323551 | 0.026787071 |
| LINC00514 | 7.53076465 | 2.912796359 | 0.003235859 | 0.026787071 |
| AP005230.1 | 12.30070076 | 3.620668602 | 0.003246624 | 0.02685014 |
| C8orf49 | 23.14305227 | 4.532507245 | 0.003249401 | 0.026866592 |
| KNOP1P5 | 14.44247559 | 3.852246151 | 0.00326188 | 0.026943668 |
| PGK2 | 25.42280524 | 4.668051326 | 0.003297187 | 0.027202389 |
| C6orf99 | 6.424263409 | 2.683531048 | 0.00332598 | 0.027401675 |
| SLURP1 | 7.747498928 | 2.95373065 | 0.003358505 | 0.027628136 |
| LRFN2 | 6.819859675 | 2.769742055 | 0.003367228 | 0.027693219 |
| MGAT5B | 4.432465715 | 2.148109472 | 0.003417732 | 0.028047751 |
| TGIF2LX | 27.82426252 | 4.798271545 | 0.003453497 | 0.02830723 |
| EFNA2 | 12.48817618 | 3.642490891 | 0.003485156 | 0.028525629 |
| DMRT1 | 12.79528926 | 3.677540857 | 0.00350877 | 0.028691379 |
| LINC01475 | 22.0148661 | 4.460406165 | 0.003514739 | 0.028726425 |
| AC109462.2 | 0.218180749 | -2.196404283 | 0.003546161 | 0.028949972 |
| C20orf204 | 5.266149822 | 2.396748567 | 0.003546833 | 0.028949972 |
| AC093627.6 | 0.244298982 | -2.033280245 | 0.00354718 | 0.028949972 |
| AC116025.2 | 14.48373251 | 3.856361533 | 0.003568634 | 0.029055597 |
| AC138904.1 | 5.804299779 | 2.537122033 | 0.003572679 | 0.029074657 |
| SPDYC | 163.0976113 | 7.349591843 | 0.003580838 | 0.029113285 |
| RNU6ATAC18P | 0.239487962 | -2.061974953 | 0.003601812 | 0.029262898 |
| CYCSP6 | 11.84112439 | 3.565734175 | 0.003605894 | 0.029289092 |
| LINC01281 | 10.376797 | 3.375289292 | 0.003625717 | 0.029436092 |
| IGF2BP3 | 6.268078354 | 2.648023214 | 0.003645738 | 0.029589905 |
| AC008406.3 | 10.05697334 | 3.330124285 | 0.003652925 | 0.029635838 |
| LINC00603 | 0.218841243 | -2.192043439 | 0.003658349 | 0.029665745 |
| DPYSL5 | 21.90344829 | 4.453086108 | 0.003688638 | 0.029897154 |
| PRSS41 | 13.2605317 | 3.729066719 | 0.003721265 | 0.030118684 |
| LINC01518 | 33.03288002 | 5.045830854 | 0.003733238 | 0.030208429 |
| ABCB11 | 0.235432062 | -2.086617289 | 0.003759245 | 0.030346187 |
| AC131097.1 | 11.53363969 | 3.527775953 | 0.003759429 | 0.030346187 |
| PRSS29P | 11.15216379 | 3.479251749 | 0.003770391 | 0.030393768 |
| RHBG | 6.394791734 | 2.676897374 | 0.003791155 | 0.03053951 |
| KLKP1 | 9.380384455 | 3.229647053 | 0.00380171 | 0.03060229 |
| PSLNR | 65.19110955 | 6.026603325 | 0.003803512 | 0.03060294 |
| RNU6-848P | 31.22415172 | 4.964590473 | 0.003829637 | 0.030798616 |
| PPBP | 0.239651125 | -2.060992384 | 0.003836008 | 0.030825286 |
| KCNA4 | 0.13888604 | -2.848026501 | 0.003903087 | 0.031256751 |
| SELENOOLP | 26.78449899 | 4.743326405 | 0.003916434 | 0.031348925 |
| HMX2 | 27.22014482 | 4.766602838 | 0.003929719 | 0.031433148 |
| HBG2 | 0.201876964 | -2.308451798 | 0.003934639 | 0.03145039 |
| LINC00670 | 0.213682786 | -2.226457405 | 0.003941997 | 0.03147891 |
| LGALS9B | 7.139806608 | 2.835884997 | 0.003943511 | 0.03147891 |
| IL36RN | 8.682283428 | 3.118074519 | 0.003952987 | 0.031537958 |
| SYN1 | 4.200207852 | 2.070460723 | 0.00395438 | 0.031541698 |
| AL162426.1 | 0.241686417 | -2.048791703 | 0.003969861 | 0.03163561 |
| AC244669.2 | 0.204368663 | -2.290754101 | 0.003982196 | 0.031696904 |
| CYP1A2 | 0.191588746 | -2.383915273 | 0.004001848 | 0.031845902 |
| PGA5 | 0.198573798 | -2.332252824 | 0.004023733 | 0.031990227 |
| AC093063.1 | 11.66985205 | 3.544714365 | 0.004058565 | 0.032229612 |
| LINC02438 | 15.98801197 | 3.998918653 | 0.004059998 | 0.032230902 |
| AC009320.1 | 0.248425517 | -2.009114729 | 0.004076357 | 0.032333293 |
| KCNG3 | 7.059167709 | 2.819498097 | 0.004092428 | 0.032432395 |
| MAGEA10 | 50.73448479 | 5.664894791 | 0.004092653 | 0.032432395 |
| ERVV-1 | 9.88384311 | 3.30507211 | 0.004152748 | 0.032817188 |
| KCNJ16 | 0.202567053 | -2.30352855 | 0.004166996 | 0.032906927 |
| LINC02466 | 20.46828813 | 4.355318542 | 0.004177469 | 0.032974378 |
| MGAT3-AS1 | 0.240194294 | -2.057726214 | 0.004185523 | 0.033007415 |
| LINC00460 | 7.846412205 | 2.972033128 | 0.00418833 | 0.033021916 |
| IDO1 | 4.180539763 | 2.063689225 | 0.004193956 | 0.033058642 |
| AC010947.1 | 14.43085778 | 3.851085152 | 0.004198434 | 0.033086295 |
| NOTUM | 5.625897188 | 2.492083188 | 0.004231278 | 0.033314359 |
| LINC02475 | 7.467242101 | 2.900575507 | 0.004246709 | 0.033412728 |
| TERT | 7.025325595 | 2.81256509 | 0.004251598 | 0.03343578 |
| AC096637.3 | 7.803006679 | 2.964030135 | 0.004295572 | 0.033727204 |
| AC025252.3 | 27.5198751 | 4.782402017 | 0.004372588 | 0.034276711 |
| LINC00858 | 8.547389134 | 3.095483805 | 0.00437566 | 0.034292914 |
| LINC01297 | 20.60132025 | 4.364664891 | 0.004383268 | 0.034332757 |
| LINC01194 | 11.94442187 | 3.578265122 | 0.004383761 | 0.034332757 |
| AC026355.3 | 18.89702163 | 4.240086963 | 0.004415602 | 0.034542497 |
| LINC01633 | 26.86677808 | 4.747751415 | 0.004419802 | 0.034561852 |
| DLL3 | 7.037799746 | 2.815124464 | 0.004422728 | 0.034566551 |
| H2AC9P | 10.30997016 | 3.365968252 | 0.004435925 | 0.034645891 |
| SIRPB3P | 0.249301844 | -2.004034542 | 0.004454995 | 0.034731256 |
| PPY2P | 19.6453165 | 4.296113506 | 0.004465428 | 0.034804639 |
| NT5DC4 | 8.870540185 | 3.149021962 | 0.004475192 | 0.034856867 |
| AC100801.1 | 4.500064043 | 2.169945533 | 0.004497936 | 0.034986109 |
| LYPD8 | 6.568051134 | 2.715465359 | 0.004527359 | 0.03519091 |
| NUPR2 | 4.061425172 | 2.021986064 | 0.004562721 | 0.035393237 |
| CACNA1B | 6.283615474 | 2.651594897 | 0.004566037 | 0.035410907 |
| AL691420.1 | 15.83582996 | 3.985120576 | 0.004579494 | 0.035499139 |
| MIR6503 | 0.227926398 | -2.133360072 | 0.00458106 | 0.035503212 |
| TCL1B | 38.88676493 | 5.281207314 | 0.004585482 | 0.035521351 |
| AF279873.3 | 17.30550399 | 4.113159053 | 0.004589734 | 0.035546222 |
| DPEP1 | 5.483932311 | 2.455210764 | 0.004608528 | 0.035635177 |
| DMRTA2 | 8.89487796 | 3.152974811 | 0.004621625 | 0.03572026 |
| CXCL17 | 6.393554232 | 2.676618161 | 0.004656995 | 0.035963803 |
| DLX2 | 4.307286437 | 2.106779267 | 0.004667974 | 0.036029539 |
| AC024595.1 | 18.01024129 | 4.170745605 | 0.004681551 | 0.036093521 |
| TMEM92 | 6.07736541 | 2.603446038 | 0.004712246 | 0.036280998 |
| LINC00392 | 174.8042423 | 7.449596387 | 0.004717645 | 0.036314376 |
| HAR1B | 7.341342778 | 2.876043965 | 0.004724998 | 0.036354576 |
| CYSRT1 | 4.864513774 | 2.28229561 | 0.004726839 | 0.036360543 |
| ADAMTS19 | 4.836667025 | 2.27401322 | 0.004758287 | 0.03652761 |
| SCGB1A1 | 0.077683137 | -3.686254726 | 0.004785456 | 0.03668545 |
| MUC19 | 16.52443955 | 4.046529436 | 0.004786274 | 0.03668545 |
| AL162574.1 | 0.226776473 | -2.140657121 | 0.004842397 | 0.037049059 |
| PEX5L | 6.541360712 | 2.709590772 | 0.004845853 | 0.037058887 |
| IFNB1 | 16.37667171 | 4.033570278 | 0.004873228 | 0.037217598 |
| MYOC | 0.071239724 | -3.811174263 | 0.004904069 | 0.037400011 |
| INA | 14.19179168 | 3.826984833 | 0.004917533 | 0.037464356 |
| PYY | 6.416279498 | 2.681736988 | 0.00492342 | 0.037500836 |
| AC016705.1 | 13.31506921 | 3.734988023 | 0.004934695 | 0.037561569 |
| TUBA4B | 8.264097087 | 3.046857203 | 0.004965524 | 0.037770963 |
| MIR7152 | 0.215745307 | -2.212598919 | 0.004980782 | 0.037861712 |
| AC080037.2 | 12.39183696 | 3.631318163 | 0.004983308 | 0.037872473 |
| ENTHD1 | 7.460681832 | 2.899307484 | 0.00500912 | 0.037992506 |
| GABRA5 | 67.04269798 | 6.067008303 | 0.005035479 | 0.038175463 |
| ZBED2 | 5.793053986 | 2.53432411 | 0.005074715 | 0.038430238 |
| AKAP14 | 9.501301742 | 3.248125186 | 0.005083414 | 0.038479042 |
| GAD1 | 9.91908498 | 3.31020704 | 0.005111742 | 0.038623069 |
| SLC22A24 | 18.01107551 | 4.170812427 | 0.005113808 | 0.038623454 |
| SSX1 | 73.32606137 | 6.196254144 | 0.005140764 | 0.038775565 |
| LINC02263 | 17.29031833 | 4.111892525 | 0.005148173 | 0.038822865 |
| SLC4A1 | 0.234051678 | -2.095100984 | 0.005156373 | 0.038843117 |
| IGFBPL1 | 5.5653539 | 2.476473431 | 0.00515783 | 0.038843117 |
| FAM72A | 5.290466925 | 2.403395057 | 0.005165373 | 0.038875269 |
| ACSM2B | 0.205068749 | -2.285820442 | 0.005230874 | 0.039298913 |
| SERPINA9 | 6.265544922 | 2.647439988 | 0.005237459 | 0.039331071 |
| AC010894.3 | 7.350014341 | 2.877747065 | 0.005255075 | 0.039437329 |
| ILDR2 | 4.851290872 | 2.278368683 | 0.005260135 | 0.039452941 |
| KCNMB2-AS1 | 6.599295013 | 2.722311913 | 0.005260623 | 0.039452941 |
| JPH3 | 5.381954307 | 2.428130142 | 0.005264331 | 0.039463405 |
| OR7E62P | 8.070103652 | 3.012587204 | 0.005301444 | 0.039698023 |
| TCL6 | 6.536579939 | 2.708535988 | 0.005331061 | 0.039871136 |
| LHX9 | 12.5083286 | 3.64481712 | 0.005331571 | 0.039871136 |
| AL359697.1 | 7.259227969 | 2.859816123 | 0.005338662 | 0.039906675 |
| AC062028.1 | 6.185705889 | 2.628938239 | 0.005345093 | 0.03993725 |
| AC008780.1 | 0.221951384 | -2.17168439 | 0.005349277 | 0.03995977 |
| AP003071.3 | 0.226549265 | -2.142103283 | 0.005374924 | 0.040098698 |
| ADORA2BP1 | 0.243149889 | -2.04008216 | 0.005414801 | 0.04033448 |
| TUBB4A | 4.962999316 | 2.311212256 | 0.005419307 | 0.040350128 |
| DSCR4 | 45.62486192 | 5.511748287 | 0.005435252 | 0.040425057 |
| LINC02506 | 13.29480466 | 3.732790674 | 0.005460446 | 0.040585908 |
| MIR4713HG | 12.29211265 | 3.619660988 | 0.005491749 | 0.040756477 |
| CALHM1 | 7.417230746 | 2.890880651 | 0.005500312 | 0.040801338 |
| LRRC38 | 0.072439274 | -3.787084108 | 0.0055266 | 0.040970542 |
| AL356311.1 | 7.952589056 | 2.991424623 | 0.005548907 | 0.041109128 |
| ZNF716 | 9.066700593 | 3.180577644 | 0.005555855 | 0.041126396 |
| LINC01574 | 16.23898626 | 4.021389668 | 0.005556892 | 0.041126396 |
| AC120498.4 | 5.172667516 | 2.370908462 | 0.005561701 | 0.041141916 |
| SERPINB7 | 12.26080195 | 3.61598144 | 0.005561771 | 0.041141916 |
| KCNH6 | 15.59890071 | 3.963372458 | 0.005599962 | 0.041361676 |
| NAT16 | 10.28492645 | 3.362459572 | 0.005680976 | 0.041860406 |
| SMC1B | 4.950665975 | 2.307622613 | 0.005690915 | 0.04191594 |
| AL160408.1 | 7.475661915 | 2.902201325 | 0.005700951 | 0.041979904 |
| LINC02254 | 0.230632655 | -2.116331298 | 0.005743122 | 0.042218072 |
| ATXN8OS | 39.48390645 | 5.303192829 | 0.005772326 | 0.042396222 |
| AL033519.4 | 8.121913888 | 3.021819731 | 0.005812423 | 0.042617357 |
| AC135584.1 | 0.229959092 | -2.120550854 | 0.005817895 | 0.04264832 |
| SNX19P1 | 10.23297671 | 3.355153973 | 0.005892586 | 0.043158769 |
| ADGRF5P2 | 0.23501543 | -2.089172615 | 0.005897219 | 0.043174172 |
| AC123023.1 | 12.75544279 | 3.673041076 | 0.005943704 | 0.043449252 |
| AC013724.1 | 8.32636746 | 3.057687229 | 0.005947232 | 0.043465732 |
| GPR19 | 5.052130841 | 2.336892003 | 0.00596134 | 0.043550188 |
| LINC02388 | 0.239731455 | -2.060508876 | 0.005963717 | 0.043558237 |
| AGAP1-IT1 | 6.765516589 | 2.758200097 | 0.005983522 | 0.04367485 |
| MIR3150BHG | 6.756751701 | 2.756329839 | 0.006048478 | 0.044082984 |
| AC245100.3 | 9.646046037 | 3.269937696 | 0.006097455 | 0.044392541 |
| CDIPTOSP | 6.269408075 | 2.648329238 | 0.006113899 | 0.04447285 |
| BMP8A | 5.346206761 | 2.418515634 | 0.006116586 | 0.044474904 |
| SMKR1 | 4.004660649 | 2.001679995 | 0.006125789 | 0.04452285 |
| UNC93A | 11.18990268 | 3.484125584 | 0.0061397 | 0.044595469 |
| LRRC73 | 4.245506136 | 2.085936558 | 0.006168292 | 0.044774557 |
| SIX3-AS1 | 8.07832928 | 3.014056953 | 0.006192491 | 0.044902469 |
| OR4K12P | 6.723896411 | 2.749297498 | 0.006242306 | 0.045206249 |
| RPSAP65 | 23.08846789 | 4.529100536 | 0.00626131 | 0.045295627 |
| ACP7 | 7.019340005 | 2.811335387 | 0.006275845 | 0.045362318 |
| AL133387.1 | 6.851473532 | 2.776414299 | 0.006376961 | 0.045956941 |
| LINC00337 | 7.059392099 | 2.819543955 | 0.006401251 | 0.046063905 |
| TACC1P1 | 14.68931868 | 3.876695578 | 0.006418584 | 0.046149715 |
| SNTN | 7.002173216 | 2.807802751 | 0.006464283 | 0.046448936 |
| LINC00940 | 5.36154563 | 2.422648962 | 0.006522121 | 0.046825096 |
| LINC01505 | 7.820856757 | 2.96732666 | 0.00653956 | 0.046920801 |
| KLHL30-AS1 | 0.212312081 | -2.235741627 | 0.006554101 | 0.047003928 |
| AC107023.1 | 20.3342631 | 4.345840804 | 0.006555293 | 0.047003928 |
| HASPIN | 4.072840728 | 2.026035398 | 0.006575545 | 0.047103201 |
| GTF3AP6 | 51.11675333 | 5.675724302 | 0.006592622 | 0.047192275 |
| STXBP5L | 6.975916533 | 2.802382778 | 0.006622264 | 0.047374657 |
| FIBCD1 | 4.021151484 | 2.007608686 | 0.006651304 | 0.04753259 |
| CCDC92B | 11.18374383 | 3.483331315 | 0.006692601 | 0.047757716 |
| ASCL4 | 7.633993416 | 2.932437942 | 0.006738483 | 0.047994814 |
| CDH2 | 5.906940862 | 2.562411168 | 0.006740086 | 0.04799622 |
| AC104564.4 | 10.30601073 | 3.365414094 | 0.0067491 | 0.048030346 |
| GIMAP3P | 0.241568196 | -2.049497566 | 0.00679596 | 0.048343666 |
| ZBBX | 8.135347002 | 3.024203883 | 0.006824984 | 0.048489495 |
| AP005121.1 | 6.28974382 | 2.653001258 | 0.00683406 | 0.048528493 |
| CT45A1 | 108.1692457 | 6.757146565 | 0.006888447 | 0.048859015 |
| FAM72B | 5.048183062 | 2.335764228 | 0.006904483 | 0.048962584 |
| SPTB | 0.152863133 | -2.709687586 | 0.00698542 | 0.049423585 |
| FEZF1-AS1 | 7.545378948 | 2.915593358 | 0.006991072 | 0.049443071 |
| AC090358.1 | 19.41251706 | 4.278915287 | 0.007016714 | 0.049593595 |
| AC006357.1 | 11.24692844 | 3.491459147 | 0.007040872 | 0.049723156 |
| CPA6 | 4.040475216 | 2.014524984 | 0.007044774 | 0.049736528 |
| AP000251.1 | 6.054883374 | 2.598099172 | 0.007068896 | 0.049859161 |
