# Supplementary material for: A System Pharmacology Model for Decoding the Synergistic Mechanisms of Compound Kushen Injection in Treating Breast Cancer
Source: Front Pharmacol. 2021 Nov 16;12:723147. doi: 10.3389/fphar.2021.723147 (PMC8660088; doi:10.3389/fphar.2021.723147)
Supplement: Supplementary file 8 [file Table5.DOCX]

**Table S5 |** The targets information of *Radix Sophorae Flavescentis* and *Rhizoma Heterosmilacis* obtained from the online tools

| **Herb** | **Target** | **Herb** | **Target** | **Herb** | **Target** |
| --- | --- | --- | --- | --- | --- |
| *Rhizoma Heterosmilacis* | ATP12A | *Radix Sophorae Flavescentis* | FGR | *Radix Sophorae Flavescentis* | NRAS |
| *Rhizoma Heterosmilacis* | ATP1A1 | *Radix Sophorae Flavescentis* | FRK | *Radix Sophorae Flavescentis* | SLC28A3 |
| *Rhizoma Heterosmilacis* | EBP | *Radix Sophorae Flavescentis* | FYN | *Radix Sophorae Flavescentis* | ADORA2B |
| *Rhizoma Heterosmilacis* | ERG2 | *Radix Sophorae Flavescentis* | GABRG1 | *Radix Sophorae Flavescentis* | LCTL |
| *Rhizoma Heterosmilacis* | G6PD | *Radix Sophorae Flavescentis* | LHCGR | *Radix Sophorae Flavescentis* | NACHRALPHA5 |
| *Rhizoma Heterosmilacis* | GABBR1 | *Radix Sophorae Flavescentis* | O75342 | *Radix Sophorae Flavescentis* | BRPF3 |
| *Rhizoma Heterosmilacis* | GABRA4 | *Radix Sophorae Flavescentis* | YES1 | *Radix Sophorae Flavescentis* | KAT2A |
| *Rhizoma Heterosmilacis* | GABRB2 | *Radix Sophorae Flavescentis* | ALDH1A2 | *Radix Sophorae Flavescentis* | KAT2B |
| *Rhizoma Heterosmilacis* | GABRD | *Radix Sophorae Flavescentis* | ALDH1B1 | *Radix Sophorae Flavescentis & Rhizoma Heterosmilacis* | ABCB1 |
| *Rhizoma Heterosmilacis* | GRIN1 | *Radix Sophorae Flavescentis* | ERBB3 | *Radix Sophorae Flavescentis & Rhizoma Heterosmilacis* | ABCB1A |
| *Rhizoma Heterosmilacis* | GRIN2B | *Radix Sophorae Flavescentis* | ERBB4 | *Radix Sophorae Flavescentis & Rhizoma Heterosmilacis* | ABCC1 |
| *Rhizoma Heterosmilacis* | GRIN2C | *Radix Sophorae Flavescentis* | ESRRG | *Radix Sophorae Flavescentis & Rhizoma Heterosmilacis* | ABCC2 |
| *Rhizoma Heterosmilacis* | GRIN2D | *Radix Sophorae Flavescentis* | NCOA1 | *Radix Sophorae Flavescentis & Rhizoma Heterosmilacis* | ABCG2 |
| *Rhizoma Heterosmilacis* | NR1I3 | *Radix Sophorae Flavescentis* | NCOA2 | *Radix Sophorae Flavescentis & Rhizoma Heterosmilacis* | ACP1 |
| *Rhizoma Heterosmilacis* | SERPINA6 | *Radix Sophorae Flavescentis* | TRPV2 | *Radix Sophorae Flavescentis & Rhizoma Heterosmilacis* | ADORA1 |
| *Rhizoma Heterosmilacis* | SLCO1A1 | *Radix Sophorae Flavescentis* | CHRFAM7A | *Radix Sophorae Flavescentis & Rhizoma Heterosmilacis* | ADORA2A |
| *Rhizoma Heterosmilacis* | SMT1 | *Radix Sophorae Flavescentis* | CHRNA1 | *Radix Sophorae Flavescentis & Rhizoma Heterosmilacis* | AKR1A1 |
| *Rhizoma Heterosmilacis* | ST3GAL1 | *Radix Sophorae Flavescentis* | CHRNA2 | *Radix Sophorae Flavescentis & Rhizoma Heterosmilacis* | AKR1B1 |
| *Rhizoma Heterosmilacis* | ST3GAL2 | *Radix Sophorae Flavescentis* | CHRNA3 | *Radix Sophorae Flavescentis & Rhizoma Heterosmilacis* | AKR1B10 |
| *Rhizoma Heterosmilacis* | UGT1A1 | *Radix Sophorae Flavescentis* | CHRNA5 | *Radix Sophorae Flavescentis & Rhizoma Heterosmilacis* | AKR1C21 |
| *Rhizoma Heterosmilacis* | UGT1A10 | *Radix Sophorae Flavescentis* | CHRNA6 | *Radix Sophorae Flavescentis & Rhizoma Heterosmilacis* | AKT1 |
| *Rhizoma Heterosmilacis* | UGT1A3 | *Radix Sophorae Flavescentis* | CHRNA7 | *Radix Sophorae Flavescentis & Rhizoma Heterosmilacis* | ALK |
| *Rhizoma Heterosmilacis* | UGT1A4 | *Radix Sophorae Flavescentis* | CHRNB4 | *Radix Sophorae Flavescentis & Rhizoma Heterosmilacis* | ALOX12 |
| *Rhizoma Heterosmilacis* | UGT1A5 | *Radix Sophorae Flavescentis* | L3MBTL3 | *Radix Sophorae Flavescentis & Rhizoma Heterosmilacis* | ALOX15 |
| *Rhizoma Heterosmilacis* | UGT1A6 | *Radix Sophorae Flavescentis* | HSD11B1 | *Radix Sophorae Flavescentis & Rhizoma Heterosmilacis* | ALOX15B |
| *Rhizoma Heterosmilacis* | UGT1A7 | *Radix Sophorae Flavescentis* | HSD11B1L | *Radix Sophorae Flavescentis & Rhizoma Heterosmilacis* | ALOX5 |
| *Rhizoma Heterosmilacis* | UGT1A8 | *Radix Sophorae Flavescentis* | MBNL1 | *Radix Sophorae Flavescentis & Rhizoma Heterosmilacis* | ALPI |
| *Rhizoma Heterosmilacis* | UGT1A9 | *Radix Sophorae Flavescentis* | MBNL2 | *Radix Sophorae Flavescentis & Rhizoma Heterosmilacis* | AMPC |
| *Rhizoma Heterosmilacis* | KIT | *Radix Sophorae Flavescentis* | MBNL3 | *Radix Sophorae Flavescentis & Rhizoma Heterosmilacis* | AMY1A |
| *Rhizoma Heterosmilacis* | ACAT | *Radix Sophorae Flavescentis* | BETA-TUBULIN | *Radix Sophorae Flavescentis & Rhizoma Heterosmilacis* | AMY1B |
| *Rhizoma Heterosmilacis* | ACER2 | *Radix Sophorae Flavescentis* | CAN2 | *Radix Sophorae Flavescentis & Rhizoma Heterosmilacis* | AMY1C |
| *Rhizoma Heterosmilacis* | ADH1A | *Radix Sophorae Flavescentis* | CYP2C19 | *Radix Sophorae Flavescentis & Rhizoma Heterosmilacis* | APEX1 |
| *Rhizoma Heterosmilacis* | ADH1B | *Radix Sophorae Flavescentis* | FABH | *Radix Sophorae Flavescentis & Rhizoma Heterosmilacis* | ARG1 |
| *Rhizoma Heterosmilacis* | ADH1C | *Radix Sophorae Flavescentis* | FADS1 | *Radix Sophorae Flavescentis & Rhizoma Heterosmilacis* | ATP5A1 |
| *Rhizoma Heterosmilacis* | ADH7 | *Radix Sophorae Flavescentis* | MTTP | *Radix Sophorae Flavescentis & Rhizoma Heterosmilacis* | ATP5B |
| *Rhizoma Heterosmilacis* | AGRA | *Radix Sophorae Flavescentis* | SLC6A11 | *Radix Sophorae Flavescentis & Rhizoma Heterosmilacis* | ATP5C1 |
| *Rhizoma Heterosmilacis* | ASAH1 | *Radix Sophorae Flavescentis* | SLC6A13 | *Radix Sophorae Flavescentis & Rhizoma Heterosmilacis* | AURKB |
| *Rhizoma Heterosmilacis* | ATG4B | *Radix Sophorae Flavescentis* | SOAT1 | *Radix Sophorae Flavescentis & Rhizoma Heterosmilacis* | AVPR2 |
| *Rhizoma Heterosmilacis* | CAH1 | *Radix Sophorae Flavescentis* | TUBB1 | *Radix Sophorae Flavescentis & Rhizoma Heterosmilacis* | AXL |
| *Rhizoma Heterosmilacis* | CAH2 | *Radix Sophorae Flavescentis* | TUBB3 | *Radix Sophorae Flavescentis & Rhizoma Heterosmilacis* | BACE1 |
| *Rhizoma Heterosmilacis* | CDC25B | *Radix Sophorae Flavescentis* | CDK2 | *Radix Sophorae Flavescentis & Rhizoma Heterosmilacis* | CA1 |
| *Rhizoma Heterosmilacis* | CES1 | *Radix Sophorae Flavescentis* | CDK4 | *Radix Sophorae Flavescentis & Rhizoma Heterosmilacis* | CA12 |
| *Rhizoma Heterosmilacis* | CES2C | *Radix Sophorae Flavescentis* | CDK5R1 | *Radix Sophorae Flavescentis & Rhizoma Heterosmilacis* | CA13 |
| *Rhizoma Heterosmilacis* | CES3 | *Radix Sophorae Flavescentis* | CDK6 | *Radix Sophorae Flavescentis & Rhizoma Heterosmilacis* | CA14 |
| *Rhizoma Heterosmilacis* | CES5A | *Radix Sophorae Flavescentis* | CFTR | *Radix Sophorae Flavescentis & Rhizoma Heterosmilacis* | CA2 |
| *Rhizoma Heterosmilacis* | COL4A3BP | *Radix Sophorae Flavescentis* | ERAP1 | *Radix Sophorae Flavescentis & Rhizoma Heterosmilacis* | CA3 |
| *Rhizoma Heterosmilacis* | CPT1 | *Radix Sophorae Flavescentis* | MIF | *Radix Sophorae Flavescentis & Rhizoma Heterosmilacis* | CA4 |
| *Rhizoma Heterosmilacis* | CPT1B | *Radix Sophorae Flavescentis* | ALPHA-MAN-IA | *Radix Sophorae Flavescentis & Rhizoma Heterosmilacis* | CA5A |
| *Rhizoma Heterosmilacis* | CQSS | *Radix Sophorae Flavescentis* | HSD17B12 | *Radix Sophorae Flavescentis & Rhizoma Heterosmilacis* | CA6 |
| *Rhizoma Heterosmilacis* | CYP4F2 | *Radix Sophorae Flavescentis* | MAN1 | *Radix Sophorae Flavescentis & Rhizoma Heterosmilacis* | CA7 |
| *Rhizoma Heterosmilacis* | DAGLA | *Radix Sophorae Flavescentis* | MAN2A1 | *Radix Sophorae Flavescentis & Rhizoma Heterosmilacis* | CA9 |
| *Rhizoma Heterosmilacis* | DNM1 | *Radix Sophorae Flavescentis* | MAN2B1 | *Radix Sophorae Flavescentis & Rhizoma Heterosmilacis* | CAMK2B |
| *Rhizoma Heterosmilacis* | DNTT | *Radix Sophorae Flavescentis* | MAN2C1 | *Radix Sophorae Flavescentis & Rhizoma Heterosmilacis* | CBR1 |
| *Rhizoma Heterosmilacis* | DXS | *Radix Sophorae Flavescentis* | ADRA1A | *Radix Sophorae Flavescentis & Rhizoma Heterosmilacis* | CCR4 |
| *Rhizoma Heterosmilacis* | ECR | *Radix Sophorae Flavescentis* | ADRA1B | *Radix Sophorae Flavescentis & Rhizoma Heterosmilacis* | CDK1 |
| *Rhizoma Heterosmilacis* | ENPP2 | *Radix Sophorae Flavescentis* | ADRA1D | *Radix Sophorae Flavescentis & Rhizoma Heterosmilacis* | CISD1 |
| *Rhizoma Heterosmilacis* | EPHX1 | *Radix Sophorae Flavescentis* | ADRA2A | *Radix Sophorae Flavescentis & Rhizoma Heterosmilacis* | CREB1 |
| *Rhizoma Heterosmilacis* | EST1 | *Radix Sophorae Flavescentis* | ADRA2B | *Radix Sophorae Flavescentis & Rhizoma Heterosmilacis* | CSNK2A1 |
| *Rhizoma Heterosmilacis* | FABP3 | *Radix Sophorae Flavescentis* | ADRA2C | *Radix Sophorae Flavescentis & Rhizoma Heterosmilacis* | CXCR1 |
| *Rhizoma Heterosmilacis* | FBA1 | *Radix Sophorae Flavescentis* | HRH1 | *Radix Sophorae Flavescentis & Rhizoma Heterosmilacis* | CYP19A1 |
| *Rhizoma Heterosmilacis* | FBPC | *Radix Sophorae Flavescentis* | HTR1A | *Radix Sophorae Flavescentis & Rhizoma Heterosmilacis* | CYP1A2 |
| *Rhizoma Heterosmilacis* | FDPS | *Radix Sophorae Flavescentis* | HTR1B | *Radix Sophorae Flavescentis & Rhizoma Heterosmilacis* | CYP1B1 |
| *Rhizoma Heterosmilacis* | FFAR4 | *Radix Sophorae Flavescentis* | NR3C1 | *Radix Sophorae Flavescentis & Rhizoma Heterosmilacis* | CYP2C8 |
| *Rhizoma Heterosmilacis* | FPPS | *Radix Sophorae Flavescentis* | L3MBTL1 | *Radix Sophorae Flavescentis & Rhizoma Heterosmilacis* | CYP2C9 |
| *Rhizoma Heterosmilacis* | GGPS1 | *Radix Sophorae Flavescentis* | DPP4 | *Radix Sophorae Flavescentis & Rhizoma Heterosmilacis* | DAPK1 |
| *Rhizoma Heterosmilacis* | GNAI1 | *Radix Sophorae Flavescentis* | FAP | *Radix Sophorae Flavescentis & Rhizoma Heterosmilacis* | DDL |
| *Rhizoma Heterosmilacis* | GNAI3 | *Radix Sophorae Flavescentis* | TRHR | *Radix Sophorae Flavescentis & Rhizoma Heterosmilacis* | DRD4 |
| *Rhizoma Heterosmilacis* | GNAO1 | *Radix Sophorae Flavescentis* | ABCB4 | *Radix Sophorae Flavescentis & Rhizoma Heterosmilacis* | DYRK1A |
| *Rhizoma Heterosmilacis* | GPR174 | *Radix Sophorae Flavescentis* | ALDH1A3 | *Radix Sophorae Flavescentis & Rhizoma Heterosmilacis* | E6 |
| *Rhizoma Heterosmilacis* | GPR34 | *Radix Sophorae Flavescentis* | GPR84 | *Radix Sophorae Flavescentis & Rhizoma Heterosmilacis* | EGFR |
| *Rhizoma Heterosmilacis* | GSTK1 | *Radix Sophorae Flavescentis* | KASA | *Radix Sophorae Flavescentis & Rhizoma Heterosmilacis* | ELAVL1 |
| *Rhizoma Heterosmilacis* | HAO1 | *Radix Sophorae Flavescentis* | MCL1 | *Radix Sophorae Flavescentis & Rhizoma Heterosmilacis* | ERBB2 |
| *Rhizoma Heterosmilacis* | HDAH | *Radix Sophorae Flavescentis* | MT-CYB | *Radix Sophorae Flavescentis & Rhizoma Heterosmilacis* | ESR1 |
| *Rhizoma Heterosmilacis* | HK | *Radix Sophorae Flavescentis* | MVFR | *Radix Sophorae Flavescentis & Rhizoma Heterosmilacis* | ESR2 |
| *Rhizoma Heterosmilacis* | HMGCR | *Radix Sophorae Flavescentis* | TLR1 | *Radix Sophorae Flavescentis & Rhizoma Heterosmilacis* | ESRRA |
| *Rhizoma Heterosmilacis* | HPD | *Radix Sophorae Flavescentis* | UNG | *Radix Sophorae Flavescentis & Rhizoma Heterosmilacis* | ESRRB |
| *Rhizoma Heterosmilacis* | IL6ST | *Radix Sophorae Flavescentis* | UQCR11 | *Radix Sophorae Flavescentis & Rhizoma Heterosmilacis* | F2 |
| *Rhizoma Heterosmilacis* | JHE | *Radix Sophorae Flavescentis* | ARSD | *Radix Sophorae Flavescentis & Rhizoma Heterosmilacis* | FABG |
| *Rhizoma Heterosmilacis* | KDM5A | *Radix Sophorae Flavescentis* | ARSE | *Radix Sophorae Flavescentis & Rhizoma Heterosmilacis* | FABI |
| *Rhizoma Heterosmilacis* | LAP3 | *Radix Sophorae Flavescentis* | ARSF | *Radix Sophorae Flavescentis & Rhizoma Heterosmilacis* | FABZ |
| *Rhizoma Heterosmilacis* | LASR | *Radix Sophorae Flavescentis* | ARSH | *Radix Sophorae Flavescentis & Rhizoma Heterosmilacis* | FASN |
| *Rhizoma Heterosmilacis* | LPAR1 | *Radix Sophorae Flavescentis* | BAD | *Radix Sophorae Flavescentis & Rhizoma Heterosmilacis* | FLT3 |
| *Rhizoma Heterosmilacis* | LPAR2 | *Radix Sophorae Flavescentis* | STS | *Radix Sophorae Flavescentis & Rhizoma Heterosmilacis* | GLO1 |
| *Rhizoma Heterosmilacis* | LPAR3 | *Radix Sophorae Flavescentis* | P42336 | *Radix Sophorae Flavescentis & Rhizoma Heterosmilacis* | GPR35 |
| *Rhizoma Heterosmilacis* | LPAR4 | *Radix Sophorae Flavescentis* | P48736 | *Radix Sophorae Flavescentis & Rhizoma Heterosmilacis* | GRK6 |
| *Rhizoma Heterosmilacis* | LPAR5 | *Radix Sophorae Flavescentis* | O00329 | *Radix Sophorae Flavescentis & Rhizoma Heterosmilacis* | GSK3B |
| *Rhizoma Heterosmilacis* | LPAR6 | *Radix Sophorae Flavescentis* | CELA1 | *Radix Sophorae Flavescentis & Rhizoma Heterosmilacis* | GUSB |
| *Rhizoma Heterosmilacis* | LPXC | *Radix Sophorae Flavescentis* | CYP11B1 | *Radix Sophorae Flavescentis & Rhizoma Heterosmilacis* | HCK |
| *Rhizoma Heterosmilacis* | LSS | *Radix Sophorae Flavescentis* | CYP11B2 | *Radix Sophorae Flavescentis & Rhizoma Heterosmilacis* | HSD17B1 |
| *Rhizoma Heterosmilacis* | LUXR | *Radix Sophorae Flavescentis* | EGLN1 | *Radix Sophorae Flavescentis & Rhizoma Heterosmilacis* | HSD17B2 |
| *Rhizoma Heterosmilacis* | MGLL | *Radix Sophorae Flavescentis* | ERCC5 | *Radix Sophorae Flavescentis & Rhizoma Heterosmilacis* | IAP |
| *Rhizoma Heterosmilacis* | MPEG1 | *Radix Sophorae Flavescentis* | FBA | *Radix Sophorae Flavescentis & Rhizoma Heterosmilacis* | IGF1R |
| *Rhizoma Heterosmilacis* | MSR1 | *Radix Sophorae Flavescentis* | FEN1 | *Radix Sophorae Flavescentis & Rhizoma Heterosmilacis* | IL2 |
| *Rhizoma Heterosmilacis* | MT-ND1 | *Radix Sophorae Flavescentis* | GABRA3 | *Radix Sophorae Flavescentis & Rhizoma Heterosmilacis* | IL5 |
| *Rhizoma Heterosmilacis* | MURF | *Radix Sophorae Flavescentis* | GABRB3 | *Radix Sophorae Flavescentis & Rhizoma Heterosmilacis* | KCND3 |
| *Rhizoma Heterosmilacis* | NAAA | *Radix Sophorae Flavescentis* | GABRG2 | *Radix Sophorae Flavescentis & Rhizoma Heterosmilacis* | KDM4E |
| *Rhizoma Heterosmilacis* | NDUFAB1 | *Radix Sophorae Flavescentis* | GRM7 | *Radix Sophorae Flavescentis & Rhizoma Heterosmilacis* | KDR |
| *Rhizoma Heterosmilacis* | NDUFV1 | *Radix Sophorae Flavescentis* | HNF4A | *Radix Sophorae Flavescentis & Rhizoma Heterosmilacis* | LOX1.1 |
| *Rhizoma Heterosmilacis* | NOD1 | *Radix Sophorae Flavescentis* | HSC82 | *Radix Sophorae Flavescentis & Rhizoma Heterosmilacis* | LPO |
| *Rhizoma Heterosmilacis* | OXER1 | *Radix Sophorae Flavescentis* | KLK7 | *Radix Sophorae Flavescentis & Rhizoma Heterosmilacis* | LYZ1 |
| *Rhizoma Heterosmilacis* | P2RY10 | *Radix Sophorae Flavescentis* | MMP26 | *Radix Sophorae Flavescentis & Rhizoma Heterosmilacis* | MAOA |
| *Rhizoma Heterosmilacis* | PAOX | *Radix Sophorae Flavescentis* | MTNR1A | *Radix Sophorae Flavescentis & Rhizoma Heterosmilacis* | MAPT |
| *Rhizoma Heterosmilacis* | PEPN | *Radix Sophorae Flavescentis* | MTNR1B | *Radix Sophorae Flavescentis & Rhizoma Heterosmilacis* | MDH |
| *Rhizoma Heterosmilacis* | PHZR | *Radix Sophorae Flavescentis* | NQO1 | *Radix Sophorae Flavescentis & Rhizoma Heterosmilacis* | MET |
| *Rhizoma Heterosmilacis* | PLA2G10 | *Radix Sophorae Flavescentis* | NR4A2 | *Radix Sophorae Flavescentis & Rhizoma Heterosmilacis* | MMP13 |
| *Rhizoma Heterosmilacis* | PLA2G2C | *Radix Sophorae Flavescentis* | PDK3 | *Radix Sophorae Flavescentis & Rhizoma Heterosmilacis* | MMP2 |
| *Rhizoma Heterosmilacis* | PLA2G4A | *Radix Sophorae Flavescentis* | PDK4 | *Radix Sophorae Flavescentis & Rhizoma Heterosmilacis* | MMP3 |
| *Rhizoma Heterosmilacis* | PLA2G4B | *Radix Sophorae Flavescentis* | RCE1 | *Radix Sophorae Flavescentis & Rhizoma Heterosmilacis* | MMP9 |
| *Rhizoma Heterosmilacis* | PLA2G4C | *Radix Sophorae Flavescentis* | SENP7 | *Radix Sophorae Flavescentis & Rhizoma Heterosmilacis* | MPG |
| *Rhizoma Heterosmilacis* | PLB1 | *Radix Sophorae Flavescentis* | SHC | *Radix Sophorae Flavescentis & Rhizoma Heterosmilacis* | MPO |
| *Rhizoma Heterosmilacis* | PLC | *Radix Sophorae Flavescentis* | SLC16A3 | *Radix Sophorae Flavescentis & Rhizoma Heterosmilacis* | NANH |
| *Rhizoma Heterosmilacis* | POLH | *Radix Sophorae Flavescentis* | TOP2B | *Radix Sophorae Flavescentis & Rhizoma Heterosmilacis* | NEK2 |
| *Rhizoma Heterosmilacis* | POLI | *Radix Sophorae Flavescentis* | TPPP | *Radix Sophorae Flavescentis & Rhizoma Heterosmilacis* | NEK6 |
| *Rhizoma Heterosmilacis* | POLK | *Radix Sophorae Flavescentis* | TRPM7 | *Radix Sophorae Flavescentis & Rhizoma Heterosmilacis* | NMUR2 |
| *Rhizoma Heterosmilacis* | POLM | *Radix Sophorae Flavescentis* | UL26 | *Radix Sophorae Flavescentis & Rhizoma Heterosmilacis* | NOX4 |
| *Rhizoma Heterosmilacis* | PTGER2 | *Radix Sophorae Flavescentis* | CTSD | *Radix Sophorae Flavescentis & Rhizoma Heterosmilacis* | NT5E |
| *Rhizoma Heterosmilacis* | RARB | *Radix Sophorae Flavescentis* | NAPSA | *Radix Sophorae Flavescentis & Rhizoma Heterosmilacis* | NTBBF1.1 |
| *Rhizoma Heterosmilacis* | S1PR2 | *Radix Sophorae Flavescentis* | REN | *Radix Sophorae Flavescentis & Rhizoma Heterosmilacis* | NUAK1 |
| *Rhizoma Heterosmilacis* | S1PR3 | *Radix Sophorae Flavescentis* | PTGER1 | *Radix Sophorae Flavescentis & Rhizoma Heterosmilacis* | OPRD1 |
| *Rhizoma Heterosmilacis* | S1PR4 | *Radix Sophorae Flavescentis* | NFKB1 | *Radix Sophorae Flavescentis & Rhizoma Heterosmilacis* | P4HB |
| *Rhizoma Heterosmilacis* | S1PR5 | *Radix Sophorae Flavescentis* | PDE5A | *Radix Sophorae Flavescentis & Rhizoma Heterosmilacis* | PFMRK |
| *Rhizoma Heterosmilacis* | SI | *Radix Sophorae Flavescentis* | IMPDH1 | *Radix Sophorae Flavescentis & Rhizoma Heterosmilacis* | PGF |
| *Rhizoma Heterosmilacis* | SLC22A1 | *Radix Sophorae Flavescentis* | IMPDH2 | *Radix Sophorae Flavescentis & Rhizoma Heterosmilacis* | PIK3CG |
| *Rhizoma Heterosmilacis* | SLC22A2 | *Radix Sophorae Flavescentis* | PLAA | *Radix Sophorae Flavescentis & Rhizoma Heterosmilacis* | PIK3R1 |
| *Rhizoma Heterosmilacis* | SLC22A20 | *Radix Sophorae Flavescentis* | SLC5A10 | *Radix Sophorae Flavescentis & Rhizoma Heterosmilacis* | PIM1 |
| *Rhizoma Heterosmilacis* | SLC22A8 | *Radix Sophorae Flavescentis* | SLC5A3 | *Radix Sophorae Flavescentis & Rhizoma Heterosmilacis* | PKN1 |
| *Rhizoma Heterosmilacis* | SLC25A20 | *Radix Sophorae Flavescentis* | SLC5A4 | *Radix Sophorae Flavescentis & Rhizoma Heterosmilacis* | PLA2G1B |
| *Rhizoma Heterosmilacis* | SLC6A8 | *Radix Sophorae Flavescentis* | SLC5A9 | *Radix Sophorae Flavescentis & Rhizoma Heterosmilacis* | POL |
| *Rhizoma Heterosmilacis* | SPHK1 | *Radix Sophorae Flavescentis* | SLC18A3 | *Radix Sophorae Flavescentis & Rhizoma Heterosmilacis* | POLA |
| *Rhizoma Heterosmilacis* | SPHK2 | *Radix Sophorae Flavescentis* | FLT4 | *Radix Sophorae Flavescentis & Rhizoma Heterosmilacis* | PON1 |
| *Rhizoma Heterosmilacis* | SPPS | *Radix Sophorae Flavescentis* | GLRA1 | *Radix Sophorae Flavescentis & Rhizoma Heterosmilacis* | PRSS1 |
| *Rhizoma Heterosmilacis* | THRA | *Radix Sophorae Flavescentis* | GPR18 | *Radix Sophorae Flavescentis & Rhizoma Heterosmilacis* | PTK2 |
| *Rhizoma Heterosmilacis* | THRB | *Radix Sophorae Flavescentis* | KIF20B | *Radix Sophorae Flavescentis & Rhizoma Heterosmilacis* | PTPRS |
| *Rhizoma Heterosmilacis* | TRAR | *Radix Sophorae Flavescentis* | LDHB | *Radix Sophorae Flavescentis & Rhizoma Heterosmilacis* | PYGL |
| *Rhizoma Heterosmilacis* | TRPV1 | *Radix Sophorae Flavescentis* | LTB4R | *Radix Sophorae Flavescentis & Rhizoma Heterosmilacis* | QDPR |
| *Rhizoma Heterosmilacis* | ALPL | *Radix Sophorae Flavescentis* | LTB4R2 | *Radix Sophorae Flavescentis & Rhizoma Heterosmilacis* | REP |
| *Rhizoma Heterosmilacis* | ARPP19 | *Radix Sophorae Flavescentis* | PTGER4 | *Radix Sophorae Flavescentis & Rhizoma Heterosmilacis* | REST |
| *Rhizoma Heterosmilacis* | NOS3 | *Radix Sophorae Flavescentis* | SERPINE1 | *Radix Sophorae Flavescentis & Rhizoma Heterosmilacis* | SLC16A1 |
| *Rhizoma Heterosmilacis* | RECA | *Radix Sophorae Flavescentis* | SERPINE2 | *Radix Sophorae Flavescentis & Rhizoma Heterosmilacis* | SLC16A7 |
| *Rhizoma Heterosmilacis* | FNTA | *Radix Sophorae Flavescentis* | TRPA1 | *Radix Sophorae Flavescentis & Rhizoma Heterosmilacis* | SLCO2B1 |
| *Rhizoma Heterosmilacis* | FNTB | *Radix Sophorae Flavescentis* | CTSK | *Radix Sophorae Flavescentis & Rhizoma Heterosmilacis* | SNCA |
| *Rhizoma Heterosmilacis* | MYB | *Radix Sophorae Flavescentis* | CTSL1 | *Radix Sophorae Flavescentis & Rhizoma Heterosmilacis* | ST3GAL3 |
| *Rhizoma Heterosmilacis* | PGGT1B | *Radix Sophorae Flavescentis* | CTSL2 | *Radix Sophorae Flavescentis & Rhizoma Heterosmilacis* | ST6GAL1 |
| *Rhizoma Heterosmilacis* | REL | *Radix Sophorae Flavescentis* | CTSS | *Radix Sophorae Flavescentis & Rhizoma Heterosmilacis* | SYN1 |
| *Rhizoma Heterosmilacis* | DBI | *Radix Sophorae Flavescentis* | CDK3 | *Radix Sophorae Flavescentis & Rhizoma Heterosmilacis* | TERT |
| *Rhizoma Heterosmilacis* | DPEP1 | *Radix Sophorae Flavescentis* | FBP1 | *Radix Sophorae Flavescentis & Rhizoma Heterosmilacis* | TOP1 |
| *Rhizoma Heterosmilacis* | NAT2 | *Radix Sophorae Flavescentis* | PTGES | *Radix Sophorae Flavescentis & Rhizoma Heterosmilacis* | TOP2A |
| *Rhizoma Heterosmilacis* | PAFAH1B2 | *Radix Sophorae Flavescentis* | RDL | *Radix Sophorae Flavescentis & Rhizoma Heterosmilacis* | TTR |
| *Rhizoma Heterosmilacis* | PRKACA | *Radix Sophorae Flavescentis* | TCF4 | *Radix Sophorae Flavescentis & Rhizoma Heterosmilacis* | TYR |
| *Rhizoma Heterosmilacis* | RHO | *Radix Sophorae Flavescentis* | 17HSDCL | *Radix Sophorae Flavescentis & Rhizoma Heterosmilacis* | VEGFA |
| *Rhizoma Heterosmilacis* | ABCC4 | *Radix Sophorae Flavescentis* | AKR1B15 | *Radix Sophorae Flavescentis & Rhizoma Heterosmilacis* | V-FPS |
| *Rhizoma Heterosmilacis* | AMY2A | *Radix Sophorae Flavescentis* | AKR1C3 | *Radix Sophorae Flavescentis & Rhizoma Heterosmilacis* | XDH |
| *Rhizoma Heterosmilacis* | BCL2L2 | *Radix Sophorae Flavescentis* | AKR1C4 | *Radix Sophorae Flavescentis & Rhizoma Heterosmilacis* | CHRM1 |
| *Rhizoma Heterosmilacis* | CXCR2 | *Radix Sophorae Flavescentis* | CNA1 | *Radix Sophorae Flavescentis & Rhizoma Heterosmilacis* | CHRM2 |
| *Rhizoma Heterosmilacis* | CYP11A1 | *Radix Sophorae Flavescentis* | CYSLTR1 | *Radix Sophorae Flavescentis & Rhizoma Heterosmilacis* | CHRM3 |
| *Rhizoma Heterosmilacis* | CYP125 | *Radix Sophorae Flavescentis* | FOS | *Radix Sophorae Flavescentis & Rhizoma Heterosmilacis* | CHRM4 |
| *Rhizoma Heterosmilacis* | CYP17A1 | *Radix Sophorae Flavescentis* | GAG-POL | *Radix Sophorae Flavescentis & Rhizoma Heterosmilacis* | CHRM5 |
| *Rhizoma Heterosmilacis* | DHCR7 | *Radix Sophorae Flavescentis* | HD1B | *Radix Sophorae Flavescentis & Rhizoma Heterosmilacis* | CYP51A1 |
| *Rhizoma Heterosmilacis* | EPHA2 | *Radix Sophorae Flavescentis* | HDA106 | *Radix Sophorae Flavescentis & Rhizoma Heterosmilacis* | EIF4A1 |
| *Rhizoma Heterosmilacis* | EPHA5 | *Radix Sophorae Flavescentis* | HDAC8 | *Radix Sophorae Flavescentis & Rhizoma Heterosmilacis* | GPBAR1 |
| *Rhizoma Heterosmilacis* | EPHA7 | *Radix Sophorae Flavescentis* | HSPD1 | *Radix Sophorae Flavescentis & Rhizoma Heterosmilacis* | HSD17B3 |
| *Rhizoma Heterosmilacis* | GBA2 | *Radix Sophorae Flavescentis* | ICMT | *Radix Sophorae Flavescentis & Rhizoma Heterosmilacis* | POLB |
| *Rhizoma Heterosmilacis* | HTR2A | *Radix Sophorae Flavescentis* | KCNA3 | *Radix Sophorae Flavescentis & Rhizoma Heterosmilacis* | SHBG |
| *Rhizoma Heterosmilacis* | NR1H3 | *Radix Sophorae Flavescentis* | NFE2L2 | *Radix Sophorae Flavescentis & Rhizoma Heterosmilacis* | SIGMAR1 |
| *Rhizoma Heterosmilacis* | PTAFR | *Radix Sophorae Flavescentis* | NOS2 | *Radix Sophorae Flavescentis & Rhizoma Heterosmilacis* | ABCC3 |
| *Rhizoma Heterosmilacis* | RORA | *Radix Sophorae Flavescentis* | NR0B2 | *Radix Sophorae Flavescentis & Rhizoma Heterosmilacis* | ACHE |
| *Rhizoma Heterosmilacis* | SGK1 | *Radix Sophorae Flavescentis* | NS3 | *Radix Sophorae Flavescentis & Rhizoma Heterosmilacis* | CBR3 |
| *Rhizoma Heterosmilacis* | SLC10A1 | *Radix Sophorae Flavescentis* | P29474 | *Radix Sophorae Flavescentis & Rhizoma Heterosmilacis* | CYP1A1 |
| *Rhizoma Heterosmilacis* | SREBF2 | *Radix Sophorae Flavescentis* | P29475 | *Radix Sophorae Flavescentis & Rhizoma Heterosmilacis* | CYP3A4 |
| *Rhizoma Heterosmilacis* | STAT1 | *Radix Sophorae Flavescentis* | P2RX1 | *Radix Sophorae Flavescentis & Rhizoma Heterosmilacis* | FUT4 |
| *Rhizoma Heterosmilacis* | STAT2 | *Radix Sophorae Flavescentis* | P35228 | *Radix Sophorae Flavescentis & Rhizoma Heterosmilacis* | FUT7 |
| *Rhizoma Heterosmilacis* | STAT3 | *Radix Sophorae Flavescentis* | PLA2G2E | *Radix Sophorae Flavescentis & Rhizoma Heterosmilacis* | KLK2 |
| *Rhizoma Heterosmilacis* | STAT4 | *Radix Sophorae Flavescentis* | PTPA | *Radix Sophorae Flavescentis & Rhizoma Heterosmilacis* | MAOB |
| *Rhizoma Heterosmilacis* | UGCG | *Radix Sophorae Flavescentis* | RBBP9 | *Radix Sophorae Flavescentis & Rhizoma Heterosmilacis* | NR3C2 |
| *Rhizoma Heterosmilacis* | VDR | *Radix Sophorae Flavescentis* | RMTA | *Radix Sophorae Flavescentis & Rhizoma Heterosmilacis* | ODC1 |
| *Rhizoma Heterosmilacis* | ABCB11 | *Radix Sophorae Flavescentis* | SENP6 | *Radix Sophorae Flavescentis & Rhizoma Heterosmilacis* | PGD |
| *Rhizoma Heterosmilacis* | ATIC | *Radix Sophorae Flavescentis* | STXA | *Radix Sophorae Flavescentis & Rhizoma Heterosmilacis* | PLA2G2A |
| *Rhizoma Heterosmilacis* | CD4 | *Radix Sophorae Flavescentis* | TLR4 | *Radix Sophorae Flavescentis & Rhizoma Heterosmilacis* | PLA2G5 |
| *Rhizoma Heterosmilacis* | CDC45 | *Radix Sophorae Flavescentis* | TNFRSF1A | *Radix Sophorae Flavescentis & Rhizoma Heterosmilacis* | PNLIP |
| *Rhizoma Heterosmilacis* | CYP27B1 | *Radix Sophorae Flavescentis* | TRPM2 | *Radix Sophorae Flavescentis & Rhizoma Heterosmilacis* | PPO2 |
| *Rhizoma Heterosmilacis* | EPHA1 | *Radix Sophorae Flavescentis* | TUBA4A | *Radix Sophorae Flavescentis & Rhizoma Heterosmilacis* | RDH8 |
| *Rhizoma Heterosmilacis* | EPHA4 | *Radix Sophorae Flavescentis* | TXNRD1 | *Radix Sophorae Flavescentis & Rhizoma Heterosmilacis* | SECA1 |
| *Rhizoma Heterosmilacis* | EPHA6 | *Radix Sophorae Flavescentis* | SLC5A11 | *Radix Sophorae Flavescentis & Rhizoma Heterosmilacis* | SQLE |
| *Rhizoma Heterosmilacis* | EPHA8 | *Radix Sophorae Flavescentis* | PDPK1 | *Radix Sophorae Flavescentis & Rhizoma Heterosmilacis* | SSSIM |
| *Rhizoma Heterosmilacis* | EPHB1 | *Radix Sophorae Flavescentis* | SLC37A4 | *Radix Sophorae Flavescentis & Rhizoma Heterosmilacis* | TAS2R31 |
| *Rhizoma Heterosmilacis* | EPHB2 | *Radix Sophorae Flavescentis* | AURKA | *Radix Sophorae Flavescentis & Rhizoma Heterosmilacis* | TDP1 |
| *Rhizoma Heterosmilacis* | EPHB3 | *Radix Sophorae Flavescentis* | AURKC | *Radix Sophorae Flavescentis & Rhizoma Heterosmilacis* | VKORC1 |
| *Rhizoma Heterosmilacis* | FABP1 | *Radix Sophorae Flavescentis* | Q969P6 | *Radix Sophorae Flavescentis & Rhizoma Heterosmilacis* | ZWF1 |
| *Rhizoma Heterosmilacis* | GC | *Radix Sophorae Flavescentis* | EDNRA | *Radix Sophorae Flavescentis & Rhizoma Heterosmilacis* | CA15 |
| *Rhizoma Heterosmilacis* | LDLR | *Radix Sophorae Flavescentis* | EDNRB | *Radix Sophorae Flavescentis & Rhizoma Heterosmilacis* | FGFR1 |
| *Rhizoma Heterosmilacis* | LRP8 | *Radix Sophorae Flavescentis* | CYBA | *Radix Sophorae Flavescentis & Rhizoma Heterosmilacis* | FLT1 |
| *Rhizoma Heterosmilacis* | NR1H2 | *Radix Sophorae Flavescentis* | MT-ND4 | *Radix Sophorae Flavescentis & Rhizoma Heterosmilacis* | MMP1 |
| *Rhizoma Heterosmilacis* | P05093 | *Radix Sophorae Flavescentis* | N1L | *Radix Sophorae Flavescentis & Rhizoma Heterosmilacis* | MMP10 |
| *Rhizoma Heterosmilacis* | RORC | *Radix Sophorae Flavescentis* | YBTE | *Radix Sophorae Flavescentis & Rhizoma Heterosmilacis* | MMP12 |
| *Rhizoma Heterosmilacis* | SHH | *Radix Sophorae Flavescentis* | CLK2 | *Radix Sophorae Flavescentis & Rhizoma Heterosmilacis* | MMP27 |
| *Rhizoma Heterosmilacis* | SLC10A2 | *Radix Sophorae Flavescentis* | CLK3 | *Radix Sophorae Flavescentis & Rhizoma Heterosmilacis* | PTGS1 |
| *Rhizoma Heterosmilacis* | SLC22A3 | *Radix Sophorae Flavescentis* | CLK4 | *Radix Sophorae Flavescentis & Rhizoma Heterosmilacis* | PTGS2 |
| *Rhizoma Heterosmilacis* | SREBF1 | *Radix Sophorae Flavescentis* | ACVRL1 | *Radix Sophorae Flavescentis & Rhizoma Heterosmilacis* | SRC |
| *Rhizoma Heterosmilacis* | VDRA | *Radix Sophorae Flavescentis* | AROA | *Radix Sophorae Flavescentis & Rhizoma Heterosmilacis* | BACE2 |
| *Rhizoma Heterosmilacis* | VLDLR | *Radix Sophorae Flavescentis* | CYP27A1 | *Radix Sophorae Flavescentis & Rhizoma Heterosmilacis* | MMP20 |
| *Rhizoma Heterosmilacis* | UGT2B7 | *Radix Sophorae Flavescentis* | MAP3K7 | *Radix Sophorae Flavescentis & Rhizoma Heterosmilacis* | PYGM |
| *Rhizoma Heterosmilacis* | DNM2 | *Radix Sophorae Flavescentis* | SLC5A5 | *Radix Sophorae Flavescentis & Rhizoma Heterosmilacis* | SUB-1 |
| *Rhizoma Heterosmilacis* | DNM3 | *Radix Sophorae Flavescentis* | CYP2D6 | *Radix Sophorae Flavescentis & Rhizoma Heterosmilacis* | AR |
| *Rhizoma Heterosmilacis* | GBA | *Radix Sophorae Flavescentis* | ACVR2A | *Radix Sophorae Flavescentis & Rhizoma Heterosmilacis* | CDC25A |
| *Rhizoma Heterosmilacis* | GLTP | *Radix Sophorae Flavescentis* | ACVR2B | *Radix Sophorae Flavescentis & Rhizoma Heterosmilacis* | CDC25C |
| *Rhizoma Heterosmilacis* | GRIN2A | *Radix Sophorae Flavescentis* | CAMK4 | *Radix Sophorae Flavescentis & Rhizoma Heterosmilacis* | CES2 |
| *Rhizoma Heterosmilacis* | P0504H10.9 | *Radix Sophorae Flavescentis* | CDC42BPB | *Radix Sophorae Flavescentis & Rhizoma Heterosmilacis* | CNR1 |
| *Rhizoma Heterosmilacis* | RAD52 | *Radix Sophorae Flavescentis* | CDK13 | *Radix Sophorae Flavescentis & Rhizoma Heterosmilacis* | CNR2 |
| *Radix Sophorae Flavescentis* | ACLY | *Radix Sophorae Flavescentis* | CDK15 | *Radix Sophorae Flavescentis & Rhizoma Heterosmilacis* | EPHX2 |
| *Radix Sophorae Flavescentis* | AHR | *Radix Sophorae Flavescentis* | CDK18 | *Radix Sophorae Flavescentis & Rhizoma Heterosmilacis* | FAAH |
| *Radix Sophorae Flavescentis* | APP | *Radix Sophorae Flavescentis* | CDKL5 | *Radix Sophorae Flavescentis & Rhizoma Heterosmilacis* | GAA |
| *Radix Sophorae Flavescentis* | BLAIMP-1 | *Radix Sophorae Flavescentis* | CRK2 | *Radix Sophorae Flavescentis & Rhizoma Heterosmilacis* | KCNK2 |
| *Radix Sophorae Flavescentis* | CALM1 | *Radix Sophorae Flavescentis* | CSNK1A1L | *Radix Sophorae Flavescentis & Rhizoma Heterosmilacis* | LUXP |
| *Radix Sophorae Flavescentis* | CD38 | *Radix Sophorae Flavescentis* | GNB1 | *Radix Sophorae Flavescentis & Rhizoma Heterosmilacis* | MEP2 |
| *Radix Sophorae Flavescentis* | CLK1 | *Radix Sophorae Flavescentis* | GNG2 | *Radix Sophorae Flavescentis & Rhizoma Heterosmilacis* | P35218 |
| *Radix Sophorae Flavescentis* | CSNK2A2 | *Radix Sophorae Flavescentis* | MAK | *Radix Sophorae Flavescentis & Rhizoma Heterosmilacis* | PAM |
| *Radix Sophorae Flavescentis* | CSNK2B | *Radix Sophorae Flavescentis* | MNAT1 | *Radix Sophorae Flavescentis & Rhizoma Heterosmilacis* | POLL |
| *Radix Sophorae Flavescentis* | KARS | *Radix Sophorae Flavescentis* | PIP5K1C | *Radix Sophorae Flavescentis & Rhizoma Heterosmilacis* | PREP |
| *Radix Sophorae Flavescentis* | NAE1 | *Radix Sophorae Flavescentis* | STK32A | *Radix Sophorae Flavescentis & Rhizoma Heterosmilacis* | PRKCA |
| *Radix Sophorae Flavescentis* | PARP1 | *Radix Sophorae Flavescentis* | VRK2 | *Radix Sophorae Flavescentis & Rhizoma Heterosmilacis* | PRKCD |
| *Radix Sophorae Flavescentis* | PPARG | *Radix Sophorae Flavescentis* | TRPM8 | *Radix Sophorae Flavescentis & Rhizoma Heterosmilacis* | PRKCH |
| *Radix Sophorae Flavescentis* | PRKDC | *Radix Sophorae Flavescentis* | DPP7 | *Radix Sophorae Flavescentis & Rhizoma Heterosmilacis* | Q9Y2D0 |
| *Radix Sophorae Flavescentis* | RMLD | *Radix Sophorae Flavescentis* | GRM1 | *Radix Sophorae Flavescentis & Rhizoma Heterosmilacis* | SLC22A6 |
| *Radix Sophorae Flavescentis* | SLC6A3 | *Radix Sophorae Flavescentis* | GRM5 | *Radix Sophorae Flavescentis & Rhizoma Heterosmilacis* | TLR2 |
| *Radix Sophorae Flavescentis* | SYK | *Radix Sophorae Flavescentis* | KDM4A | *Radix Sophorae Flavescentis & Rhizoma Heterosmilacis* | CA5B |
| *Radix Sophorae Flavescentis* | TEM-1 | *Radix Sophorae Flavescentis* | KDM4B | *Radix Sophorae Flavescentis & Rhizoma Heterosmilacis* | ADORA3 |
| *Radix Sophorae Flavescentis* | TNKS | *Radix Sophorae Flavescentis* | KDM4C | *Radix Sophorae Flavescentis & Rhizoma Heterosmilacis* | ERN1 |
| *Radix Sophorae Flavescentis* | TNKS2 | *Radix Sophorae Flavescentis* | MMP8 | *Radix Sophorae Flavescentis & Rhizoma Heterosmilacis* | OPRK1 |
| *Radix Sophorae Flavescentis* | AKT2 | *Radix Sophorae Flavescentis* | CYP2E1 | *Radix Sophorae Flavescentis & Rhizoma Heterosmilacis* | CHRNB1 |
| *Radix Sophorae Flavescentis* | AKT3 | *Radix Sophorae Flavescentis* | E2 | *Radix Sophorae Flavescentis & Rhizoma Heterosmilacis* | CHRND |
| *Radix Sophorae Flavescentis* | ALDH2 | *Radix Sophorae Flavescentis* | FFAR1 | *Radix Sophorae Flavescentis & Rhizoma Heterosmilacis* | CHRNG |
| *Radix Sophorae Flavescentis* | CTSV | *Radix Sophorae Flavescentis* | HTR5A | *Radix Sophorae Flavescentis & Rhizoma Heterosmilacis* | CRYZ |
| *Radix Sophorae Flavescentis* | HIF1A | *Radix Sophorae Flavescentis* | MCHR1 | *Radix Sophorae Flavescentis & Rhizoma Heterosmilacis* | PRKCB |
| *Radix Sophorae Flavescentis* | O76074 | *Radix Sophorae Flavescentis* | NR0B1 | *Radix Sophorae Flavescentis & Rhizoma Heterosmilacis* | PRKCG |
| *Radix Sophorae Flavescentis* | P18054 | *Radix Sophorae Flavescentis* | TUBA1A | *Radix Sophorae Flavescentis & Rhizoma Heterosmilacis* | PRKCQ |
| *Radix Sophorae Flavescentis* | Q9HCR9 | *Radix Sophorae Flavescentis* | IL1B | *Radix Sophorae Flavescentis & Rhizoma Heterosmilacis* | PTPN1 |
| *Radix Sophorae Flavescentis* | XPO1 | *Radix Sophorae Flavescentis* | PTPN11 | *Radix Sophorae Flavescentis & Rhizoma Heterosmilacis* | PTPN2 |
| *Radix Sophorae Flavescentis* | CHRNA4 | *Radix Sophorae Flavescentis* | Q96JD6 | *Radix Sophorae Flavescentis & Rhizoma Heterosmilacis* | RELA |
| *Radix Sophorae Flavescentis* | CHRNB2 | *Radix Sophorae Flavescentis* | PTPRF | *Radix Sophorae Flavescentis & Rhizoma Heterosmilacis* | PLCG1 |
| *Radix Sophorae Flavescentis* | KCNH2 | *Radix Sophorae Flavescentis* | EP300 | *Radix Sophorae Flavescentis & Rhizoma Heterosmilacis* | BCHE |
| *Radix Sophorae Flavescentis* | KCNH6 | *Radix Sophorae Flavescentis* | ITGAL | *Radix Sophorae Flavescentis & Rhizoma Heterosmilacis* | BCL2 |
| *Radix Sophorae Flavescentis* | KCNH7 | *Radix Sophorae Flavescentis* | NR1I2 | *Radix Sophorae Flavescentis & Rhizoma Heterosmilacis* | BCL2L1 |
| *Radix Sophorae Flavescentis* | OPRL1 | *Radix Sophorae Flavescentis* | SIRT1 | *Radix Sophorae Flavescentis & Rhizoma Heterosmilacis* | DRD2 |
| *Radix Sophorae Flavescentis* | OPRM1 | *Radix Sophorae Flavescentis* | SLCO1B1 | *Radix Sophorae Flavescentis & Rhizoma Heterosmilacis* | DRD3 |
| *Radix Sophorae Flavescentis* | Q14957 | *Radix Sophorae Flavescentis* | BLA(TEM-2) | *Radix Sophorae Flavescentis & Rhizoma Heterosmilacis* | FGF1 |
| *Radix Sophorae Flavescentis* | HTR3B | *Radix Sophorae Flavescentis* | IKBKG | *Radix Sophorae Flavescentis & Rhizoma Heterosmilacis* | FGF2 |
| *Radix Sophorae Flavescentis* | CD81 | *Radix Sophorae Flavescentis* | KCNA1 | *Radix Sophorae Flavescentis & Rhizoma Heterosmilacis* | NPC1L1 |
| *Radix Sophorae Flavescentis* | F3 | *Radix Sophorae Flavescentis* | KCNA10 | *Radix Sophorae Flavescentis & Rhizoma Heterosmilacis* | POLA1 |
| *Radix Sophorae Flavescentis* | HSD11B2 | *Radix Sophorae Flavescentis* | KCNA2 | *Radix Sophorae Flavescentis & Rhizoma Heterosmilacis* | SLC5A1 |
| *Radix Sophorae Flavescentis* | PLA2 | *Radix Sophorae Flavescentis* | KCNA4 | *Radix Sophorae Flavescentis & Rhizoma Heterosmilacis* | SLC5A2 |
| *Radix Sophorae Flavescentis* | PROTEASE | *Radix Sophorae Flavescentis* | KCNA5 | *Radix Sophorae Flavescentis & Rhizoma Heterosmilacis* | SRD5A2 |
| *Radix Sophorae Flavescentis* | PTPN6 | *Radix Sophorae Flavescentis* | KCNA6 | *Radix Sophorae Flavescentis & Rhizoma Heterosmilacis* | CSF1R |
| *Radix Sophorae Flavescentis* | SLC6A14 | *Radix Sophorae Flavescentis* | KCNA7 | *Radix Sophorae Flavescentis & Rhizoma Heterosmilacis* | FGFR2 |
| *Radix Sophorae Flavescentis* | SLC6A5 | *Radix Sophorae Flavescentis* | TLR9 | *Radix Sophorae Flavescentis & Rhizoma Heterosmilacis* | CYP24A1 |
| *Radix Sophorae Flavescentis* | SLC6A7 | *Radix Sophorae Flavescentis* | B4GALT1 | *Radix Sophorae Flavescentis & Rhizoma Heterosmilacis* | HRH3 |
| *Radix Sophorae Flavescentis* | SLC6A9 | *Radix Sophorae Flavescentis* | FIMH | *Radix Sophorae Flavescentis & Rhizoma Heterosmilacis* | NR1H4 |
| *Radix Sophorae Flavescentis* | TPH1 | *Radix Sophorae Flavescentis* | LGALS4 | *Radix Sophorae Flavescentis & Rhizoma Heterosmilacis* | PGR |
| *Radix Sophorae Flavescentis* | U38 | *Radix Sophorae Flavescentis* | LGALS3 | *Radix Sophorae Flavescentis & Rhizoma Heterosmilacis* | SLC6A2 |
| *Radix Sophorae Flavescentis* | ALOXE3 | *Radix Sophorae Flavescentis* | LGALS8 | *Radix Sophorae Flavescentis & Rhizoma Heterosmilacis* | SLC6A4 |
| *Radix Sophorae Flavescentis* | CHRNB3 | *Radix Sophorae Flavescentis* | LGALS9 | *Radix Sophorae Flavescentis & Rhizoma Heterosmilacis* | SRD5A1 |
