# Supplementary material for: A System Pharmacology Model for Decoding the Synergistic Mechanisms of Compound Kushen Injection in Treating Breast Cancer
Source: Front Pharmacol. 2021 Nov 16;12:723147. doi: 10.3389/fphar.2021.723147 (PMC8660088; doi:10.3389/fphar.2021.723147)
Supplement: Supplementary file 9 [file Table3.DOCX]

**Table S3 |** The detail information of the components in CKI based on TCMSP database

| **Herb** | **Molecule name** | **MW** | **AlogP** | **nHDon** | **nHAcc** | **Caco-2** | **DL** | **FASA-** | **TPSA** | **RBN** |
| --- | --- | --- | --- | --- | --- | --- | --- | --- | --- | --- |
| *Radix Sophorae Flavescentis* | luteolin | 286.25 | 2.07 | 4 | 6 | 0.19 | 0.25 | 0.39 | 111.13 | 1 |
| *Radix Sophorae Flavescentis* | apigenin | 270.25 | 2.33 | 3 | 5 | 0.43 | 0.21 | 0.41 | 90.9 | 1 |
| *Radix Sophorae Flavescentis* | luteolin-7-o-glucoside | 448.41 | 0.16 | 7 | 11 | -1.23 | 0.78 | 0.32 | 190.28 | 4 |
| *Radix Sophorae Flavescentis* | quercetin | 302.25 | 1.5 | 5 | 7 | 0.05 | 0.28 | 0.38 | 131.36 | 1 |
| *Radix Sophorae Flavescentis* | formononetin | 268.28 | 2.58 | 1 | 4 | 0.78 | 0.21 | 0 | 59.67 | 2 |
| *Radix Sophorae Flavescentis* | Soyasaponin I | 943.26 | 0.97 | 11 | 18 | -2.75 | 0.05 | 0 | 294.98 | 9 |
| *Radix Sophorae Flavescentis* | Phaseolin | 322.38 | 3.46 | 1 | 4 | 1.09 | 0.73 | 0.33 | 47.92 | 0 |
| *Radix Sophorae Flavescentis* | Kushenol E | 424.53 | 5.74 | 4 | 6 | 0.58 | 0.59 | 0.32 | 107.22 | 5 |
| *Radix Sophorae Flavescentis* | (2R)-5,7-dihydroxy-2-(4-hydroxyphenyl)chroman-4-one | 272.27 | 2.3 | 3 | 5 | 0.38 | 0.21 | 0.41 | 86.99 | 1 |
| *Radix Sophorae Flavescentis* | Inermine | 284.28 | 2.44 | 1 | 5 | 0.89 | 0.54 | 0.3 | 57.15 | 0 |
| *Radix Sophorae Flavescentis* | hyperforin | 536.87 | 8.62 | 1 | 4 | 0.87 | 0.6 | 0 | 71.44 | 11 |
| *Radix Sophorae Flavescentis* | 8-Isopentenyl-kaempferol | 354.38 | 3.63 | 4 | 6 | 0.53 | 0.39 | 0 | 111.13 | 3 |
| *Radix Sophorae Flavescentis* | sophocarpine | 246.39 | 1.39 | 0 | 3 | 0.99 | 0.25 | 0.26 | 23.55 | 0 |
| *Radix Sophorae Flavescentis* | Soyasapogenol B | 458.8 | 5.11 | 3 | 3 | 0.43 | 0.75 | 0.21 | 60.69 | 1 |
| *Radix Sophorae Flavescentis* | Inermin | 284.28 | 2.44 | 1 | 5 | 0.91 | 0.54 | 0.3 | 57.15 | 0 |
| *Radix Sophorae Flavescentis* | Wighteone | 338.38 | 3.92 | 3 | 5 | 0.64 | 0.36 | 0.31 | 90.9 | 3 |
| *Radix Sophorae Flavescentis* | Sophoramine | 244.37 | 1.15 | 0 | 3 | 1.43 | 0.25 | 0.22 | 25.24 | 0 |
| *Radix Sophorae Flavescentis* | sophoridine | 248.41 | 1.42 | 0 | 3 | 1.13 | 0.25 | 0.18 | 23.55 | 0 |
| *Radix Sophorae Flavescentis* | cis-Dihydroquercetin | 304.27 | 1.49 | 5 | 7 | -0.34 | 0.27 | 0.4 | 127.45 | 1 |
| *Radix Sophorae Flavescentis* | (2R)-7-hydroxy-2-(4-hydroxyphenyl)chroman-4-one | 256.27 | 2.57 | 2 | 4 | 0.41 | 0.18 | 0 | 66.76 | 1 |
| *Radix Sophorae Flavescentis* | 5,7-dihydroxy-2-(3-hydroxy-4-methoxyphenyl)chroman-4-one | 302.3 | 2.28 | 3 | 6 | 0.28 | 0.27 | 0.31 | 96.22 | 2 |
| *Radix Sophorae Flavescentis* | matrine | 248.41 | 1.42 | 0 | 3 | 1.39 | 0.25 | 0 | 23.55 | 0 |
| *Radix Sophorae Flavescentis* | (+)-14alpha-hydroxymatrine | 264.41 | 0.74 | 1 | 4 | 0.53 | 0.29 | 0.21 | 43.78 | 0 |
| *Radix Sophorae Flavescentis* | (+)-7,11-dehydromatrine,(leontalbinine) | 246.39 | 1.42 | 0 | 3 | 1.06 | 0.25 | 0.16 | 23.55 | 0 |
| *Radix Sophorae Flavescentis* | (+)-9alpha-hydroxymatrine | 264.41 | 0.45 | 1 | 4 | 0.61 | 0.29 | 0.19 | 43.78 | 0 |
| *Radix Sophorae Flavescentis* | (+)-allomatrine | 248.41 | 1.42 | 0 | 3 | 1.08 | 0.25 | 0.18 | 23.55 | 0 |
| *Radix Sophorae Flavescentis* | AIDS211310 | 248.41 | 1.42 | 0 | 3 | 1.15 | 0.25 | 0.17 | 23.55 | 0 |
| *Radix Sophorae Flavescentis* | (+)-lehmannine | 246.39 | 1.11 | 0 | 3 | 1.21 | 0.25 | 0.21 | 23.55 | 0 |
| *Radix Sophorae Flavescentis* | (+)-sophoranol | 264.41 | 0.67 | 1 | 4 | 0.44 | 0.28 | 0.2 | 43.78 | 0 |
| *Radix Sophorae Flavescentis* | isosophocarpine | 246.39 | 1.39 | 0 | 3 | 1.39 | 0.25 | 0.24 | 23.55 | 0 |
| *Radix Sophorae Flavescentis* | (-)-14beta-hydroxymatrine | 264.41 | 0.74 | 1 | 4 | 0.77 | 0.29 | 0.21 | 43.78 | 0 |
| *Radix Sophorae Flavescentis* | (-)-9alpha-hydroxysophoramine | 262.39 | 0.43 | 1 | 4 | 0.38 | 0.29 | 0.25 | 43.78 | 0 |
| *Radix Sophorae Flavescentis* | agyrine | 244.37 | 1.15 | 0 | 3 | 1.16 | 0.24 | 0 | 25.24 | 0 |
| *Radix Sophorae Flavescentis* | 1,4-diazaindan-type,alkaloid,flavascensine | 348.64 | 5.4 | 2 | 3 | 1.13 | 0.24 | 0.23 | 41.13 | 10 |
| *Radix Sophorae Flavescentis* | 13,14-dehydrosophoridine | 246.39 | 1.39 | 0 | 3 | 1.06 | 0.25 | 0.24 | 23.55 | 0 |
| *Radix Sophorae Flavescentis* | 2-Hydroxychalcone | 224.27 | 3.44 | 1 | 2 | 1.06 | 0.1 | 0.48 | 37.3 | 3 |
| *Radix Sophorae Flavescentis* | 2-n-hencosyl-5,7-dihydroxy-6,8-dimethylchromone | 500.84 | 11.53 | 2 | 4 | 1.24 | 0.66 | 0.18 | 70.67 | 20 |
| *Radix Sophorae Flavescentis* | 2-n-heptadecyl-5,7-dihydroxy-6,8-dimethyl chromone | 444.72 | 9.71 | 2 | 4 | 1.18 | 0.65 | 0.19 | 70.67 | 16 |
| *Radix Sophorae Flavescentis* | 2-n-nodecyl-5,7-dihydroxy-6,8-dimethyl chromone | 472.78 | 10.62 | 2 | 4 | 1.2 | 0.68 | 0.18 | 70.67 | 18 |
| *Radix Sophorae Flavescentis* | 2-n-pentacosyl-5,7-dihydroxy-6,8-dimethyl chromone | 556.96 | 13.36 | 2 | 4 | 1.31 | 0.53 | 0.17 | 70.67 | 24 |
| *Radix Sophorae Flavescentis* | 2-n-pentadecyl-5,7-dihydroxy-6,8-dimethyl chromone | 416.66 | 8.79 | 2 | 4 | 1.16 | 0.58 | 0.2 | 70.67 | 14 |
| *Radix Sophorae Flavescentis* | 2-n-tricosyl-5,7-dihydroxy-6,8-dimethyl chromone | 528.9 | 12.44 | 2 | 4 | 1.27 | 0.6 | 0.18 | 70.67 | 22 |
| *Radix Sophorae Flavescentis* | 2-n-tridecyl-5,7-dihydroxy-6,8-dimethyl chromone | 388.6 | 7.88 | 2 | 4 | 1.16 | 0.48 | 0.2 | 70.67 | 12 |
| *Radix Sophorae Flavescentis* | 5α,9α-dihydroxymatrine | 280.41 | -0.3 | 2 | 5 | 0.04 | 0.32 | 0.2 | 64.01 | 0 |
| *Radix Sophorae Flavescentis* | 7,11-dehydromatrine | 246.39 | 1.42 | 0 | 3 | 1.11 | 0.25 | 0.16 | 23.55 | 0 |
| *Radix Sophorae Flavescentis* | 9alpha-hydroxy-7,11-dehydromatrine | 262.39 | 0.46 | 1 | 4 | 0.35 | 0.29 | 0.18 | 43.78 | 0 |
| *Radix Sophorae Flavescentis* | Kushenol I | 438.51 | 5.04 | 5 | 7 | 0.44 | 0.63 | 0.35 | 131.36 | 6 |
| *Radix Sophorae Flavescentis* | Kushenol M | 522.69 | 7.13 | 4 | 7 | 0.35 | 0.77 | 0.31 | 116.45 | 9 |
| *Radix Sophorae Flavescentis* | N-allomatrine | 248.41 | 1.42 | 0 | 3 | 0.99 | 0.25 | 0.18 | 23.55 | 0 |
| *Radix Sophorae Flavescentis* | N-oxysophocarpine | 262.39 | -0.86 | 0 | 3 | 1.02 | 0.29 | 0.05 | 37.38 | 0 |
| *Radix Sophorae Flavescentis* | Artepillin C | 300.43 | 5.35 | 2 | 3 | 0.83 | 0.17 | 0.35 | 57.53 | 6 |
| *Radix Sophorae Flavescentis* | 7,14-Methano-4H,6H-dipyrido(1,2-a:1',2'-e)(1,5)diazocin-4-one, 7,7a,8,9,10,11,13,14-octahydro-9-hydroxy-, (7R-(7alpha,7abeta,9alpha,14alpha))- | 260.37 | -0.34 | 1 | 4 | 0.42 | 0.28 | 0.22 | 45.47 | 0 |
| *Radix Sophorae Flavescentis* | Thc-9-cooh | 358.52 | 5.69 | 2 | 4 | 0.89 | 0.43 | 0.27 | 66.76 | 5 |
| *Radix Sophorae Flavescentis* | 7-Demethylsuberosin | 230.28 | 3.49 | 1 | 3 | 0.98 | 0.11 | 0.34 | 50.44 | 2 |
| *Radix Sophorae Flavescentis* | Deoxyhumulone | 346.51 | 5.86 | 3 | 4 | 0.79 | 0.22 | 0.3 | 77.76 | 7 |
| *Radix Sophorae Flavescentis* | Eciphin | 165.26 | 1.24 | 2 | 2 | 0.92 | 0.03 | 0.34 | 32.26 | 3 |
| *Radix Sophorae Flavescentis* | (2R)-flavanone | 224.27 | 3.1 | 0 | 2 | 1.25 | 0.13 | 0.44 | 26.3 | 1 |
| *Radix Sophorae Flavescentis* | Glyceollin | 338.38 | 2.85 | 2 | 5 | 0.53 | 0.76 | 0.35 | 68.15 | 0 |
| *Radix Sophorae Flavescentis* | OMD | 168.16 | 0.93 | 3 | 4 | 0.24 | 0.04 | 0.41 | 77.76 | 2 |
| *Radix Sophorae Flavescentis* | 3,4',5-Trihydroxy-7-methoxy-8-isopente-nylflavone | 368.41 | 3.88 | 3 | 6 | 0.56 | 0.43 | 0.33 | 100.13 | 4 |
| *Radix Sophorae Flavescentis* | 1-[2,4-dihydroxy-3-(3-methylbut-2-enyl)phenyl]-3-phenylprop-2-en-1-one | 308.4 | 5.02 | 2 | 3 | 1.04 | 0.23 | 0.42 | 57.53 | 5 |
| *Radix Sophorae Flavescentis* | kuraranine | 222.32 | -0.36 | 2 | 4 | 0.16 | 0.09 | 0.25 | 56.33 | 2 |
| *Radix Sophorae Flavescentis* | isokurarinone | 438.56 | 5.81 | 3 | 6 | 0.56 | 0.66 | 0.31 | 96.22 | 7 |
| *Radix Sophorae Flavescentis* | (E)-1-(2,6-dihydroxyphenyl)-3-(4-hydroxyphenyl)prop-2-en-1-one | 256.27 | 2.9 | 3 | 4 | 0.54 | 0.15 | 0.49 | 77.76 | 3 |
| *Radix Sophorae Flavescentis* | (2S)-7-hydroxy-2-(4-hydroxyphenyl)-5-methoxy-8-(3-methylbut-2-enyl)chroman-4-one | 354.43 | 4.41 | 2 | 5 | 0.8 | 0.39 | 0.3 | 75.99 | 4 |
| *Radix Sophorae Flavescentis* | kosamol,q | 454.61 | 6.67 | 4 | 6 | 0.7 | 0.69 | 0.33 | 107.22 | 8 |
| *Radix Sophorae Flavescentis* | kosamol,r | 450.62 | 6.89 | 2 | 5 | 1.13 | 0.67 | 0.31 | 75.99 | 9 |
| *Radix Sophorae Flavescentis* | kurarainone | 438.56 | 5.81 | 3 | 6 | 0.67 | 0.63 | 0.32 | 96.22 | 7 |
| *Radix Sophorae Flavescentis* | kuraridin | 438.56 | 6.15 | 4 | 6 | 0.47 | 0.53 | 0.34 | 107.22 | 9 |
| *Radix Sophorae Flavescentis* | kuraridine | 494.63 | 5.95 | 5 | 7 | 0.25 | 0.75 | 0.33 | 127.45 | 9 |
| *Radix Sophorae Flavescentis* | kuraridinol | 472.58 | 4.48 | 6 | 8 | -0.37 | 0.58 | 0.33 | 147.68 | 10 |
| *Radix Sophorae Flavescentis* | kurarinol | 456.58 | 4.71 | 4 | 7 | 0.12 | 0.67 | 0.3 | 116.45 | 8 |
| *Radix Sophorae Flavescentis* | kurarinone | 424.53 | 5.69 | 4 | 6 | 0.46 | 0.49 | 0.34 | 107.22 | 8 |
| *Radix Sophorae Flavescentis* | kushenin | 286.3 | 2.39 | 2 | 5 | 0.71 | 0.38 | 0.27 | 68.15 | 1 |
| *Radix Sophorae Flavescentis* | kushenol A | 408.53 | 5.83 | 3 | 5 | 0.67 | 0.55 | 0.33 | 86.99 | 6 |
| *Radix Sophorae Flavescentis* | kushenol B | 492.66 | 7.42 | 4 | 6 | 0.61 | 0.75 | 0.31 | 107.22 | 8 |
| *Radix Sophorae Flavescentis* | kushenol D | 452.59 | 6.4 | 3 | 6 | 0.63 | 0.57 | 0.3 | 96.22 | 10 |
| *Radix Sophorae Flavescentis* | kushenol F | 424.53 | 5.56 | 4 | 6 | 0.45 | 0.61 | 0.33 | 107.22 | 6 |
| *Radix Sophorae Flavescentis* | KushenolG | 456.53 | 3.94 | 6 | 8 | -0.28 | 0.67 | 0.33 | 151.59 | 7 |
| *Radix Sophorae Flavescentis* | kushenol J | 580.59 | -0.87 | 7 | 14 | -2.2 | 0.74 | 0.31 | 214.06 | 7 |
| *Radix Sophorae Flavescentis* | kushenol J_qt | 286.3 | 2.27 | 2 | 5 | 0.24 | 0.24 | 0.37 | 75.99 | 2 |
| *Radix Sophorae Flavescentis* | (2R,3R)-2-(2,4-dihydroxyphenyl)-3,7-dihydroxy-8-[(2R)-2-isopropenyl-5-methylhex-4-enyl]-5-methoxy-4-chromanone | 454.56 | 5.27 | 4 | 7 | 0.11 | 0.66 | 0.3 | 116.45 | 7 |
| *Radix Sophorae Flavescentis* | kushenol O | 562.57 | -0.56 | 6 | 13 | -1.67 | 0.76 | 0.29 | 197.74 | 7 |
| *Radix Sophorae Flavescentis* | kushenol,t | 442.55 | 4.46 | 5 | 7 | -0.05 | 0.64 | 0.35 | 127.45 | 7 |
| *Radix Sophorae Flavescentis* | kushequinone A | 290.39 | 2.84 | 1 | 4 | 0.65 | 0.14 | 0.28 | 63.6 | 6 |
| *Radix Sophorae Flavescentis* | leachianone,a | 438.56 | 5.81 | 3 | 6 | 0.63 | 0.65 | 0.31 | 96.22 | 7 |
| *Radix Sophorae Flavescentis* | leachianone,g | 356.4 | 3.89 | 4 | 6 | 0.33 | 0.4 | 0.37 | 107.22 | 3 |
| *Radix Sophorae Flavescentis* | Lehmanine | 246.39 | 1.11 | 0 | 3 | 1.18 | 0.25 | 0.21 | 23.55 | 0 |
| *Radix Sophorae Flavescentis* | (+)-Lupanine | 248.41 | 1.42 | 0 | 3 | 1.16 | 0.24 | 0.18 | 23.55 | 0 |
| *Radix Sophorae Flavescentis* | mamanine | 262.39 | 0.58 | 2 | 4 | 0.25 | 0.17 | 0.23 | 56.33 | 2 |
| *Radix Sophorae Flavescentis* | Norartocarpetin | 286.25 | 2.07 | 4 | 6 | 0.14 | 0.24 | 0.42 | 111.13 | 1 |
| *Radix Sophorae Flavescentis* | (2R)-2-(2,4-dihydroxyphenyl)-5,7-dihydroxy-8-[(2S)-2-isopropenyl-5-methylhex-4-enyl]-4-chromanone | 424.53 | 5.56 | 4 | 6 | 0.52 | 0.6 | 0.35 | 107.22 | 6 |
| *Radix Sophorae Flavescentis* | (2S)-2-(3,4-dihydroxyphenyl)-6-[(2E)-3,7-dimethylocta-2,6-dienyl]-5,7-dihydroxychroman-4-one | 424.53 | 5.71 | 4 | 6 | 0.48 | 0.65 | 0.34 | 107.22 | 6 |
| *Radix Sophorae Flavescentis* | DIMETHYLALLYLUMBELLIFERONE, 0- | 230.28 | 3.35 | 0 | 3 | 1.17 | 0.12 | 0.33 | 39.44 | 3 |
| *Radix Sophorae Flavescentis* | oxymatrine | 264.41 | -0.83 | 0 | 3 | 1 | 0.28 | 0.03 | 37.38 | 0 |
| *Radix Sophorae Flavescentis* | oxysophocarpine | 262.39 | -0.86 | 0 | 3 | 1.04 | 0.29 | 0.03 | 37.38 | 0 |
| *Radix Sophorae Flavescentis* | Psi-ephedrin | 165.26 | 1.24 | 2 | 2 | 1.03 | 0.03 | 0.34 | 32.26 | 3 |
| *Radix Sophorae Flavescentis* | Pterocarpine | 298.31 | 2.69 | 0 | 5 | 1.12 | 0.6 | 0.25 | 46.15 | 1 |
| *Radix Sophorae Flavescentis* | TNP00221 | 244.37 | 1.14 | 0 | 3 | 1.24 | 0.17 | 0.25 | 25.24 | 3 |
| *Radix Sophorae Flavescentis* | sokurarinone | 450.62 | 6.89 | 2 | 5 | 0.77 | 0.71 | 0.33 | 75.99 | 9 |
| *Radix Sophorae Flavescentis* | sophoraflavanone,g | 410.5 | 5.44 | 5 | 6 | 0.27 | 0.46 | 0.39 | 118.22 | 7 |
| *Radix Sophorae Flavescentis* | sophoraflavoside II | 973.24 | 0.09 | 12 | 20 | -3.62 | 0.05 | 0.3 | 332.28 | 10 |
| *Radix Sophorae Flavescentis* | sophoraflavosideⅢ_qt | 488.78 | 4.23 | 4 | 5 | -0.33 | 0.72 | 0.22 | 97.99 | 2 |
| *Radix Sophorae Flavescentis* | sophoraflavoside IV | 1,267.53 | -2.9 | 17 | 29 | -5.64 | 0.01 | 0.3 | 470.35 | 15 |
| *Radix Sophorae Flavescentis* | sophoraflavoside IV_qt | 488.78 | 4.23 | 4 | 5 | -0.36 | 0.72 | 0.24 | 97.99 | 2 |
| *Radix Sophorae Flavescentis* | sophoraflavosideⅢ | 1,105.37 | -0.72 | 14 | 24 | -4.63 | 0.02 | 0.29 | 391.2 | 12 |
| *Radix Sophorae Flavescentis* | sophoraisoflavanone,a | 398.49 | 4.83 | 3 | 6 | 0.52 | 0.52 | 0.32 | 96.22 | 6 |
| *Radix Sophorae Flavescentis* | sophoranol N-oxide | 280.41 | -1.58 | 1 | 4 | 0.59 | 0.32 | 0.03 | 57.61 | 0 |
| *Radix Sophorae Flavescentis* | sophranol | 264.41 | 0.67 | 1 | 4 | 0.6 | 0.28 | 0.19 | 43.78 | 0 |
| *Radix Sophorae Flavescentis* | (-)-Maackiain-3-O-glucosyl-6'-O-malote | 532.49 | 0.7 | 4 | 13 | -1.45 | 0.52 | 0.34 | 179.67 | 7 |
| *Radix Sophorae Flavescentis* | Trifolirhizin | 446.44 | 0.54 | 4 | 10 | -0.83 | 0.79 | 0.28 | 136.3 | 3 |
| *Radix Sophorae Flavescentis* | trifolrhizin | 462.44 | 0.95 | 4 | 11 | -0.85 | 0.74 | 0.24 | 145.53 | 4 |
| *Radix Sophorae Flavescentis* | xanthohumol | 368.46 | 5.09 | 3 | 5 | 0.68 | 0.35 | 0.34 | 86.99 | 7 |
| *Rhizoma Heterosmilacis* | beta-Eudesmol | 222.41 | 3.72 | 1 | 1 | 1.32 | 0.1 | 0 | 20.23 | 1 |
| *Rhizoma Heterosmilacis* | palmitic acid | 256.48 | 6.37 | 1 | 2 | 1.09 | 0.1 | 0 | 37.3 | 14 |
| *Rhizoma Heterosmilacis* | Istidi | 155.18 | -1.01 | 4 | 4 | -0.25 | 0.03 | 0 | 92 | 3 |
| *Rhizoma Heterosmilacis* | quercetin | 302.25 | 1.5 | 5 | 7 | 0.05 | 0.28 | 0.38 | 131.36 | 1 |
| *Rhizoma Heterosmilacis* | (L)-alpha-Terpineol | 154.28 | 2.42 | 1 | 1 | 1.39 | 0.03 | 0.27 | 20.23 | 1 |
| *Rhizoma Heterosmilacis* | EIC | 280.5 | 6.39 | 1 | 2 | 1.16 | 0.14 | 0.25 | 37.3 | 14 |
| *Rhizoma Heterosmilacis* | L-Bornyl acetate | 196.32 | 2.35 | 0 | 2 | 1.29 | 0.08 | 0.23 | 26.3 | 2 |
| *Rhizoma Heterosmilacis* | (R)-lilool | 154.28 | 2.74 | 1 | 1 | 1.33 | 0.02 | 0.32 | 20.23 | 4 |
| *Rhizoma Heterosmilacis* | succinic acid | 118.1 | -0.41 | 2 | 4 | -0.44 | 0.01 | 0.42 | 74.6 | 3 |
| *Rhizoma Heterosmilacis* | Sitogluside | 576.95 | 6.34 | 4 | 6 | -0.14 | 0.62 | 0.23 | 99.38 | 9 |
| *Rhizoma Heterosmilacis* | beta-sitosterol | 414.79 | 8.08 | 1 | 1 | 1.32 | 0.75 | 0.23 | 20.23 | 6 |
| *Rhizoma Heterosmilacis* | sitosterol | 414.79 | 8.08 | 1 | 1 | 1.32 | 0.75 | 0.22 | 20.23 | 6 |
| *Rhizoma Heterosmilacis* | FER | 194.2 | 1.62 | 2 | 4 | 0.47 | 0.06 | 0.34 | 66.76 | 3 |
| *Rhizoma Heterosmilacis* | Stigmasterol | 412.77 | 7.64 | 1 | 1 | 1.44 | 0.76 | 0.22 | 20.23 | 5 |
| *Rhizoma Heterosmilacis* | diosgenin | 414.69 | 4.63 | 1 | 3 | 0.82 | 0.81 | 0.19 | 38.69 | 0 |
| *Rhizoma Heterosmilacis* | ()-Terpinen-4-ol | 154.28 | 2.55 | 1 | 1 | 1.36 | 0.03 | 0.25 | 20.23 | 1 |
| *Rhizoma Heterosmilacis* | delta-amorphene | 204.39 | 4.94 | 0 | 0 | 1.85 | 0.08 | 0.24 | 0 | 1 |
| *Rhizoma Heterosmilacis* | oleic acid | 282.52 | 6.84 | 1 | 2 | 1.17 | 0.14 | 0.2 | 37.3 | 15 |
| *Rhizoma Heterosmilacis* | trans-2,4-decadiel | 152.26 | 3.21 | 0 | 1 | 1.4 | 0.02 | 0.32 | 17.07 | 6 |
| *Rhizoma Heterosmilacis* | (2S,3R)-3,5,7-trihydroxy-2-(4-hydroxyphenyl)chroman-4-one | 288.27 | 1.75 | 4 | 6 | -0.1 | 0.24 | 0.41 | 107.22 | 1 |
| *Rhizoma Heterosmilacis* | HMF | 126.12 | 0.67 | 1 | 3 | 0.05 | 0.02 | 0.22 | 50.44 | 2 |
| *Rhizoma Heterosmilacis* | NCA | 122.14 | -0.32 | 2 | 3 | 0.44 | 0.02 | 0.33 | 55.98 | 1 |
| *Rhizoma Heterosmilacis* | stearic acid | 284.54 | 7.28 | 1 | 2 | 1.15 | 0.14 | 0.19 | 37.3 | 16 |
| *Rhizoma Heterosmilacis* | MYS | 212.47 | 7.22 | 0 | 0 | 1.81 | 0.05 | 0.15 | 0 | 12 |
| *Rhizoma Heterosmilacis* | hexadecane | 226.5 | 7.67 | 0 | 0 | 1.81 | 0.06 | 0.14 | 0 | 13 |
| *Rhizoma Heterosmilacis* | Cedrol | 222.41 | 3.16 | 1 | 1 | 1.35 | 0.12 | 0.24 | 20.23 | 0 |
| *Rhizoma Heterosmilacis* | Tricosane | 324.71 | 10.86 | 0 | 0 | 1.85 | 0.21 | 0.13 | 0 | 20 |
| *Rhizoma Heterosmilacis* | Dodekan | 170.38 | 5.85 | 0 | 0 | 1.79 | 0.02 | 0.16 | 0 | 9 |
| *Rhizoma Heterosmilacis* | myristic acid | 228.42 | 5.46 | 1 | 2 | 1.07 | 0.07 | 0.19 | 37.3 | 12 |
| *Rhizoma Heterosmilacis* | Methyllinolete | 292.51 | 6.2 | 0 | 2 | 1.48 | 0.17 | 0.24 | 26.3 | 14 |
| *Rhizoma Heterosmilacis* | TWT | 310.68 | 10.41 | 0 | 0 | 1.85 | 0.18 | 0.13 | 0 | 19 |
| *Rhizoma Heterosmilacis* | (-)-taxifolin | 304.27 | 1.49 | 5 | 7 | -0.24 | 0.27 | 0.41 | 127.45 | 1 |
| *Rhizoma Heterosmilacis* | Dihydro-beta-ionone | 194.35 | 3.24 | 0 | 1 | 1.35 | 0.05 | 0.27 | 17.07 | 3 |
| *Rhizoma Heterosmilacis* | Cedar acid | 198.19 | 1.13 | 2 | 5 | 0.5 | 0.06 | 0 | 75.99 | 3 |
| *Rhizoma Heterosmilacis* | n-butyl-β-D-fructopyronoside | 236.3 | -0.83 | 4 | 6 | -0.56 | 0.08 | 0.21 | 99.38 | 5 |
| *Rhizoma Heterosmilacis* | n-butyl-β-D-fructoufranoside | 236.3 | -0.83 | 4 | 6 | -0.78 | 0.08 | 0.25 | 99.38 | 6 |
| *Rhizoma Heterosmilacis* | ZINC00968101 | 154.28 | 1.98 | 1 | 1 | 1.27 | 0.05 | 0.24 | 20.23 | 0 |
| *Rhizoma Heterosmilacis* | Dioscin | 869.17 | 1.17 | 8 | 16 | -2.19 | 0.06 | 0.21 | 235.68 | 7 |
| *Rhizoma Heterosmilacis* | Smilagenin | 416.71 | 4.88 | 1 | 3 | 0.73 | 0.81 | 0.19 | 38.69 | 0 |
| *Rhizoma Heterosmilacis* | Syrionylglycerol-beta-syringaresinol | 969.05 | -1.2 | 10 | 23 | -2.86 | 0.09 | 0 | 322.29 | 19 |
| *Rhizoma Heterosmilacis* | Syrionylglycerol-beta-syringaresinol_qt | 644.73 | 2.61 | 4 | 13 | -0.56 | 0.6 | 0 | 163.99 | 13 |
| *Rhizoma Heterosmilacis* | ringenin | 272.27 | 2.3 | 3 | 5 | 0.28 | 0.21 | 0.4 | 86.99 | 1 |
| *Rhizoma Heterosmilacis* | isoengelitin | 434.43 | 0.89 | 6 | 10 | -0.91 | 0.7 | 0.35 | 166.14 | 3 |
| *Rhizoma Heterosmilacis* | Aromadedrin | 288.27 | 1.75 | 4 | 6 | -0.08 | 0.24 | 0.43 | 107.22 | 1 |
| *Rhizoma Heterosmilacis* | engeletin | 434.43 | 0.89 | 6 | 10 | -1.01 | 0.7 | 0.37 | 166.14 | 3 |
| *Rhizoma Heterosmilacis* | astilbin | 450.43 | 0.63 | 7 | 11 | -1.29 | 0.74 | 0.37 | 186.37 | 3 |
| *Rhizoma Heterosmilacis* | taxifolin | 304.27 | 1.49 | 5 | 7 | -0.23 | 0.27 | 0.39 | 127.45 | 1 |
| *Rhizoma Heterosmilacis* | cis-Dihydroquercetin | 304.27 | 1.49 | 5 | 7 | -0.34 | 0.27 | 0.4 | 127.45 | 1 |
| *Rhizoma Heterosmilacis* | alpha-Eudesmol | 222.41 | 3.67 | 1 | 1 | 1.31 | 0.1 | 0.25 | 20.23 | 1 |
| *Rhizoma Heterosmilacis* | none | 128.29 | 4.48 | 0 | 0 | 1.75 | 0.01 | 0.19 | 0 | 6 |
| *Rhizoma Heterosmilacis* | SKM | 174.17 | -1.18 | 4 | 5 | -1.16 | 0.04 | 0.32 | 97.99 | 1 |
| *Rhizoma Heterosmilacis* | Palmitone | 450.93 | 12.95 | 0 | 1 | 1.57 | 0.48 | 0.16 | 17.07 | 28 |
| *Rhizoma Heterosmilacis* | (-)-epicatechin | 290.29 | 1.92 | 5 | 6 | -0.03 | 0.24 | 0.34 | 110.38 | 1 |
| *Rhizoma Heterosmilacis* | β-terpineol | 154.28 | 2.47 | 1 | 1 | 1.19 | 0.03 | 0.29 | 20.23 | 1 |
| *Rhizoma Heterosmilacis* | n-butyl-α-D-fructoufranoside | 236.3 | -0.83 | 4 | 6 | -0.85 | 0.08 | 0.21 | 99.38 | 6 |
| *Rhizoma Heterosmilacis* | Dihydroresveratrol | 230.28 | 3.46 | 3 | 3 | 0.81 | 0.11 | 0.4 | 60.69 | 3 |
| *Rhizoma Heterosmilacis* | Tannin | 1,701.27 | 8.18 | 25 | 46 | -6.1 | 0.03 | 0.49 | 777.98 | 31 |
| *Rhizoma Heterosmilacis* | 2H-3,9a-Methano-1-benzoxepin-9-methanol, octahydro-2,2,5a-trimethyl-, (3R-(3alpha,5aalpha,9alpha,9aalpha))- | 238.41 | 2.45 | 1 | 2 | 1.01 | 0.14 | 0.2 | 29.46 | 1 |
| *Rhizoma Heterosmilacis* | resveratrol | 228.26 | 3.01 | 3 | 3 | 0.8 | 0.11 | 0.49 | 60.69 | 2 |
| *Rhizoma Heterosmilacis* | 3-O-caffeoylshikimic acid | 336.32 | 0.8 | 5 | 8 | -0.81 | 0.3 | 0.38 | 144.52 | 5 |
| *Rhizoma Heterosmilacis* | 4,7-Dihydroxy-5-methoxyl-6-methyl-8-formyl-flavan | 314.36 | 2.69 | 2 | 5 | 0.48 | 0.28 | 0.3 | 75.99 | 3 |
| *Rhizoma Heterosmilacis* | Neoastilbin | 450.43 | 0.63 | 7 | 11 | -1.07 | 0.74 | 0.34 | 186.37 | 3 |
| *Rhizoma Heterosmilacis* | Enhydrin | 464.51 | 1.37 | 0 | 10 | -0.36 | 0.74 | 0.36 | 130.26 | 7 |
| *Rhizoma Heterosmilacis* | octacosal | 408.84 | 11.89 | 0 | 1 | 1.56 | 0.42 | 0.14 | 17.07 | 26 |
| *Rhizoma Heterosmilacis* | [(2S,3S,4R,5R)-2-[(2R,3R,4S,5S,6R)-3-acetyloxy-6-(acetyloxymethyl)-4,5-dihydroxyoxan-2-yl]oxy-4-hydroxy-2,5-bis[[(E)-3-(4-hydroxy-3-methoxyphenyl)prop-2-enoyl]oxymethyl]oxolan-3-yl] (E)-3-(4-hydroxy-3-methoxyphenyl)prop-2-enoate | 954.96 | 3.13 | 6 | 22 | -2.17 | 0.2 | 0.31 | 308.26 | 24 |
| *Rhizoma Heterosmilacis* | Smiglaside C | 820.82 | 1.28 | 5 | 20 | -1.86 | 0.31 | 0.27 | 278.8 | 21 |
| *Rhizoma Heterosmilacis* | Smiglaside D | 966.97 | 3.53 | 5 | 22 | -1.93 | 0.19 | 0.32 | 305.1 | 25 |
| *Rhizoma Heterosmilacis* | Smiglaside E | 924.93 | 3.15 | 6 | 21 | -2.06 | 0.22 | 0.33 | 299.03 | 23 |
| *Rhizoma Heterosmilacis* | Sodium tauropythocholate | 514.78 | 1.19 | 4 | 8 | -0.68 | 0.86 | 0.05 | 155.37 | 7 |
| *Rhizoma Heterosmilacis* | Tulipane | 98.11 | 0.75 | 0 | 2 | 1.1 | 0.01 | 0.34 | 26.3 | 0 |
| *Rhizoma Heterosmilacis* | isoastilbin | 450.43 | 0.63 | 7 | 11 | -1.19 | 0.74 | 0.37 | 186.37 | 3 |
| *Rhizoma Heterosmilacis* | (2R,3R)-2-(3,5-dihydroxyphenyl)-3,5,7-trihydroxychroman-4-one | 304.27 | 1.49 | 5 | 7 | -0.34 | 0.27 | 0.39 | 127.45 | 1 |
| *Rhizoma Heterosmilacis* | Isoeruboside_B | 1,081.35 | -3.27 | 14 | 24 | -3.93 | 0.02 | 0.21 | 375.52 | 12 |
| *Rhizoma Heterosmilacis* | Isoeruboside_B_qt | 432.71 | 3.72 | 2 | 4 | 0.12 | 0.79 | 0.22 | 58.92 | 0 |
