# Supplementary material for: A System Pharmacology Model for Decoding the Synergistic Mechanisms of Compound Kushen Injection in Treating Breast Cancer
Source: Front Pharmacol. 2021 Nov 16;12:723147. doi: 10.3389/fphar.2021.723147 (PMC8660088; doi:10.3389/fphar.2021.723147)
Supplement: Supplementary file 10 [file Table4.DOCX]

**Table S4 |** The active components detailed information of Radix Sophorae Flavescentis and Rhizoma Heterosmilacis

| **Herb** | **Label** | **molecule_name** | **MW** | **AlogP** | **Caco-2** | **nHDon** | **nHAcc** | **DL** | **RBN** | **TPSA** | **FASA-** |
| --- | --- | --- | --- | --- | --- | --- | --- | --- | --- | --- | --- |
| *Radix Sophorae Flavescentis* | KS1 | luteolin | 286.25 | 2.067 | 0.185 | 4 | 6 | 0.24552 | 1 | 111.13 | 0.393265 |
| *Radix Sophorae Flavescentis* | KS2 | apigenin | 270.25 | 2.334 | 0.4256 | 3 | 5 | 0.21306 | 1 | 90.9 | 0.408862 |
| *Radix Sophorae Flavescentis* | KS3 | quercetin | 302.25 | 1.504 | 0.04842 | 5 | 7 | 0.27525 | 1 | 131.36 | 0.384534 |
| *Radix Sophorae Flavescentis* | KS4 | formononetin | 268.28 | 2.583 | 0.78264 | 1 | 4 | 0.21202 | 2 | 59.67 | 0 |
| *Radix Sophorae Flavescentis* | KS5 | Phaseolin | 322.38 | 3.461 | 1.08623 | 1 | 4 | 0.72891 | 0 | 47.92 | 0.332172 |
| *Radix Sophorae Flavescentis* | KS6 | Kushenol E | 424.53 | 5.743 | 0.5766 | 4 | 6 | 0.59003 | 5 | 107.22 | 0.321476 |
| *Radix Sophorae Flavescentis* | KS7 | (2R)-5,7-dihydroxy-2-(4-hydroxyphenyl)chroman-4-one | 272.27 | 2.298 | 0.37818 | 3 | 5 | 0.21141 | 1 | 86.99 | 0.408272 |
| *Radix Sophorae Flavescentis* | KS8 | Inermine | 284.28 | 2.442 | 0.89459 | 1 | 5 | 0.53754 | 0 | 57.15 | 0.299921 |
| *Radix Sophorae Flavescentis* | KS9 | hyperforin | 536.87 | 8.618 | 0.86693 | 1 | 4 | 0.598 | 11 | 71.44 | 0 |
| *Radix Sophorae Flavescentis* | KS10 | 8-Isopentenyl-kaempferol | 354.38 | 3.628 | 0.53297 | 4 | 6 | 0.3948 | 3 | 111.13 | 0 |
| *Radix Sophorae Flavescentis* | KS11 | sophocarpine | 246.39 | 1.394 | 0.99031 | 0 | 3 | 0.25078 | 0 | 23.55 | 0.260445 |
| *Radix Sophorae Flavescentis* | KS12 | Soyasapogenol B | 458.8 | 5.111 | 0.42544 | 3 | 3 | 0.74754 | 1 | 60.69 | 0.205596 |
| *Radix Sophorae Flavescentis* | KS13 | Inermin | 284.28 | 2.442 | 0.91157 | 1 | 5 | 0.53754 | 0 | 57.15 | 0.302024 |
| *Radix Sophorae Flavescentis* | KS14 | Wighteone | 338.38 | 3.921 | 0.63938 | 3 | 5 | 0.3622 | 3 | 90.9 | 0.311914 |
| *Radix Sophorae Flavescentis* | KS15 | Sophoramine | 244.37 | 1.145 | 1.42719 | 0 | 3 | 0.25182 | 0 | 25.24 | 0.222626 |
| *Radix Sophorae Flavescentis* | KS16 | sophoridine | 248.41 | 1.418 | 1.12698 | 0 | 3 | 0.24941 | 0 | 23.55 | 0.178768 |
| *Radix Sophorae Flavescentis* | KS17 | cis-Dihydroquercetin | 304.27 | 1.485 | -0.3396 | 5 | 7 | 0.27344 | 1 | 127.45 | 0.395924 |
| *Radix Sophorae Flavescentis* | KS18 | (2R)-7-hydroxy-2-(4-hydroxyphenyl)chroman-4-one | 256.27 | 2.565 | 0.41458 | 2 | 4 | 0.18303 | 1 | 66.76 | 0 |
| *Radix Sophorae Flavescentis* | KS19 | 5,7-dihydroxy-2-(3-hydroxy-4-methoxyphenyl)chroman-4-one | 302.3 | 2.281 | 0.27541 | 3 | 6 | 0.27226 | 2 | 96.22 | 0.31149 |
| *Radix Sophorae Flavescentis* | KS20 | matrine | 248.41 | 1.418 | 1.38831 | 0 | 3 | 0.24931 | 0 | 23.55 | 0 |
| *Radix Sophorae Flavescentis* | KS21 | (+)-14alpha-hydroxymatrine | 264.41 | 0.737 | 0.52962 | 1 | 4 | 0.28667 | 0 | 43.78 | 0.205063 |
| *Radix Sophorae Flavescentis* | KS22 | (+)-7,11-dehydromatrine,(leontalbinine) | 246.39 | 1.424 | 1.06311 | 0 | 3 | 0.2502 | 0 | 23.55 | 0.164645 |
| *Radix Sophorae Flavescentis* | KS23 | (+)-9alpha-hydroxymatrine | 264.41 | 0.451 | 0.61088 | 1 | 4 | 0.28641 | 0 | 43.78 | 0.191903 |
| *Radix Sophorae Flavescentis* | KS24 | (+)-allomatrine | 248.41 | 1.418 | 1.08121 | 0 | 3 | 0.24941 | 0 | 23.55 | 0.183574 |
| *Radix Sophorae Flavescentis* | KS25 | AIDS211310 | 248.41 | 1.418 | 1.14983 | 0 | 3 | 0.24936 | 0 | 23.55 | 0.173407 |
| *Radix Sophorae Flavescentis* | KS26 | (+)-lehmannine | 246.39 | 1.108 | 1.21374 | 0 | 3 | 0.25065 | 0 | 23.55 | 0.212346 |
| *Radix Sophorae Flavescentis* | KS27 | (+)-sophoranol | 264.41 | 0.668 | 0.43989 | 1 | 4 | 0.28186 | 0 | 43.78 | 0.200318 |
| *Radix Sophorae Flavescentis* | KS28 | isosophocarpine | 246.39 | 1.394 | 1.38618 | 0 | 3 | 0.25081 | 0 | 23.55 | 0.239272 |
| *Radix Sophorae Flavescentis* | KS29 | (-)-14beta-hydroxymatrine | 264.41 | 0.737 | 0.77024 | 1 | 4 | 0.28667 | 0 | 43.78 | 0.21087 |
| *Radix Sophorae Flavescentis* | KS30 | (-)-9alpha-hydroxysophoramine | 262.39 | 0.427 | 0.38227 | 1 | 4 | 0.2882 | 0 | 43.78 | 0.249694 |
| *Radix Sophorae Flavescentis* | KS31 | anagyrine | 244.37 | 1.145 | 1.15965 | 0 | 3 | 0.24338 | 0 | 25.24 | 0 |
| *Radix Sophorae Flavescentis* | KS32 | 1,4-diazaindan-type,alkaloid,flavascensine | 348.64 | 5.395 | 1.12965 | 2 | 3 | 0.24095 | 10 | 41.13 | 0.233207 |
| *Radix Sophorae Flavescentis* | KS33 | 13,14-dehydrosophoridine | 246.39 | 1.394 | 1.05819 | 0 | 3 | 0.25078 | 0 | 23.55 | 0.239704 |
| *Radix Sophorae Flavescentis* | KS34 | 2-n-hencosyl-5,7-dihydroxy-6,8-dimethylchromone | 500.84 | 11.531 | 1.23857 | 2 | 4 | 0.66273 | 20 | 70.67 | 0.179026 |
| *Radix Sophorae Flavescentis* | KS35 | 2-n-heptadecyl-5,7-dihydroxy-6,8-dimethyl chromone | 444.72 | 9.706 | 1.18424 | 2 | 4 | 0.6547 | 16 | 70.67 | 0.186674 |
| *Radix Sophorae Flavescentis* | KS36 | 2-n-nonadecyl-5,7-dihydroxy-6,8-dimethyl chromone | 472.78 | 10.619 | 1.20333 | 2 | 4 | 0.68402 | 18 | 70.67 | 0.18293 |
| *Radix Sophorae Flavescentis* | KS37 | 2-n-pentacosyl-5,7-dihydroxy-6,8-dimethyl chromone | 556.96 | 13.356 | 1.30973 | 2 | 4 | 0.52736 | 24 | 70.67 | 0.169319 |
| *Radix Sophorae Flavescentis* | KS38 | 2-n-pentadecyl-5,7-dihydroxy-6,8-dimethyl chromone | 416.66 | 8.794 | 1.15816 | 2 | 4 | 0.58048 | 14 | 70.67 | 0.199435 |
| *Radix Sophorae Flavescentis* | KS39 | 2-n-tricosyl-5,7-dihydroxy-6,8-dimethyl chromone | 528.9 | 12.443 | 1.26627 | 2 | 4 | 0.6038 | 22 | 70.67 | 0.175546 |
| *Radix Sophorae Flavescentis* | KS40 | 2-n-tridecyl-5,7-dihydroxy-6,8-dimethyl chromone | 388.6 | 7.881 | 1.15523 | 2 | 4 | 0.48219 | 12 | 70.67 | 0.201112 |
| *Radix Sophorae Flavescentis* | KS41 | 5α,9α-dihydroxymatrine | 280.41 | -0.299 | 0.04071 | 2 | 5 | 0.32076 | 0 | 64.01 | 0.199359 |
| *Radix Sophorae Flavescentis* | KS42 | 7,11-dehydromatrine | 246.39 | 1.424 | 1.11497 | 0 | 3 | 0.2503 | 0 | 23.55 | 0.163397 |
| *Radix Sophorae Flavescentis* | KS43 | 9alpha-hydroxy-7,11-dehydromatrine | 262.39 | 0.457 | 0.35029 | 1 | 4 | 0.28753 | 0 | 43.78 | 0.1769 |
| *Radix Sophorae Flavescentis* | KS44 | Kushenol I | 438.51 | 5.037 | 0.44306 | 5 | 7 | 0.63482 | 6 | 131.36 | 0.348095 |
| *Radix Sophorae Flavescentis* | KS45 | Kushenol M | 522.69 | 7.125 | 0.35142 | 4 | 7 | 0.77228 | 9 | 116.45 | 0.305069 |
| *Radix Sophorae Flavescentis* | KS46 | N-allomatrine | 248.41 | 1.418 | 0.99458 | 0 | 3 | 0.24939 | 0 | 23.55 | 0.175239 |
| *Radix Sophorae Flavescentis* | KS47 | N-oxysophocarpine | 262.39 | -0.857 | 1.02331 | 0 | 3 | 0.28605 | 0 | 37.38 | 0.051418 |
| *Radix Sophorae Flavescentis* | KS48 | 7,14-Methano-4H,6H-dipyrido(1,2-a:1',2'-e)(1,5)diazocin-4-one, 7,7a,8,9,10,11,13,14-octahydro-9-hydroxy-, (7R-(7alpha,7abeta,9alpha,14alpha))- | 260.37 | -0.34 | 0.41726 | 1 | 4 | 0.28203 | 0 | 45.47 | 0.215444 |
| *Radix Sophorae Flavescentis* | KS49 | Thc-9-cooh | 358.52 | 5.689 | 0.89294 | 2 | 4 | 0.42801 | 5 | 66.76 | 0.266268 |
| *Radix Sophorae Flavescentis* | KS50 | Deoxyhumulone | 346.51 | 5.856 | 0.78529 | 3 | 4 | 0.21545 | 7 | 77.76 | 0.296687 |
| *Radix Sophorae Flavescentis* | KS51 | Glyceollin | 338.38 | 2.847 | 0.53096 | 2 | 5 | 0.75964 | 0 | 68.15 | 0.350383 |
| *Radix Sophorae Flavescentis* | KS52 | 3,4',5-Trihydroxy-7-methoxy-8-isopente-nylflavone | 368.41 | 3.879 | 0.56413 | 3 | 6 | 0.42634 | 4 | 100.13 | 0.334728 |
| *Radix Sophorae Flavescentis* | KS53 | 1-[2,4-dihydroxy-3-(3-methylbut-2-enyl)phenyl]-3-phenylprop-2-en-1-one | 308.4 | 5.024 | 1.03648 | 2 | 3 | 0.22756 | 5 | 57.53 | 0.418104 |
| *Radix Sophorae Flavescentis* | KS54 | isokurarinone | 438.56 | 5.814 | 0.56037 | 3 | 6 | 0.65898 | 7 | 96.22 | 0.307169 |
| *Radix Sophorae Flavescentis* | KS55 | (2S)-7-hydroxy-2-(4-hydroxyphenyl)-5-methoxy-8-(3-methylbut-2-enyl)chroman-4-one | 354.43 | 4.405 | 0.79598 | 2 | 5 | 0.38968 | 4 | 75.99 | 0.300253 |
| *Radix Sophorae Flavescentis* | KS56 | kosamol,q | 454.61 | 6.671 | 0.69833 | 4 | 6 | 0.68605 | 8 | 107.22 | 0.329571 |
| *Radix Sophorae Flavescentis* | KS57 | kosamol,r | 450.62 | 6.886 | 1.12694 | 2 | 5 | 0.66975 | 9 | 75.99 | 0.313996 |
| *Radix Sophorae Flavescentis* | KS58 | kurarainone | 438.56 | 5.814 | 0.67282 | 3 | 6 | 0.63262 | 7 | 96.22 | 0.319861 |
| *Radix Sophorae Flavescentis* | KS59 | kuraridin | 438.56 | 6.149 | 0.47176 | 4 | 6 | 0.52683 | 9 | 107.22 | 0.338788 |
| *Radix Sophorae Flavescentis* | KS60 | kuraridine | 494.63 | 5.953 | 0.24775 | 5 | 7 | 0.75281 | 9 | 127.45 | 0.329033 |
| *Radix Sophorae Flavescentis* | KS61 | kuraridinol | 472.58 | 4.484 | -0.37 | 6 | 8 | 0.57942 | 10 | 147.68 | 0.33311 |
| *Radix Sophorae Flavescentis* | KS62 | kurarinol | 456.58 | 4.713 | 0.11518 | 4 | 7 | 0.67075 | 8 | 116.45 | 0.300877 |
| *Radix Sophorae Flavescentis* | KS63 | kurarinone | 424.53 | 5.693 | 0.464 | 4 | 6 | 0.48925 | 8 | 107.22 | 0.336518 |
| *Radix Sophorae Flavescentis* | KS64 | kushenin | 286.3 | 2.39 | 0.71377 | 2 | 5 | 0.38034 | 1 | 68.15 | 0.272668 |
| *Radix Sophorae Flavescentis* | KS65 | kushenol A | 408.53 | 5.83 | 0.66514 | 3 | 5 | 0.54728 | 6 | 86.99 | 0.334826 |
| *Radix Sophorae Flavescentis* | KS66 | kushenol B | 492.66 | 7.42 | 0.61056 | 4 | 6 | 0.74758 | 8 | 107.22 | 0.308843 |
| *Radix Sophorae Flavescentis* | KS67 | kushenol D | 452.59 | 6.4 | 0.63221 | 3 | 6 | 0.56601 | 10 | 96.22 | 0.29864 |
| *Radix Sophorae Flavescentis* | KS68 | kushenol F | 424.53 | 5.563 | 0.45138 | 4 | 6 | 0.61318 | 6 | 107.22 | 0.328249 |
| *Radix Sophorae Flavescentis* | KS69 | KushenolG | 456.53 | 3.935 | -0.2766 | 6 | 8 | 0.67137 | 7 | 151.59 | 0.328424 |
| *Radix Sophorae Flavescentis* | KS70 | kushenol J_qt | 286.3 | 2.271 | 0.24172 | 2 | 5 | 0.23654 | 2 | 75.99 | 0.36699 |
| *Radix Sophorae Flavescentis* | KS71 | (2R,3R)-2-(2,4-dihydroxyphenyl)-3,7-dihydroxy-8-[(2R)-2-isopropenyl-5-methylhex-4-enyl]-5-methoxy-4-chromanone | 454.56 | 5.269 | 0.10573 | 4 | 7 | 0.66295 | 7 | 116.45 | 0.300719 |
| *Radix Sophorae Flavescentis* | KS72 | kushenol,t | 442.55 | 4.462 | -0.04639 | 5 | 7 | 0.64125 | 7 | 127.45 | 0.350284 |
| *Radix Sophorae Flavescentis* | KS73 | leachianone,a | 438.56 | 5.814 | 0.6283 | 3 | 6 | 0.64663 | 7 | 96.22 | 0.306968 |
| *Radix Sophorae Flavescentis* | KS74 | leachianone,g | 356.4 | 3.887 | 0.32824 | 4 | 6 | 0.40307 | 3 | 107.22 | 0.367275 |
| *Radix Sophorae Flavescentis* | KS75 | Lehmanine | 246.39 | 1.108 | 1.18426 | 0 | 3 | 0.25068 | 0 | 23.55 | 0.209863 |
| *Radix Sophorae Flavescentis* | KS76 | (+)-Lupanine | 248.41 | 1.418 | 1.1622 | 0 | 3 | 0.2408 | 0 | 23.55 | 0.17618 |
| *Radix Sophorae Flavescentis* | KS77 | Norartocarpetin | 286.25 | 2.067 | 0.14164 | 4 | 6 | 0.24494 | 1 | 111.13 | 0.419489 |
| *Radix Sophorae Flavescentis* | KS78 | (2R)-2-(2,4-dihydroxyphenyl)-5,7-dihydroxy-8-[(2S)-2-isopropenyl-5-methylhex-4-enyl]-4-chromanone | 424.53 | 5.563 | 0.52416 | 4 | 6 | 0.60031 | 6 | 107.22 | 0.346687 |
| *Radix Sophorae Flavescentis* | KS79 | (2S)-2-(3,4-dihydroxyphenyl)-6-[(2E)-3,7-dimethylocta-2,6-dienyl]-5,7-dihydroxychroman-4-one | 424.53 | 5.714 | 0.47801 | 4 | 6 | 0.6514 | 6 | 107.22 | 0.336382 |
| *Radix Sophorae Flavescentis* | KS80 | oxymatrine | 264.41 | -0.834 | 1.00266 | 0 | 3 | 0.28431 | 0 | 37.38 | 0.026657 |
| *Radix Sophorae Flavescentis* | KS81 | oxysophocarpine | 262.39 | -0.857 | 1.03962 | 0 | 3 | 0.28604 | 0 | 37.38 | 0.026965 |
| *Radix Sophorae Flavescentis* | KS82 | Pterocarpine | 298.31 | 2.693 | 1.1173 | 0 | 5 | 0.59932 | 1 | 46.15 | 0.251539 |
| *Radix Sophorae Flavescentis* | KS83 | sokurarinone | 450.62 | 6.886 | 0.76885 | 2 | 5 | 0.71488 | 9 | 75.99 | 0.332413 |
| *Radix Sophorae Flavescentis* | KS84 | sophoraflavanone,g | 410.5 | 5.442 | 0.27411 | 5 | 6 | 0.46315 | 7 | 118.22 | 0.387412 |
| *Radix Sophorae Flavescentis* | KS85 | sophoraflavosideⅢ_qt | 488.78 | 4.229 | -0.33037 | 4 | 5 | 0.71814 | 2 | 97.99 | 0.222558 |
| *Radix Sophorae Flavescentis* | KS86 | sophoraflavoside IV_qt | 488.78 | 4.229 | -0.35576 | 4 | 5 | 0.71811 | 2 | 97.99 | 0.240134 |
| *Radix Sophorae Flavescentis* | KS87 | sophoraisoflavanone,a | 398.49 | 4.825 | 0.52016 | 3 | 6 | 0.5223 | 6 | 96.22 | 0.319861 |
| *Radix Sophorae Flavescentis* | KS88 | sophoranol N-oxide | 280.41 | -1.583 | 0.59372 | 1 | 4 | 0.31705 | 0 | 57.61 | 0.029234 |
| *Radix Sophorae Flavescentis* | KS89 | sophranol | 264.41 | 0.668 | 0.59561 | 1 | 4 | 0.28192 | 0 | 43.78 | 0.190065 |
| *Radix Sophorae Flavescentis* | KS91 | Trifolirhizin | 446.44 | 0.538 | -0.82995 | 4 | 10 | 0.78562 | 3 | 136.3 | 0.281654 |
| *Radix Sophorae Flavescentis* | KS90 | xanthohumol | 368.46 | 5.089 | 0.67598 | 3 | 5 | 0.34712 | 7 | 86.99 | 0.344723 |
| *Radix Sophorae Flavescentis* | KS92 | N-Methylcytisine | N/A | N/A | N/A | N/A | N/A | N/A | N/A | N/A | N/A |
| *Rhizoma Heterosmilacis* | BTL1 | quercetin | 302.25 | 1.504 | 0.04842 | 5 | 7 | 0.27525 | 1 | 131.36 | 0.384534 |
| *Rhizoma Heterosmilacis* | BTL2 | Sitogluside | 576.95 | 6.337 | -0.1362 | 4 | 6 | 0.6241 | 9 | 99.38 | 0.227022 |
| *Rhizoma Heterosmilacis* | BTL3 | beta-sitosterol | 414.79 | 8.084 | 1.32463 | 1 | 1 | 0.75123 | 6 | 20.23 | 0.225506 |
| *Rhizoma Heterosmilacis* | BTL4 | sitosterol | 414.79 | 8.084 | 1.32059 | 1 | 1 | 0.7512 | 6 | 20.23 | 0.224795 |
| *Rhizoma Heterosmilacis* | BTL5 | Stigmasterol | 412.77 | 7.64 | 1.44458 | 1 | 1 | 0.75665 | 5 | 20.23 | 0.216794 |
| *Rhizoma Heterosmilacis* | BTL6 | diosgenin | 414.69 | 4.634 | 0.81998 | 1 | 3 | 0.80979 | 0 | 38.69 | 0.190773 |
| *Rhizoma Heterosmilacis* | BTL7 | (2S,3R)-3,5,7-trihydroxy-2-(4-hydroxyphenyl)chroman-4-one | 288.27 | 1.752 | -0.10456 | 4 | 6 | 0.23898 | 1 | 107.22 | 0.4073 |
| *Rhizoma Heterosmilacis* | BTL8 | Tricosane | 324.71 | 10.864 | 1.84718 | 0 | 0 | 0.2088 | 20 | 0 | 0.127646 |
| *Rhizoma Heterosmilacis* | BTL9 | (-)-taxifolin | 304.27 | 1.485 | -0.24278 | 5 | 7 | 0.27342 | 1 | 127.45 | 0.408426 |
| *Rhizoma Heterosmilacis* | BTL10 | Smilagenin | 416.71 | 4.884 | 0.73335 | 1 | 3 | 0.80715 | 0 | 38.69 | 0.193609 |
| *Rhizoma Heterosmilacis* | BTL11 | octacosanal | 272.27 | 2.298 | 0.28421 | 3 | 5 | 0.21128 | 1 | 86.99 | 0.395873 |
| *Rhizoma Heterosmilacis* | BTL12 | Aromadedrin | 288.27 | 1.752 | -0.0751 | 4 | 6 | 0.23896 | 1 | 107.22 | 0.426497 |
| *Rhizoma Heterosmilacis* | BTL13 | taxifolin | 304.27 | 1.485 | -0.22844 | 5 | 7 | 0.27345 | 1 | 127.45 | 0.390146 |
| *Rhizoma Heterosmilacis* | BTL14 | cis-Dihydroquercetin | 304.27 | 1.485 | -0.3396 | 5 | 7 | 0.27344 | 1 | 127.45 | 0.395924 |
| *Rhizoma Heterosmilacis* | BTL15 | Palmitone | 450.93 | 12.951 | 1.56725 | 0 | 1 | 0.47535 | 28 | 17.07 | 0.162234 |
| *Rhizoma Heterosmilacis* | BTL16 | (-)-epicatechin | 290.29 | 1.92 | -0.03279 | 5 | 6 | 0.24163 | 1 | 110.38 | 0.343654 |
| *Rhizoma Heterosmilacis* | BTL17 | 4,7-Dihydroxy-5-methoxyl-6-methyl-8-formyl-flavan | 314.36 | 2.693 | 0.48404 | 2 | 5 | 0.27872 | 3 | 75.99 | 0.301456 |
| *Rhizoma Heterosmilacis* | BTL18 | Enhydrin | 464.51 | 1.367 | -0.3569 | 0 | 10 | 0.73615 | 7 | 130.26 | 0.356262 |
| *Rhizoma Heterosmilacis* | BTL19 | octacosanal | 408.84 | 11.889 | 1.56086 | 0 | 1 | 0.42297 | 26 | 17.07 | 0.139732 |
| *Rhizoma Heterosmilacis* | BTL20 | (2R,3R)-2-(3,5-dihydroxyphenyl)-3,5,7-trihydroxychroman-4-one | 304.27 | 1.485 | -0.34301 | 5 | 7 | 0.27209 | 1 | 127.45 | 0.393719 |
| *Rhizoma Heterosmilacis* | BTL21 | Isoeruboside_B_qt | 432.71 | 3.715 | 0.11768 | 2 | 4 | 0.79012 | 0 | 58.92 | 0.220859 |

N/A, not applicable.
