# Supplementary material for: A System Pharmacology Model for Decoding the Synergistic Mechanisms of Compound Kushen Injection in Treating Breast Cancer
Source: Front Pharmacol. 2021 Nov 16;12:723147. doi: 10.3389/fphar.2021.723147 (PMC8660088; doi:10.3389/fphar.2021.723147)
Supplement: Supplementary file 11 [file Table6.DOCX]

**Table S6 |** The result of SCD values of each component based on mathematical formula.

| **Components** | | **Ci** | **CB/TB** | **Cbi-Cki/Cbi+Cki** | **Tj** | **NORM(di)** | **SCD** | **SCD/SUMSCD** |
| --- | --- | --- | --- | --- | --- | --- | --- | --- |
| KS80 | 0.01560 | | 0.07365 | 1.00000 | 0.12963 | 1.00000 | 1.00217 | 0.34035 |
| KS20 | 0.00410 | | 0.04835 | 1.00000 | 0.33597 | 0.63944 | 0.64088 | 0.21765 |
| KS81 | 0.00848 | | 0.06886 | 1.00000 | 0.12720 | 0.31670 | 0.31785 | 0.10795 |
| KS16 | 0.00386 | | 0.05780 | 1.00000 | 0.12873 | 0.19596 | 0.19649 | 0.06673 |
| KS11 | 0.00367 | | 0.06343 | 1.00000 | 0.10028 | 0.18856 | 0.18895 | 0.06417 |
| BTL15 | 0.17779 | | 0.18885 | 1.00000 | 0.37488 | 0.00000 | 0.07923 | 0.02691 |
| KS92 | 0.01179 | | 0.12139 | 1.00000 | 0.04337 | 0.04739 | 0.04797 | 0.01629 |
| KS4 | 0.07045 | | 0.07000 | 1.00000 | 0.54806 | 0.00000 | 0.04131 | 0.01403 |
| KS77 | 0.06571 | | 0.07670 | 1.00000 | 0.45973 | 0.00000 | 0.03252 | 0.01105 |
| KS2 | 0.04740 | | 0.06677 | 1.00000 | 0.60531 | 0.00000 | 0.03060 | 0.01039 |
| KS1 | 0.04331 | | 0.06987 | 1.00000 | 0.53076 | 0.00000 | 0.02459 | 0.00835 |
| KS53 | 0.06429 | | 0.08646 | 1.00000 | 0.33125 | 0.00000 | 0.02314 | 0.00786 |
| BTL3 | 0.04542 | | 0.09689 | 1.00000 | 0.35569 | 0.00000 | 0.01772 | 0.00602 |
| KS91 | 0.02860 | | 0.09425 | 1.00000 | 0.12472 | 0.01338 | 0.01728 | 0.00587 |
| BTL19 | 0.04901 | | 0.13367 | 1.00000 | 0.30796 | 0.00000 | 0.01711 | 0.00581 |
| BTL1 | 0.05904 | | 0.07734 | 0.42929 | 0.55716 | 0.00000 | 0.01667 | 0.00566 |
| KS3 | 0.05904 | | 0.07734 | 0.42929 | 0.55716 | 0.00000 | 0.01667 | 0.00566 |
| KS70 | 0.03296 | | 0.05984 | 1.00000 | 0.45116 | 0.00000 | 0.01576 | 0.00535 |
| KS90 | 0.04228 | | 0.07234 | 1.00000 | 0.33410 | 0.00000 | 0.01515 | 0.00514 |
| BTL4 | 0.04121 | | 0.10053 | 1.00000 | 0.32729 | 0.00000 | 0.01485 | 0.00504 |
| KS52 | 0.02301 | | 0.05270 | 1.00000 | 0.50724 | 0.00000 | 0.01229 | 0.00417 |
| KS19 | 0.02064 | | 0.04609 | 1.00000 | 0.45682 | 0.00000 | 0.00986 | 0.00335 |
| BTL8 | 0.04409 | | 0.16807 | 1.00000 | 0.18953 | 0.00000 | 0.00976 | 0.00332 |
| BTL2 | 0.04269 | | 0.10816 | 1.00000 | 0.19980 | 0.00000 | 0.00945 | 0.00321 |
| BTL5 | 0.02378 | | 0.08455 | 1.00000 | 0.30162 | 0.00000 | 0.00778 | 0.00264 |
| KS14 | 0.01661 | | 0.05292 | 1.00000 | 0.40292 | 0.00000 | 0.00705 | 0.00239 |
| KS10 | 0.01223 | | 0.05297 | 1.00000 | 0.39110 | 0.00000 | 0.00504 | 0.00171 |
| KS12 | 0.02167 | | 0.07246 | 1.00000 | 0.20868 | 0.00000 | 0.00485 | 0.00165 |
| BTL11 | 0.00979 | | 0.04364 | 1.00000 | 0.47282 | 0.00000 | 0.00483 | 0.00164 |
| KS79 | 0.01119 | | 0.03818 | 1.00000 | 0.39877 | 0.00000 | 0.00463 | 0.00157 |
| KS49 | 0.02082 | | 0.06510 | 1.00000 | 0.20765 | 0.00000 | 0.00461 | 0.00156 |
| BTL16 | 0.01496 | | 0.05258 | 1.00000 | 0.28257 | 0.00000 | 0.00445 | 0.00151 |
| KS7 | 0.00889 | | 0.04292 | 1.00000 | 0.47336 | 0.00000 | 0.00439 | 0.00149 |
| KS64 | 0.01611 | | 0.05792 | 1.00000 | 0.20735 | 0.00000 | 0.00353 | 0.00120 |
| BTL9 | 0.00751 | | 0.04811 | 1.00000 | 0.40308 | 0.00000 | 0.00317 | 0.00108 |
| KS69 | 0.01010 | | 0.04762 | 1.00000 | 0.28436 | 0.00000 | 0.00301 | 0.00102 |
| KS63 | 0.00944 | | 0.05109 | 1.00000 | 0.28563 | 0.00000 | 0.00283 | 0.00096 |
| BTL6 | 0.01529 | | 0.09744 | 1.00000 | 0.16716 | 0.00000 | 0.00281 | 0.00095 |
| BTL10 | 0.02847 | | 0.16733 | 1.00000 | 0.08414 | 0.00000 | 0.00280 | 0.00095 |
| BTL13 | 0.00867 | | 0.05304 | 1.00000 | 0.29094 | 0.00000 | 0.00266 | 0.00090 |
| KS35 | 0.00899 | | 0.05271 | 1.00000 | 0.27925 | 0.00000 | 0.00264 | 0.00090 |
| BTL7 | 0.00562 | | 0.04120 | 1.00000 | 0.44828 | 0.00000 | 0.00262 | 0.00089 |
| KS18 | 0.00579 | | 0.04181 | 1.00000 | 0.42570 | 0.00000 | 0.00257 | 0.00087 |
| KS9 | 0.01300 | | 0.05634 | 1.00000 | 0.17611 | 0.00000 | 0.00242 | 0.00082 |
| BTL12 | 0.00664 | | 0.04422 | 1.00000 | 0.34843 | 0.00000 | 0.00241 | 0.00082 |
| KS86 | 0.01302 | | 0.08466 | 1.00000 | 0.16790 | 0.00000 | 0.00237 | 0.00081 |
| BTL18 | 0.01805 | | 0.07576 | 1.00000 | 0.11922 | 0.00000 | 0.00232 | 0.00079 |
| KS85 | 0.01218 | | 0.08356 | 1.00000 | 0.16764 | 0.00000 | 0.00221 | 0.00075 |
| KS59 | 0.00707 | | 0.04404 | 1.00000 | 0.29780 | 0.00000 | 0.00220 | 0.00075 |
| KS50 | 0.00805 | | 0.04299 | 1.00000 | 0.24603 | 0.00000 | 0.00207 | 0.00070 |
| KS37 | 0.01062 | | 0.06981 | 1.00000 | 0.17065 | 0.00000 | 0.00194 | 0.00066 |
| KS38 | 0.00993 | | 0.06789 | 1.00000 | 0.18075 | 0.00000 | 0.00192 | 0.00065 |
| KS36 | 0.01008 | | 0.06446 | 1.00000 | 0.17727 | 0.00000 | 0.00190 | 0.00065 |
| KS67 | 0.00626 | | 0.04526 | 1.00000 | 0.27689 | 0.00000 | 0.00181 | 0.00062 |
| KS60 | 0.00850 | | 0.04460 | 1.00000 | 0.20131 | 0.00000 | 0.00179 | 0.00061 |
| KS82 | 0.01985 | | 0.10458 | 1.00000 | 0.07689 | 0.00000 | 0.00169 | 0.00057 |
| KS43 | 0.00905 | | 0.06320 | 1.00000 | 0.16830 | 0.00000 | 0.00162 | 0.00055 |
| KS5 | 0.00755 | | 0.05096 | 1.00000 | 0.20185 | 0.00000 | 0.00160 | 0.00054 |
| KS40 | 0.00810 | | 0.06740 | 1.00000 | 0.18076 | 0.00000 | 0.00156 | 0.00053 |
| KS44 | 0.00496 | | 0.04559 | 1.00000 | 0.29336 | 0.00000 | 0.00152 | 0.00052 |
| KS34 | 0.00731 | | 0.06036 | 1.00000 | 0.19512 | 0.00000 | 0.00151 | 0.00051 |
| KS51 | 0.00615 | | 0.04270 | 1.00000 | 0.22439 | 0.00000 | 0.00144 | 0.00049 |
| KS41 | 0.00826 | | 0.05797 | 1.00000 | 0.14998 | 0.00000 | 0.00131 | 0.00045 |
| KS30 | 0.00749 | | 0.05450 | 1.00000 | 0.16480 | 0.00000 | 0.00130 | 0.00044 |
| KS55 | 0.00383 | | 0.03564 | 1.00000 | 0.31897 | 0.00000 | 0.00127 | 0.00043 |
| KS21 | 0.00582 | | 0.06098 | 1.00000 | 0.19704 | 0.00000 | 0.00122 | 0.00041 |
| KS29 | 0.00582 | | 0.06098 | 1.00000 | 0.19704 | 0.00000 | 0.00122 | 0.00041 |
| KS74 | 0.00352 | | 0.03422 | 1.00000 | 0.31576 | 0.00000 | 0.00115 | 0.00039 |
| BTL20 | 0.00624 | | 0.05634 | 1.00000 | 0.17278 | 0.00000 | 0.00114 | 0.00039 |
| KS45 | 0.00398 | | 0.03647 | 1.00000 | 0.26676 | 0.00000 | 0.00110 | 0.00037 |
| KS39 | 0.00650 | | 0.07227 | 1.00000 | 0.15697 | 0.00000 | 0.00109 | 0.00037 |
| KS23 | 0.00516 | | 0.05882 | 1.00000 | 0.19974 | 0.00000 | 0.00109 | 0.00037 |
| KS6 | 0.00348 | | 0.03497 | 1.00000 | 0.30277 | 0.00000 | 0.00109 | 0.00037 |
| KS56 | 0.00344 | | 0.02976 | 1.00000 | 0.30640 | 0.00000 | 0.00108 | 0.00037 |
| KS65 | 0.00261 | | 0.02941 | 1.00000 | 0.36316 | 0.00000 | 0.00098 | 0.00033 |
| KS68 | 0.00237 | | 0.03001 | 1.00000 | 0.38351 | 0.00000 | 0.00093 | 0.00032 |
| KS88 | 0.00543 | | 0.04918 | 1.00000 | 0.16283 | 0.00000 | 0.00093 | 0.00032 |
| KS8 | 0.00415 | | 0.04376 | 1.00000 | 0.21231 | 0.00000 | 0.00092 | 0.00031 |
| KS13 | 0.00478 | | 0.05128 | 1.00000 | 0.17839 | 0.00000 | 0.00090 | 0.00031 |
| BTL14 | 0.00765 | | 0.04817 | 0.21155 | 0.40459 | 0.00000 | 0.00080 | 0.00027 |
| KS17 | 0.00765 | | 0.04817 | 0.21155 | 0.40459 | 0.00000 | 0.00080 | 0.00027 |
| KS54 | 0.00180 | | 0.02868 | 1.00000 | 0.38384 | 0.00000 | 0.00071 | 0.00024 |
| KS84 | 0.00280 | | 0.03811 | 1.00000 | 0.22365 | 0.00000 | 0.00065 | 0.00022 |
| KS66 | 0.00238 | | 0.03257 | 1.00000 | 0.26201 | 0.00000 | 0.00064 | 0.00022 |
| KS87 | 0.00187 | | 0.03009 | 1.00000 | 0.32934 | 0.00000 | 0.00064 | 0.00022 |
| BTL17 | 0.00247 | | 0.03689 | 1.00000 | 0.23969 | 0.00000 | 0.00061 | 0.00021 |
| KS32 | 0.00822 | | 0.07207 | 1.00000 | 0.06815 | 0.00000 | 0.00060 | 0.00020 |
| KS27 | 0.00309 | | 0.04696 | 1.00000 | 0.18313 | 0.00000 | 0.00059 | 0.00020 |
| KS89 | 0.00309 | | 0.04696 | 1.00000 | 0.18313 | 0.00000 | 0.00059 | 0.00020 |
| BTL21 | 0.00886 | | 0.15344 | 1.00000 | 0.05695 | 0.00000 | 0.00058 | 0.00020 |
| KS46 | 0.00387 | | 0.05293 | 1.00000 | 0.14186 | 0.00000 | 0.00058 | 0.00020 |
| KS24 | 0.00328 | | 0.04786 | 1.00000 | 0.16805 | 0.00000 | 0.00058 | 0.00020 |
| KS73 | 0.00234 | | 0.03388 | 1.00000 | 0.22365 | 0.00000 | 0.00054 | 0.00018 |
| KS83 | 0.00128 | | 0.02672 | 1.00000 | 0.34978 | 0.00000 | 0.00046 | 0.00016 |
| KS61 | 0.00220 | | 0.03908 | 1.00000 | 0.19648 | 0.00000 | 0.00045 | 0.00015 |
| KS62 | 0.00116 | | 0.02820 | 1.00000 | 0.33466 | 0.00000 | 0.00040 | 0.00014 |
| KS58 | 0.00170 | | 0.03258 | 1.00000 | 0.22381 | 0.00000 | 0.00039 | 0.00013 |
| KS57 | 0.00152 | | 0.03143 | 1.00000 | 0.24988 | 0.00000 | 0.00039 | 0.00013 |
| KS78 | 0.00165 | | 0.03242 | 1.00000 | 0.22419 | 0.00000 | 0.00038 | 0.00013 |
| KS48 | 0.00643 | | 0.09744 | 1.00000 | 0.05143 | 0.00000 | 0.00036 | 0.00012 |
| KS22 | 0.00652 | | 0.11184 | 1.00000 | 0.04578 | 0.00000 | 0.00033 | 0.00011 |
| KS76 | 0.00367 | | 0.05592 | 1.00000 | 0.08359 | 0.00000 | 0.00032 | 0.00011 |
| KS31 | 0.00637 | | 0.11050 | 1.00000 | 0.04362 | 0.00000 | 0.00031 | 0.00011 |
| KS72 | 0.00106 | | 0.03065 | 1.00000 | 0.27736 | 0.00000 | 0.00030 | 0.00010 |
| KS33 | 0.00296 | | 0.06375 | 1.00000 | 0.09144 | 0.00000 | 0.00029 | 0.00010 |
| KS42 | 0.00477 | | 0.09605 | 1.00000 | 0.05277 | 0.00000 | 0.00028 | 0.00009 |
| KS71 | 0.00175 | | 0.04034 | 1.00000 | 0.14519 | 0.00000 | 0.00027 | 0.00009 |
| KS25 | 0.00263 | | 0.05793 | 1.00000 | 0.08717 | 0.00000 | 0.00024 | 0.00008 |
| KS15 | 0.00447 | | 0.10497 | 1.00000 | 0.04532 | 0.00000 | 0.00022 | 0.00008 |
| KS75 | 0.00224 | | 0.06513 | 1.00000 | 0.09385 | 0.00000 | 0.00022 | 0.00008 |
| KS26 | 0.00277 | | 0.06773 | 1.00000 | 0.07182 | 0.00000 | 0.00021 | 0.00007 |
| KS28 | 0.00210 | | 0.06178 | 1.00000 | 0.09288 | 0.00000 | 0.00021 | 0.00007 |
| KS47 | 0.00160 | | 0.09554 | 1.00000 | 0.02696 | 0.00000 | 0.00005 | 0.00002 |
